# Merging Flow Synthesis and Enzymatic Maturation to Expand the Chemical Space of Lasso Peptides

**DOI:** 10.1021/jacs.4c03898

**Published:** 2024-05-17

**Authors:** Kevin Schiefelbein, Jakob Lang, Matthias Schuster, Claire E. Grigglestone, Robin Striga, Laurent Bigler, Meredith C. Schuman, Oliver Zerbe, Yanyan Li, Nina Hartrampf

**Affiliations:** † Department of Chemistry, University of Zurich, Winterthurerstrasse 190, 8057 Zurich, Switzerland; ‡ Department of Geography, University of Zurich, Winterthurerstrasse 190, 8057 Zurich, Switzerland; § Laboratory Molecules of Communication and Adaptation of Microorganisms (MCAM). UMR7245, CNRS-Muséum National d’Histoire Naturelle (MNHN), Alliance Sorbonne Université, 57 rue Cuvier, 75005 Paris, France

## Abstract

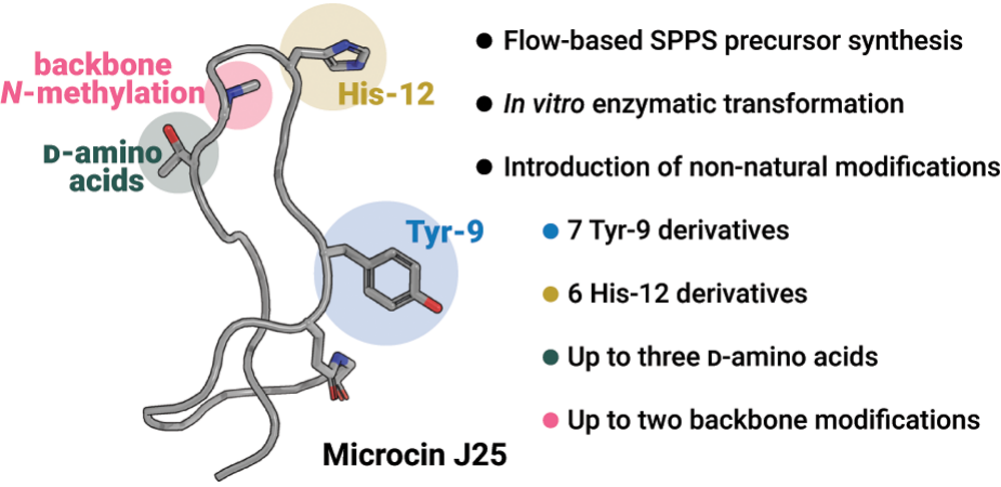

Many peptidic natural products, such as lasso peptides, cyclic peptides, and cyclotides, are conformationally constrained and show biological stability, making them attractive scaffolds for drug development. Although many peptides can be synthesized and modified through chemical methods, knot-like lasso peptides such as microcin J25 (MccJ25) and their analogues remain elusive. As the chemical space of MccJ25 analogues accessible through purely biological methods is also limited, we proposed a hybrid approach: flow-based chemical synthesis of non-natural precursor peptides, followed by *in vitro* transformation with recombinant maturation enzymes, to yield a more diverse array of lasso peptides. Herein, we established the rapid, flow-based synthesis of chemically modified MccJ25 precursor peptides (57 amino acids). Heterologous expression of enzymes McjB and McjC was extensively optimized to improve yields and facilitate the synthesis of multiple analogues of MccJ25, including the incorporation of non-canonical tyrosine and histidine derivatives into the lasso scaffold. Finally, using our chemoenzymatic strategy, we produced a biologically active analogue containing three d-amino acids in the loop region and incorporated backbone *N*-methylations. Our method provides rapid access to chemically modified lasso peptides that could be used to investigate structure–activity relationships, epitope grafting, and the improvement of therapeutic properties.

## Introduction

**Figure 1:**
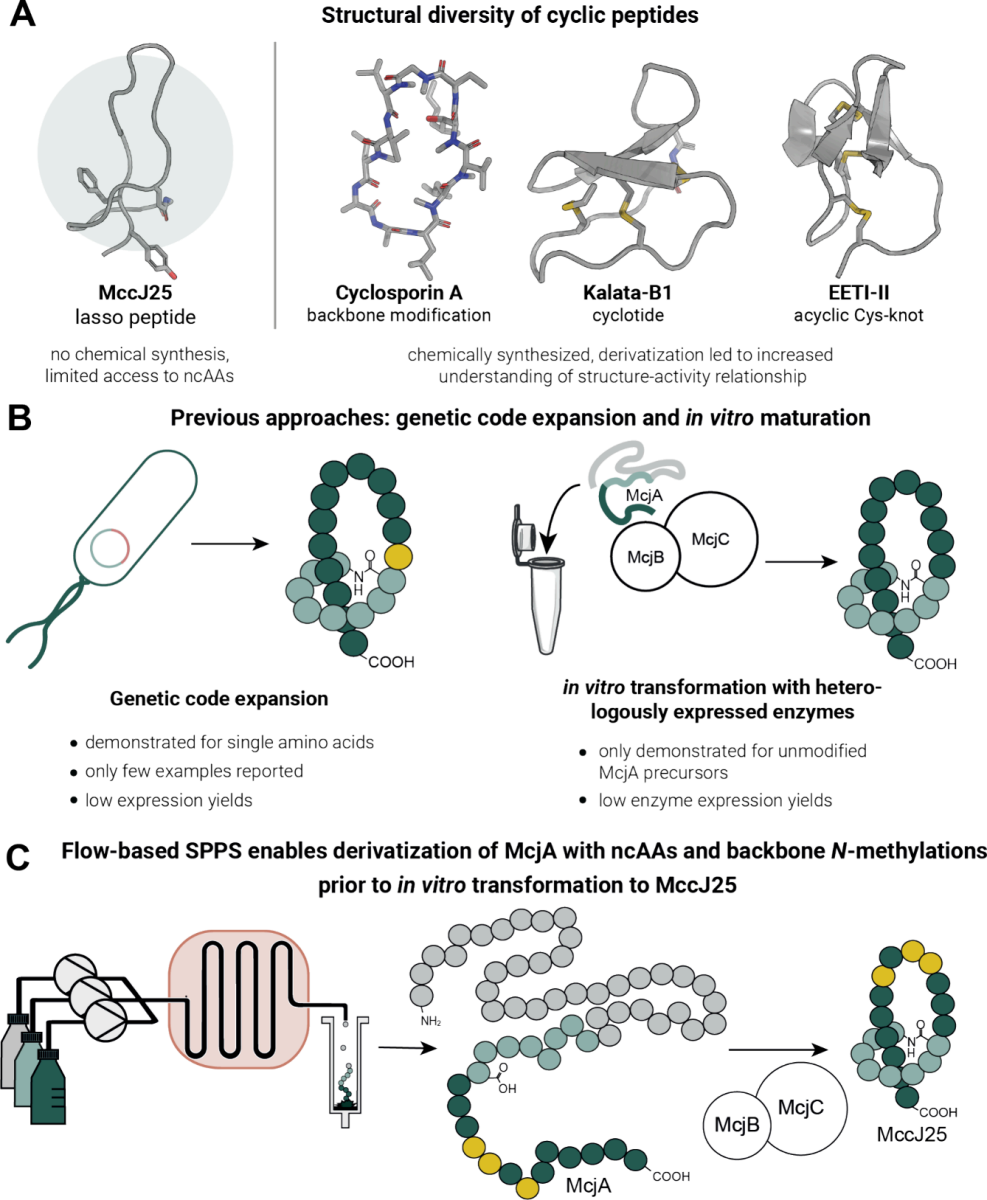
Microcin J25 (bio)synthesis and derivatization. (A) Natural cyclic peptides show great diversity such as knot-like structure, cyclization, and backbone modifications. (B) Heterologous production of MccJ25 in *E. coli* enables the incorporation of a single ncAA via genetic code expansion. *In vitro* transformation of precursor McjA using recombinantly expressed enzymes McjB and McjC yields native MccJ25. (C) This approach: synthesis of chemically modified precursor peptide McjA and subsequent *in vitro* transformation resulted in the formation of MccJ25 bearing ncAAs and backbone modifications.

Lasso peptides are a subclass of ribosomally synthesized and post-translationally modified peptides (RiPPs) classified by their mechanically constrained, knot-like structure.^1^ They are highly attractive starting points for drug discovery efforts, as they combine remarkable enzymatic, thermal, and chemical stability^2−4^ with a variety of biological activities.^5,6^ Rational design to increase the chemical space of the lasso scaffold would require methods for incorporating (multiple) non-canonical amino acids (ncAAs), currently inaccessible via biological approaches. Chemical synthesis would enable the incorporation of ncAAs; however, unlike other cyclic and conformationally constrained peptides (Figure 1A),^7−10^ the bioactive lasso peptide microcin J25 (MccJ25) has resisted numerous synthesis attempts.^11,12^

Nevertheless, biological methods were explored to derivatize MccJ25 with canonical amino acids to alter its activity and study its biogenesis.^13−16^ Biosynthetically, the lasso structure of MccJ25 (21 AA) is achieved through enzymatic maturation of the precursor peptide McjA (57 AA) by two enzymes: McjB removes the leader sequence, and McjC, a lasso cyclase, forms the isopeptide linkage between the N-terminal amine and the Glu-8 side chain.^17^ Previous research has shown that McjB and McjC tolerate up to three simultaneous point mutations in McjA, with a higher acceptance in MccJ25’s C-terminal loop, residues 9–19, with respect to lasso production and activity.^18^ Besides canonical amino acids, the incorporation of ncAAs could significantly modify and improve MccJ25’s properties but remains challenging and underexplored.^19−23^ Link and co-workers used genetic code expansion to incorporate four separate phenylalanine derivatives into MccJ25’s lasso scaffold, but expression yields were significantly lower than for the wild-type peptide (Figure 1B).^24^ Thus far, post-isolation modifications of MccJ25 have included tyrosine nitration,^25^ histidine carbethoxylation, or C-terminal amidation,^26^ which are narrow in scope and not site-selective. A generally applicable chemical total synthesis strategy for lasso peptides, enabling more diverse modifications, remains elusive.^27^

An underexplored approach to incorporate a wider array of ncAAs and backbone modifications into lasso peptides would entail the chemical synthesis of modified precursor peptides, followed by enzymatic transformation *in vitro*. However, this would require overcoming two key hurdles: the synthesis of peptide precursors and enzyme expression. Previously, an approach toward the structurally unrelated thiocillin RiPPs required recombinant expression of the leader, solution-phase synthesis of the core, and native chemical ligation of the two to yield the precursor peptides for enzymatic processing.^28^ While an exclusively chemical precursor synthesis approach would substantially expand the accessible chemical space, the prerequisite long sequences, like McjA (57 AA), can be challenging using standard solid-phase peptide synthesis (SPPS) protocols.^29^ Furthermore, although the required enzymes McjB and McjC were previously obtained through recombinant expression in *Escherichia coli* (Figure 1B), yield and purity were insufficient for a systematic study.^17,30^

Herein, we describe a method to generate chemically modified MccJ25 analogues containing various ncAAs and backbone *N*-methylations, many of which would be difficult or impossible to access by biological methods. First, we employed automated fast-flow peptide synthesis (AFPS), an optimized platform for single-shot synthesis of medium and long sequences, to produce modified precursor peptides.^31,32^ Next, we established a reliable expression protocol for enzymes McjB and McjC. We enzymatically transformed over 20 MccJ25 precursor peptides, containing multiple ncAAs and core peptide substitutions, into lasso peptides to study the impact of these modifications on maturation and bioactivity (Figure 1C). Our hybrid approach uncovers the extensive promiscuity of McjB and McjC to ncAAs and could serve as a blueprint for exploring other lasso peptides and their application in mechanistic studies and drug discovery campaigns.

## Results and Discussion

### The Low-Yielding Expression of Lasso Maturation Enzymes McjB and McjC Was Optimized to Establish a Reproducible Protocol

**Figure 2:**
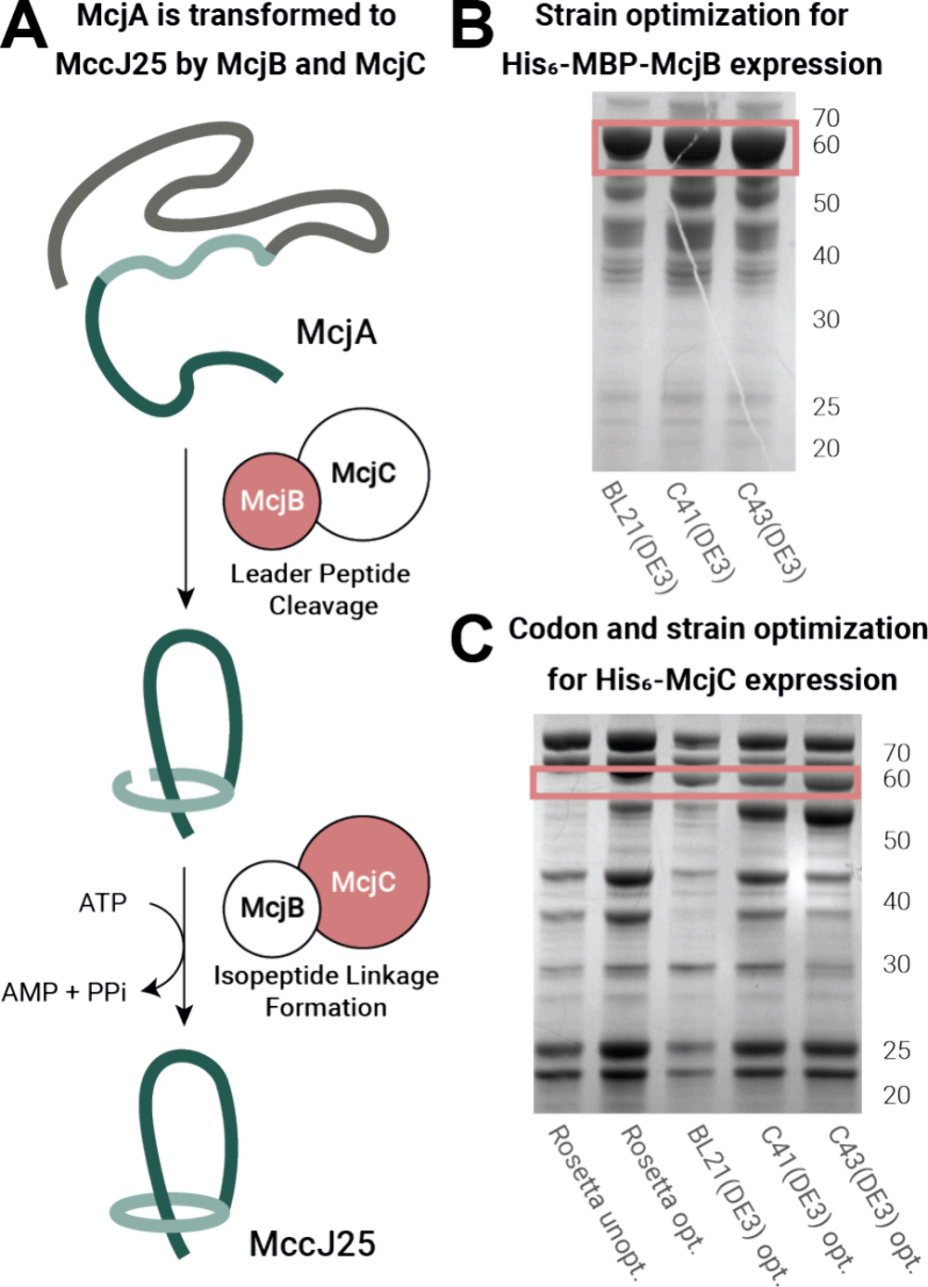
Production optimization of recombinant lasso enzymes McjB and McjC. (A) Mechanism of maturation from precursor peptide McjA to the corresponding lasso peptide MccJ25 via cleavage of the leader peptide by McjB and ring closure by McjC. (B) SDS-PAGE gel analysis of His_6_-MBP-McjB expression, comparing expression outcome of different strains (*E. coli* BL21(DE3), C41(DE3), and C43(DE3)) after single Ni-NTA chromatography purification. *E. coli* C43(DE3) shows the highest protein yield. (C) SDS-PAGE gel analysis of optimized expression procedure of His_6_-McjC in different strains (*E. coli* Rosetta(DE3)pLysS, BL21(DE3), C41(DE3), and C43(DE3)) before and after codon optimization of the gene of interest (unopt. vs opt.). Codon optimization and expression in *E. coli* C43(DE3) yielded the best results.

The biosynthetic production of MccJ25 requires the simultaneous presence of the leader peptidase (McjB) and lasso cyclase (McjC) (Figure 2A).^17^ To date, their molecular mechanism remains cryptic due to difficulties in obtaining enzymes in sufficient quantity and quality.^12^ To further investigate enzyme promiscuity, we set out to develop a more robust expression protocol for McjB (Figure 2B) and McjC (Figure 2C). In our hands, the expression of N-terminal His_6_-tagged McjC in *E. coli* Rosetta(DE3)pLysS only yielded trace amounts, matching previous reports.^12,17^ To improve expression yields, we performed a codon optimization of the McjC sequence before testing the expression in various *E. coli* strains.^33^ Expression in Rosetta(DE3)pLysS cells using the codon-optimized plasmid did not significantly increase enzyme production, but standard BL21(DE3) cells showed a clear improvement of the yields of His_6_-McjC (Figure 2C), as shown by sodium dodecyl sulfate-polyacrylamide gel electrophoresis (SDS-PAGE). To further improve expression conditions, *E. coli* C41(DE3) and C43(DE3) were tested owing to their increased tolerance toward overexpression of toxic recombinant proteins,^34^ and indeed, they showed superior yields of His_6_-McjC. The resulting protein solutions were analyzed by LC-MS, confirming the presence of His_6_-McjC (Figure S67), which was further corroborated by standard trypsin digestion of the corresponding gel band (Figure 2C, highlighted in pink). Overall, C43(DE3) cells were found to be the best expression host for His_6_-McjC, as it gave a higher protein yield.

Since McjB was active even as an MBP-fusion protein with a His_6_-tag (Figure 2B) for enhanced solubility,^17^ we tested its expression in *E. coli* BL21(DE3) following the reported procedure. Although a clear band for His_6_-MBP-McjB was observed in cell lysates by SDS-PAGE, we were inspired by the results for McjC overexpression and investigated expression in C41(DE3) and C43(DE3) again.^34^ Again, these strains proved to be superior expression hosts, and all subsequent recombinant expressions utilized C43(DE3) cells.

Cultivation medium can also have a significant influence on the yield of recombinant protein expression.^35^ Changing the expression medium from lysogeny broth (LB) to terrific broth (TB) medium gave greater amounts of cells and ultimately resulted in higher enzyme yields for both His_6_-MBP-McjB and His_6_-McjC. In summary, the optimized expression conditions featured C43(DE3) cells and TB medium, yielding the highest amounts of both processing enzymes. After a single step of gravity-flow Co-affinity chromatography purification and desalting via PD-10 column (Figure S70), both enzymes, although not pure, were used for the transformation of McjA to lasso peptide MccJ25 with the concentrations being calculated from the mixture.^17,30^

### Flow-based Peptide Synthesis Enables the Rapid Production of Lasso Precursor Peptides and Their Subsequent *In Vitro* Enzymatic Maturation

**Figure 3:**
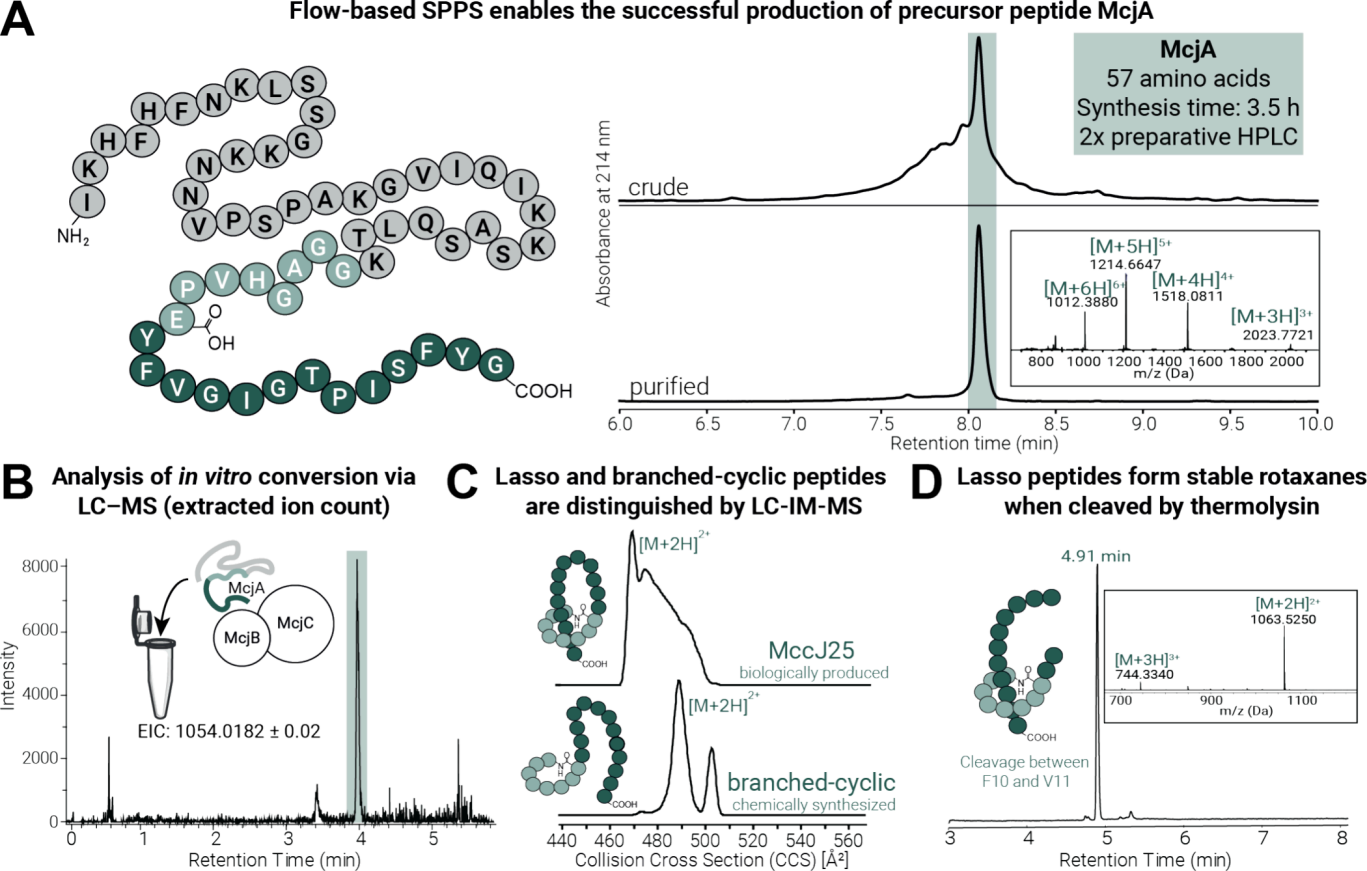
Establishing the chemical synthesis of precursor peptides and the analysis of lasso peptides after successful *in vitro* transformation. (A) Schematic representation of McjA and corresponding LC and MS analysis of synthetic McjA, using AFPS before and after purification by RP-HPLC. (B) LC-MS analysis (extracted ion chromatogram, EIC) of MccJ25 (highlighted in green) following the *in vitro* McjA transformation assay with McjB and McjC. (C) Peptide conformation analysis via LC-IM-MS to distinguish lasso and branched-cyclic peptides. (D) Digestion with thermolysin forms [2]rotaxanes from MccJ25 due to Phe-19 and Tyr-20 residues, confirming the lasso peptide structure.

We next sought to establish the chemical synthesis of native McjA as a blueprint for chemically modified MccJ25 derivatives. The rapid, flow-based synthesis of wild-type McjA[2–58] (Figure 3A) could be accomplished using standard AFPS conditions without further optimization.^31^ The peptide was synthesized on HMPB-linked resin within 3.5 h (crude purity: 43%) and then cleaved and purified twice by reversed-phase high-performance liquid chromatography (RP-HPLC) to obtain McjA bearing the C-terminal carboxylic acid with a purity of 94% and an isolated yield of 8% (4.1 mg, 0.7 μmol) (Figure 3A).

After successfully synthesizing McjA and expressing McjB and McjC, the *in vitro* maturation of synthetically produced MccJ25 precursor was performed.^17,30^ The precursor McjA was mixed in a ratio of 2:1:1 with His_6_-MBP- McjB and His_6_-McjC, supplemented with ATP, MgCl_2_, and TCEP (pH = 8). The transformation was carried out on a 1.5 nmol scale (with respect to McjA) and analyzed by LC-MS (Figure 3B), with partial conversion observed (Figure S76). Lasso and branched-cyclic (unthreaded) peptide products were distinguished with LC-IM-MS measurements by comparing the mobilograms and the smallest collision cross section (CCS) peak (Figure 3C), as previously established for lasso peptides.^11,12,36^ In addition, threadedness could be evaluated by thermolysin digestion combined with MS analysis, as MccJ25 cleavage forms [2]rotaxanes, owing to the steric plugs Phe-19 and Tyr-20 (Figure 3D), while branched-cyclic peptides are cleaved into separate, not interlocked peptides.^37^ For control samples, we used biologically produced MccJ25 and its chemically synthesized branched-cyclic analogue, which was obtained via on-resin cyclization.^38^ Comparison of MccJ25 obtained from *in vitro* maturation with the control samples confirmed the successful transformation of synthetic McjA to the corresponding lasso peptide. We assume all lasso peptides produced by enzymatic maturation are right-handed, like native MccJ25. With an established *in vitro* maturation protocol in hand, we progressed to the chemical synthesis of lasso peptide precursors with non-canonical single point mutations and their enzymatic processing by McjB and McjC.

### Chemical Synthesis Allows for the Rapid Incorporation of Non-canonical Tyrosine and Histidine Derivatives as Single Point Mutations

**Figure 4:**
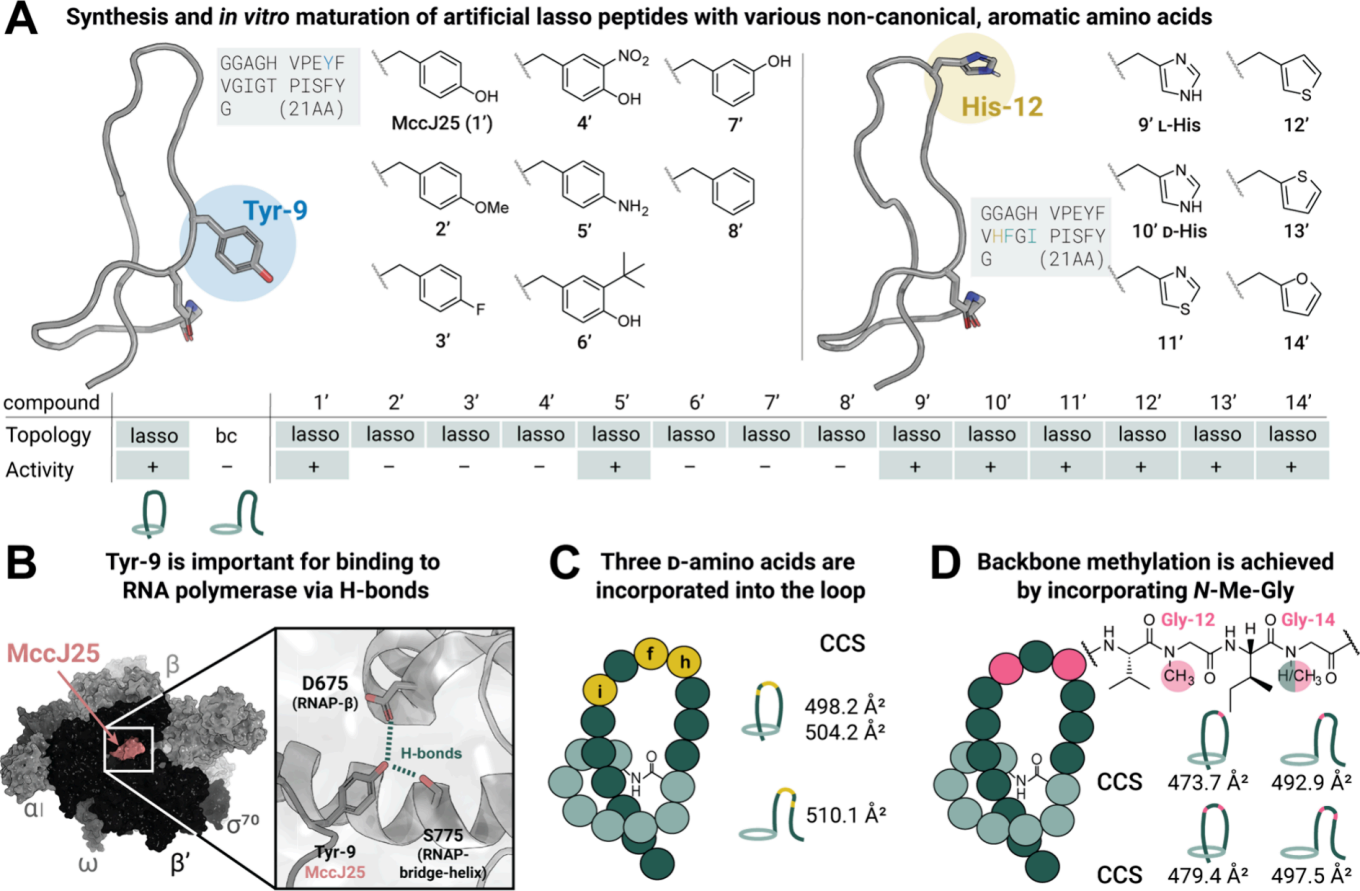
Investigation of MccJ25 derivatives bearing ncAA and backbone modifications for their acceptance by maturation enzymes and the resulting antimicrobial activity. (A) Structure of MccJ25 with Tyr-9 (PDB: 1Q71) and His-12 (mutated using PyMOL from PDB: 1Q71) side chains. The table indicates the transformation of all derivatives to lasso peptides with their corresponding CCS values and activity against *Salmonella enterica* serotype Enteritidis (+/green: active, −: inactive). (B) Binding of MccJ25 into the secondary channel of *E. coli* RNA polymerase sigma70-holoenzyme (PDB: 6N60), highlighting the H-bonds of the Tyr-9 side chain inside the RNAP. (C) Schematic representation of MccJ25 with three substituted d-amino acids (highlighted in yellow) and CCS values of the corresponding lasso peptide and branched-cyclic peptide. (D) *N*-methylation of Gly-12 and additionally Gly-14 residue via incorporation of Fmoc-protected *N*-methyl glycine with CCS values of the lasso peptide and the branched-cyclic peptide.

The incorporation of ncAAs could yield additional insights into enzyme promiscuity and enable structure–activity relationship studies. As a first case study, we investigated the incorporation of ncAA derivatives^a^ of Tyr-9 in wild-type MccJ25 (Figure 4A). Tyr-9 is reportedly essential for antimicrobial activity, as it is involved in a key hydrogen bond to RNA polymerase (RNAP), inhibiting transcription by interfering with NTP binding (Figure 4B).^39^ Furthermore, it affects the membrane respiratory chain, resulting in superoxide production.^40^ Therefore, we investigated derivatives of Tyr-9 that either lack the H-bond donor (-methoxy **2′**, -fluoro **3′**), contain a different donor moiety (aniline **5′**), or are devoid of donor and acceptor attributes (Y9F, **8′**). Notably, there are conflicting reports regarding the bioactivity of MccJ25 Y9F variants.^13,41^ We additionally studied the impact of shifting to a *meta*-hydroxy group (**7′**) on H-bonding in the RNAP-binding pocket. A nitro (**4′**) or *tert*-butyl group (**6′**) was introduced in the *meta* position of Tyr-9 to alter the electronic and steric properties. The syntheses of all Tyr-9 modified analogues of McjA (**2–8**) were performed using AFPS technology, except for the ncAAs, which were incorporated via batch-SPPS using PyAOP and DIPEA (see the Supporting Information (SI)). Aniline derivative Fmoc-Phe(4-NHTrt)-OH was incorporated as a crude mixture after side-chain protection of Fmoc-Phe(4-NH_2_)-OH using trityl chloride (see the SI). After synthesis, peptides were cleaved and purified by RP-HPLC to obtain all precursor peptides (**2–8**) in moderate-to-good isolated yields (2–6%) and high purity (87% to >95%).

Next, we explored His-12 variants of MccJ25, inspired by previous reports of the improved antibacterial activity of MccJ25 after simultaneous installation of His-12, Phe-13, and Ile-15 into the loop region (**9′–14′**).^18^ The previously described His-12 MccJ25 mutant **9′** was further derivatized with ncAAs, including d-His (**10′**) and four other five-membered heterocycles (compounds **11′**–**14′**). All His-12 derivatives of McjA (**9**–**14**) were prepared by AFPS, as described for Tyr-9 derivatives **2**–**8**, but with lower isolated yields due to co-elution of target peptides with deletion side products, and only RP-HPLC fractions with very high purity (>95%) were selected for folding and activity studies.

With McjA peptides bearing either Tyr-9 or His-12 modifications in hand, *in vitro* transformations using His_6_-MBP-McjB and His_6_-McjC were performed and analyzed for conversion by LC-MS. To our delight, conversion of all McjA derivatives was confirmed, demonstrating the capability of McjB and McjC to produce cyclic peptides with various ncAAs in the McjA scaffold. To distinguish branched-cyclic from lasso peptides, the products of the *in vitro* assays were analyzed by LC-IM-MS and compared to standards (Figure 3C), evaluating the mobilogram shape and the CCS of the peak with the smallest CCS value. The CCS value of the [M + 2H]^2+^ charge state of expressed lasso standard MccJ25 (469.4 Å^2^) is noticeably different from that of the branched-cyclic analogue (**bc-1′**)(489.4 Å^2^). The results of the maturation assay of WT-McjA (**1**) and Tyr-9 McjA analogues Phe(4-OMe) **2**, Phe(4-F) **3**, Tyr(3-NO_2_) **4**, Phe(3-OH) **7**, and Phe **8** mimic the mobilogram of the native lasso standard, indicating the successful formation of a lasso structure. Tyr-9 derivative **6′** (Tyr(3-*t*Bu)) showed a CCS value of 481.3 Å^2^, which falls between the lasso and the branched-cyclic standard. To unambiguously assign whether the peptide is threaded, a branched-cyclic version (**bc-6′**) was synthesized, and its CCS value was measured via LC-IM-MS (497.3 Å^2^), demonstrating the formation of a lasso peptide after enzymatic maturation of compound **6**. For aniline derivative **5′**, the similar mobilograms of product and branched-cyclic peptide (**bc-5′**) from LC-IM-MS analysis of the [M + 2H]^2+^ species (Figure S85) precluded definitive assignment. However, the formation of a [2]rotaxane upon thermolysin digestion indirectly confirmed the successful maturation of aniline derivative **5′**. The digestion of the corresponding chemically synthesized branched-cyclic peptide resulted in the formation of separate peptides. For the His-12 derivatives, the presence of the lasso peptide was confirmed via LC-IM-MS analysis. In the case of His-12 derivatives, successful transformation into lasso peptides of all heterocyclic derivatives could be confirmed via LC-IM-MS analysis. Moreover, configurations with both l- and d-His were accepted and transformed, demonstrating the high versatility of this approach.

We next evaluated all lasso peptides and their corresponding branched-cyclic peptides for antimicrobial activity in spot-on-lawn assays against *Salmonella enterica* serotype Enteritidis.^42^ As expected, the tested branched-cyclic peptides bearing l-His or d-His did not show antimicrobial activity, while enzymatically matured His-12 lasso derivatives **9′**–**14′** were indeed active. The derivatives bearing azole side chains (**9′**, **10′**, and **11′**) displayed slightly stronger activity (Figure S77). For Tyr-9 derivatives, activities were only observed for matured WT-McjA (MccJ25, **1′**) and aniline derivative **5′**, indicating the importance of the H-bond donor at this position, while the H-bond acceptor properties of Tyr-9 only play a minor role (Figure S78). Moreover, steric constraints in the tight binding pocket are a limiting factor for Tyr-9 substitutions. To further highlight the importance of the lasso structure, branched-cyclic versions of (**1′**) and (**5′**) were evaluated (Figure S79) and confirmed to be inactive. In conclusion, this study establishes the broad substrate scope of maturation enzymes McjB and McjC for single non-canonical mutations, even at a key residue of MccJ25, enabling the rapid production of non-natural lasso peptides.

### *In Vitro* Maturation of Chemically Synthesized McjA Precursor Peptides Enables the Production of MccJ25 Derivatives Previously Inaccessible

After the successful incorporation of d-His, we considered simultaneously exchanging all three substitutions reported by Link and co-workers (His-12, Phe-13, and Ile-15)^18^ to their corresponding d-amino acids (Figure 4C), which could give access to antimicrobial lasso peptides with improved proteolytic stability. The synthesis of the precursor peptide was performed on the AFPS system, coupling the three d-amino acids with the same synthesis procedure used for the native peptide. The resulting McjA derivative **15** was transformed *in vitro* following our standard protocol, which yielded the lasso peptide **15′** as indicated by the CCS values (compared to the corresponding branched-cyclic analogue (**bc-15′**)). The antimicrobial activity of **15′** tested by a spot-on-lawn assay gave comparable results to the lasso peptides bearing only l-amino acids or one d-histidine, while the branched-cyclic analogue **bc-15′** was inactive. Having access to an active derivative bearing three d-amino acids in the loop portion provides an opportunity for further design of antimicrobial lasso peptides with enhanced stability against proteases in the loop region.

**Figure 5:**
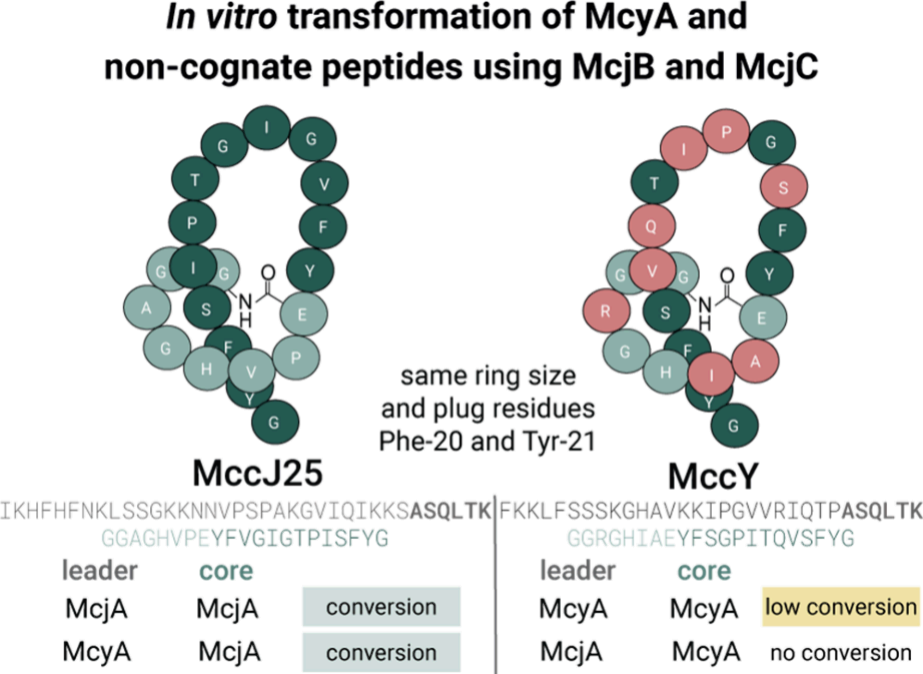
Comparison between MccJ25 and MccY. Representation of lasso peptide sequences and comparison of precursor peptides: McjA for MccJ25 and McyA for MccY. Results demonstrate successful transformations of the precursor peptide McyA and a noncognate peptide containing McyA leader and McjA core. Conversely, transformation was not observed with a noncognate peptide containing McjA leader and McyA core using the McjB/McjC lasso synthetase complex.

To probe the limits of McjB and McjC promiscuity, we attempted to transform even more modified precursor peptides. First, we employed a synthetic d-McjA precursor to establish whether McjB and McjC could recognize it and synthesize d-lasso peptide MccJ25. However, the maturation assay did not yield the desired d-lasso peptide. We observed the same result when only the core peptide sequence was replaced with d-amino acids. Next, we explored if McjB and McjC are capable of transforming precursor peptides of other lasso peptides. To this end, the transformation of chemically produced precursor McyA to microcin Y (MccY),^43^ a lasso peptide sharing key structural features with MccJ25 despite having eight canonical substitutions in the core peptide, was performed (Figure 5). Although LC-MS analysis indicated only low conversion, the enzymes’ ability to produce mature lasso peptide MccY using a different leader peptide is remarkable. Notably, by synthesizing and transforming mixed derivatives between McjA and McyA, we determined that the McyA core with the McjA leader did not show conversion, while the leader peptide of McyA is accepted with the MccJ25 core sequence.

Introducing backbone modifications is a common strategy to further improve peptide stability.^44−46^ However, they are very challenging to incorporate by purely biochemical methods and have never been reported for MccJ25. Therefore, we synthesized McjA derivatives in which loop residue Gly-12 alone or in combination with Gly-14 was *N*-methylated (Figure 4D). Due to reported difficulties in the production of *N*-methylated peptides,^43^ synthesis conditions were slightly adapted for couplings following the *N*-methylated amino acids, giving both peptides in good yield (8% and 5%, respectively). The peptides underwent enzymatic transformation to the cyclized product as discerned by LC-MS. To draw conclusions regarding the three-dimensional structure, branched-cyclic analogues were synthesized in excellent yields (23% and 20%, respectively), and LC-IM-MS measurements allowed for clear differentiation: CCS values after enzymatic transformations were 473.7 Å^2^ for singly and 479.4 Å^2^ for doubly methylated lasso peptides, while the branched-cyclic derivatives (**bc-16′** and **bc-17′**) showed much higher values (492.9 and 497.5 Å^2^). In summary, the unmatched synthetic flexibility of SPPS, paired with the remarkable maturation enzyme versatility, facilitated the effective integration of numerous ncAAs and backbone *N*-methylations into MccJ25. This process yielded several novel lasso peptide derivatives that were previously unattainable.

## Conclusion

In this study, we established the production of lasso peptides containing ncAAs via flow-based chemical synthesis of precursor peptide McjA derivatives, followed by enzymatic transformation, to rapidly screen enzyme promiscuity and antimicrobial activity of target peptides. The production of the enzymes McjB and McjC was optimized to obtain a reproducible and robust protocol with improved yields. We demonstrated that these enzymes are capable of transforming all synthesized non-natural precursors into lasso peptides *in vitro*, enabling studies of their antimicrobial activity. While all His-12 derivatives were active, only the Tyr-9 and its aniline analogue **5′** retained activity, underscoring the importance of an H-bond donor at this position for biological activity. Inspired by a single point mutation with d-His in the lasso scaffold, we incorporated three d-amino acids into the loop region, which could lead to enhanced peptide stability. Remarkably, installing potentially conformation-altering *N*-methylations in the loop region did not disrupt the enzymes’ ability to mature the precursor peptide into the peptide lariat knot.^44−46^ Overall, the incorporation of His-12 and Tyr-9 derivatives as well as several d-amino acids and backbone modifications demonstrate the potential of combining enzymatic maturation with chemical synthesis to obtain biologically active modified lasso peptides unattainable with current biological methods.^47^

Furthermore, investigations of the related lasso peptide MccY gave insights into enzyme promiscuity regarding precursor recognition (Figure 5). McjB/McjC transformed native precursor McyA (albeit with low conversion) but not the mixed species of McjA leader and McyA core. This contrasts with previous implications of the McjA leader peptide in the binding of peptidase McjB and indicates more nuanced interactions between the leader peptide, core peptide, and McjB/McjC lasso synthetase complex.^48,49^ The transformation of McyA aligns with previous results seen with the lasso synthetase complex of fuscanodin. In this case, TfuB1/TfuB2/TfuC enzymes were able to transform precursor peptide TceA but with less efficient conversion compared to the natural precursor TfuA.^50^ Furthermore, a noncognate precursor peptide with TfuA leader and citrulassin A core was not transformed by fuscanodin (called fusilassin in this study) processing enzymes. This matches the results seen with our noncognate McjA leader and McyA core precursor peptide.^51^ However, the inverse precursor peptide with the McjA core and McyA leader was successfully converted into MccJ25. This can be rationalized as the first six amino acids in the leader sequence of McyA and McjA are identical, and McjA variants with minimal leader sequences are still (inefficiently) transformed.^52,53^ Overall, these synthetic hybrid precursor peptides could complement computational tools to further study lasso biogenesis.^54,55^

The main advantage of our *in vitro* approach lies in the rapid production of a series of ncAA-containing McjA precursors for processing by maturation enzymes McjB and McjC. This permits the rapid progression to biological screening, circumventing potentially cumbersome genetic code expansion for each derivative. We also demonstrate the first incorporation of multiple ncAAs (three d-amino acids) and unnatural backbone modifications into MccJ25, a significant advancement for epitope grafting onto the lasso scaffold.^15^ On the backdrop of MccJ25’s antimicrobial activity, this opens new avenues for discovering antibiotics with enhanced activity and stability.

The technology of flow-based chemical synthesis of precursor peptides could be further expanded to other *in vitro* lasso peptide maturation systems^50,51^ or cell-free biosynthesis procedures,^56^ as shown for fuscanodin. With over 80 discovered lasso peptides^12^ and more to be identified by genome mining,^57,58^ there is significant room for future exploration. The combination of reliable chemical peptide synthesis and reproducible enzymatic expression thus adds lasso peptides to the RiPP enzyme toolbox. It could shed light on transformation mechanisms as well as offer possibilities to add novel functionalities, paving the way for RiPP-based therapeutics with improved drug-like properties.^59^

## Data Availability

Data for this paper, including raw LC-MS, UHPLC, LC-IM-MS data are available at Zenodo at https://doi.org/10.5281/zenodo.11198426.

## Supporting Information Available

Additional experimental details, materials and methods, and supplementary data (PDF)

## Abbreviations

AA: amino acid
AFPS: automated fast-flow peptide synthesizer
ATP: adenosine triphosphate
CCS: collision cross section
*E. coli*: *Escherichia coli*
LB: lysogeny broth
LC-MS: liquid chromatography–mass spectrometry
LC-IM-MS: liquid chromatography–ion mobility–mass spectrometry
MccJ25: microcin J25
ncAA: non-canonical amino acid
NTP: nucleotide triphosphate
RiPP: ribosomally synthesized and post-translationally modified peptide
RP-HPLC: reverse-phase high-performance liquid chromatography
RNAP: RNA polymerase
TB: terrific broth
TCEP: tris(2-carboxyethyl)phosphine
WT: wild-type

## Footnotes

a Precursor peptides containing the leader sequence are numbered (e.g., McjA = **1**). Corresponding matured lasso peptides are indicated with the number of the precursor followed by prime (′) (e.g., MccJ25 = **1′**). Branched-cyclic peptides are indicated by the abbreviation “**bc**” followed by the number of the related precursor peptide and prime (e.g., branched-cyclic MccJ25 = **bc-1′**).
