# Supplementary material for: Merging Flow Synthesis and Enzymatic Maturation to Expand the Chemical Space of Lasso Peptides
Source: J Am Chem Soc. 2024 May 17;146(25):17261–9. doi: 10.1021/jacs.4c03898 (PMC11212047; doi:10.1021/jacs.4c03898)
Supplement: Supplementary file 1 — ja4c03898_si_001.pdf [file ja4c03898_si_001.pdf]

Supporting Information

Merging Flow Synthesis and Enzymatic Maturation to Expand the Chemical Space of Lasso Peptides

Authors: Kevin Schiefelbein[1], Jakob Lang[1,2], Matthias Schuster[1], Claire E. Grigglestone[1], Robin Striga[3], Laurent Bigler[1], Meredith C. Schuman[1,2], Oliver Zerbe[1], Yanyan Li[3], Nina Hartrampf[1,*]

Affiliations:

[1] Department of Chemistry, University of Zurich, Winterthurerstrasse 190, 8057 Zurich, Switzerland

[2] Department of Geography, University of Zurich, Winterthurerstrasse 190, 8057 Zurich, Switzerland

[3] Laboratory Molecules of Communication and Adaptation of Microorganisms (MCAM). UMR7245, CNRS-Muséum National d'Histoire Naturelle (MNHN), Alliance Sorbonne Université, 57 rue Cuvier, 75005 Paris, France

* Correspondence to: nina.hartrampf@chem.uzh.ch

Table of Content

1 Materials and general methods ... 4

1.1 Reagents and solvents ... 4

1.2 Automated Fast-Flow Peptide Synthesis (AFPS) ... 5

1.3 Manual coupling of the first amino acid to HMPB resin ... 6

1.4 Manual coupling of non-canonical amino acids ... 6

1.5 Synthesis of branched-cyclic peptides ... 7

1.6 Cleavage of peptidyl-resin and analysis of crude products ... 7

1.7 Liquid Chromatography High-Resolution Electrospray Ionization Mass Spectrometry (LC-MS) ... 7

1.8 Analytical Ultra High-Performance Liquid Chromatography (UHPLC) ... 8

1.9 Semi-Preparative Reverse-Phase High-Performance Liquid Chromatography (RP-HPLC) ... 9

1.10 *E. coli* strains and plasmids ... 9

1.11 Medium and antibiotics ... 9

1.12 SDS-PAGE analysis ... 10

1.13 Enzyme concentration determination ... 10

1.14 LC-MS analysis of enzyme samples ... 10

1.15 In-gel protein digestion of SDS-PAGE gel band and LC-MS/MS analysis ... 10

1.16 Ion-Mobility Mass Spectrometry coupled to LC (LC-IM-MS) ... 11

2 Synthesis and analysis of peptide precursors ... 12

2.1 Synthesis of Tyr-derivatives of McjA ... 12

2.1.1 WT-McjA (**1**) ... 12

2.1.2 McjA with Tyr(O-Me) = Y‘ (**2**) ... 14

2.1.3 McjA with Phe(4-F) = Y‘ (**3**) ... 16

2.1.4 McjA with Tyr(3-NO_2) = Y‘ (**4**) ... 18

2.1.5 McjA with Phe(4-NH_2) = Y‘ (**5**) ... 20

2.1.6 McjA with Tyr(3-tBu) = Y‘ (**6**) ... 23

2.1.7 McjA with Phe(3-OH) = Y‘ (**7**) ... 25

2.1.8 McjA with Phe (**8**) ... 27

2.2 Synthesis of His-derivatives of Link-McjA[5] ... 29

2.2.1 Link-McjA with L-His12 (**9**) ... 29

2.2.2 Link-McjA with D-His12 = h (**10**) ... 31

2.2.3 Link-McjA with 3-(4-Thiazolyl)-L-alanine = H’ (**11**) ... 33

2.2.4 Link-McjA with 3-Thienyl-L-alanine = H’ (**12**) ... 35

2.2.5 Link-McjA with 2-Thienyl-L-alanine = H’ (**13**) ... 37

2.2.6 Link-McjA with 2-Furyl-L-alanine = H’ (**14**) ... 39

2.2.7 Link-McjA with three D-amino acids (**15**) 41
2.3 Synthesis of D-amino acid containing McjA-derivatives 43
2.3.1 Full-D-McjA 43
2.3.2 Leader-L- and core-D-McjA 45
2.4 Synthesis of Microcin Y (MccY) precursor peptides 47
2.4.1 WT-McyA 47
2.4.2 McjA-leader with McyA-core 49
2.4.3 McyA-leader with McjA-core 51
2.5 Synthesis of backbone *N*-methylated-derivatives of McjA 53
2.5.1 McjA with N-methylation at Gly12 (**16**) 53
2.5.2 McjA with N-methylation at Gly12 and G14 (**17**) 55
2.6 Synthesis of branched-cyclic MccJ25 analogs 57
2.6.1 WT-branched-cyclic MccJ25 (**bc-1'**) 57
2.6.2 Branched-cyclic MccJ25 with Phe(4-NH_2) = Y' (**bc-5'**) 59
2.6.3 Branched-cyclic MccJ25 with Tyr(3-tBu) = Y' (**bc-6'**) 61
2.6.4 Branched-cyclic Link-MccJ25 with L-His (**bc-9'**) 63
2.6.5 Branched-cyclic Link-MccJ25 with D-His (**bc-10'**) 65
2.6.6 Branched-cyclic Link-MccJ25 with three D-amino acids (**bc-15'**) 67
2.6.7 Branched-cyclic MccJ25 with N-Me-Gly12 (**bc-16'**) 69
2.6.8 Branched-cyclic MccJ25 with N-Me-Gly12 and N-Me-Gly14 (**bc-17'**) 72
3. Expression of lasso-processing enzymes McjB and McjC 75
3.1 Optimization of His_6-MBP-McjB expression 75
3.1.1 Protein sequence of His_6-MBP-McjB 75
3.1.2 DNA sequence encoding for McjB 75
3.1.3 Purification via Ni-NTA vs Co resin 75
3.1.4 Expression in different growth medium and cell lines 76
3.2 Optimization of His_6-McjC expression 79
3.2.1 Protein sequence of His_6-McjC 79
3.2.2 Codon optimization of pET28-mcjC 79
3.2.3 Expression in different growth medium and cell lines 80
3.3 Expression of His_6-MBP-McjB and His_6-McjC for transformation of McjA derivatives 83
3.3.1 Expression of His_6-MBP-McjB and purification with Co resin 83
3.3.2 Expression of His_6-McjC and purification with Co resin 84
3.3.3 Expression of His_6-MBP-McjB and purification with Ni-NTA resin 86
3.3.4 Expression of His_6-McjC and purification with Ni-NTA resin 87

4. Transformation of precursor peptides and analysis thereof 89
4.1 Direct expression of wild-type MccJ25 (**lasso**) in *E. coli* 89
4.2 *In vitro* transformation of precursor peptides to lasso peptides 91
4.3 Antimicrobial activity assay against Salmonella strain 93
4.3.1 Preparation of M63 agar 93
4.3.2 General procedure of spot-on-lawn assay 93
4.3.3 Preparation of peptide solutions and results of antimicrobial test 93
4.4 Results of Ion-Mobility Mass-Spectrometry (IM-MS) coupled to LC 95
4.5 Thermolysin digestion of MccJ25 and LC-MS analysis thereof 107
4.5.1 Thermolysin protocol for MccJ25 and branched-cyclic peptide standards 107
4.5.2 Analysis of MccJ25 derivatives after maturation 114
5. Literature 116

1 Materials and general methods

1.1 Reagents and solvents

For peptide synthesis:

Fmoc- and side chain-protected L-amino acids (Fmoc-Ala-OH, Fmoc-Arg(Pbf)-OH, Fmoc-Asn(Trt)-OH, Fmoc-Asp(O*t*Bu)-OH, Fmoc-Cys(Trt)-OH, Fmoc-Gln(Trt)-OH, Fmoc-Glu(O*t*Bu)-OH, Fmoc-Gly-OH, Fmoc-His(Trt)-OH, Fmoc-Ile-OH, Fmoc-Leu-OH, Fmoc-Lys(Boc)-OH, Fmoc-Met-OH, Fmoc-Phe-OH, Fmoc-Pro-OH, Fmoc-Ser(*t*Bu)-OH, Fmoc-Thr(*t*Bu)-OH, Fmoc-Trp(Boc)-OH, Fmoc-Tyr(*t*Bu)-OH, Fmoc-Val-OH) were purchased from the Novabiochem line from Sigma-Aldrich Canada Ltd. or Bachem AG; Fmoc- and side chain-protected D-amino acids (Fmoc-Ala-OH, Fmoc-Arg(Pbf)-OH, Fmoc-Asn(Trt)-OH, Fmoc-Asp(O*t*Bu)-OH, Fmoc-Cys(Trt)-OH, Fmoc-Gln(Trt)-OH, Fmoc-Glu(O*t*Bu)-OH, Fmoc-Gly-OH, Fmoc-His(Trt)-OH, Fmoc-Ile-OH, Fmoc-Leu-OH, Fmoc-Lys(Boc)-OH, Fmoc-Met-OH, Fmoc-Phe-OH, Fmoc-Pro-OH, Fmoc-Ser(*t*Bu)-OH, Fmoc-Thr(*t*Bu)-OH, Fmoc-Trp(Boc)-OH, Fmoc-Tyr(*t*Bu)-OH, Fmoc-Val-OH) were purchased from Advanced ChemTech CreoSalus; Fmoc-L-His(Boc)-OH was purchased from Advanced ChemTech CreoSalus; *O*-(7-azabenzotriazol-1-yl)-*N,N,N',N'*-tetramethyluronium hexafluorophosphate (HATU) and (7-azabenzotriazol-1-yloxy)tripyrrolidinophosphonium hexafluorophosphate (PyAOP) were purchased from Advanced ChemTech CreoSalus or Bachem AG; *N*,*N*-diisopropylethylamine (*i*Pr_2NEt, DIPEA, 99.5%) was purchased from Sigma-Aldrich; trifluoroacetic acid (TFA, for HPLC, ≥99.0%), triisopropylsilane (TIPS, 98%) and 3,6-dioxa-1,8-octane-dithiol (DODT, 95%) were purchased from Sigma-Aldrich or Fluorochem. *N,N*-Dimethylformamide (DMF) was purchased from the Supelco line from Sigma-Aldrich Canada Ltd.; AldraAmine trapping packets (volume 1,000-4,000 mL) were purchased from Sigma-Aldrich Canada Ltd. DMF was treated with AldraAmine trapping packets at least 24 hours before experiments. Dichloromethane (DCM, ≥99.8%) was purchased from Fischer Scientific Ltd.; diethyl ether was purchased from Honeywell Riedel-de Haën; acetonitrile (MeCN, for HPLC gradient grade, ≥99.9%) was purchased from Sigma-Aldrich. HMPB ChemMatrix® resin (0.48 mmol/g loading) was purchased from Sigma-Aldrich. NovaPEG HMPB resin (0.62 mmol/g loading) was purchased from the Novabiochem line from Sigma-Aldrich Canada Ltd., and TentaGel XV HMPA resin (0.29 mmol/g loading) was purchased from Rapp Polymere. Fmoc-protected Tyr derivatives *N*-Fmoc-3-hydroxy-L-phenylalanine (95%) and *N*-Fmoc-3-*tert*-butyl-L-tyrosine (95%) were purchased from abcr, *N*-Fmoc-L-tyrosine methyl ether, *N*-Fmoc-4-fluoro-L-phenylalanine, and *N*-Fmoc-3-nitro-L-tyrosine *N*-Fmoc-Glu(*O*-All)-OH were purchased from Fluorochem, *N*-Fmoc-4-amino-L-phenylalanine was purchased from Combi-Blocks. Fmoc-protected His-derivative *N*-Fmoc-3-(4-thiazolyl)-L-alanine (95%) was purchased from Thermo Fisher, *N*-Fmoc-2-furyl- L-alanine was purchased from Fluorochem, *N*-Fmoc-3-(3-thienyl)-L-alanine was purchased from MedChemExpress, *N*-Fmoc-3-(2-thienyl)-L-alanine was purchased from the Novabiochem line from Sigma-Aldrich Canada Ltd, and [(9H-Fluoren-9-ylmethoxycarbonyl)-methyl-amino]-acetic acid (Fmoc-Sar-OH) was purchased from Fluorochem.. Palladium(0) tetrakis ($Pd(PPh_3)_4$) was purchased from Thermo Fisher; phenysilane (PhSiH3) was purchased from Fluorochem.

For enzyme expression:

Tryptone Plus (for biotechnological purposes), Fermtech® Yeast extract (for biotechnology), TCEP (Neutral) were purchased from Millipore® line from Merck KGaA; sodium chloride (NaCl) and glycerol (for molecular biology) were purchased from Fisher BioReagents; Trizma®base (≥99.9%), Kanamycin sulfate from Streptomyces kanamyceticus (BioReagent), chloramphenicol (BioReagent), methanol (for HPLC, gradient grade, ≥99.9%), and cobalt(II) chloride hexahydrate ($CoCl_2·6H_2O$, bioreagent) were purchased from Sigma-Aldrich; potassium dihydrogen-phosphate (KH_2PO_4) (>98%) was purchased from Roth; di-

potassium hydrogenphosphate (K_2HPO_4) (anhydrous, BioChemical), and acetic acid (BioChemical) were purchased from PanReac Applichem; imidazole (ACS 99+%), LDS Sample Buffer (non-reducing, 4x), and nickel(II) chloride hexahydrate ($NiCl_2·6H_2O$) were purchased from Thermo-Scientific; Isopropyl ß-D-1-thiogalactopyranoside (IPTG, dioxane-free) was purchased from Apollo Scientific, RNase A (from bovine pancreas) and DNase I (from bovine pancreas grade II) were purchased from Roche Diagnostics GmbH; 0.1 % Coomassie® Brilliant Blue G 250 was purchased from Merck KGaA; Carbenicillin disodium salt (for biochemistry) was purchased from BioFroxx; formic acid (ULC/MS, 99%) was purchased from Biosolve Chimie; ethanol (A15-A, 100%) was purchased from Reuss-Chemie AG.

HisPur™ Ni-NTA resin and HisPur™ Cobalt resin were purchased from Thermo Fisher Scientific; QIAprep Spin Miniprep Kit was purchased from Qiagen, Amicon® Ultra-4 (30K), Amicon® Ultra-15 (10K), and Millex® Syringe-driven Filter Unit (sterile, 0.22 μm) were purchased from Millipore®-line from Merck KGaA, PD-10 columns (pre-packed with Sephadex™ G-25 M) was purchased from Cytiva.

For lasso transformation and expression:

Trizma® base (≥99.9%, T1503), Adenosine 5'-triphosphate disodium salt hydrate (BioXtra, ≥99.9%, A7699), magnesium sulfate monohydrate ($MgSO_4·H_2O$) (97%), and glucose (≥99.5%) were purchased from Sigma-Aldrich; magnesium chloride hexahydrate ($MgCl_2·6H_2O$) (CELLURE, ≥99.9%, HN03.1) and potassium dihydrogenphosphate (KH_2PO_4) (≥98%) were purchased from Roth; di-potassium hydrogenphosphate (K_2HPO_4) (anhydrous, BioChemical) was purchased from PanReac Applichem; ammonium sulfate ($(NH_4)_2SO_4$) (ultra pure) was purchased from MP Biomedicals; thiamine hydrochloride (Vitamin B1) was purchased from Apollo Scientific; TCEP, Neutral (580561) was purchased from EMD Millipore Corp.; Bacto™ Casamino acids were purchased from gibco.

For antimicrobial test:

Potassium dihydrogenphosphate (KH_2PO_4), di-potassium hydrogenphosphate (K_2HPO_4), ammonium sulfate ($(NH_4)_2SO_4$), magnesium sulfate ($MgSO_4$), thiamine hydrochloride (Vitamin B1), and glucose were purchased from Sigma-Aldrich; Bacto™ Casamino acids and Difco™ agar was purchased from BD Biosciences.

For ion-mobility MS and thermolysin digestion:

Acetonitrile and Methanol were obtained from Biosolve (ULC grade, Valkenswaard, Netherlands) and formic acid from VWR Chemicals (LC–MS grade, Dietikon, Switzerland). Ultrapure water (< 2 ppb TOC) was produced using a Milli-Q Advantage A10 water purification system (Merck, Burlington, MA, USA). Mass and ion mobility calibration was performed using ESI-L low concentration tune mix bought from Agilent (Santa Clara, CA, USA).

Trizma® base (≥99.9%, T1503), urea (powder, BioReagent for molecular biology, U5378), calcium chloride dehydrate ($CaCl_2·2H_2O$) (ReagentPlus®, ≥99.0%, C3881), Thermolysin from Geobacillus stearothermophilus (Type X, P1512) was purchased from Sigma-Aldrich; ammonium hydrogen carbonate (NH_4HCO_3) (LC-MS grade) was purchased from VWR; acetic acid (BioChemica, 100%, A3701) was purchased from PanReac AppliChem.

1.2 Automated Fast-Flow Peptide Synthesis (AFPS)

Peptides were synthesized on an automated-flow system, which was built in the Hartrampf lab (similar to the published AFPS system).[1] Capitalized letters refer to L-amino acids, lowercase letters refer to D-amino acids. Unless otherwise noted, the following settings were used for peptide synthesis: Flow rate = 20 mL/min

for coupling and deprotection steps, temperature = 85-90 °C (5' loop) and 85-90 °C (reactor). The standard synthetic cycle involves a first step of prewashing the resin at 90 °C for 60 s at 40 mL/min. During the coupling step, three HPLC pumps are used: a 50 mL/min pump head pumps the activating agent HATU or PyAOP (0.38 M), a second 50 mL/min pump head pumps the amino acid (0.40 M), and a 5 mL/min pump head pumps DIPEA (neat). The 50 mL/min pump head pumps delivered 0.398679 mL of liquid per pump stroke; the 5 mL/min pump head pumps 3.9239×10^{-2} mL of liquid per pump stroke.

All peptides were prepared by AFPS and standard Fmoc/tBu protected amino acids (0.40 M in DMF) were coupled using HATU (0.38 M in DMF) or PyAOP (0.38 M in DMF) with DIPEA (neat, 3.0 mL/min) at a total flow rate of 20 mL/min. For amino acids D, E, F, G, I, K, L, M, P, S, W, and Y, a total volume of 6.4 mL of the "coupling solution" (i.e. amino acid (0.20 M), HATU or PyAOP (0.19 M), and DIPEA in DMF) was applied for each coupling. For amino acids A, C, H, N, Q, R, S, T, and V, a total of 10.4 mL of "coupling solution" was applied for each coupling. All amino acids except C and H were preheated at 90 °C during the activation step with HATU or PyAOP, whereas C was preheated at 60 °C with PyAOP, and Fmoc-His(Trt)-OH was preactivated with PyAOP at room temperature, Fmoc-His(Boc)-OH was preactivated with PyAOP at 60°C. Removal of the Nα-Fmoc group was achieved using 20% piperidine with 1% formic acid in DMF at a flow rate of 20 mL/min and a total volume of 6.4 mL at 90 °C. Between each coupling and deprotection step, the resin was washed with DMF (32 mL) at 90 °C with a flow rate of 40 mL/min. After completion of the peptide sequence, the resins were manually washed with DMF (3x 5 mL) and DCM (3 × 5 mL) and dried under reduced pressure.

1.3 Manual coupling of the first amino acid to HMPB resin

Syntheses were either performed on HMPB ChemMatrix® resin (0.42 mmol/g or 0.48 mmol/g loading, purchased from Sigma-Aldrich), NovaPEG HMPB resin (0.62 mmol/g loading, purchased from the Novabiochem-line from Sigma-Aldrich Canada Ltd) or Tentagel XV HMPA resin (0.29 mmol/g loading, purchased from Rapp Polymere). The first amino acid (Fmoc-glycine-OH) was coupled in batch in a 6 mL fritted syringe using the following conditions: Fmoc-Gly-OH (10.0 eq.) was dissolved in DMF (3 mL). DIC (5.0 eq.) and DMAP in DMF (0.2 M, 0.1 eq.) were added, and the mixture was added to the resin. Coupling proceeded overnight (12-24 h). The resin was washed with DMF (3x5 mL) and DCM (3x5 mL), and dried under reduced pressure. The first amino acid was deprotected on the AFPS system. The synthesis using AFPS was performed following the general procedure.

1.4 Manual coupling of non-canonical amino acids

For non-canonical amino acid incorporation, the resin was removed from the flow-based peptide synthesizer to perform the coupling in the syringe in batch. If not otherwise stated, the following conditions were applied: Fmoc-protected Tyr-derivatives (5.0 eq.) were dissolved in PyAOP solution (0.38 M in DMF, 5.0 eq.) and the same volume of DMF was added. DIPEA (10.0 eq.) was added, and the solution was shaken for one minute. The mixture was added to the resin and the coupling continued for 2 h. The resin was washed with DMF (3x 6 mL). The Fmoc-protected L-His or D-His (5.0 eq.) was dissolved in PyAOP solution (0.38 M in DMF, 5.0 eq.) and the same volume of DMF was added. DIPEA (10.0 eq.) was added, and the solution was shaken for one minute. The mixture was added to the resin and the coupling continued for 2 h. The resin was washed with DMF (3x 6 mL). Fmoc-protected His-derivatives (3.0 eq.) were dissolved in PyAOP solution (0.38 M in DMF, 3.0 eq.) and the same volume of DMF was added. DIPEA (6.0 eq.) was added, and the solution was shaken for one minute. The mixture was added to the resin and the coupling continued for 2 h. The resin was washed with DMF (3x 6 mL). Fmoc-protected resin was deprotected on the AFPS system and the synthesis using AFPS was continued following the general procedure.

1.5 Synthesis of branched-cyclic peptides

The first amino acid was introduced on HMPB resin (either ChemMatrix® resin (0.48 mmol/g loading) or NovaPEG HMPB resin (0.62 mmol/g loading) or TentaGel XV HMPA resin (0.29 mmol/g loading) (as described in 1.3. The linear peptide was prepared via AFPS. The first amino acid was deprotected on the AFPS system. The synthesis using AFPS was performed following the general procedure in 1.2 except for Glu8, where Fmoc-Glu(OAll)-OH was incorporated via batch coupling following the procedure in 1.4. The sidechain allyl protection was used to enable on-resin manipulation. Afterwards, the linear peptide was further synthesized using AFPS. The incorporation of non-canonical amino acids was performed following the procedure in 1.4. The resin was weighed in, and a part was used to perform the synthesis of the branched-cyclic peptide. To remove the allyl-protecting group selectively, $Pd(PPh_3)_4$ (2 eq. to resin) and $PhSiH_3$ (20 eq. to resin) were dissolved in 2-3 mL DCM and added to the resin. The reaction proceeded for around 165 min. The resin was washed with DCM (3x5 mL), DMF (3x5 mL), and DCM (3x5 mL) before dried under reduced pressure. For cyclization of the *N*-terminus with Glu8 sidechain, PyAOP (0.38 M, 5 eq.) and DIPEA (10 eq.) were added to the resin and reaction was performed twice for 2 h and once for 12.5 h. The resin was washed with DMF (3x5 mL) and DCM (3x5 mL) and dried under reduced pressure.

1.6 Cleavage of peptidyl-resin and analysis of crude products

Cleavage of the peptidyl resin was carried out using TFA/TIPS/H_2O/DODT (94:1:2.5:2.5, *v*/*v*/*v*/*v,* 2-3 mL) for 2 h at room temperature with gentle mixing. The supernatant was then collected by filtration and concentrated under a light stream of N_2, and the peptides were triturated from ice-cold diethyl ether (14-45 mL), and collected as a pellet by centrifugation. The peptide pellet was dried under a light stream of N_2, dissolved in an aqueous solution containing 50% MeCN and 0.1% TFA, and lyophilized. The crude peptide was analyzed by LC-HR-ESI-MS and UHPLC. The pure peptide sample was obtained using semi-preparative RP-HPLC and was analyzed by LC-MS and UHPLC.

1.7 Liquid Chromatography High-Resolution Electrospray Ionization Mass Spectrometry (LC-MS)

Standard measurements for all synthesized peptides

For determination of peptide masses by LC-MS, the filtered peptide solution was diluted in 10–50% acetonitrile (MeCN) in water with 0.1% TFA (60-500 µL) to a final concentration of approximately 0.1 mg/mL. The samples were analyzed on an Acquity UPLC (Waters, Milford, USA), which is connected to an Acquity eλ diode array detector and a Synapt G2 HR-ESI-QTOF-MS (Waters©, Milford, USA). Separation was carried out on an Acquity BEH C8 HPLC column (1.7 µm particle size, 2.0 × 50 mm, Waters©) which was at room temperature, with a sample injection volume of 5 µL. The elution was performed at a flow rate of 0.4 mL/min with solvent A: H_2O + 0.02% formic acid + 0.04% TFA and solvent B: MeCN + 0.04% formic acid + 0.02% TFA following two LC-MS gradients:

(A) for lasso precursor peptides: isocratic at 10% solvent B for 3 min, followed by a linear gradient from 10–70% B within 9 min, then isocratic at 70% B for 1 min.

(B) for branched-cyclic/lasso peptides: isocratic at 3% Solvent B for 3 min, followed by a linear gradient of 3–95% Solvent B over 9 min, followed by isocratic at 95% Solvent B for 1 min.

Ion source parameters for ESI were: positive ionization mode, capillary voltage 3.0 kV, sampling cone 40 V, extraction cone 4 V, N_2 cone gas 4 L/h, N_2 desolvation gas 800 L/min and source temperature 120 °C. Parameters for the mass analyzer in resolution mode were: mass range 150–3000 *m*/*z* with a scan rate of 1

Hz; mass calibration to <2 ppm within 50–2500 *m*/*z* with a 5mM aq. soln. of HCO_2Na, lock masses: *m*/*z* 195.0882 (caffeine, 0.7 ng/mL) and 556.2771 (Leu-enkephalin, 2 ng/mL). All mass spectra show deconvoluted masses from the raw *m*/*z* values, calculated using Mestrelab Research S.L.© MestReNova v. 14.1 Mnova MS Suite. Purity based on LC-HR-ESI-MS was calculated by calculating the Area Under the Curve (AUC) of the desired product peak as a percentage of the AUC of all peaks (within 2–9 min) of the total absorbance chromatogram (λ = 214 nm). Monoisotopic and average masses for uncharged molecules were calculated using ChemDraw Version 18.2.

Measurements for peptides using lower fragmentation voltage (Agilent LC-MS)

For determination of peptide masses by LC-QTOF (for *N*-methylated branched-cyclic peptides), the filtered peptide solution was diluted in 10–50% acetonitrile (MeCN) in water with 0.1% TFA (500 µL) to a final concentration of approximately 0.1 mM. The samples were analyzed on an Agilent 1290 Infinity II Series UHPLC, which is connected to an Agilent 1260 Infinity II Series VWD, and an Agilent 6546 LC/Q-TOF. Separation was carried out on an Agilent Poroshell 300SB-C8 HPLC column (5 µm particle size, 2.1 × 75 mm) which was at 50 °C, with a sample injection volume of 5 µL. The elution was performed at a flow rate of 0.8 mL/min with solvent A: H_2O + 0.1% formic acid and solvent B: MeCN + 0.1 formic acid with the following LC-MS gradient:

> gradient: isocratic at 5% Solvent B for 1.5 min, followed by a linear gradient of 5–95% Solvent B over 5 min, followed by isocratic at 95% Solvent B for 1 min.

Ion source parameters for ESI were: positive ionization mode, capillary voltage 3.5 kV, nozzle voltage 1 kV, gas temperature 320 °C, N2 drying gas flow 8 L/min, nebulizer pressure 35 psi, sheath gas temperature 350 °C, N2 sheath gas flow 11 L/min, fragmentor voltage 100 V, and skimmer voltage 65 V. Parameters for the mass analyzer in MS (Seg) mode were: mass range 10–3200 m/z with an acquisition rate of 1 spectra/sec and time of 1000 ms/spectra; Mass calibration took place using the Agilent low-concentration tune mix. All mass spectra show deconvoluted masses from the raw m/z values, calculated using Mestrelab Research S.L.© MestReNova v. 14.1 Mnova MS Suite. Purity based on LC-QTOF was calculated by calculating the Area Under the Curve (AUC) of the desired product peak as a percentage of the AUC of all peaks (within 2–9 min) of the total absorbance chromatogram (λ = 214 nm). Monoisotopic and average masses for uncharged molecules were calculated using ChemDraw Version 18.2.

To analyze the lasso assay for residual McjA (**Fig S76**), the same method was applied. The total ion current (TIC) was recorded after 1.0 minutes due to the high salt concentration in the samples.

1.8 Analytical Ultra High-Performance Liquid Chromatography (UHPLC)

For determination of purity by UHPLC, the filtered peptide solution was diluted in 10–50% acetonitrile (MeCN) in water with 0.1% TFA (500 µL) to a final concentration of approximately 1.0 mg/mL. The samples were analyzed on Agilent 1290 Infinity II Series using Agilent OpenLab CDS and ChemStation software.

For standard analysis of all peptide samples, analytical UHPLC spectra were recorded on an analytical Agilent Zorbax 300SB-C18 column Narrow-Bore Rapid Resolution HD column (2.1 mm × 150 mm, 5 µm particle size) or on an analytical Agilent Poroshell 300SB-C8 column (2.1 mm × 75 mm, 5 µm particle size) at a flow rate of 0.8 mL/min with UV detection at 214 nm. For both, a binary solvent system was used, wherein solvent A was 5% MeCN in 95% water with 0.1% TFA, and solvent B was 95% MeCN containing 5% water and 0.1% TFA. After an isocratic period at 0% solvent A for 3 min, a linear gradient of 0–100% solvent B (corresponding to 5–95% MeCN) over 20 min was used (ca. 4.5% MeCN/min). The total method time was 23.1 min. Then, the column was re-equilibrated using a post-run method at 0% Solvent B for 2 min. Purities

of the crude and purified peptides were determined by ChemStation integration of all UHPLC signals at 214 nm in the area of 3-18 min.

1.9 Semi-Preparative Reverse-Phase High-Performance Liquid Chromatography (RP-HPLC)

Semi-preparative RP-HPLC was performed on a Shimadzu prominence HPLC system (Shimadzu Corp., Japan) with a CBM-40 system controller module, an FRC-10A fraction collector, two LC-20AR pumps, and an SPD-40 UV/VIS detector, using an Agilent Zorbax 300SB-C8 Semi-Preparative column (9.4 × 250 mm, 5 μm particle size) at a flow rate of 4 mL/min. A binary solvent system was used, wherein solvent A was H_2O containing 0.1% TFA, and Solvent B was MeCN containing 0.1% TFA. For longer peptides: crude peptides were purified with a flow-rate of 4 mL/min at 30 °C using a gradient of 5%B for 5 min, then 5%B to 25%B over 10 min (ca. 2%B/min) then 25%B to 40%B over 45 min (ca. 0.33%B/min) before flushing the column with 95%B. For shorter peptides (lasso peptide and branched-cyclic peptide): crude peptides were purified with a flow-rate of 4 mL/min at 30 °C using a gradient of 5%B for 5 min, then 5%B to 25%B over 5 min (ca. 4%B/min) then 25%B to 60%B over 70 min (ca. 0.5%B/min) before flushing the column with 95%B. Fractions (~1 mL) were collected automatically using a Shimadzu FRC-10A Fraction Collector. Fractions were analyzed for purity by LC-HR-ESI-MS. All fractions identified with correct m/z and high purity were combined and lyophilized to afford the product as a white amorphous solid.

1.10 *E. coli* strains and plasmids

E. coli strains used were DH5α for plasmid propagation and BL21(DE3), C41(DE3), C43(DE3)[2] or Rosetta(DE3)pLysS for expression. The plasmids pMAL-c5ePre-mcjB (His_6-MBP-McjB) and pET28-mcj$C_{initial}$ (His_6-McjC) were used as previously reported.[3] The plasmid pET28-mcj$C_{optimized}$ was constructed by inserting the codon-optimized mcjC gene in the pET-28a plasmid. The codon optimization is further discussed in section 2.3.2.

1.11 Medium and antibiotics

LB medium (Miller) was prepared by dissolving tryptone (10 g/L), yeast extract (5 g/L), and NaCl (10 g/L) in water. The solution was autoclaved at 121 °C for 20 min.

For 1 L of TB medium, tryptone (12 g), yeast extract (24 g), and glycerol (4 mL) were dissolved in 900 mL water. The solution was autoclaved at 121 °C for 20 min. Afterwards, 100 mL potassium phosphate buffer (0.17 M KH_2PO_4 and 0.72 M K_2HPO_4, sterile filtrated with 0.22 μm filter unit) was added.

The medium was supplemented with around 50 μg/mL carbenicillin for pMAL-c5ePre-mcjB and with around 50 μg/mL kanamycin for pET28-mcjC (before and after codon optimization). Furthermore, chloramphenicol (34 μg/mL) was added for expression in Rosetta(DE3)pLysS cells.

For 1 L of M63 medium, KH_2PO_4 (3 g), K_2HPO_4 (7 g), $(NH_4)_2SO_4$ (2 g) and Casamino acids (1 g) were dissolved in 1 L water and autoclaved. Afterwards, filtered solutions (on a 0.2 μm filter) of MgSO4 (20% m/v, 1 mL), vitamine B1 (5 g/L, 200 μL), and glucose (20% m/v, 10 mL) were added. The medium was supplemented with chloramphenicol (34 μg/mL) for MccJ25 expression.

1.12 SDS-PAGE analysis

SDS-PAGE analysis was performed with ExpressPlus™ PAGE Gels, 10x8 (8-16%, 15 wells of 40 μL) or SurePAGE™, Bis-Tris, 10x8 (8-16%, 15 wells of 40 μL) from GenScript using MOPS buffer. Tris-MOPS-SDS-Running Buffer was purchased from GenScript (M00138). To protein samples (10 μL), DTT (200 mM, 1 μL) and LDS Sample Buffer (non-reducing 4x, 2 μL) were added before analysis. To determine protein mass, PageRuler™ Prestained Protein Ladder and PageRuler™ Unstained Protein Ladder (Thermo Scientific) were used as standards. Gels were stained using 0.1 % Coomassie® Brilliant Blue G 250 in water/ethanol/acetic acid (5:4:1).

1.13 Enzyme concentration determination

Pierce™ BCA Protein Assay Kit (Thermo Scientific, Ref. 23225) was performed in 96-well plates (Thermo Fisher Scientific, 260836). The absorbance was analyzed using a Tecan Safire2 Multi-Detection Plate Reader. For analysis, a second-order polynomial curve was fitted to the standards.

1.14 LC-MS analysis of enzyme samples

Samples were diluted with 1% TFA, passed through the Mobicol filter, and transferred to an autosampler vial for LC/MS. Samples were injected into an ACQUITY UPLC@BioResolve-RP-mAb 2.7 μm 2.1 mmx150 mm, 450 Å (Waters, USA) column. For desalting resp. separation on an Acquity UPLC station, a gradient buffer A (0.1% DFA in water)/ buffer B (0.1% DFA in AN/75% 2-PrOH) at a flow rate 200 ul/min at 60°C over 30 min was applied. The analysis was performed on a Synapt G2-Si mass spectrometer directly coupled to the UPLC station.

Mass spectra were acquired in the positive-ion mode by scanning the m/z range from 400 to 5000 Da with a scan duration of 1 s and an interscan delay of 0.1s. The spray voltage was set to 3 kV, the cone voltage to 50 V, and the source temperature to 100°C. The data were recorded with the MassLynx 4.2 Software (both Waters, UK). The recorded m/z data of single peaks or their slices were deconvoluted into mass spectra by applying the maximum entropy algorithm MaxEnt1 (MaxLynx) with a resolution of the output mass 0.5 Da/channel and Uniform Gaussian Damage Model at the half height of 0.5 Da.

1.15 In-gel protein digestion of SDS-PAGE gel band and LC-MS/MS analysis

Gel bands were cut into small pieces and washed with 100 mM NH_4HCO_3/50% acetonitrile (2x) and acetonitrile (1x). All supernatants were discarded. For the digestion, the gel pieces were covered with a buffered trypsin solution at pH 8 (10 mM Tris/2 mM $CaCl_2$). Samples were enzymatically digested. Supernatants were collected and the remaining peptides were extracted from the gel bands with 0.1% TFA/50% acetonitrile. Both supernatants were combined and dried.

The digested samples were dissolved in aqueous 3% Acetonitrile with 0.1% formic acid, and the peptide concentration was estimated with the Lunatic UV/Vis absorbance spectrometer (Unchained Lab). Peptides were separated on an M-class UPLC and analyzed on an Orbitrap mass spectrometer (Thermo).

The acquired MS data were processed using the Maxquant search engine (V 2.0.1.0, PMID: 19029910). The spectra were searched against the provided customer sequences merged with the following protein background database: *E. coli* K12(83333). The following variable modifications were set: acetylation of the *N*-terminus, methionine oxidation, and deamidation of asparagine and glutamine.

1.16 Ion-Mobility Mass Spectrometry coupled to LC (LC-IM-MS)

Liquid chromatography was performed on a Vanquish Horizon UHPLC System by Thermo Fisher (Waltham, MA, USA) built from a Vanquish binary pump H, a Vanquish split sampler HT and a temperature-controlled Vanquish column compartment. Chromatographic separation was achieved on an ACQUITY UPLC BEH C8 (130 Å, 1.7 µm, 2.1 × 100 mm, Waters, Milford, MA, USA)[A] or an ACQUITY PREMIER HSS T3 (100 Å, 1.8 µm, 2.1 × 100 mm, Waters, Milford, MA, USA) Column at 30 °C. eluent A consisted of H_2O + 0.1% HCOOH and eluent B of MeCN + 0.1% HCOOH. The solvent flow was kept at 0.4 mL/min with the following gradient: (I) 20% B isocratic from 0.0 to 0.5 min; (II) linear increase to 50% B until 4.5 min; (III) linear increase to 100% until 5 min; (IV) holding 100% B until 7 min (V) instant switch back to the starting conditions of 20% B and equilibration for 2 min until the next run.

Ion mobility and mass spectra were recorded on a timsTOF Pro hybrid quadrupole-time-of-flight (QTOF) mass spectrometer employing trapped ion mobility spectrometry (TIMS) produced by Bruker (Bremen, Germany). Ionisation was performed in positive ESI mode using the following source parameters: End plate offset of 500 V, capillary voltage of 4500 V, nebulizer pressure of 3.0 bar (N2), heated dry gas flow of 8.0 L/min with a temperature of 220 °C. Mass and CSS calibration took place using the Agilent low-concentration tune mix. TIMS parameters are listed in the following according to the recommended guidelines for reporting IM-MS measurements[4]: Ion mobility data was acquired with a ramp time of 200 ms in the inverse reduced mobility range of 0.75 – 1.50 1/K0 using ion charge control (ICC) (target count 5 million). The radiofrequency of the TIMS ion funnels was set at 250 Vpp with the Δ6 voltage set to 50, 100, and 150 V for three measurements of each sample. Tunnel-in pressure and tunnel-out pressure of the TIMS cartridge were 2.6 mbar and 0.8 mbar, respectively. The drift gas was N_2 of at least 4.5 purity that was additionally purified by an HC Big Supelpure HC Hydrocarbon Trap from Sigma-Aldrich (Buchs, SG, Switzerland). otofControl 6.0 was employed for data acquisition. DataAnalysis 5.3 was used for the evaluation of the measured results. A mass deviation of up to 5 ppm was tolerated.

[A] This column was used for the lasso and WT-branched-cyclic peptide standard and all Tyr-derivatives (peptides 1-8). For these measurements, the peptide solutions after the maturation assay were diluted 1:10.
The measurements were repeated with undiluted samples on the other column and showed the same results regarding LC-TIMS-MS mobilograms.

2 Synthesis and analysis of peptide precursors

2.1 Synthesis of Tyr-derivatives of McjA

2.1.1 WT-McjA (**1**)

Sequence: `H2N-IKHFHFNKLS SGKKNNVPSP AKGVIQIKKS ASQLTKGGAG HVPEYFVGIG TPISFYG-OH`

The peptide was prepared via Automated Fast-Flow Peptide Synthesis (AFPS) using HMPB ChemMatrix® resin (loading = 0.42 mmol/g, 83 mg, 35 μmol). The first amino acid was introduced as described in 1.3. The first amino acid was deprotected on the AFPS system. The synthesis using AFPS was performed following the general procedure in 1. 2. The total synthesis time was approximately 3.5 h

Cleavage of the peptidyl-resin (25% of total resin) afforded the crude peptide (43% purity (C8) by UHPLC). The peptide was purified using semi-preparative HPLC as specified in the general procedure 1. 9. Fractions were analyzed by LC-HR-ESI-MS, combined, and lyophilized to obtain 4.1 mg (8% yield; 94% purity (C8) and >95% purity (C18) by UHPLC) of the desired peptide.

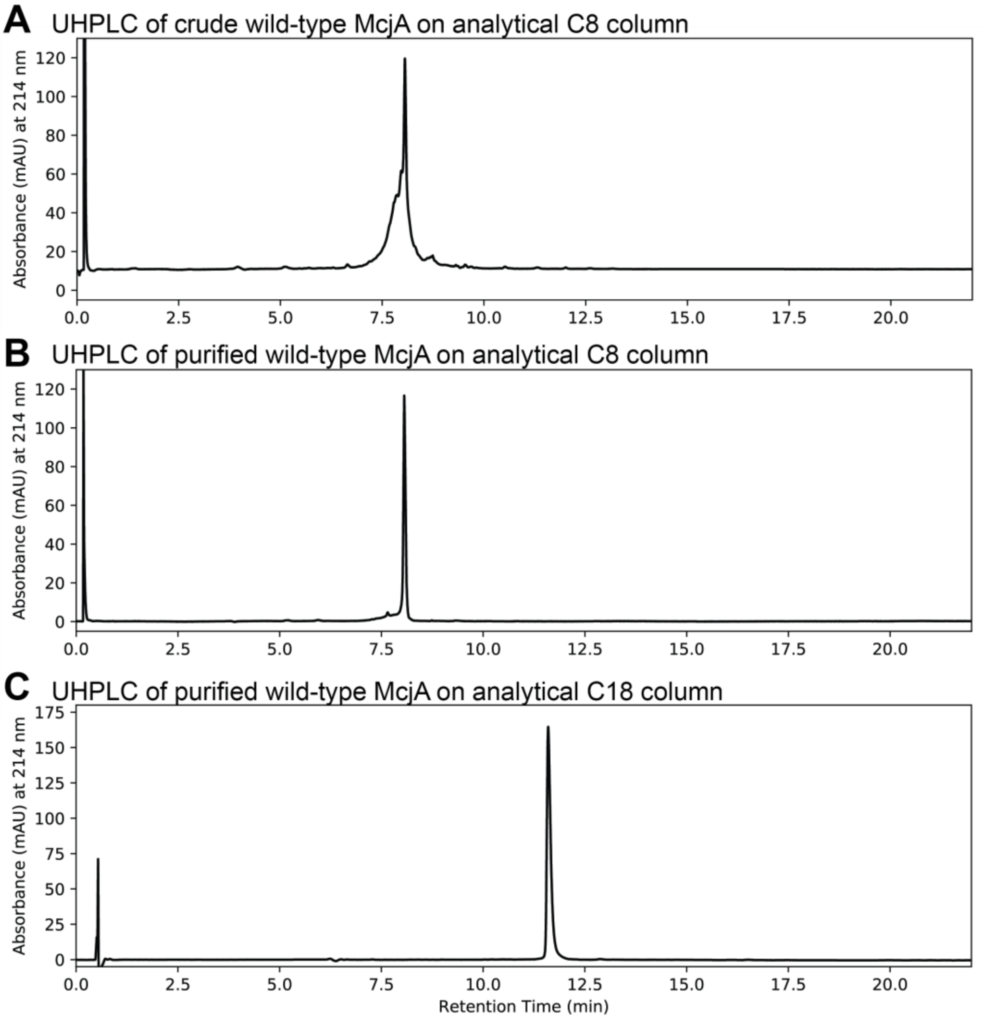

Fig. S1: **UHPLC profile of wild-type McjA:** (A) crude sample on analytical C8 column (43% purity); (B) purified sample on analytical C8 column (94% purity, R_t = 8.06 min); (C) purified sample on analytical C18 column (>95% purity, R_t = 11.60 min).

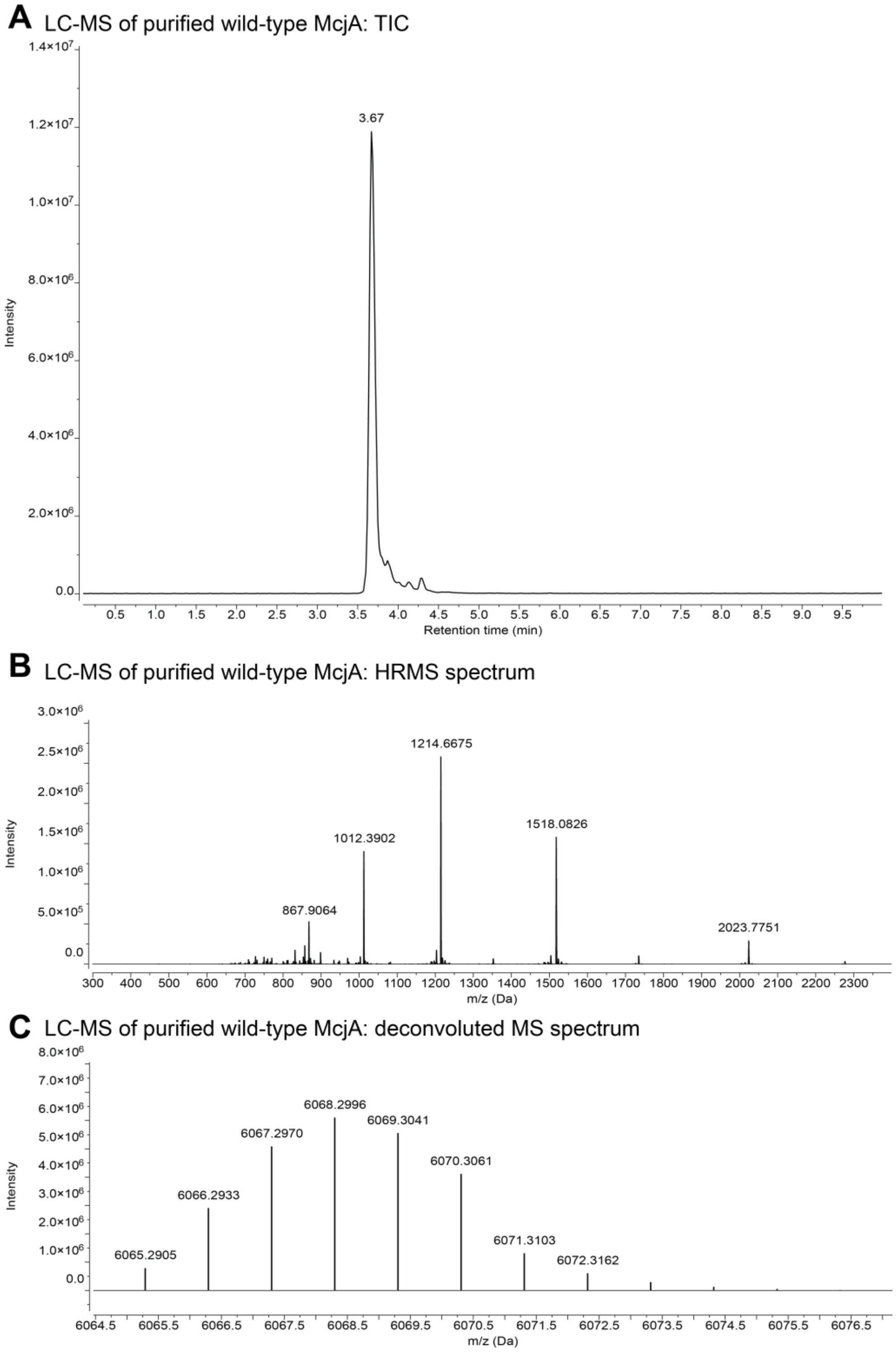

Fig. S2: **LC-MS analysis of wild-type McjA:** (A) Total ion chromatogram (TIC). (B) MS spectrum. (C) deconvoluted MS spectrum: Monoisotopic mass (ESI+): calc. [$C_{280}H_{438}N_{76}O_{75}$]: 6065.2796, found: 6065.2905; Average mass calc. [$C_{280}H_{438}N_{76}O_{75}$]: 6069.0410.

2.1.2 McjA with Tyr(O-Me) = Y‘ (**2**)

Sequence: H_2N-IKHFHFNKLS SGKKNNVPSP AKGVIQIKKS ASQLTKGGAG HVPEY’FVGIG TPISFYG-OH

The peptide was prepared via Automated Fast-Flow Peptide Synthesis (AFPS) using HMPB ChemMatrix® resin (loading = 0.42 mmol/g, 82 mg, 34 µmol). The first amino acid was introduced as described in 1.3. The first amino acid was deprotected on the AFPS system. The synthesis using AFPS was performed following the general procedure in 1.2. until the point of Tyr9, where the non-canonical amino acid Fmoc-Tyr(*O*-Me)-OH was incorporated following the general procedure 1.4. The amino acid was deprotected on the AFPS system and the synthesis using AFPS was continued. The total synthesis time was approximately 5.5 h.

Cleavage of the peptidyl-resin (29% of total resin) afforded the crude peptide (35% purity (C8) by UHPLC). The peptide was purified using semi-preparative HPLC as specified in the general procedure 1.9. Fractions were analyzed by LC-HR-ESI-MS, combined, and lyophilized to obtain 2.1 mg (3% yield; >95% purity (C8) and >95% purity (C18) by UHPLC) of the desired peptide.

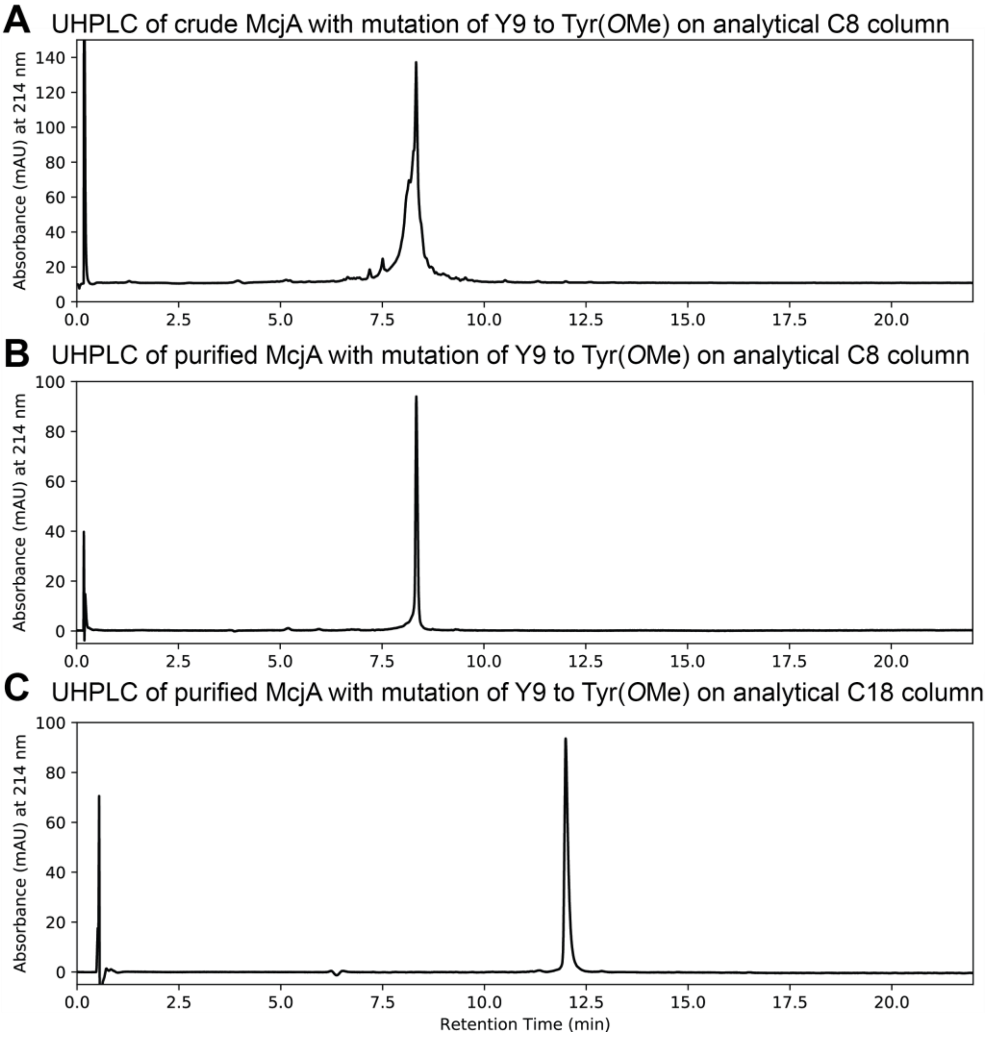

Fig. S3: **UHPLC profile of McjA with mutation of Y9 to Tyr(*O*Me):** (A) crude sample on analytical C8 column (35% purity); (B) purified sample on analytical C8 column (>95% purity, R_t = 8.34 min); (C) purified sample on analytical C18 column (>95% purity, R_t = 11.99 min).

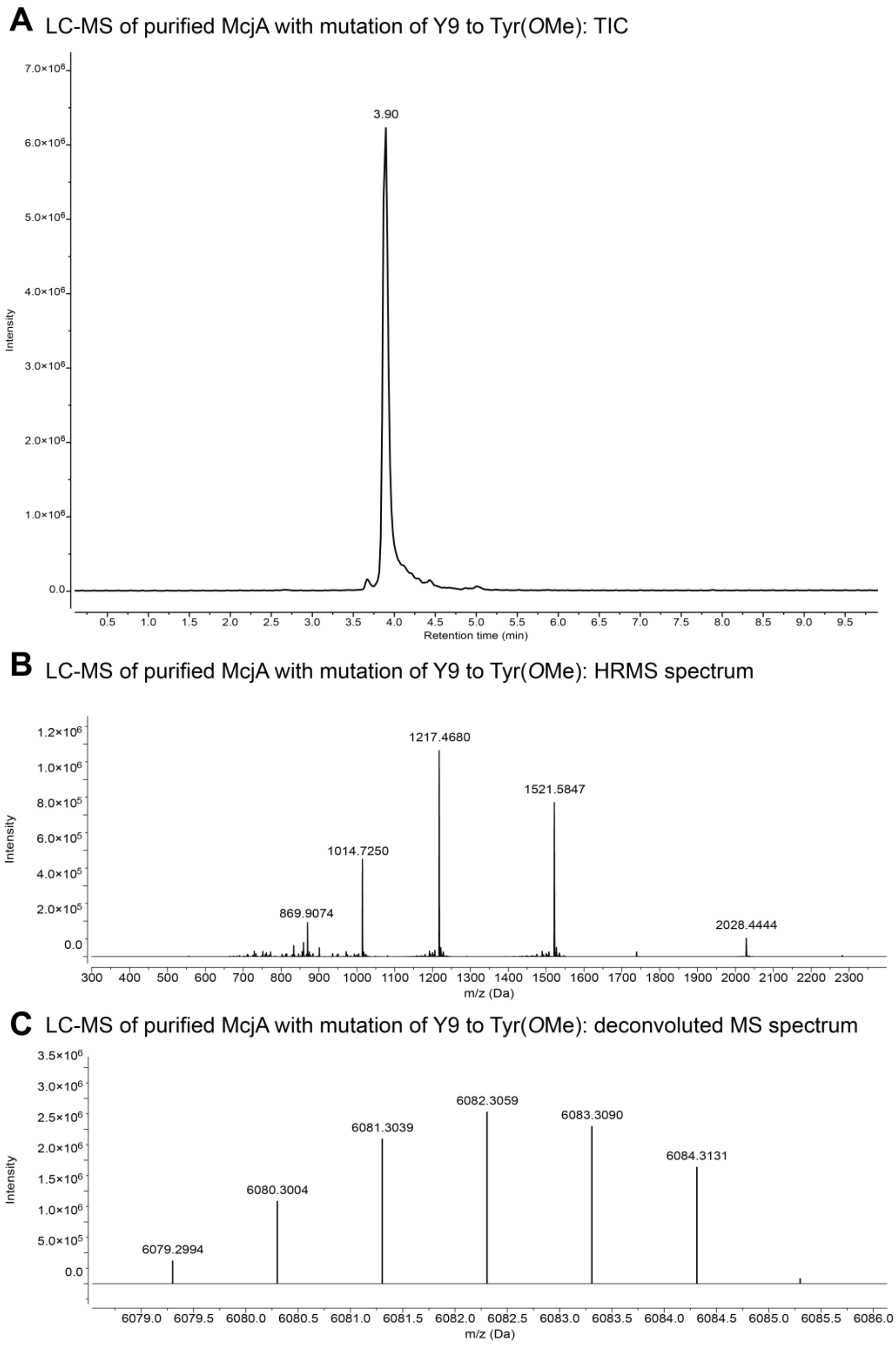

Fig. S4: LC-MS analysis of McjA with mutation of Y9 to Tyr(*O*Me): (A) Total ion chromatogram (TIC). (B) MS spectrum. (C) deconvoluted MS spectrum: Monoisotopic mass (ESI+): calc. [$C_{281}H_{440}N_{76}O_{75}$]: 6079.2952, found: 6079.2994; Average mass calc. [$C_{281}H_{440}N_{76}O_{75}$]: 6083.0680.

2.1.3 McjA with Phe(4-F) = Y' (**3**)

Sequence: H_2N-IKHFHFNKLS SGKKNNVPSP AKGVIQIKKS ASQLTKGGAG HVPEY'FVGIG TPISFYG-OH

The peptide was prepared via Automated Fast-Flow Peptide Synthesis (AFPS) using HMPB ChemMatrix® resin (loading = 0.42 mmol/g, 85 mg, 36 µmol). The first amino acid was introduced as described in 1.3. The first amino acid was deprotected on the AFPS system. The synthesis using AFPS was performed following the general procedure in 1.2. until the point of Tyr9, where the non-canonical amino acid Fmoc-Phe(4-F)-OH was incorporated following the general procedure 1.4. The amino acid was deprotected on the AFPS system and the synthesis using AFPS was continued. The total synthesis time was approximately 5.5 h.

Cleavage of the peptidyl-resin (28% of total resin) afforded the crude peptide (36% purity (C8) by UHPLC). The peptide was purified using semi-preparative HPLC as specified in the general procedure 1.9. Fractions were analyzed by LC-HR-ESI-MS, combined, and lyophilized to obtain 3.6 mg (6% yield; >95% purity (C8) and >95% purity (C18) by UHPLC) of the desired peptide.

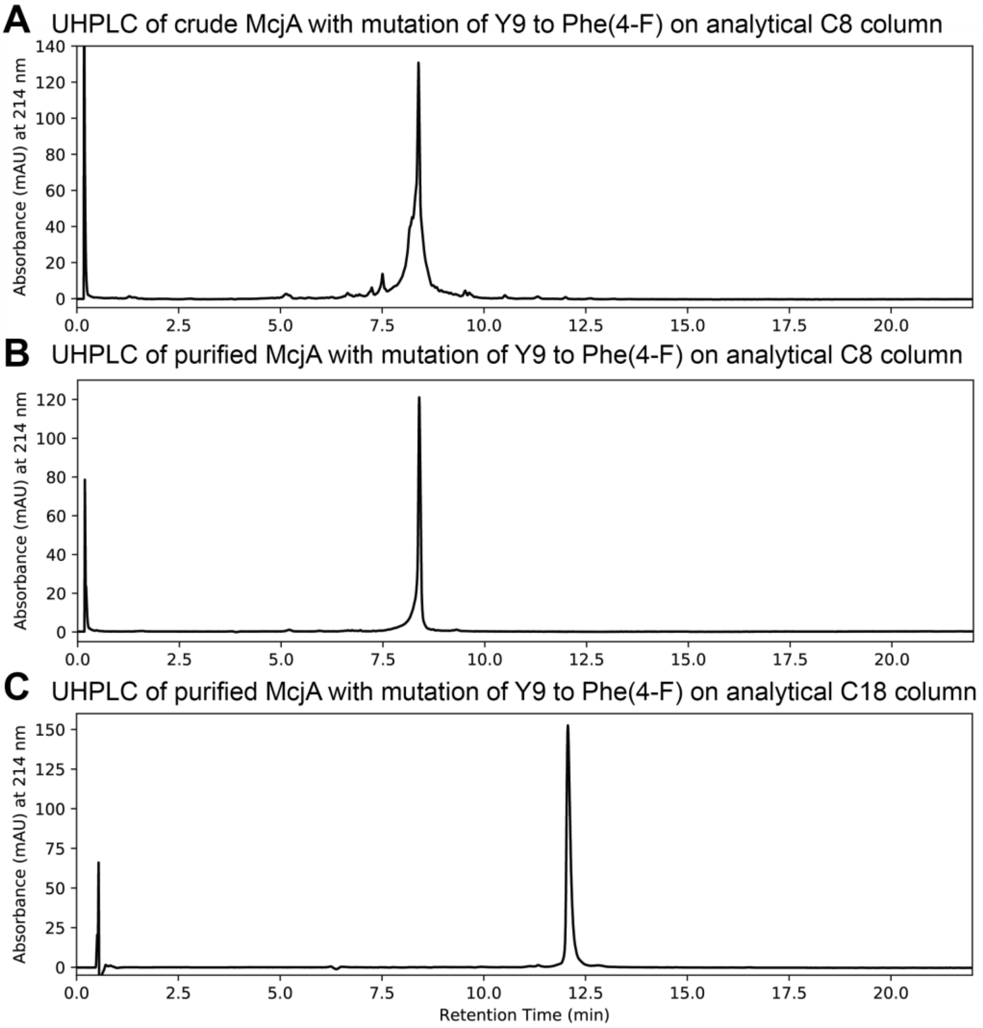

Fig. S5: UHPLC profile of McjA with mutation of Y9 to Phe(4-F): (A) crude sample on analytical C8 column (36% purity); (B) purified sample on analytical C8 column (>95% purity, R_t = 8.39 min); (C) purified sample on analytical C18 column (>95% purity, R_t = 12.07 min).

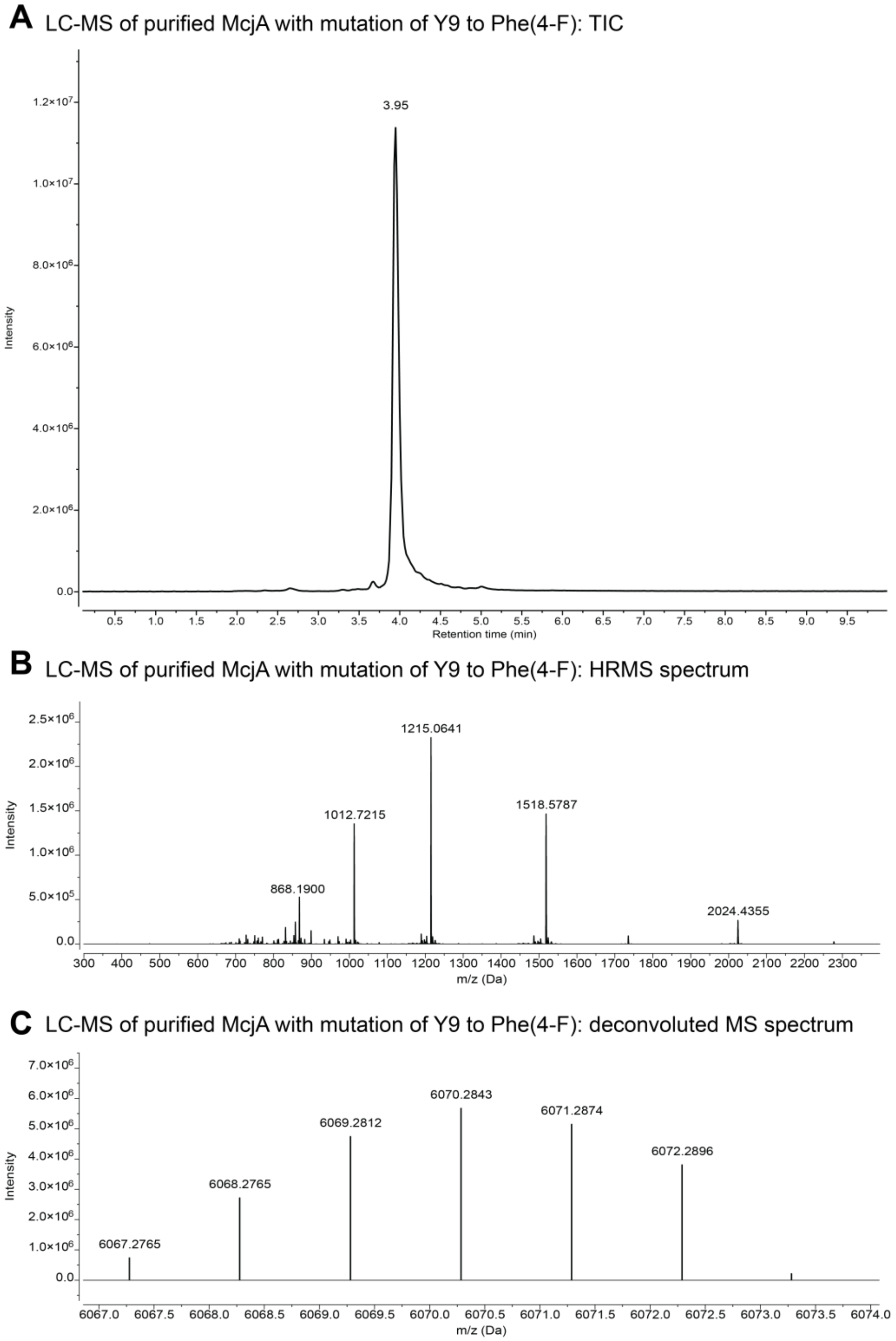

Fig. S6: LC-MS analysis of McjA with mutation of Y9 to Phe(4-F): (A) Total ion chromatogram (TIC). (B) MS spectrum. (C) deconvoluted MS spectrum: Monoisotopic mass (ESI+): calc. [$C_{280}H_{437}FN_{76}O_{74}$]: 6067.2752, found: 6067.2765; Average mass calc. [$C_{280}H_{437}FN_{76}O_{74}$]: 6071.0324.

2.1.4 McjA with Tyr(3-NO_2) = Y‘ (**4**)

Sequence: H_2N-IKHFHFNKLS SGKKNNVPSP AKGVIQIKKS ASQLTKGGAG HVPEY’FVGIG TPISFYG-OH

The peptide was prepared via Automated Fast-Flow Peptide Synthesis (AFPS) using HMPB ChemMatrix® resin (loading = 0.42 mmol/g, 85 mg, 36 µmol). The first amino acid was introduced as described in 1.3. The first amino acid was deprotected on the AFPS system. The synthesis using AFPS was performed following the general procedure in 1.2. until the point of Tyr9, where the non-canonical amino acid Fmoc-Tyr(3-NO_2)-OH was incorporated following the general procedure 1.4. The amino acid was deprotected on the AFPS system and the synthesis using AFPS was continued. The total synthesis time was approximately 5.5 h.

Cleavage of the peptidyl-resin (29% of total resin) afforded the crude peptide (48% purity (C8) by UHPLC). The peptide was purified using semi-preparative HPLC as specified in the general procedure 1.9. Fractions were analyzed by LC-HR-ESI-MS, combined, and lyophilized to obtain 3.3 mg (5% yield; >95% purity (C8) and >95% purity (C18) by UHPLC) of the desired peptide.

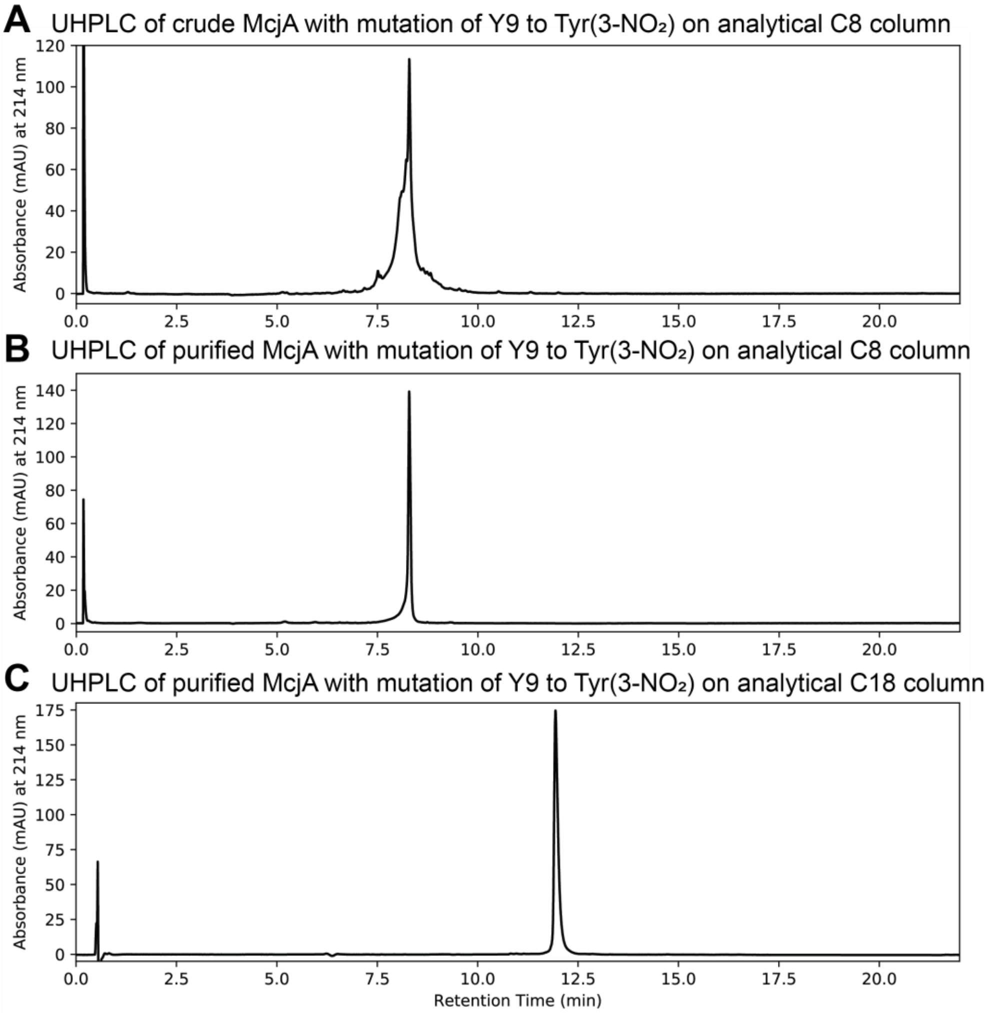

Fig. S7: UHPLC profile of McjA with mutation of Y9 to Tyr(3-NO_2): (A) crude sample on analytical C8 column (48% purity); (B) purified sample on analytical C8 column (>95% purity, R_t = 8.29 min); (C) purified sample on analytical C18 column (>95% purity, R_t = 11.94 min).

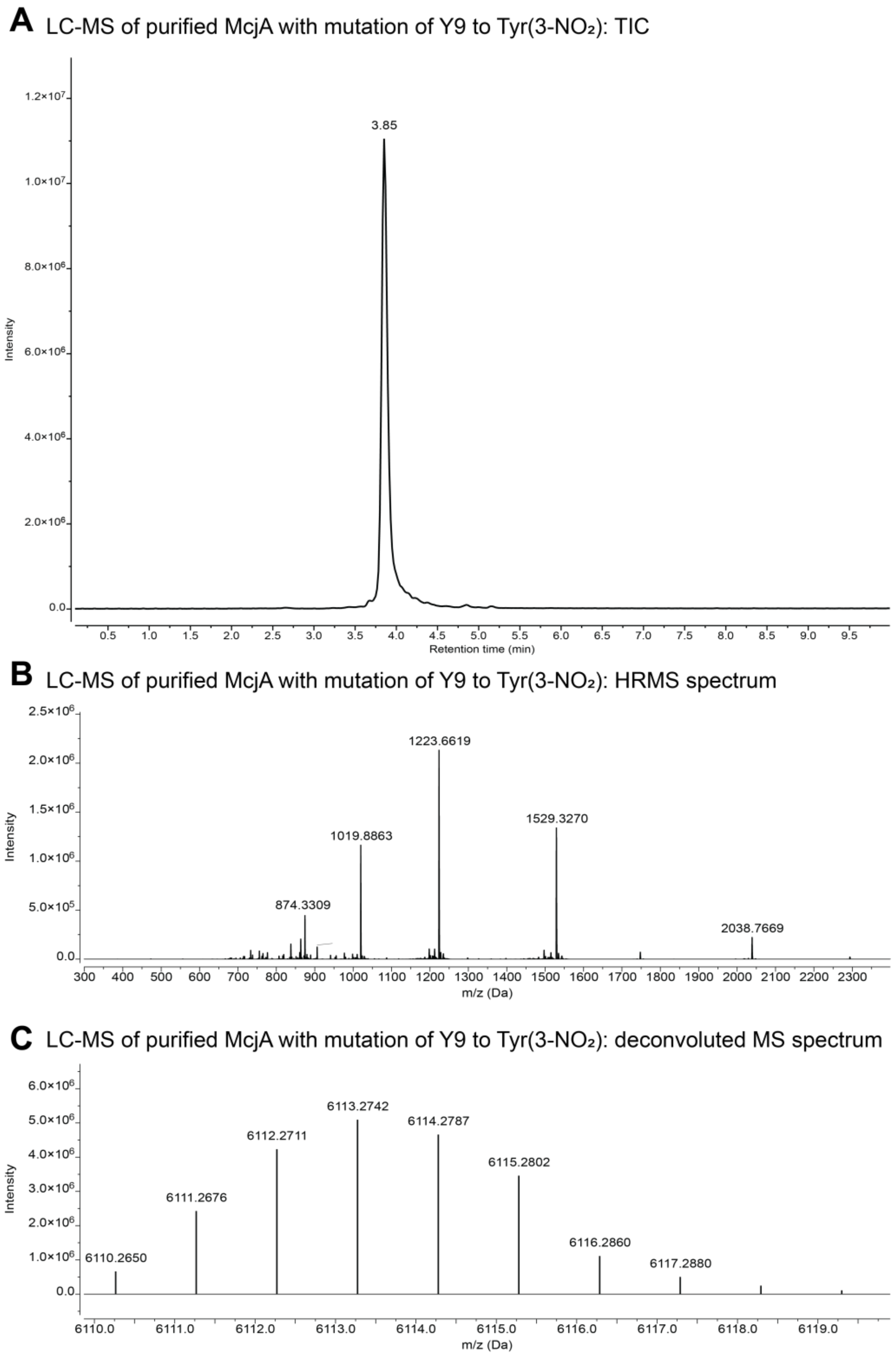

Fig. S8 : **LC-MS analysis of McjA with mutation of Y9 to Tyr(3-NO$_2$):** (A) Total ion chromatogram (TIC). (B) MS spectrum. (C) deconvoluted MS spectrum: Monoisotopic mass (ESI+): calc. [$C_{280}H_{437}N_{77}O_{77}$]: 6110.2647, found: 6110.2650; Average mass calc. [$C_{280}H_{437}N_{77}O_{77}$]: 6114.0380.

2.1.5 McjA with Phe(4-NH_2) = Y‘ (**5**)

Sequence: `H2N-IKHFHFNKLS SGKKNNVPSP AKGVIQIKKS ASQLTKGGAG HVPEY'FVGIG TPISFYG-OH`

The peptide was prepared via Automated Fast-Flow Peptide Synthesis (AFPS) using HMPB ChemMatrix® resin (loading = 0.42 mmol/g, 85 mg, 36 µmol). The first amino acid was introduced as described in 1.3. The first amino acid was deprotected on the AFPS system. The synthesis using AFPS was performed following the general procedure in 1.2. until the point of Tyr9, where the crude non-canonical amino acid Fmoc-Phe(4-NHTrt)-OH was incorporated by the following procedure: the non-canonical amino acid was incorporated using batch-synthesis after protecting the side chain: The Fmoc-Phe(4-NH_2)-OH (183.5 mg, 456 µmol, 12.7 eq.) was dissolved in 2 mL DCM. Trityl chloride (152.6 mg, 547 mmol, 15.2 eq.) and DIPEA (143 µL, 821 µmol, 22.9 eq.) were added and the reaction was stirred overnight. The DCM was evaporated, and the crude mixture was used for the coupling onto the resin. The Tyr-derivative was dissolved in 0.38 M PyAOP solution (536 µL in DMF, 6 eq.) and 536 µL DMF was added. DIPEA (75 µL, 12 eq.) was added, and the solution was shaken for one minute. The mixture was added to the resin and the coupling proceeded for 2 h. The resin was washed with DMF (3x 6 mL). The amino acid was deprotected on the AFPS system and the synthesis using AFPS was continued. The total synthesis time was approximately 5.5 h (excluding Trt protection of the side chain).

Cleavage of the peptidyl-resin (26% of total resin) afforded the crude peptide (30% purity (C8) by UHPLC). The peptide was purified using semi-preparative HPLC as specified in the general procedure 1.9. Fractions were analyzed by LC-HR-ESI-MS, combined, and lyophilized to obtain 2.4 mg (4% yield; 87% purity (C8) and >95% purity (C18) by UHPLC) of the desired peptide.

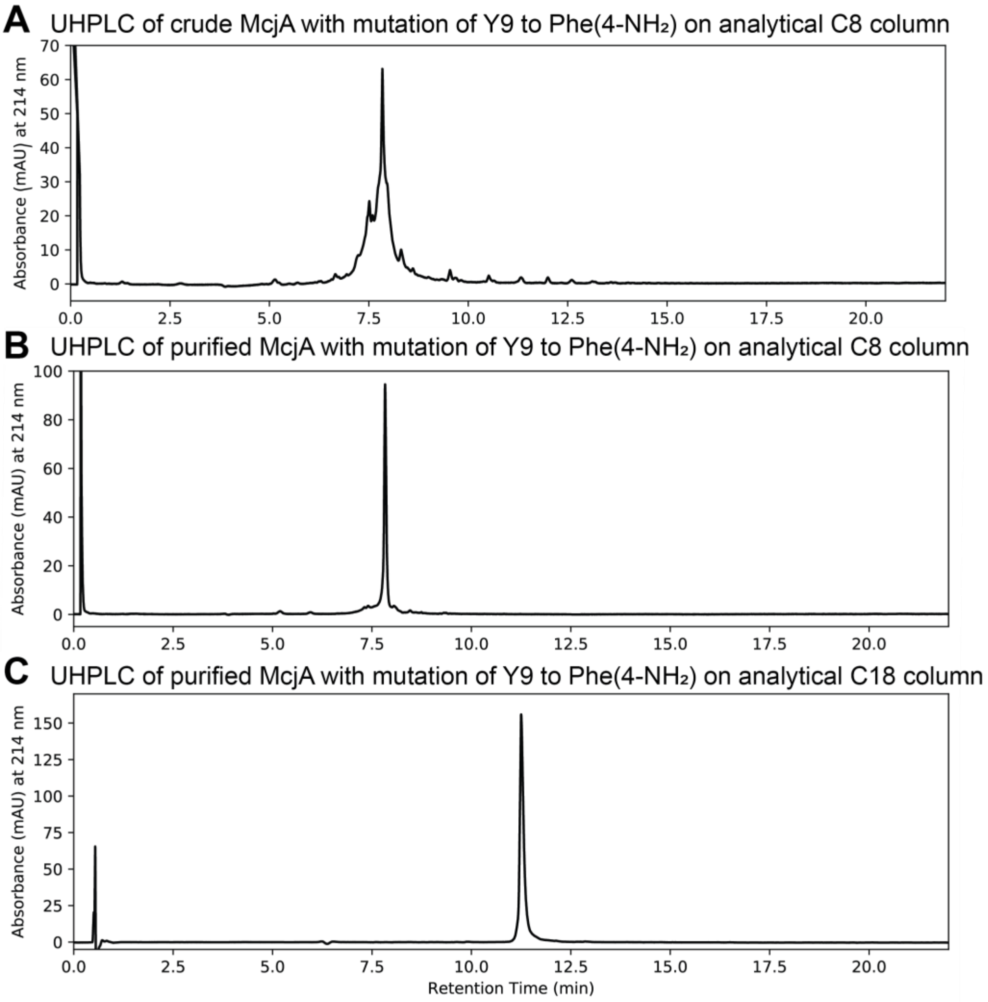

Fig. S9: **UHPLC profile of McjA with mutation of Y9 to Phe(4-NH_2):** (A) crude sample on analytical C8 column (30% purity); (B) purified sample on analytical C8 column (87% purity, R_t = 7.83 min); (C) purified sample on analytical C18 column (>95% purity, R_t = 11.25 min).

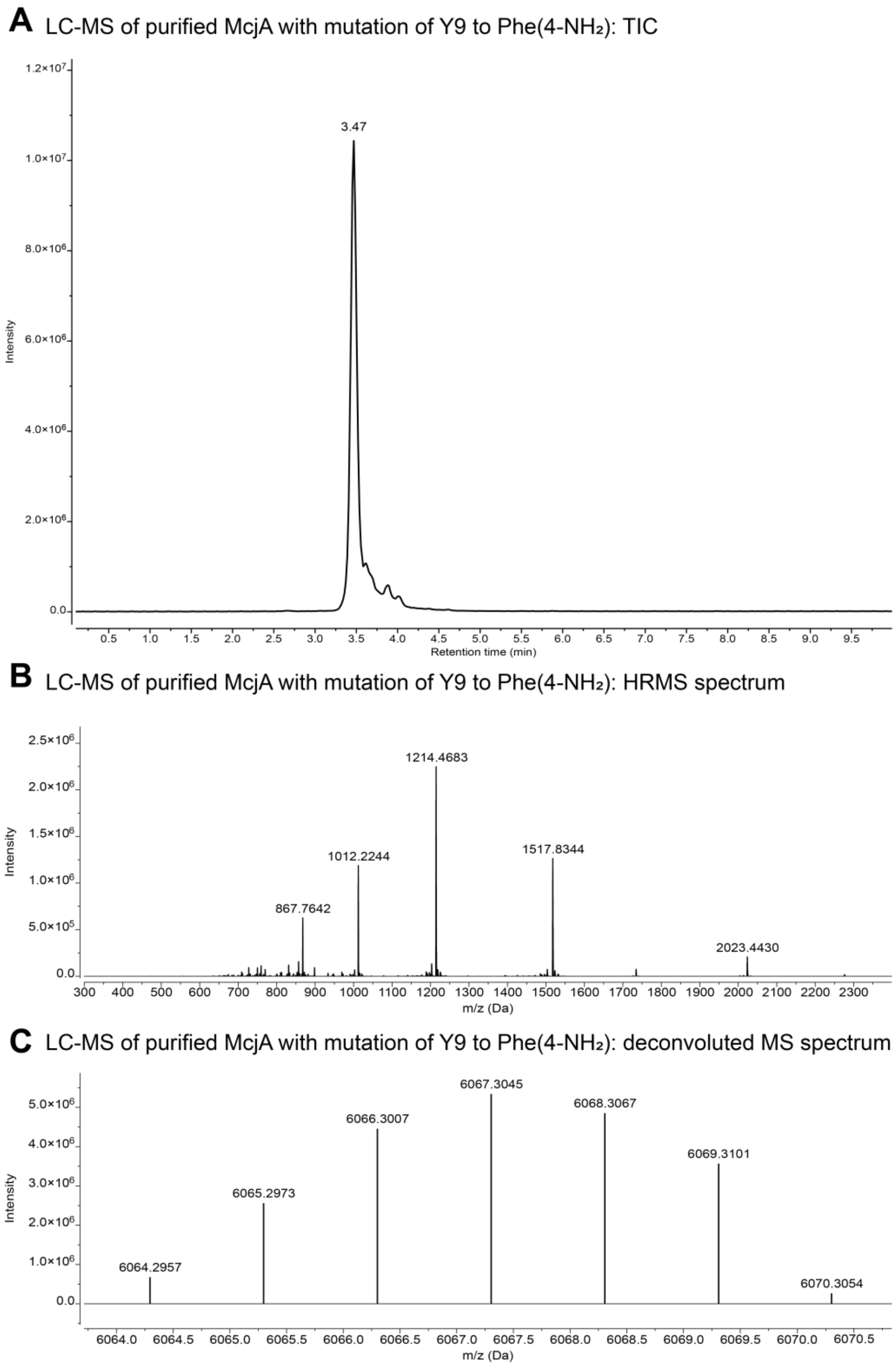

Fig. S10: LC-MS analysis of McjA with mutation of Y9 to Phe(4-NH$_2$): (A) Total ion chromatogram (TIC). (B) MS spectrum. (C) deconvoluted MS spectrum: Monoisotopic mass (ESI+): calc. [$C_{280}H_{439}N_{77}O_{74}$]: 6064.2956, found: 6064.2957; Average mass calc. [$C_{280}H_{439}N_{77}O_{74}$]: 6068.0570.

2.1.6 McjA with Tyr(3-*t*Bu) = Y‘ (**6**)

Sequence: H_2N-IKHFHFNKLS SGKKNNVPSP AKGVIQIKKS ASQLTKGGAG HVPEY’FVGIG TPISFYG-OH

The peptide was prepared via Automated Fast-Flow Peptide Synthesis (AFPS) using HMPB ChemMatrix® resin (loading = 0.42 mmol/g, 86 mg, 36 µmol). The first amino acid was introduced as described in 1.3. The first amino acid was deprotected on the AFPS system. The synthesis using AFPS was performed following the general procedure in 1.2. until the point of Tyr9, where the non-canonical amino acid Fmoc-Tyr(3-*t*Bu)-OH was incorporated following the general procedure 1.4. The amino acid was deprotected on the AFPS system and the synthesis using AFPS was continued. The total synthesis time was approximately 5.5 h.

Cleavage of the peptidyl-resin (29% of total resin) afforded the crude peptide (35% purity (C8) by UHPLC). The peptide was purified using semi-preparative HPLC as specified in the general procedure 1.9. Fractions were analyzed by LC-HR-ESI-MS, combined, and lyophilized to obtain 1.2 mg (2% yield; >95% purity (C8) and >95% purity (C18) by UHPLC) of the desired peptide.

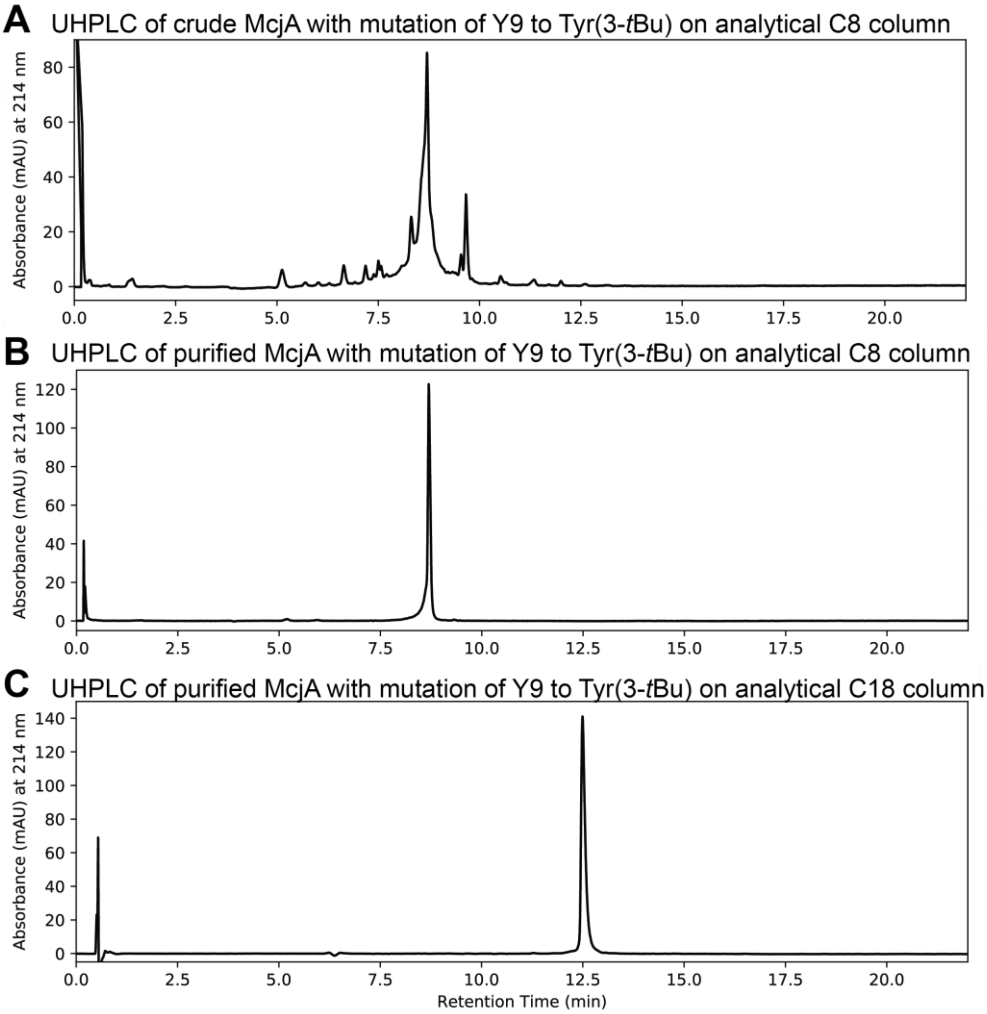

Fig. S11: UHPLC profile of McjA with mutation of Y9 to Tyr(3-*t*Bu): (A) crude sample on analytical C8 column (35% purity); (B) purified sample on analytical C8 column (>95% purity, R_t = 8.69 min); (C) purified sample on analytical C18 column (>95% purity, R_t = 12.49 min).

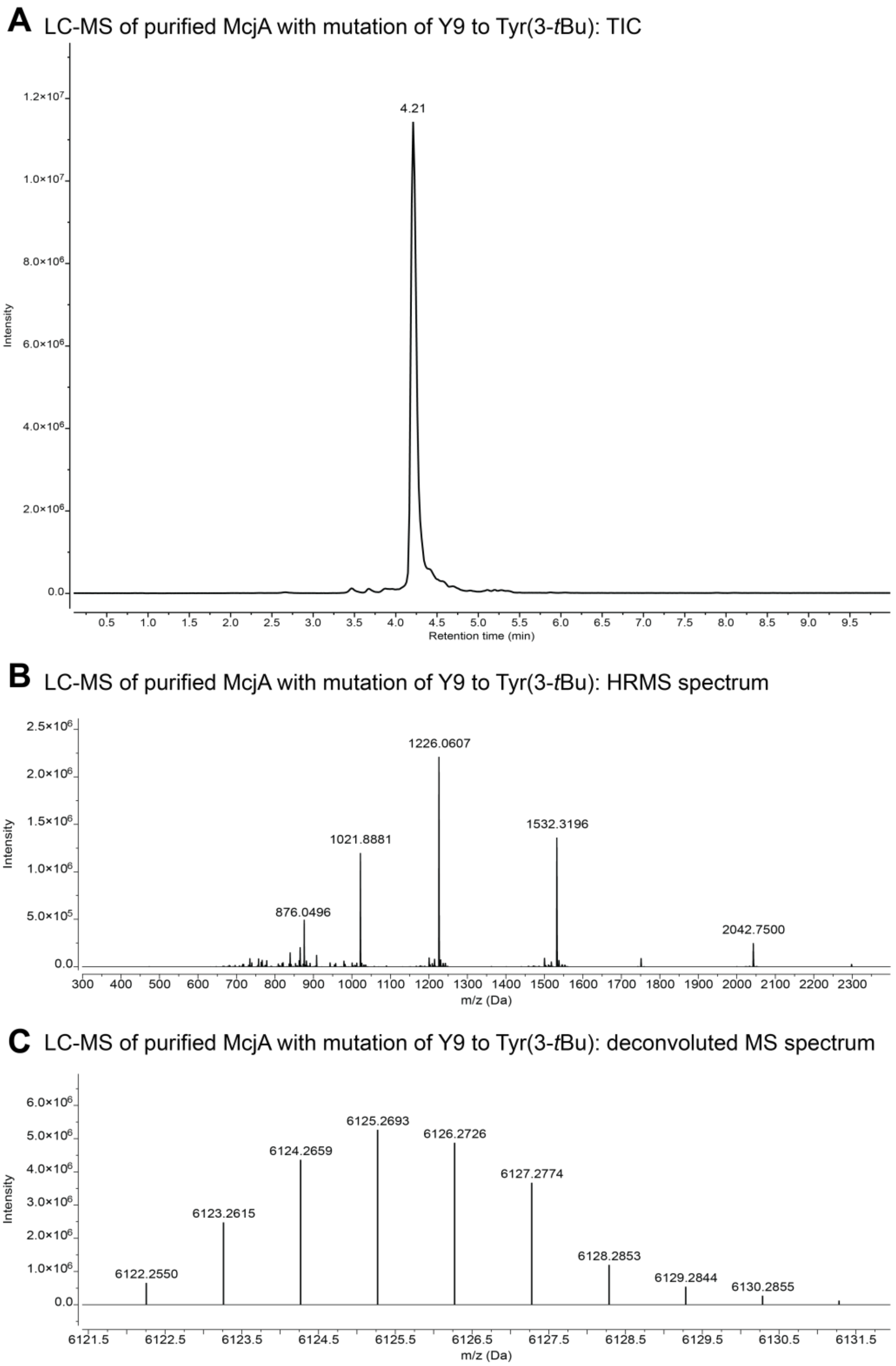

Fig. S12: LC-MS analysis of McjA with mutation of Y9 to Tyr(3-*t*Bu): (A) Total ion chromatogram (TIC). (B) MS spectrum. (C) deconvoluted MS spectrum: Monoisotopic mass (ESI+): calc. [$C_{284}H_{446}N_{76}O_{75}$]: 6121.3422, found: 6122.2550; Average mass calc. [$C_{284}H_{446}N_{76}O_{75}$]: 6125.1490.

2.1.7 McjA with Phe(3-OH) = Y‘ (**7**)

Sequence: H_2N-IKHFHFNKLS SGKKNNVPSP AKGVIQIKKS ASQLTKGGAG HVPEY’FVGIG TPISFYG-OH

The peptide was prepared via Automated Fast-Flow Peptide Synthesis (AFPS) using HMPB ChemMatrix® resin (loading = 0.42 mmol/g, 81 mg, 34 µmol). The first amino acid was introduced as described in 1.3. The first amino acid was deprotected on the AFPS system. The synthesis using AFPS was performed following the general procedure in 1.2. until the point of Tyr9, where the non-canonical amino acid Fmoc-Phe(3-OH)-OH was incorporated following the general procedure 1.4. The amino acid was deprotected on the AFPS system and the synthesis using AFPS was continued. The total synthesis time was approximately 5.5 h.

Cleavage of the peptidyl-resin (34% of total resin) afforded the crude peptide (35% purity (C8) by UHPLC). The peptide was purified using semi-preparative HPLC as specified in the general procedure 1.9. Fractions were analyzed by LC-HR-ESI-MS, combined, and lyophilized to obtain 3.2 mg (5% yield; 90% purity (C8) and >95% purity (C18) by UHPLC) of the desired peptide.

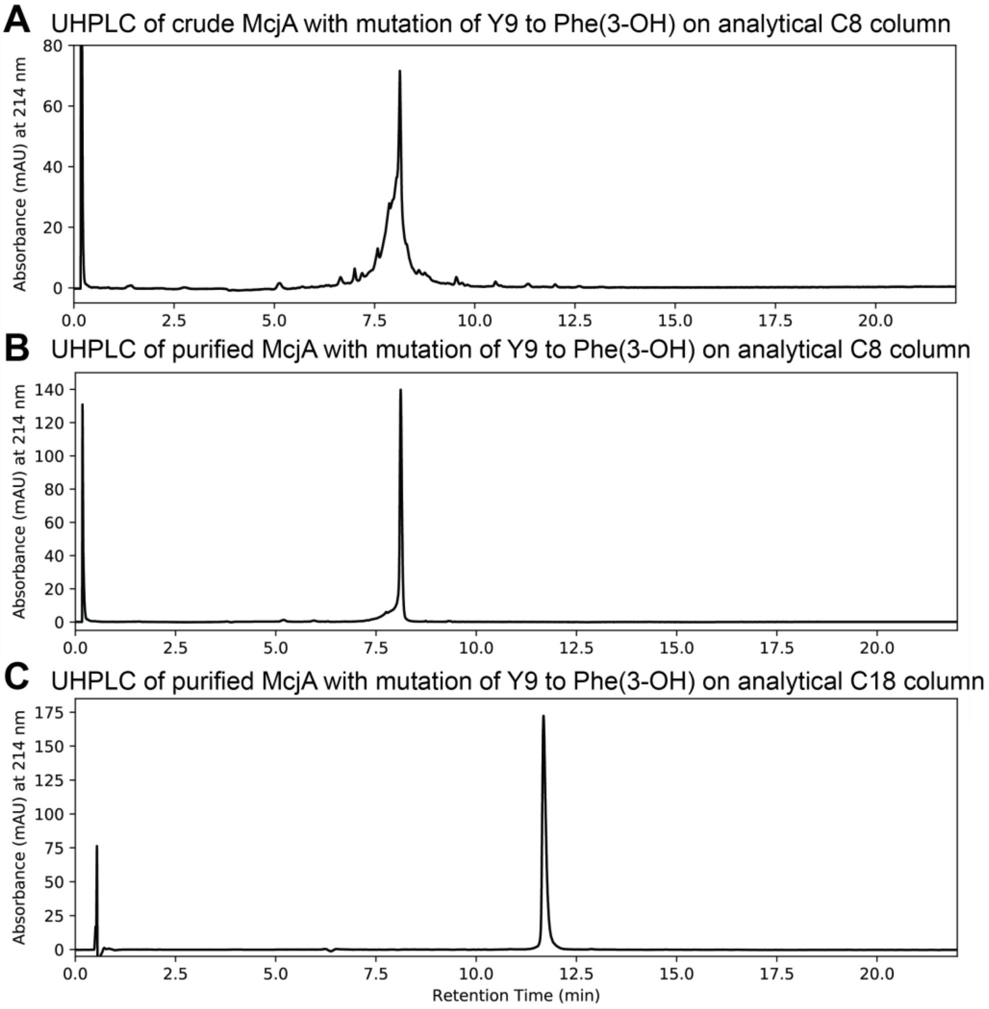

Fig. S13: UHPLC profile of McjA with mutation of Y9 to Phe(3-OH): (A) crude sample on analytical C8 column (35% purity); (B) purified sample on analytical C8 column (90% purity, R_t = 8.12 min); (C) purified sample on analytical C18 column (>95% purity, R_t = 11.68 min).

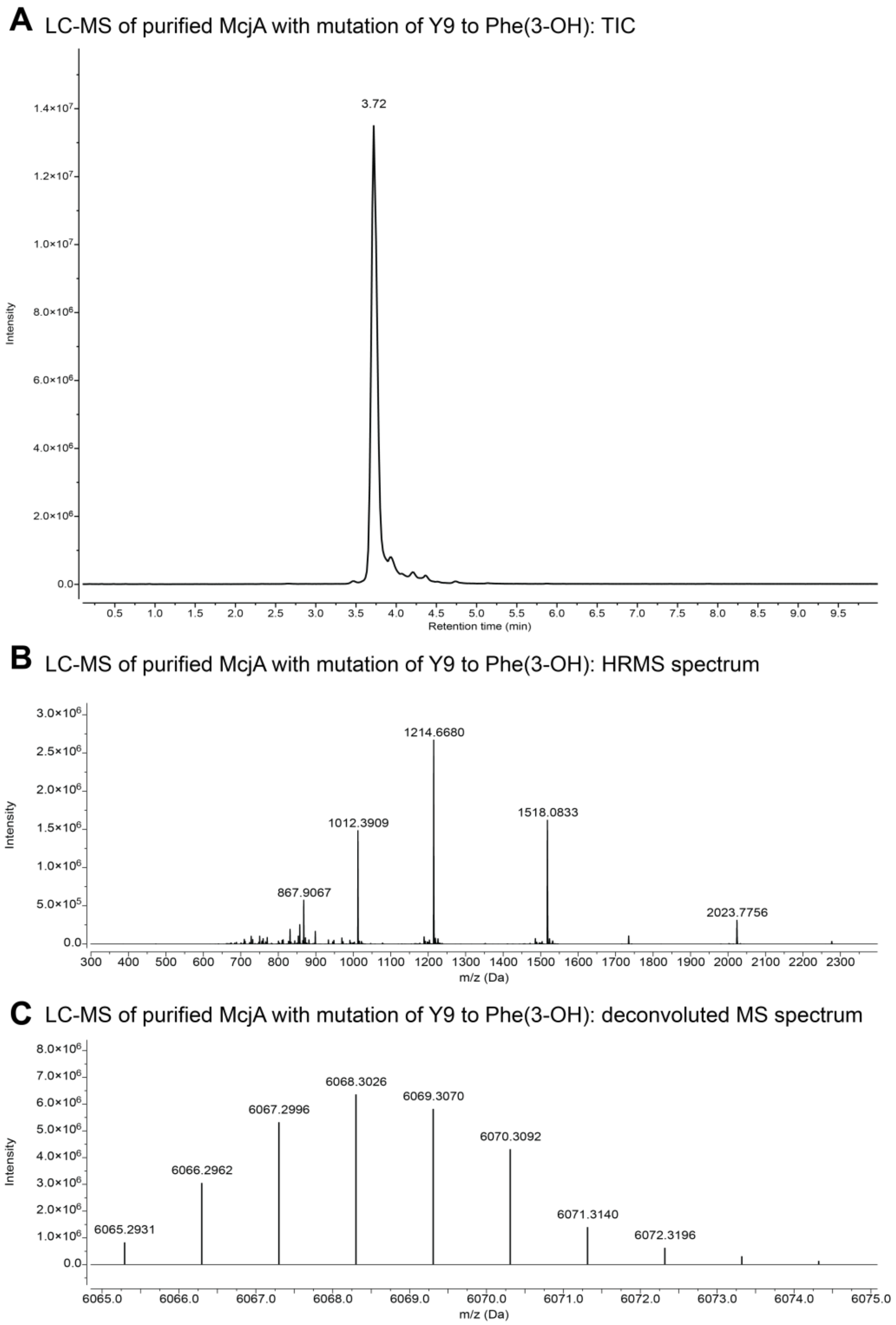

Fig. S14: LC-MS analysis of McjA with mutation of Y9 to Phe(3-OH): (A) Total ion chromatogram (TIC). (B) MS spectrum. (C) deconvoluted MS spectrum: Monoisotopic mass (ESI+): calc. [$C_{280}H_{438}N_{76}O_{75}$]: 6065.2796, found: 6065.2931; Average mass calc. [$C_{280}H_{438}N_{76}O_{75}$]: 6069.0410.

2.1.8 McjA with Phe (**8**)

Sequence: H_2N-IKHFHFNKLS SGKKNNVPSP AKGVIQIKKS ASQLTKGGAG HVPEFFVGIG TPISFYG-OH

The peptide was prepared via Automated Fast-Flow Peptide Synthesis (AFPS) using HMPB ChemMatrix® resin (loading = 0.42 mmol/g, 84 mg, 35 µmol). The first amino acid was introduced as described in 1.3. The first amino acid was deprotected on the AFPS system. The synthesis using AFPS was performed following the general procedure in 1.2. The total synthesis time was approximately 3.5 h

Cleavage of the peptidyl-resin (% of total resin) afforded the crude peptide (35% purity (C8) by UHPLC). The peptide was purified using semi-preparative HPLC as specified in the general procedure 1.9. Fractions were analyzed by LC-HR-ESI-MS, combined, and lyophilized to obtain 2.3 mg (3% yield; >95% purity (C8) and >95% purity (C18) by UHPLC) of the desired peptide.

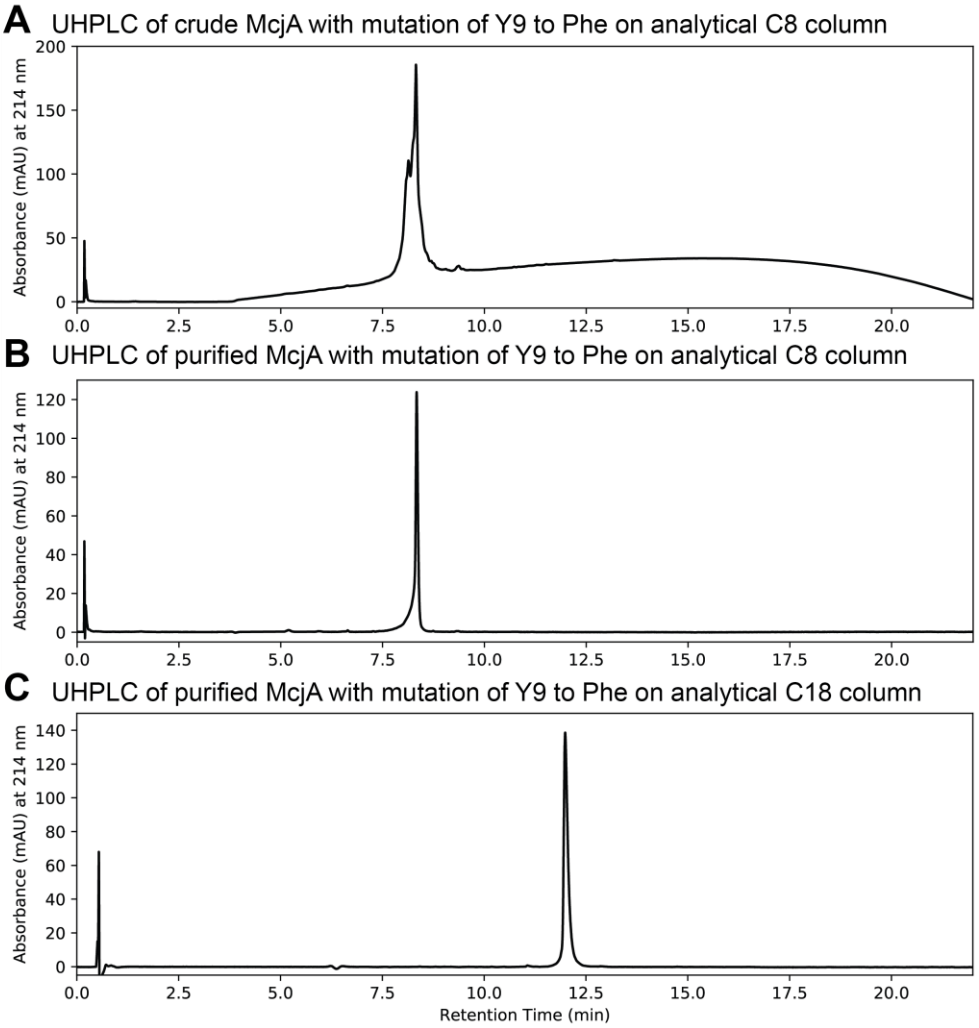

Fig. S15: UHPLC profile of McjA with mutation of Y9 to Phe: (A) crude sample on analytical C8 column (35% purity); (B) purified sample on analytical C8 column (>95% purity, R_t = 8.34 min); (C) purified sample on analytical C18 column (>95% purity, R_t = 11.99 min).

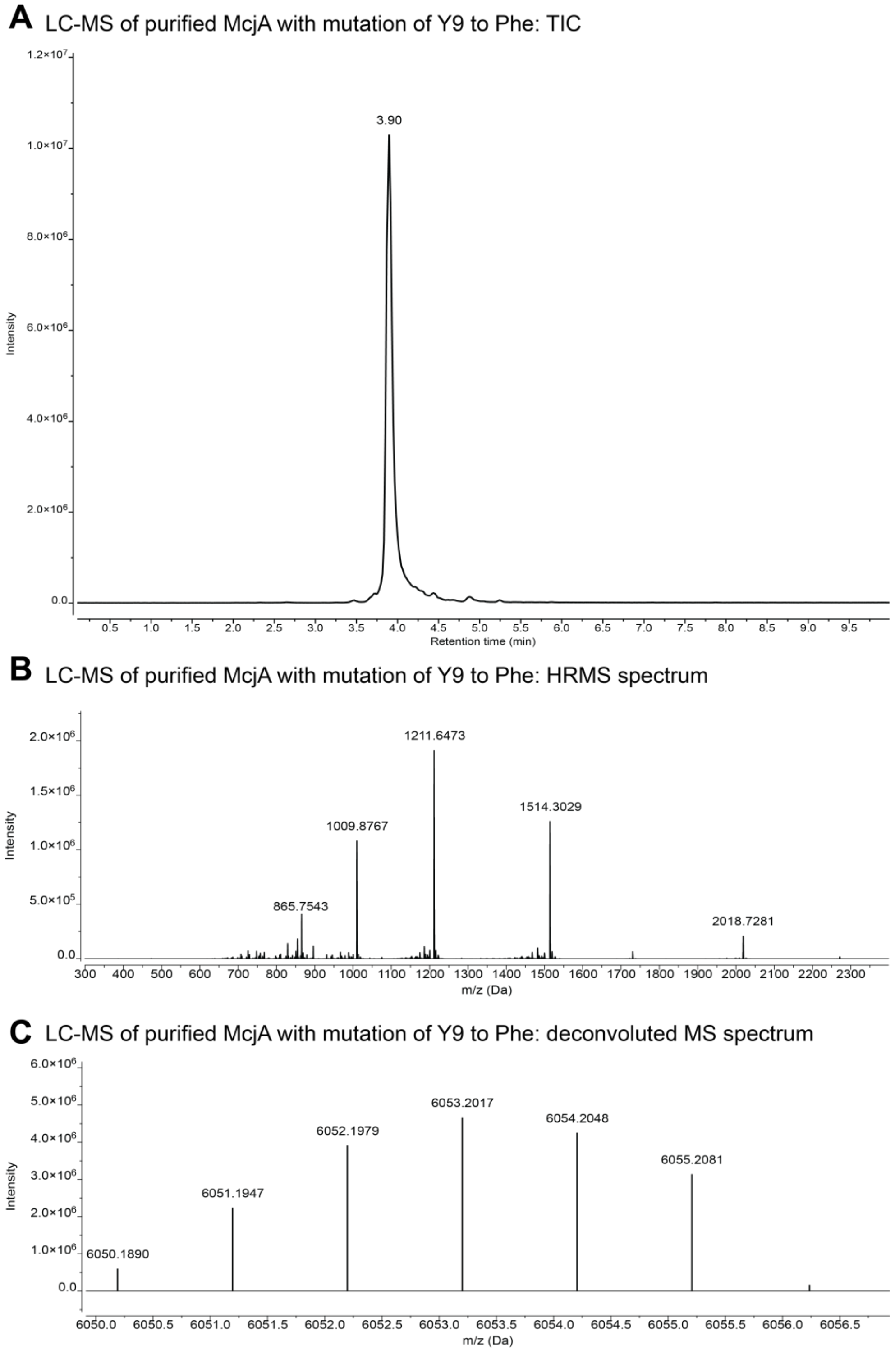

Fig. S16: LC-MS analysis of McjA with mutation of Y9 to Phe: (A) Total ion chromatogram (TIC). (B) MS spectrum. (C) deconvoluted MS spectrum: Monoisotopic mass (ESI+): calc. [$C_{280}H_{438}N_{76}O_{74}$]: 6049.2847, found: 6050.1890; Average mass calc. [$C_{280}H_{438}N_{76}O_{74}$]: 6053.0420.

2.2 Synthesis of His-derivatives of Link-McjA[5]

2.2.1 Link-McjA with L-His12 (**9**)

Sequence: `H2N-IKHFHFNKLS SGKKNNVPSP AKGVIQIKKS ASQLTKGGAG HVPEYFVHFG IPISFYG-OH`

The peptide was prepared via Automated Fast-Flow Peptide Synthesis (AFPS) using NovaPEG HMPB resin (loading = 0.62 mmol/g, 79 mg, 49 μmol). The first amino acid was introduced as described in 1.3. The first amino acid was deprotected on the AFPS system. The synthesis using AFPS was performed following the general procedure in 1.2. until the point of His-12, where Fmoc-L-His(Trt)-OH was incorporated following the general procedure 1.4. The amino acid was deprotected on the AFPS system and the synthesis using AFPS was continued. The total synthesis time was approximately 5.5 h.

Cleavage of the peptidyl-resin (27% of total resin) afforded the crude peptide (28% purity (C8) by UHPLC). The peptide was purified using semi-preparative HPLC as specified in the general procedure 1.9. Fractions were analyzed by LC-HR-ESI-MS, combined, and lyophilized to obtain 0.68 mg (0.8% yield; 86% purity (C8) and >95% purity (C18) by UHPLC) of the desired peptide.

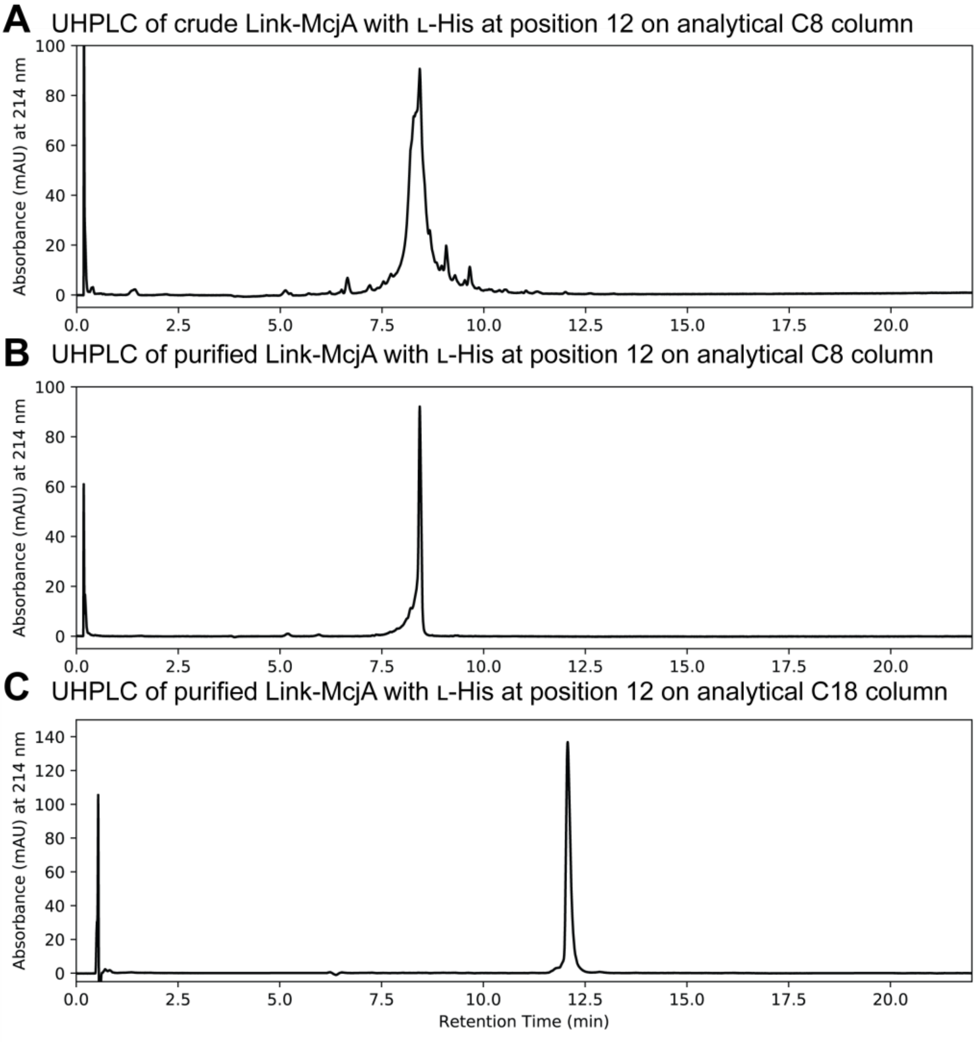

Fig. S17: UHPLC profile of Link-McjA with L-His at position 12: (A) crude sample on analytical C8 column (28% purity); (B) purified sample on analytical C8 column (86% purity, R_t = 8.43 min); (C) purified sample on analytical C18 column (>95% purity, R_t = 12.07 min).

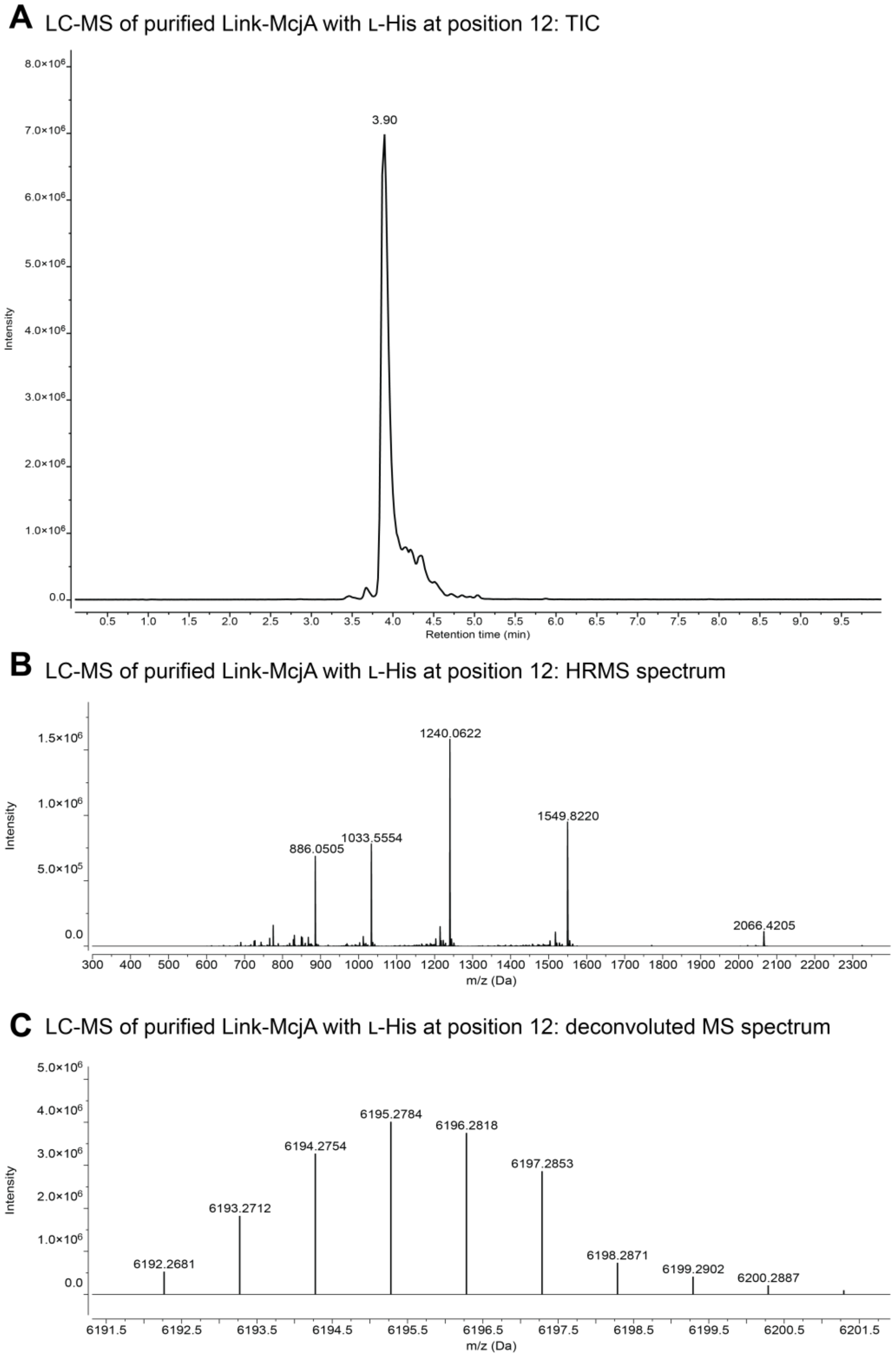

Fig. S18: **LC-MS analysis of Link-McjA with L-His at position 12:** (A) Total ion chromatogram (TIC). (B) MS spectrum. (C) deconvoluted MS spectrum: Monoisotopic mass (ESI+): calc. [$C_{289}H_{444}N_{78}O_{74}$]: 6191.3378, found: 6192.2681; Average mass calc. [$C_{289}H_{444}N_{78}O_{74}$]: 6195.2030.

2.2.2 Link-McjA with D-His12 = h (**10**)

Sequence: H_2N-IKHFHFNKLS SGKKNNVPSP AKGVIQIKKS ASQLTKGGAG HVPEYFVhFG IPISFYG-OH

The peptide was prepared via Automated Fast-Flow Peptide Synthesis (AFPS) using NovaPEG HMPB resin (loading = 0.62 mmol/g, 80 mg, 50 µmol). The first amino acid was introduced as described in 1.3. The first amino acid was deprotected on the AFPS system. The synthesis using AFPS was performed following the general procedure in 1.2. until the point of His-12, where Fmoc-D-His(Trt)-OH was incorporated following the general procedure 1.4. The amino acid was deprotected on the AFPS system and the synthesis using AFPS was continued. The total synthesis time was approximately 5.5 h.

Cleavage of the peptidyl-resin (26% of total resin) afforded the crude peptide (27% purity (C8) by UHPLC). The peptide was purified using semi-preparative HPLC as specified in the general procedure 1.9. Fractions were analyzed by LC-HR-ESI-MS, combined, and lyophilized to obtain 1.0 mg (1.3% yield; 84% purity (C8) and >95% purity (C18) by UHPLC) of the desired peptide.

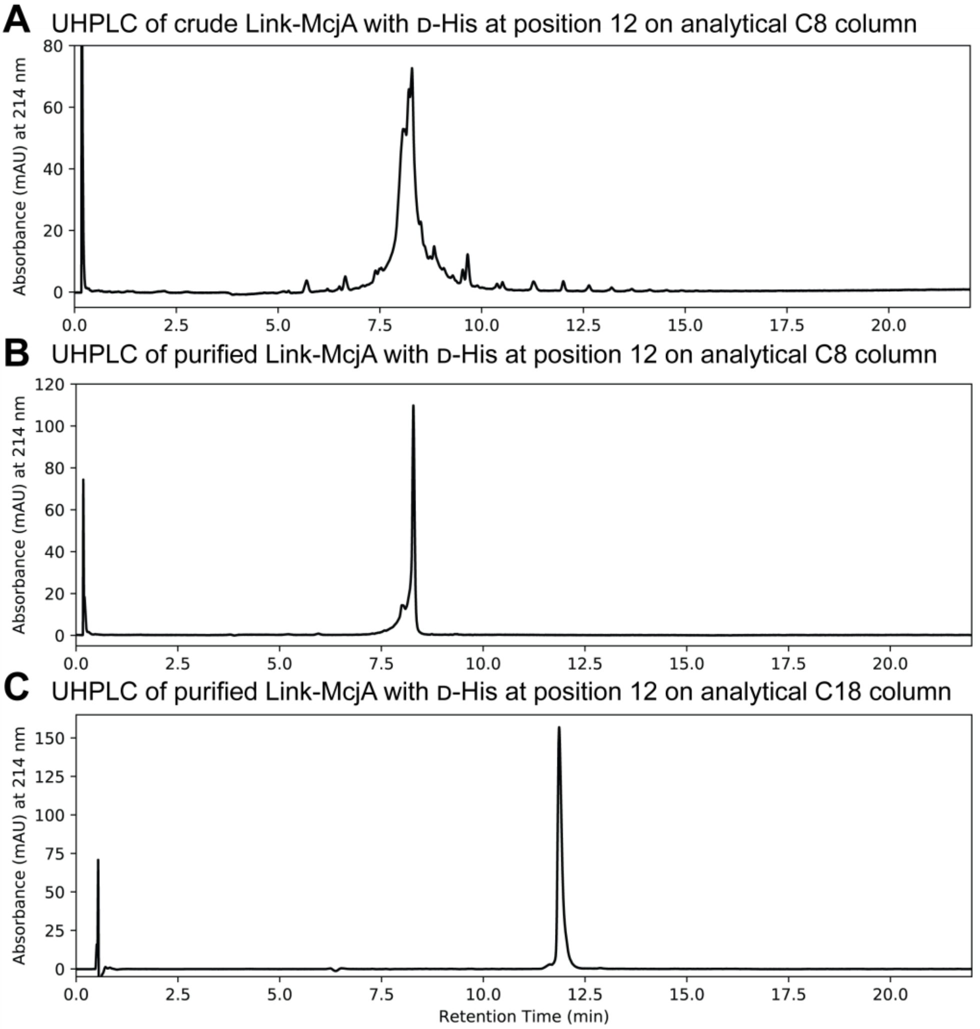

Fig. S19: UHPLC profile of Link-McjA with D-His at position 12: (A) crude sample on analytical C8 column (27% purity); (B) purified sample on analytical C8 column (84% purity, R_t = 8.29 min); (C) purified sample on analytical C18 column (>95% purity, R_t = 11.86 min).

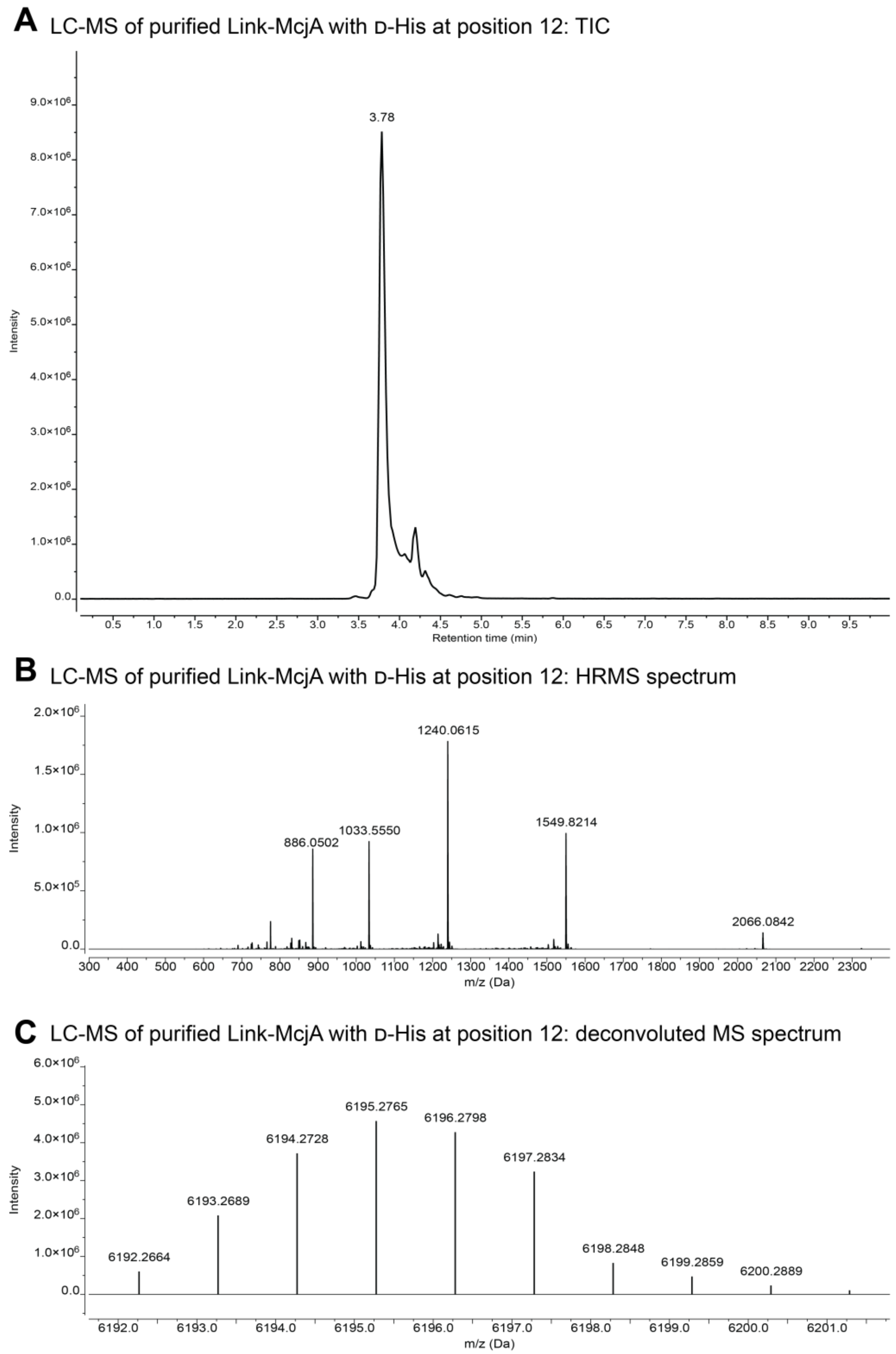

Fig. S20: LC-MS analysis of Link-McjA with D-His at position 12: (A) Total ion chromatogram (TIC). (B) MS spectrum. (C) deconvoluted MS spectrum: Monoisotopic mass (ESI+): calc. [$C_{289}H_{444}N_{78}O_{74}$]: 6191.3378, found: 6192.2664; Average mass calc. [$C_{289}H_{444}N_{78}O_{74}$]: 6195.2030.

2.2.3 Link-McjA with 3-(4-Thiazolyl)-L-alanine = H’ (**11**)

Sequence: H_2N-IKHFHFNKLS SGKKNNVPSP AKGVIQIKKS ASQLTKGGAG HVPEYFVH’FG IPISFYG-OH

The peptide was prepared via Automated Fast-Flow Peptide Synthesis (AFPS) using NovaPEG HMPB resin (loading = 0.62 mmol/g, 81 mg, 50 μmol). The first amino acid was introduced as described in 1.3. The first amino acid was deprotected on the AFPS system. The synthesis using AFPS was performed following the general procedure in 1.2. until the point of His-12, where Fmoc-3-(4-Thiazolyl)-L-alanine-OH was incorporated following the general procedure 1.4. The amino acid was deprotected on the AFPS system and the synthesis using AFPS was continued. The total synthesis time was approximately 5.5 h.

Cleavage of the peptidyl-resin (27% of total resin) afforded the crude peptide (21% purity (C8) by UHPLC). The peptide was purified using semi-preparative HPLC as specified in the general procedure 1.9. Fractions were analyzed by LC-HR-ESI-MS, combined, and lyophilized to obtain 0.18 mg (0.2% yield; >95% purity (C8) and >95% purity (C18) by UHPLC) of the desired peptide.

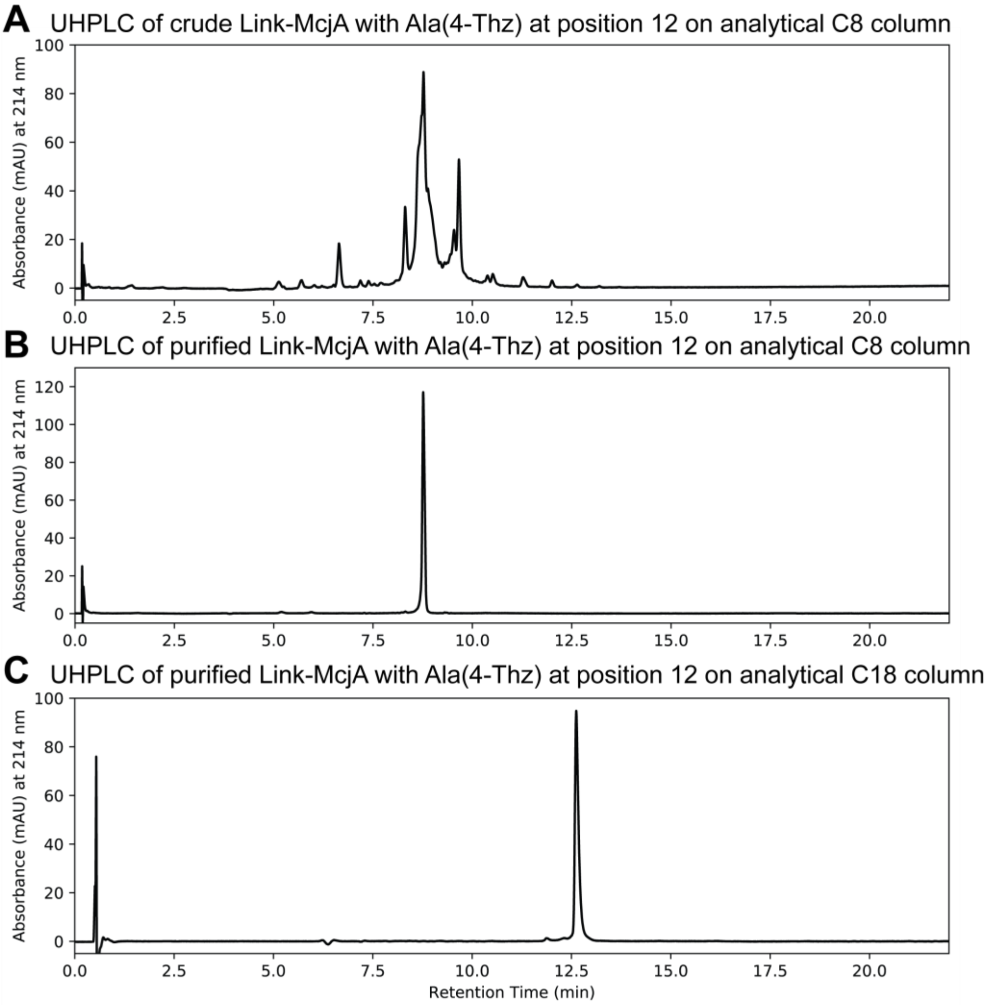

Fig. S21: UHPLC profile of Link-McjA with Ala(4-Thz) at position 12: (A) crude sample on analytical C8 column (21% purity); (B) purified sample on analytical C8 column (>95% purity, R_t = 8.76 min); (C) purified sample on analytical C18 column (>95% purity, R_t = 12.62 min).

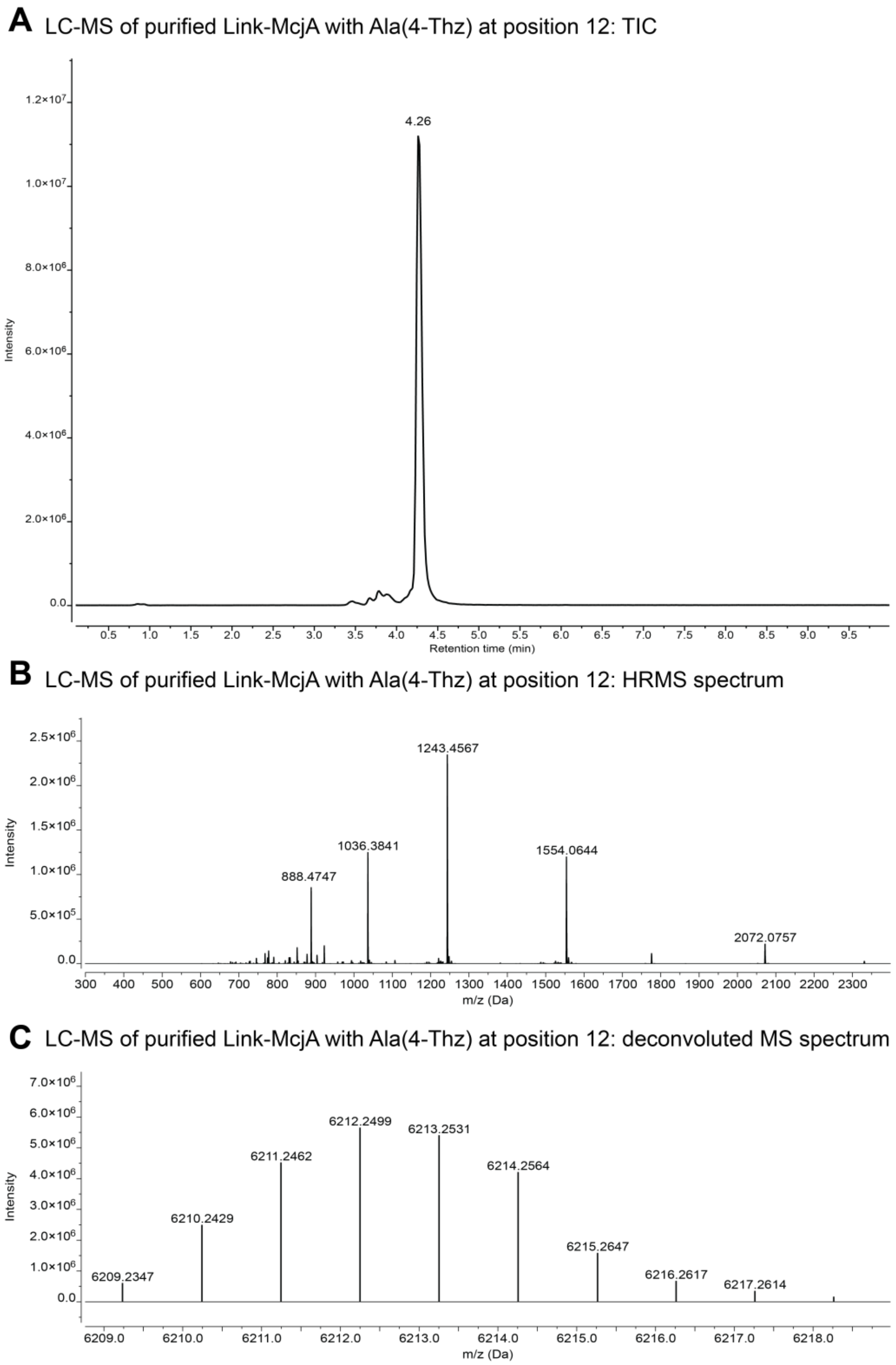

Fig. S22: **LC-MS analysis of Link-McjA with Ala(4-Thz) at position 12:** (A) Total ion chromatogram (TIC). (B) MS spectrum. (C) deconvoluted MS spectrum: Monoisotopic mass (ESI+): calc. [$C_{289}H_{443}N_{77}O_{74}S$]: 6208.2989, found: 6209.2347; Average mass calc. [$C_{289}H_{443}N_{77}O_{74}S$]: 6212.2480.

2.2.4 Link-McjA with 3-Thienyl-L-alanine = H’ (**12**)

Sequence: H_2N-IKHFHFNKLS SGKKNNVPSP AKGVIQIKKS ASQLTKGGAG HVPEYFVH’FG IPISFYG-OH

The peptide was prepared via Automated Fast-Flow Peptide Synthesis (AFPS) using NovaPEG HMPB resin (loading = 0.62 mmol/g, 81 mg, 50 μmol). The first amino acid was introduced as described in 1.3. The first amino acid was deprotected on the AFPS system. The synthesis using AFPS was performed following the general procedure in 1.2. until the point of His-12, where Fmoc-3-Thienyl-L-alanine-OH was incorporated following the general procedure 1.4. The amino acid was deprotected on the AFPS system and the synthesis using AFPS was continued. The total synthesis time was approximately 5.5 h.

Cleavage of the peptidyl-resin (25% of total resin) afforded the crude peptide (24% purity (C8) by UHPLC). The peptide was purified using semi-preparative HPLC as specified in the general procedure 1.9. Fractions were analyzed by LC-HR-ESI-MS, combined, and lyophilized to obtain 0.18 mg (0.2% yield; >95% purity (C8) and >95% purity (C18) by UHPLC) of the desired peptide.

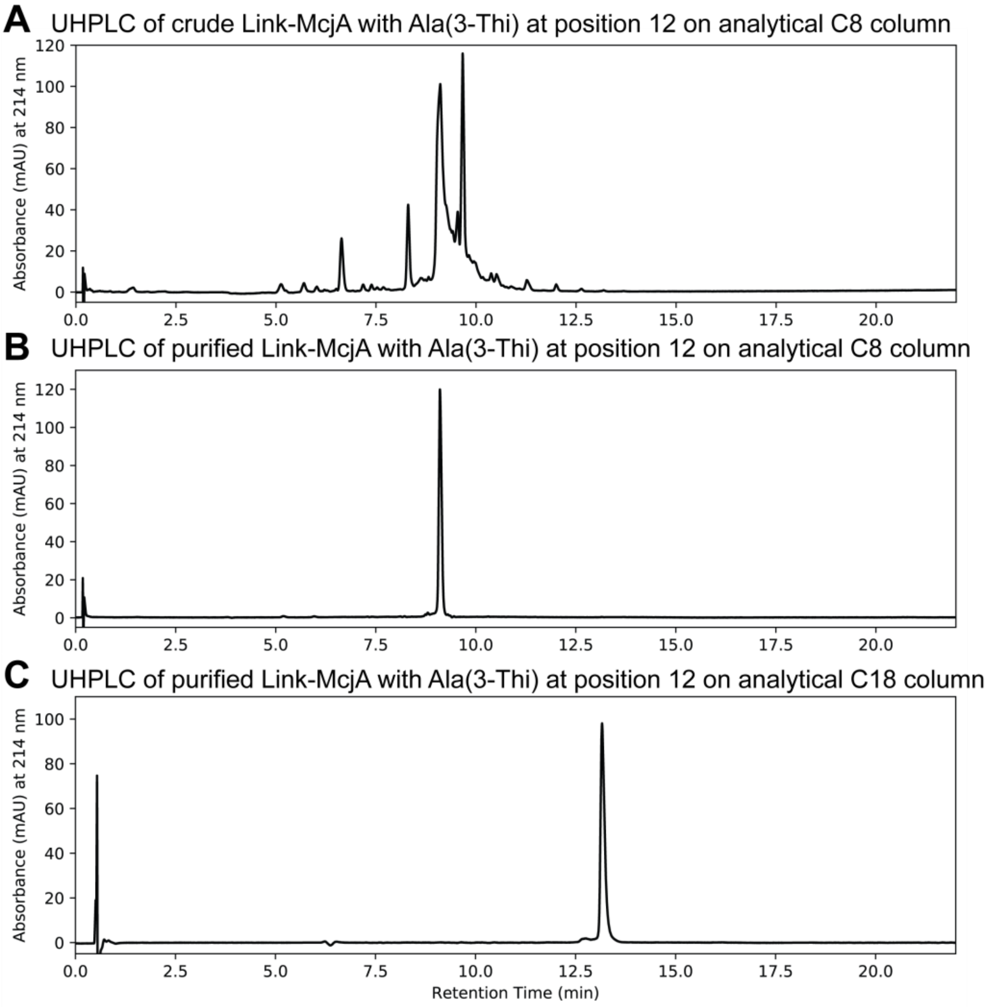

Fig. S23: UHPLC profile of Link-McjA with Ala(3-Thi) at position 12: (A) crude sample on analytical C8 column (24% purity); (B) purified sample on analytical C8 column (>95% purity, R_t = 9.10 min); (C) purified sample on analytical C18 column (>95% purity, R_t = 13.16 min).

A LC-MS of purified Link-McjA with Ala(3-Thi) at position 12: TIC

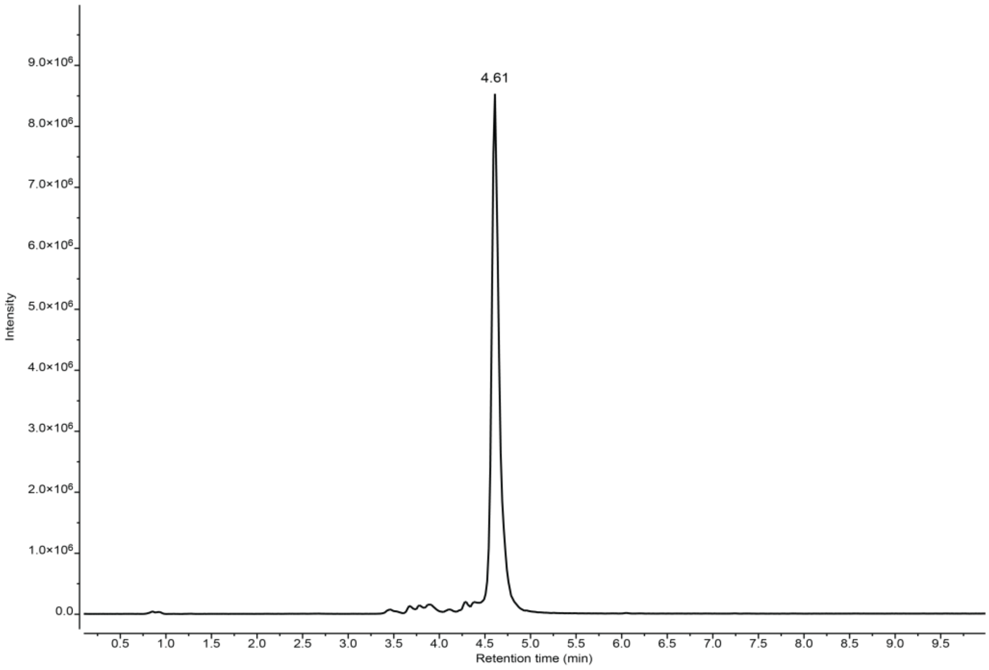

B LC-MS of purified Link-McjA with Ala(3-Thi) at position 12: HRMS spectrum

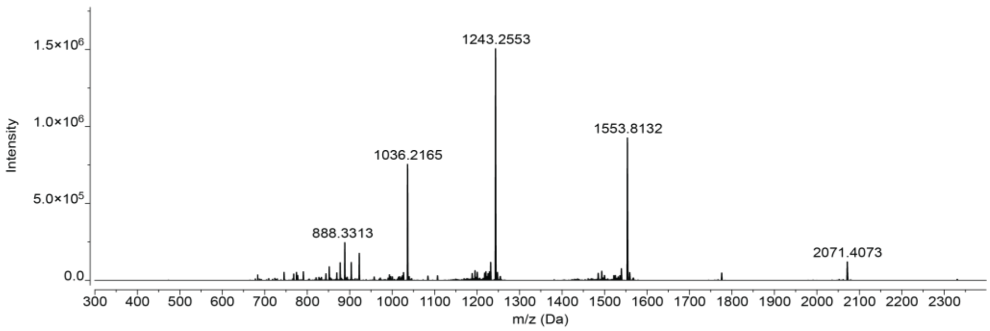

C LC-MS of purified Link-McjA with Ala(3-Thi) at position 12: deconvoluted MS spectrum

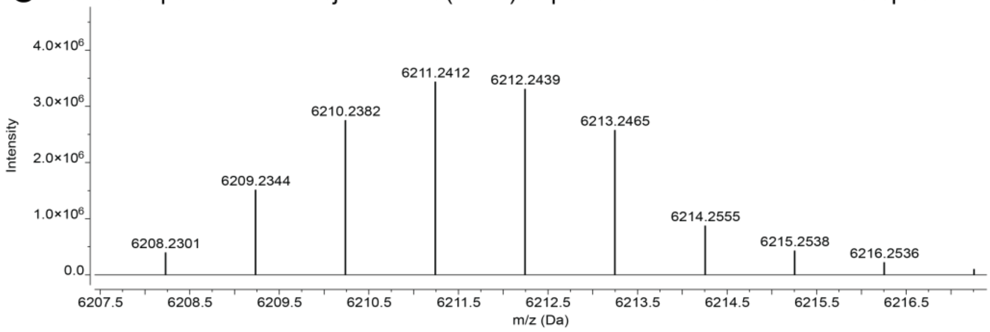

Fig. S24: **LC-MS analysis of Link-McjA with Ala(3-Thi) at position 12:** (A) Total ion chromatogram (TIC). (B) MS spectrum. (C) deconvoluted MS spectrum: Monoisotopic mass (ESI+): calc. [$C_{290}H_{444}N_{76}O_{74}S$]: 6207.3037, found: 6208.2301; Average mass calc. [$C_{290}H_{444}N_{76}O_{74}S$]: 6211.2600.

2.2.5 Link-McjA with 2-Thienyl-L-alanine = H’ (**13**)

Sequence: H_2N-IKHFHFNKLS SGKKNNVPSP AKGVIQIKKS ASQLTKGGAG HVPEYFVH’FG IPISFYG-OH

The peptide was prepared via Automated Fast-Flow Peptide Synthesis (AFPS) using NovaPEG HMPB resin (loading = 0.62 mmol/g, 82 mg, 51 μmol). The first amino acid was introduced as described in 1.3. The first amino acid was deprotected on the AFPS system. The synthesis using AFPS was performed following the general procedure in 1.2. until the point of His-12, where Fmoc-2-Thienyl-L-alanine-OH was incorporated following the general procedure 1.4. The amino acid was deprotected on the AFPS system and the synthesis using AFPS was continued. The total synthesis time was approximately 5.5 h.

Cleavage of the peptidyl-resin (26% of total resin) afforded the crude peptide (24% purity (C8) by UHPLC). The peptide was purified using semi-preparative HPLC as specified in the general procedure 1.9. Fractions were analyzed by LC-HR-ESI-MS, combined, and lyophilized to obtain 0.19 mg (0.2% yield; >95% purity (C8) and >95% purity (C18) by UHPLC) of the desired peptide.

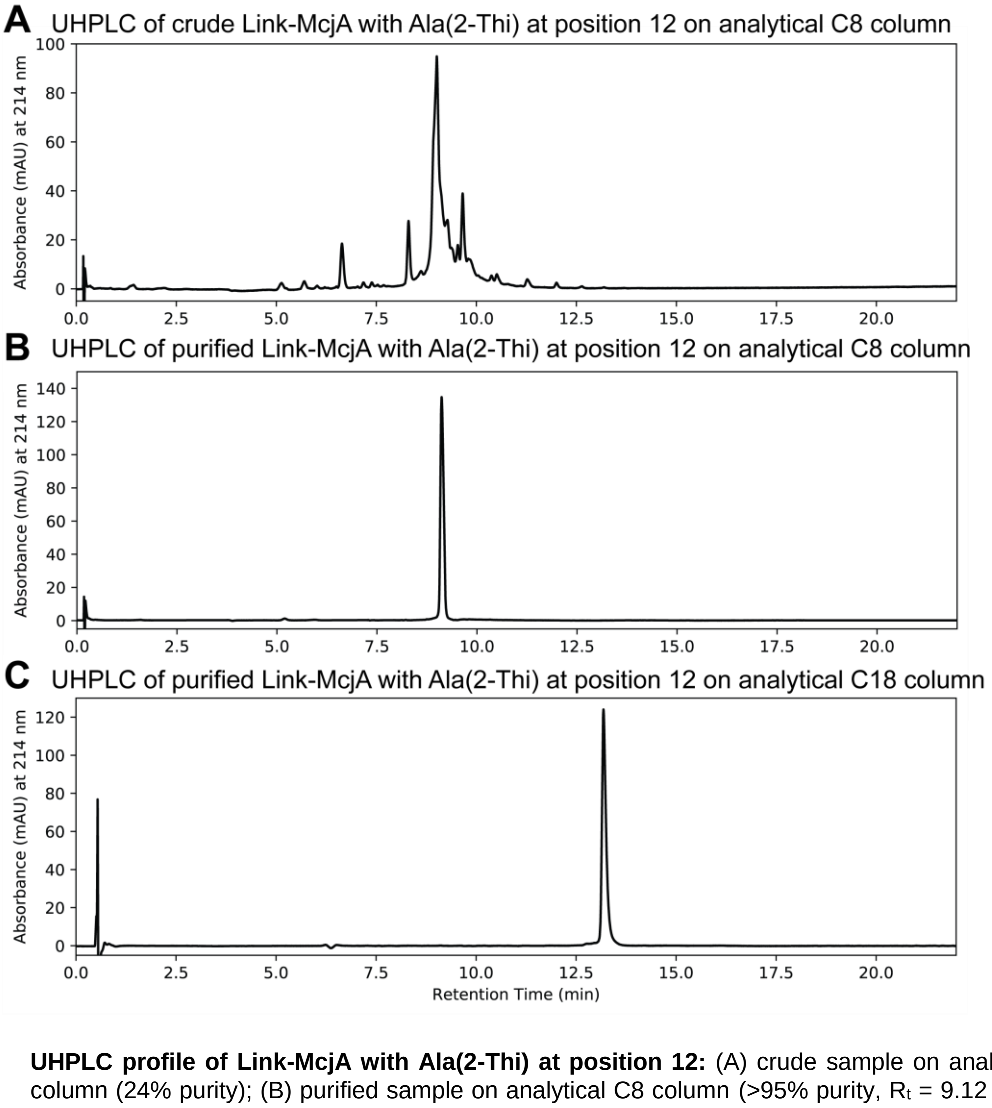

Fig. S25: UHPLC profile of Link-McjA with Ala(2-Thi) at position 12: (A) crude sample on analytical C8 column (24% purity); (B) purified sample on analytical C8 column (>95% purity, R_t = 9.12 min); (C) purified sample on analytical C18 column (>95% purity, R_t = 13.18 min).

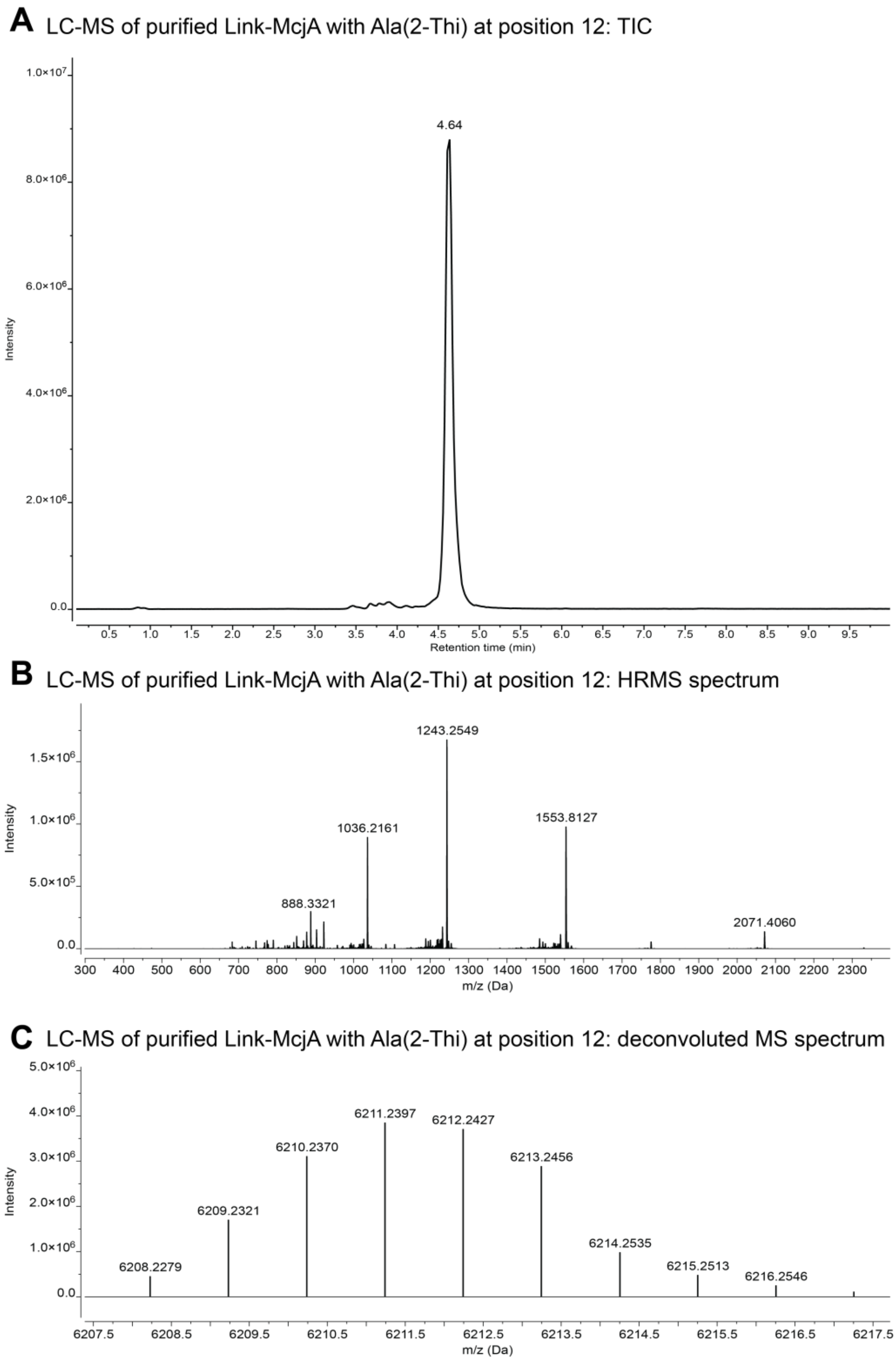

Fig. S26: LC-MS analysis of Link-McjA with Ala(2-Thi) at position 12: (A) Total ion chromatogram (TIC). (B) MS spectrum. (C) deconvoluted MS spectrum: Monoisotopic mass (ESI+): calc. [$C_{290}H_{444}N_{76}O_{74}S$]: 6207.3037, found: 6208.2279; Average mass calc. [$C_{290}H_{444}N_{76}O_{74}S$]: 6211.2600.

2.2.6 Link-McjA with 2-Furyl-L-alanine = H’ (**14**)

Sequence: H_2N-IKHFHFNKLS SGKKNNVPSP AKGVIQIKKS ASQLTKGGAG HVPEYFVH’FG IPISFYG-OH

The peptide was prepared via Automated Fast-Flow Peptide Synthesis (AFPS) using NovaPEG HMPB resin (loading = 0.62 mmol/g, 82 mg, 51 µmol). The first amino acid was introduced as described in 1.3. The first amino acid was deprotected on the AFPS system. The synthesis using AFPS was performed following the general procedure in 1.2. until the point of His-12, where Fmoc-2-Furyl-L-alanine-OH was incorporated following the general procedure 1.4. The amino acid was deprotected on the AFPS system and the synthesis using AFPS was continued. The total synthesis time was approximately 5.5 h.

Cleavage of the peptidyl-resin (27% of total resin) afforded the crude peptide (26% purity (C8) by UHPLC). The peptide was purified using semi-preparative HPLC as specified in the general procedure 1.9. Fractions were analyzed by LC-HR-ESI-MS, combined, and lyophilized to obtain 0.19 mg (0.2% yield; >95% purity (C8) and >95% purity (C18) by UHPLC) of the desired peptide.

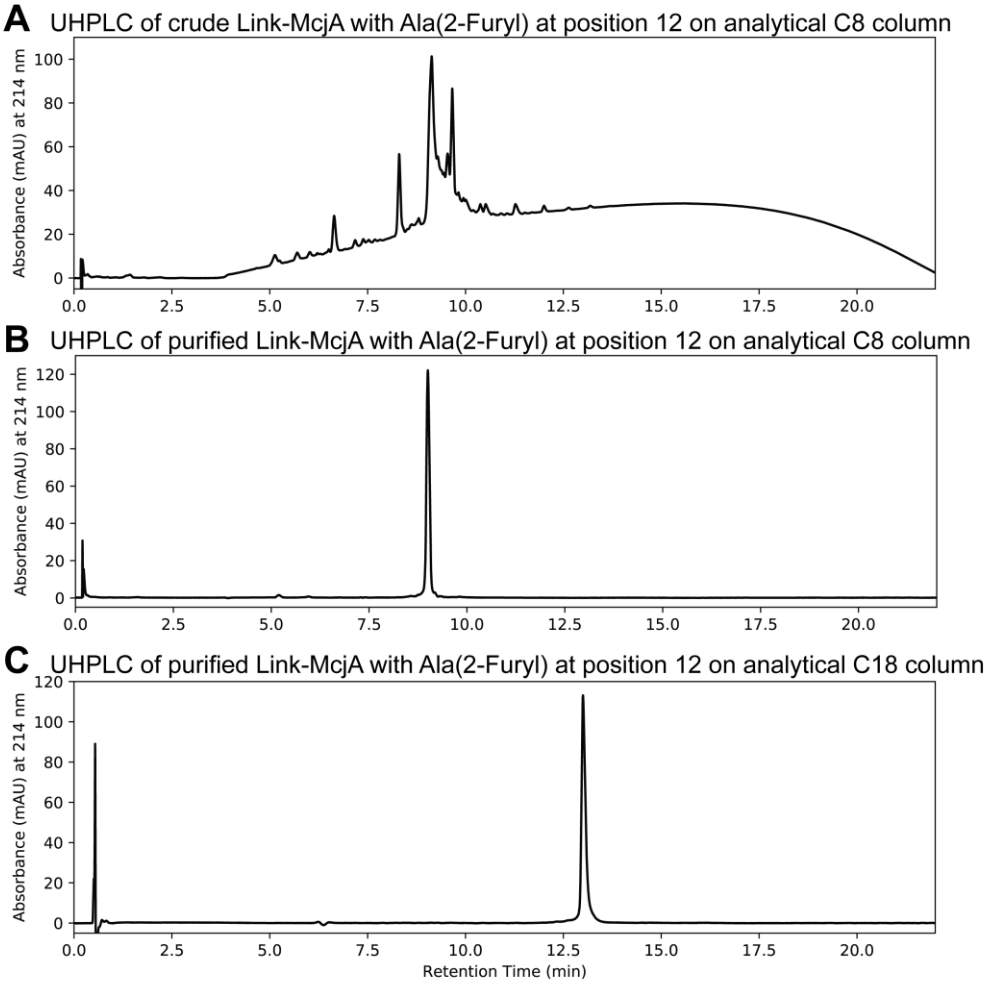

Fig. S27: UHPLC profile of Link-McjA with Ala(2-Furyl) at position 12: (A) crude sample on analytical C8 column (26% purity); (B) purified sample on analytical C8 column (>95% purity, R_t = 9.00 min); (C) purified sample on analytical C18 column (>95% purity, R_t = 13.00 min).

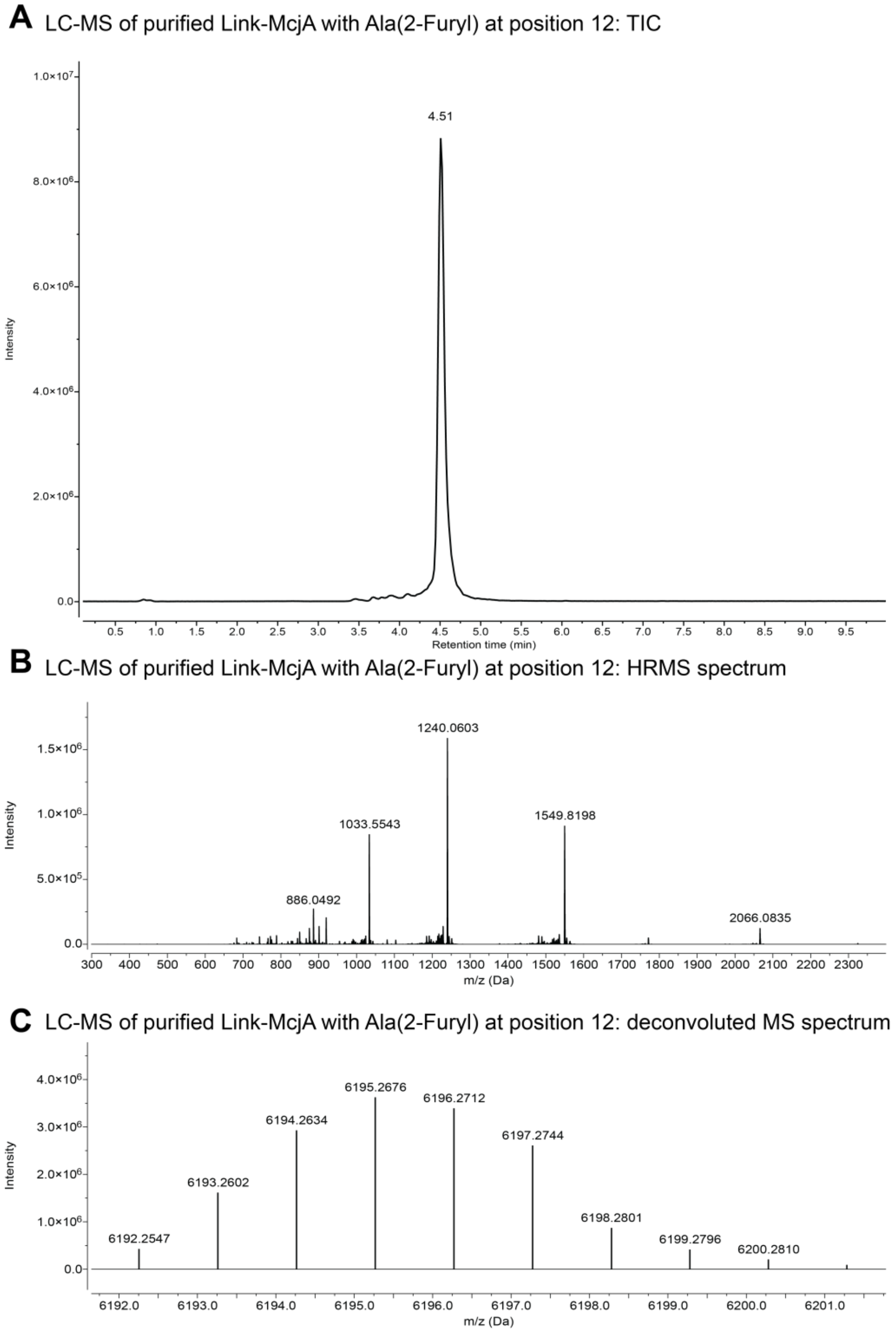

Fig. S28: **LC-MS analysis of Link-McjA with Ala(2-Furyl) at position 12:** (A) Total ion chromatogram (TIC). (B) MS spectrum. (C) deconvoluted MS spectrum: Monoisotopic mass (ESI+): calc. [$C_{290}H_{444}N_{76}O_{75}$]: 6191.3265, found: 6192.2547; Average mass calc. [$C_{290}H_{444}N_{76}O_{75}$]: 6195.1990.

2.2.7 Link-McjA with three D-amino acids (**15**)

Sequence: H_2N-IKHFHFNKLS SGKKNNVPSP AKGVIQIKKS ASQLTKGGAG HVPEYFVhfG iPISFYG-OH

The peptide was prepared via Automated Fast-Flow Peptide Synthesis (AFPS) using NovaPEG HMPB resin (loading = 0.62 mmol/g, 87 mg, 54 µmol). The first amino acid was introduced as described in 1.3. The first amino acid was deprotected on the AFPS system. The synthesis using AFPS was performed following the general procedure in 1.2., D-amino acids were incorporated in the same way as L-amino acids. The amino acid was deprotected on the AFPS system and the synthesis using AFPS was continued. The total synthesis time was approximately 3.5 h.

Cleavage of the peptidyl-resin (26% of total resin) afforded the crude peptide (27% purity (C8) by UHPLC). The peptide was purified using semi-preparative HPLC as specified in the general procedure 1.9. Fractions were analyzed by LC-HR-ESI-MS, combined, and lyophilized to obtain 0.61 mg (0.7% yield; >95% purity (C8) and >95% purity (C18) by UHPLC) of the desired peptide.

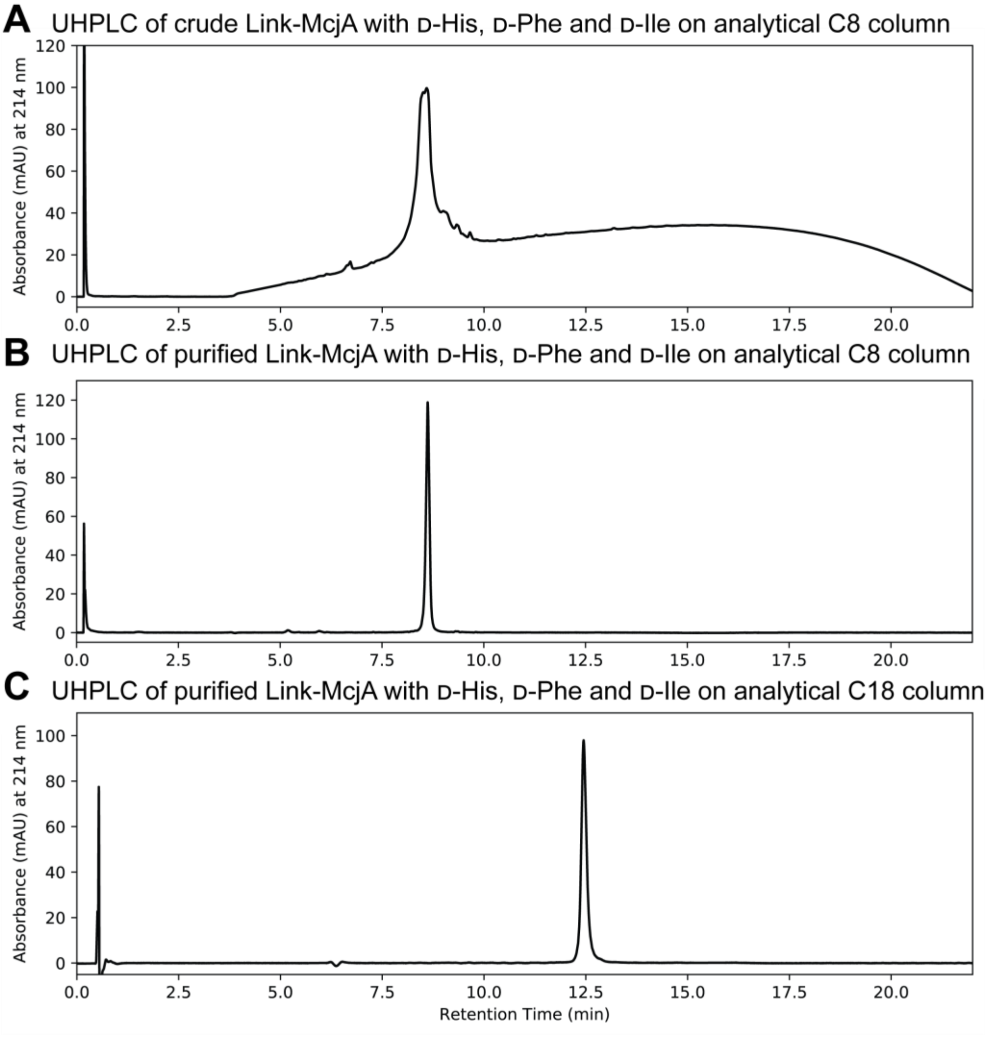

Fig. S29: UHPLC profile of Link-McjA with D-His-12, D-Phe-13 and D-Ile-15: (A) crude sample on analytical C8 column (27% purity); (B) purified sample on analytical C8 column (>95% purity, R_t = 8.62 min); (C) purified sample on analytical C18 column (>95% purity, R_t = 12.45 min).

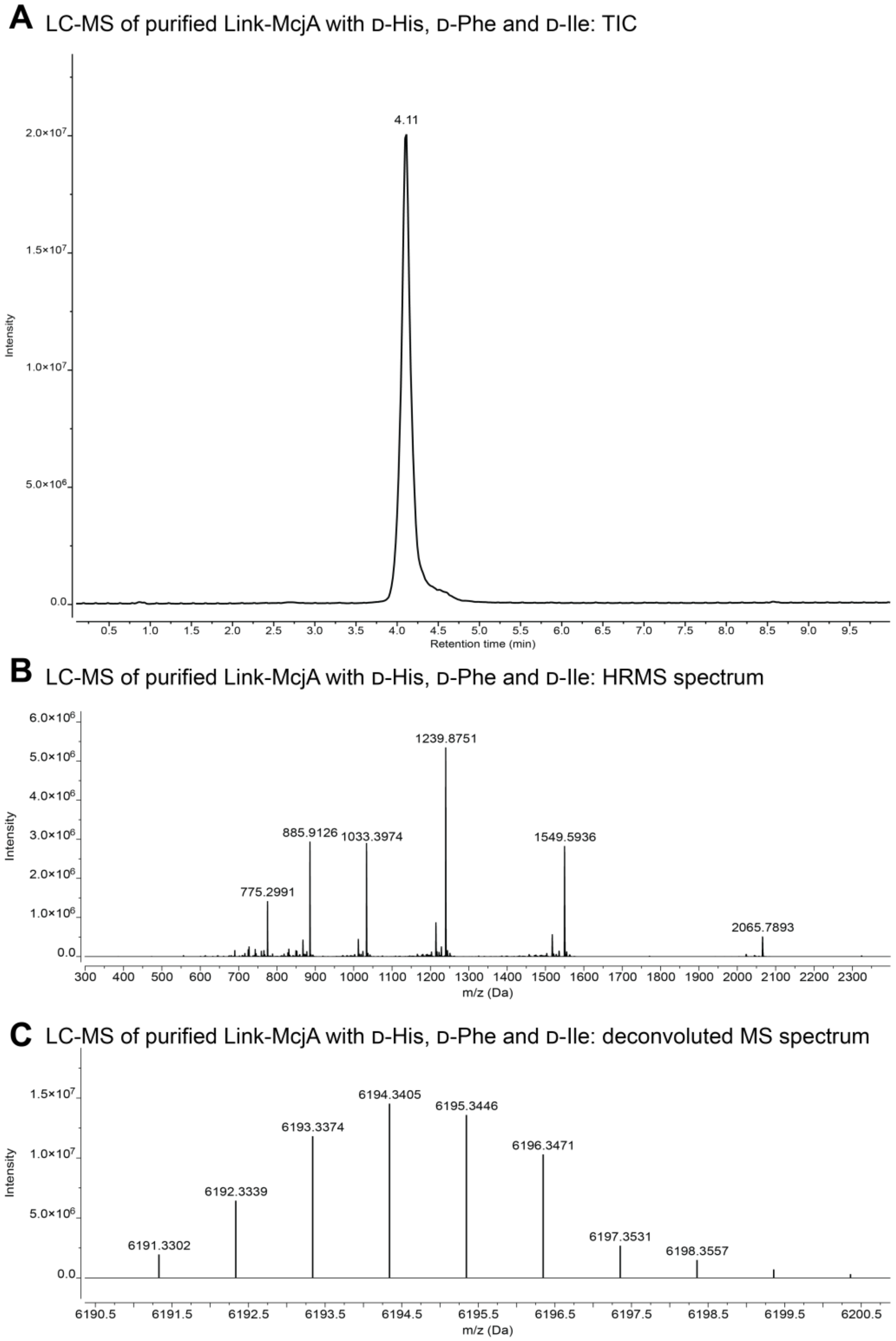

Fig. S30: LC-MS analysis of Link-McjA with D-His-12, D-Phe-13 and D-Ile-15: (A) Total ion chromatogram (TIC). (B) MS spectrum. (C) deconvoluted MS spectrum: Monoisotopic mass (ESI+): calc. [$C_{289}H_{444}N_{78}O_{74}$]: 6191.3378, found: 6191.3302; Average mass calc. [$C_{289}H_{444}N_{78}O_{74}$]: 6195.2030.

2.3 Synthesis of D-amino acid containing McjA-derivatives

2.3.1 Full-D-McjA

Sequence: `H2N-ikhfhfnkls sGkknnvpsp akGviqikks asqltkGGaG hvpeyfvGiG tpisfyG-OH`

The peptide was prepared via Automated Fast-Flow Peptide Synthesis (AFPS) using NovaPEG HMPB resin (loading = 0.62 mmol/g, 84 mg, 52 μmol). The first amino acid was introduced as described in 1.3. The first amino acid was deprotected on the AFPS system. The synthesis using AFPS was performed following the general procedure in 1.2. The total synthesis time was approximately 3.5 h

Cleavage of the peptidyl-resin (26% of total resin) afforded the crude peptide (18% purity (C8) by UHPLC). The peptide was purified using semi-preparative HPLC as specified in the general procedure 1.9. Fractions were analyzed by LC-HR-ESI-MS, combined, and lyophilized to obtain 0.89 mg (1.1% yield; 91% purity (C8) and >95% purity (C18) by UHPLC) of the desired peptide.

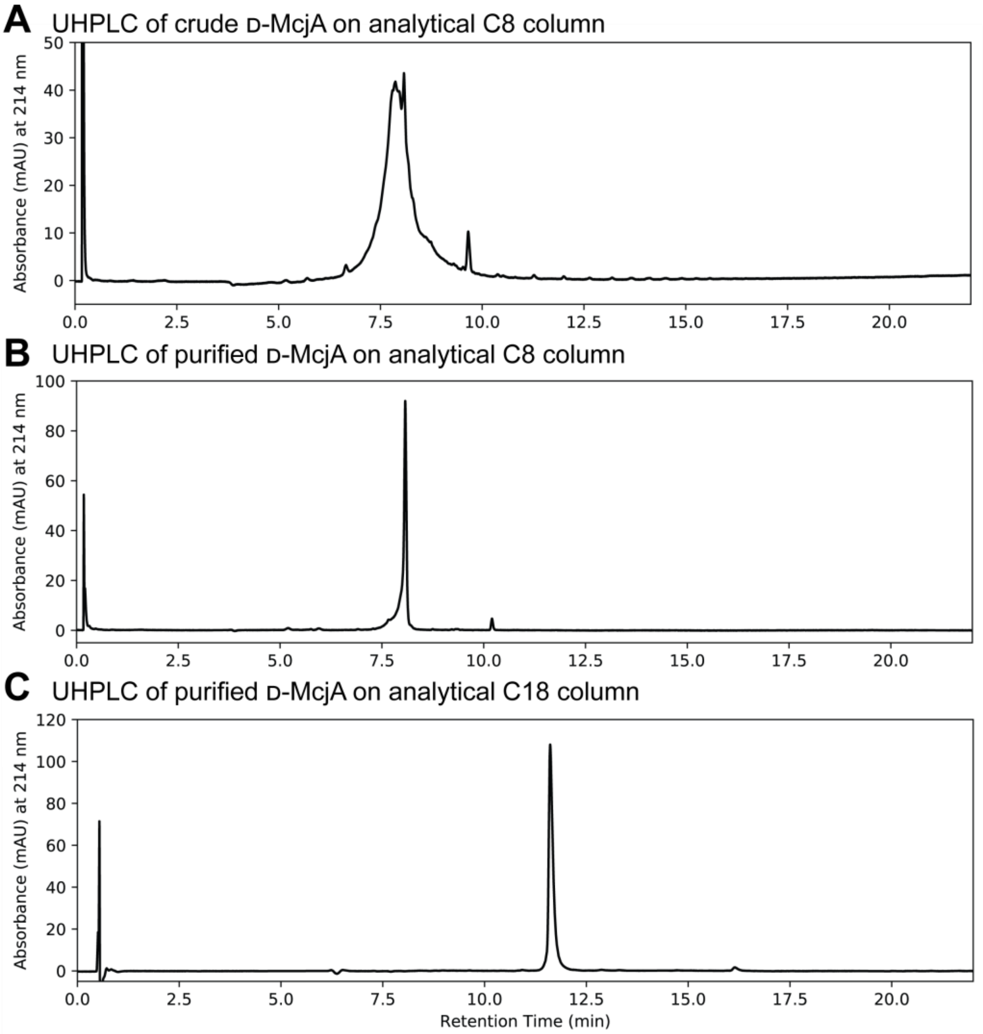

Fig. S31: **UHPLC profile of D-McjA:** (A) crude sample on analytical C8 column (18% purity); (B) purified sample on analytical C8 column (91% purity, R_t = 8.07 min); (C) purified sample on analytical C18 column (>95% purity, R_t = 11.61 min).

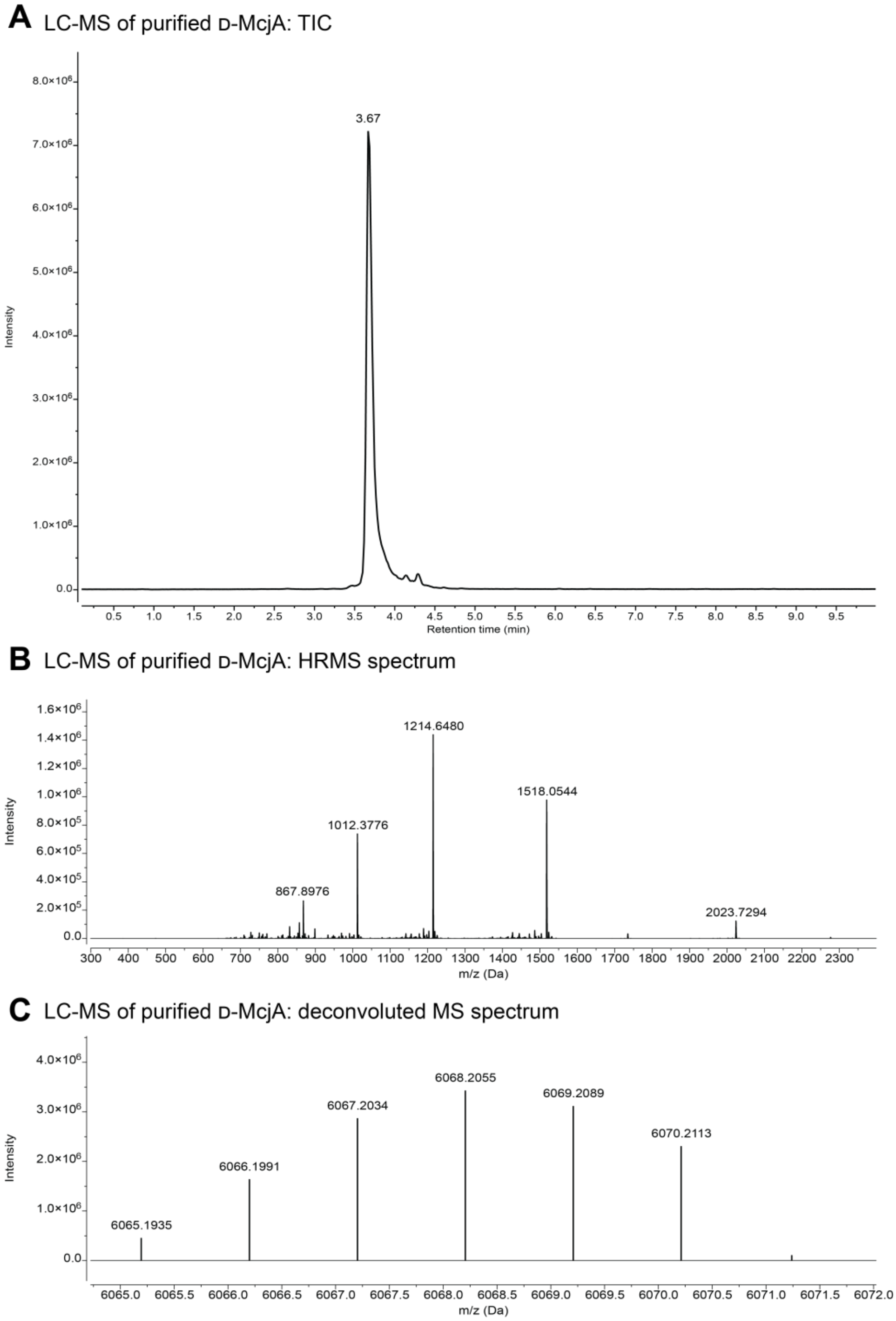

Fig. S32: **LC-MS analysis of D-McjA:** (A) Total ion chromatogram (TIC). (B) MS spectrum. (C) deconvoluted MS spectrum: Monoisotopic mass (ESI+): calc. [$C_{280}H_{438}N_{76}O_{75}$]: 6065.2796, found: 6065.1935; Average mass calc. [$C_{280}H_{438}N_{76}O_{75}$]: 6069.0410.

2.3.2 Leader-L- and core-D-McjA

Sequence: H_2N-ikhfhfnkls sGkknnvpsp akGviqikks asqltkGGAG HVPEYFVGIG TPISFYG-OH

The peptide was prepared via Automated Fast-Flow Peptide Synthesis (AFPS) using NovaPEG HMPB resin (loading = 0.62 mmol/g, 84 mg, 52 µmol). The first amino acid was introduced as described in 1.3. The first amino acid was deprotected on the AFPS system. The synthesis using AFPS was performed following the general procedure in 1.2. The total synthesis time was approximately 3.5 h

Cleavage of the peptidyl-resin (28% of total resin) afforded the crude peptide (17% purity (C8) by UHPLC). The peptide was purified using semi-preparative HPLC as specified in the general procedure 1.9. Fractions were analyzed by LC-HR-ESI-MS, combined, and lyophilized to obtain 0.65 mg (0.7% yield; >95% purity (C8) and >95% purity (C18) by UHPLC) of the desired peptide.

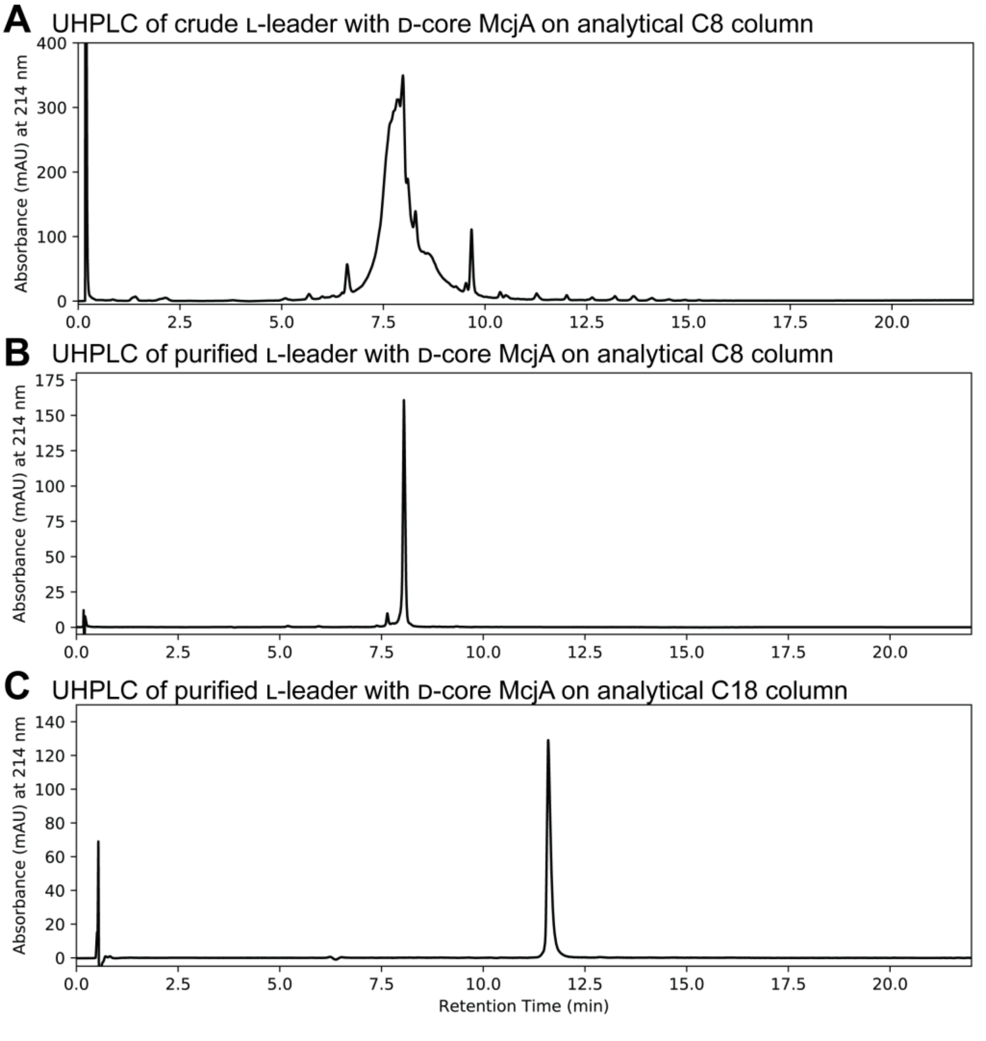

Fig. S33: UHPLC profile of McjA with L-leader and D-core peptide: (A) crude sample on analytical C8 column (17% purity); (B) purified sample on analytical C8 column (>95% purity, R_t = 8.05 min); (C) purified sample on analytical C18 column (>95% purity, R_t = 11.60 min).

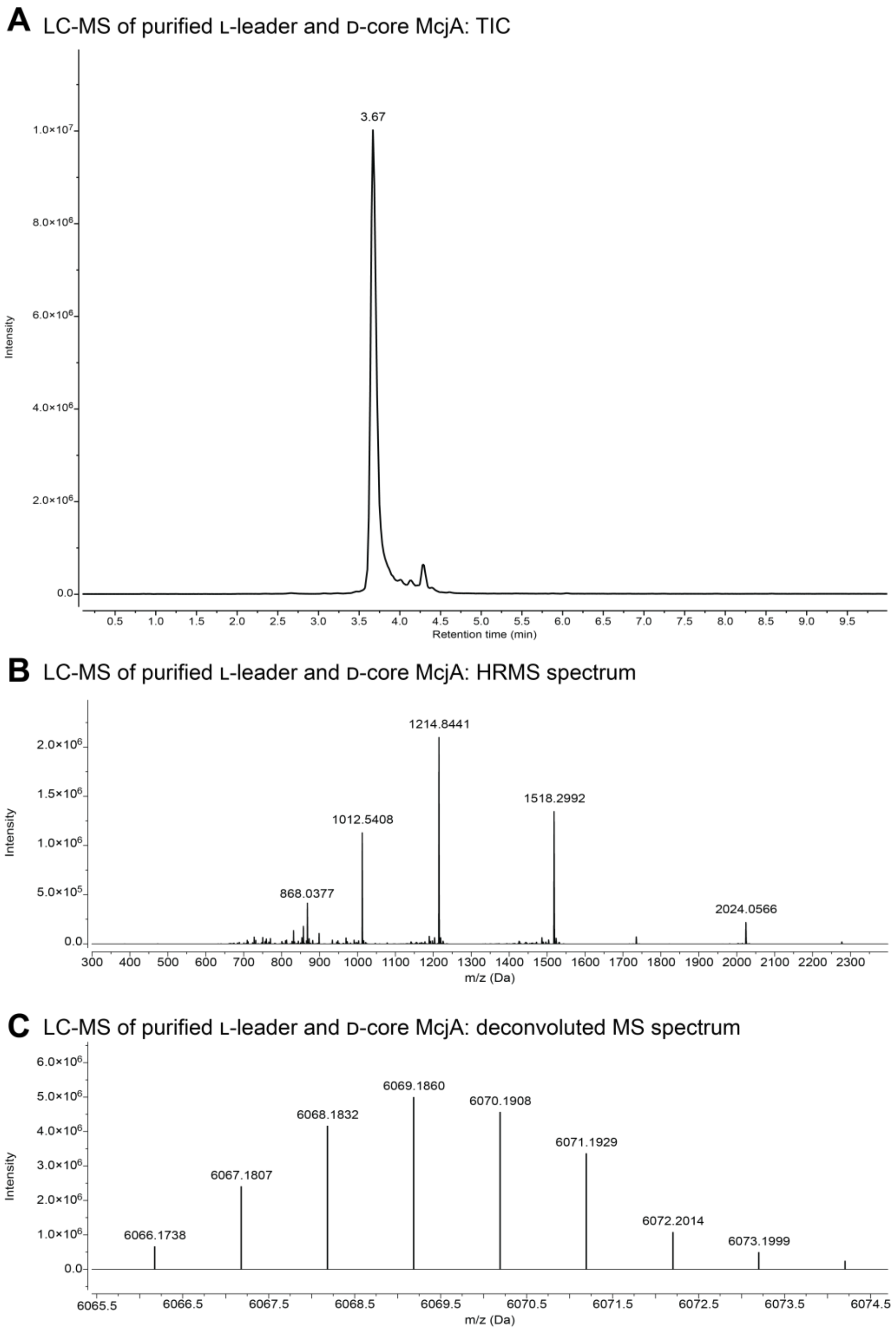

Fig. S34: LC-MS analysis of McjA with L-leader and D-core peptide: (A) Total ion chromatogram (TIC). (B) MS spectrum. (C) deconvoluted MS spectrum: Monoisotopic mass (ESI+): calc. [$C_{280}H_{438}N_{76}O_{75}$]: 6065.2796, found: 6066.1738; Average mass calc. [$C_{280}H_{438}N_{76}O_{75}$]: 6069.0410.

2.4 Synthesis of Microcin Y (MccY) precursor peptides

2.4.1 WT-McyA

Sequence: H_2N-FKKLFSSSKG HAVKKIPGVV RIQTPASQLT KGGRGHIAEY FSGPITQVSF YG-OH

The peptide was prepared via Automated Fast-Flow Peptide Synthesis (AFPS) using NovaPEG HMPB resin (loading = 0.62 mmol/g, 87 mg, 54 μmol). The first amino acid was introduced as described in 1.3. The first amino acid was deprotected on the AFPS system. The synthesis using AFPS was performed following the general procedure in 1.2. The total synthesis time was approximately 3.0 h

Cleavage of the peptidyl-resin (32% of total resin) afforded the crude peptide (21% purity (C8) by UHPLC). The peptide was purified using semi-preparative HPLC as specified in the general procedure 1.9. Fractions were analyzed by LC-HR-ESI-MS, combined, and lyophilized to obtain 1.5 mg (1.5% yield; >95% purity (C8) and >95% purity (C18) by UHPLC) of the desired peptide.

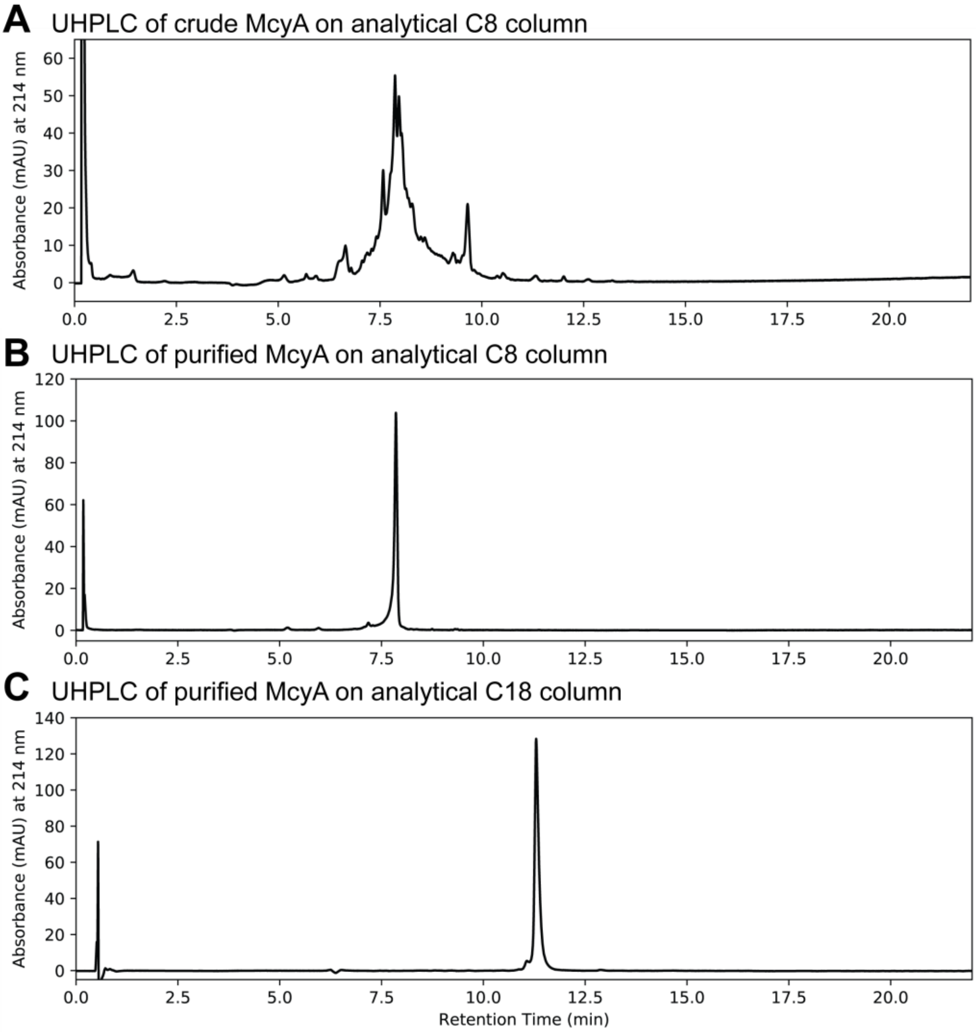

Fig. S35: UHPLC profile of McyA: (A) crude sample on analytical C8 column (21% purity); (B) purified sample on analytical C8 column (>95% purity, R_t = 7.85 min); (C) purified sample on analytical C18 column (>95% purity, R_t = 11.30 min).

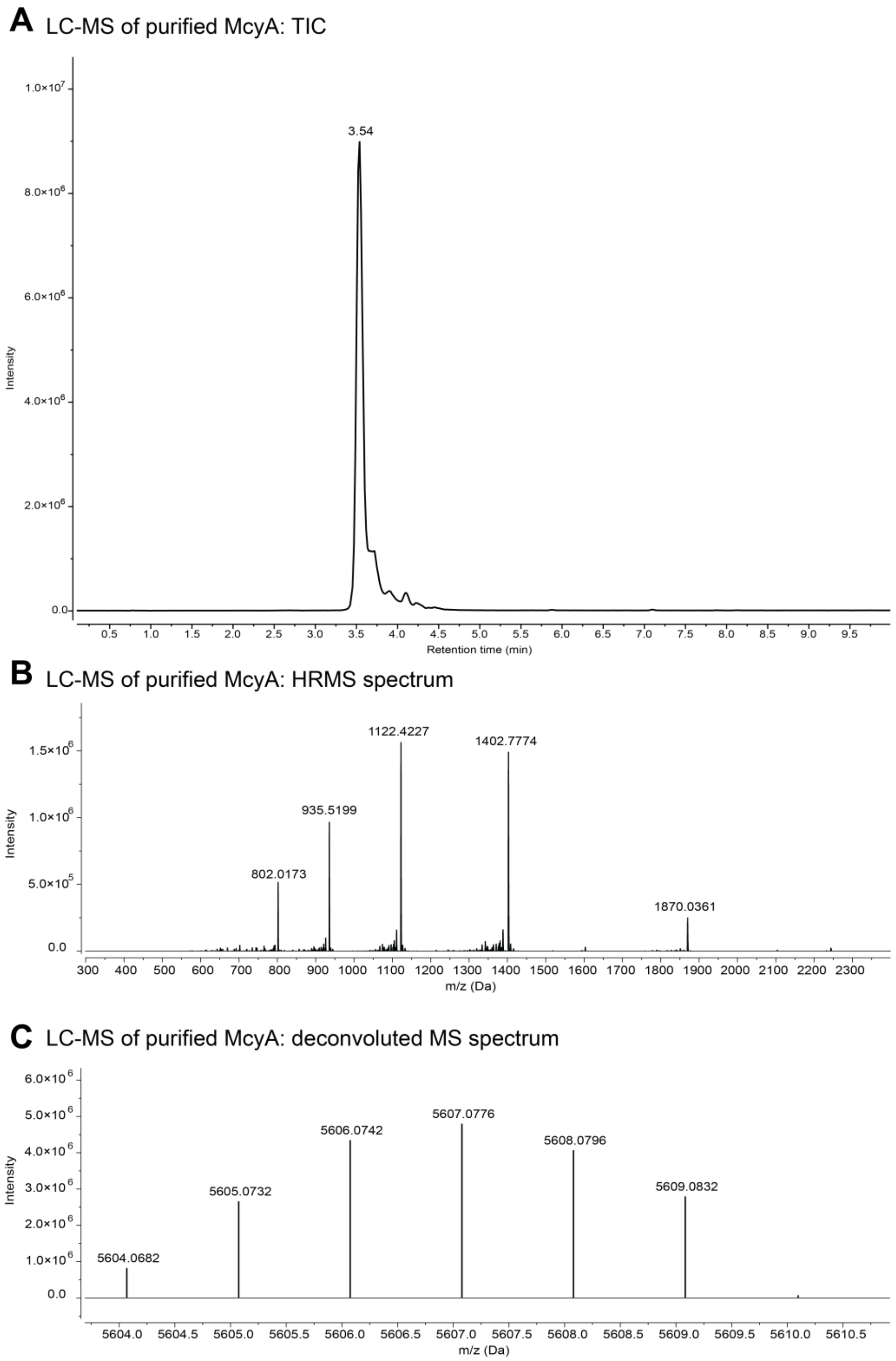

Fig. S36: **LC-MS analysis of McyA:** (A) Total ion chromatogram (TIC). (B) MS spectrum. (C) deconvoluted MS spectrum: Monoisotopic mass (ESI+): calc. [$C_{258}H_{407}N_{71}O_{69}$]: 5604.0522, found: 5604.0682; Average mass calc. [$C_{258}H_{407}N_{71}O_{69}$]: 5607.5220.

2.4.2 McjA-leader with McyA-core

Sequence: H_2N-IKHFHFNKLS SGKKNNVPSP AKGVIQIKKS ASQLTKGGRG HIAEYFSGPI TQVSFYG-OH

The peptide was prepared via Automated Fast-Flow Peptide Synthesis (AFPS) using NovaPEG HMPB resin (loading = 0.62 mmol/g, 87 mg, 54 μmol). The first amino acid was introduced as described in 1.3. The first amino acid was deprotected on the AFPS system. The synthesis using AFPS was performed following the general procedure in 1.2. The total synthesis time was approximately 3.5 h

Cleavage of the peptidyl-resin (29% of total resin) afforded the crude peptide (12% purity (C8) by UHPLC). The peptide was purified using semi-preparative HPLC as specified in the general procedure 1.9. Fractions were analyzed by LC-HR-ESI-MS, combined, and lyophilized to obtain 0.81 mg (0.8% yield; 90% purity (C8) and 94% purity (C18) by UHPLC) of the desired peptide.

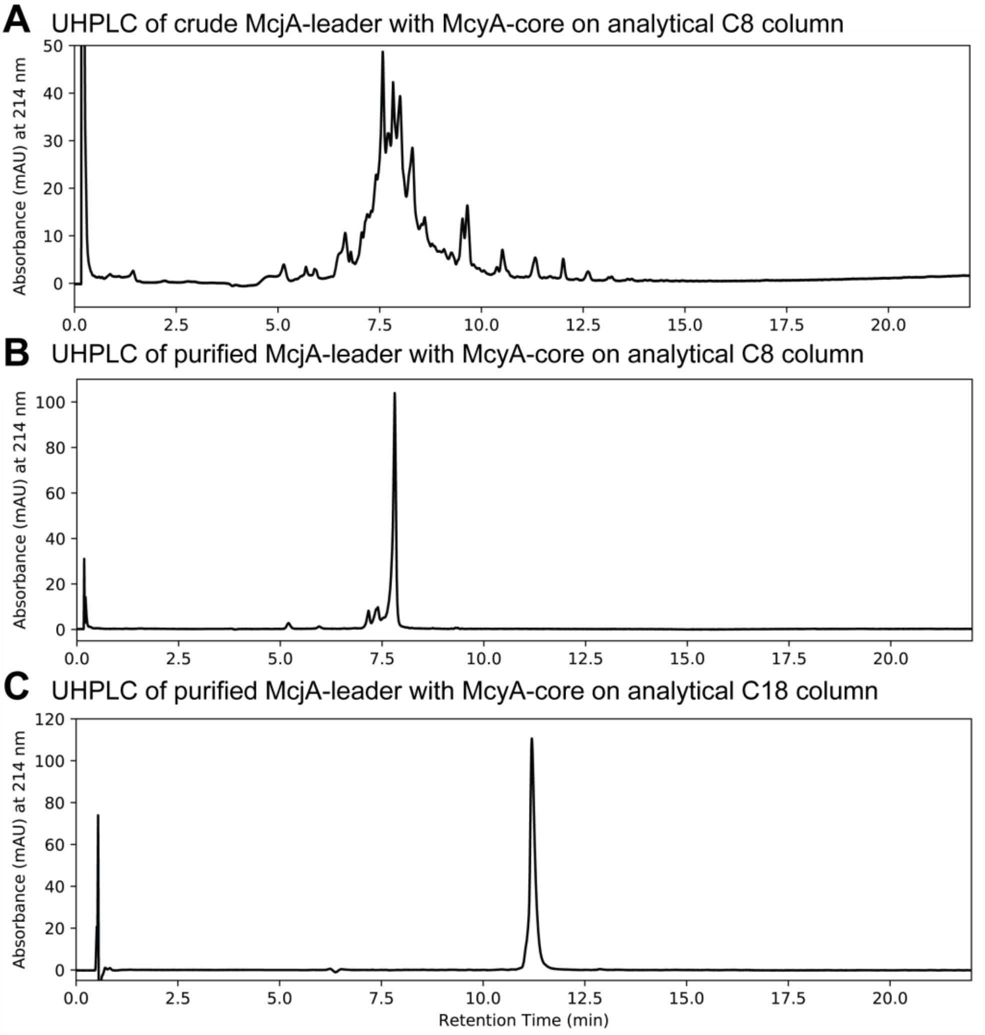

Fig. S37: UHPLC profile of McjA-leader with McyA-core peptide: (A) crude sample on analytical C8 column (12% purity); (B) purified sample on analytical C8 column (90% purity, R_t = 7.81 min); (C) purified sample on analytical C18 column (94% purity, R_t = 11.12 min).

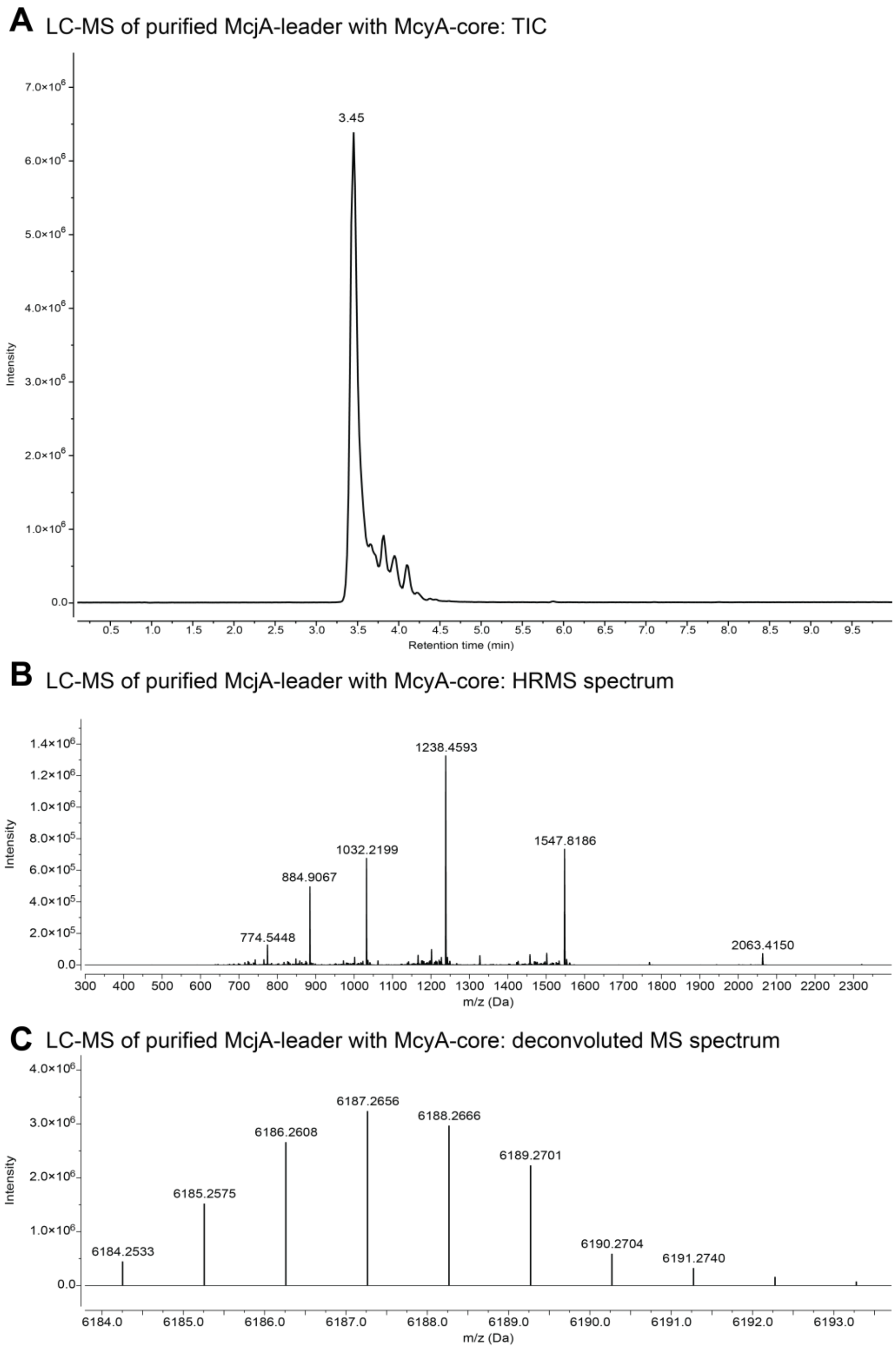

Fig. S38: LC-MS analysis of McjA-leader with McyA-core peptide: (A) Total ion chromatogram (TIC). (B) MS spectrum. (C) deconvoluted MS spectrum: Monoisotopic mass (ESI+): calc. [$C_{282}H_{444}N_{80}O_{77}$]: 6183.3287, found: 6184.2533; Average mass calc. [$C_{282}H_{444}N_{80}O_{77}$]: 6187.1370.

2.4.3 McyA-leader with McjA-core

Sequence: `H2N-FKKLFSSSKG HAVKKIPGVV RIQTPASQLT KGGAGHVPEY FVGIGTPISF YG-OH`

The peptide was prepared via Automated Fast-Flow Peptide Synthesis (AFPS) using NovaPEG HMPB resin (loading = 0.62 mmol/g, 88 mg, 55 μmol). The first amino acid was introduced as described in 1.3. The first amino acid was deprotected on the AFPS system. The synthesis using AFPS was performed following the general procedure in 1.2. The total synthesis time was approximately 3.5 h

Cleavage of the peptidyl-resin (35% of total resin) afforded the crude peptide (25% purity (C8) by UHPLC). The peptide was purified using semi-preparative HPLC as specified in the general procedure 1.9. Fractions were analyzed by LC-HR-ESI-MS, combined, and lyophilized to obtain 1.1 mg (1.0% yield; >95% purity (C8) and >95% purity (C18) by UHPLC) of the desired peptide.

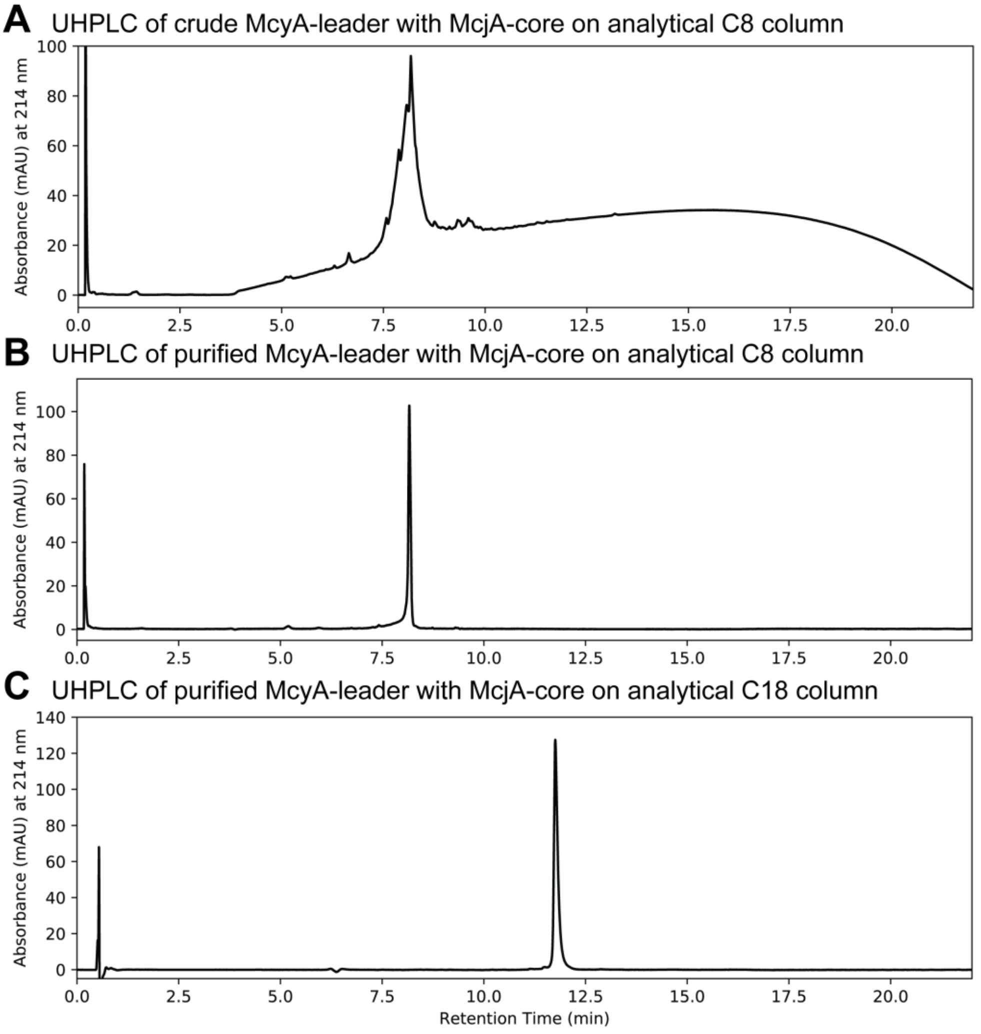

Fig. S39: UHPLC profile of McyA-leader with McjA-core peptide: (A) crude sample on analytical C8 column (25% purity); (B) purified sample on analytical C8 column (>95% purity, R_t = 8.17 min); (C) purified sample on analytical C18 column (>95% purity, R_t = 11.76 min).

A LC-MS of purified McyA-leader with McjA-core: TIC

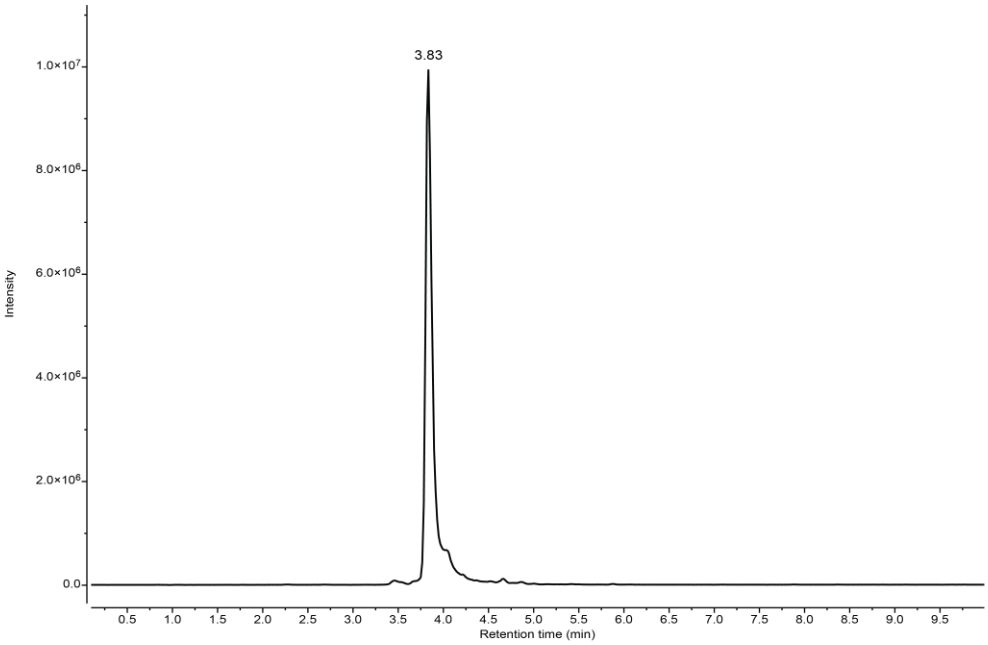

B LC-MS of purified McyA-leader with McjA-core: HRMS spectrum

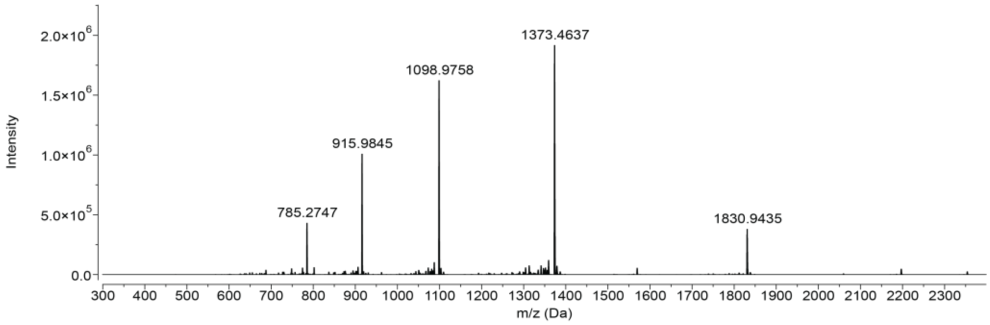

C LC-MS of purified McyA-leader with McjA-core: deconvoluted MS spectrum

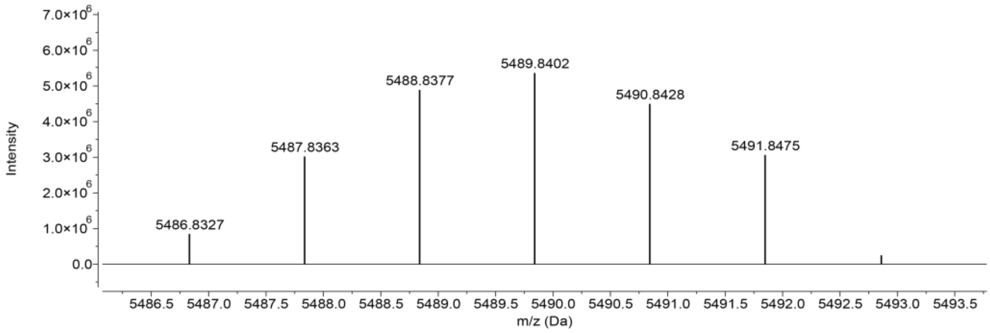

Fig. S40: LC-MS analysis of McyA-leader with McjA-core peptide: (A) Total ion chromatogram (TIC). (B) MS spectrum. (C) deconvoluted MS spectrum: Monoisotopic mass (ESI+): calc. [$C_{256}H_{401}N_{67}O_{67}$]: 5486.0031, found: 5486.8327; Average mass calc. [$C_{256}H_{401}N_{67}O_{67}$]: 5489.4260.

2.5 Synthesis of backbone *N*-methylated-derivatives of McjA

2.5.1 McjA with *N*-methylation at Gly12 (**16**)

Sequence: H_2N-IKHFHFNKLS SGKKNNVPSP AKGVIQIKKS ASQLTKGGAG HVPEYFVGIG TPISFYG-OH

The peptide was prepared via Automated Fast-Flow Peptide Synthesis (AFPS) using TentaGel XV HMPA resin (loading = 0.29 mmol/g, 101 mg, 29 µmol). The first amino acid was introduced as described in 1.3. The first amino acid was deprotected on the AFPS system. The synthesis using AFPS was performed following the general procedure in 1.2. Fmoc-Sar-OH was incorporated as Fmoc-Gly-OH using PyAOP as coupling reagent. The synthesis was performed using Fmoc-His(Boc)-OH. The total synthesis time was approximately 3.5 h

Cleavage of the peptidyl-resin (38% of total resin) afforded the crude peptide (53% purity (C8) by UHPLC). The peptide was purified using semi-preparative HPLC as specified in the general procedure 1.9. Fractions were analyzed by LC-HR-ESI-MS, combined, and lyophilized to obtain 5.4 mg (8% yield; >95% purity (C8) and >95% purity (C18) by UHPLC) of the desired peptide.

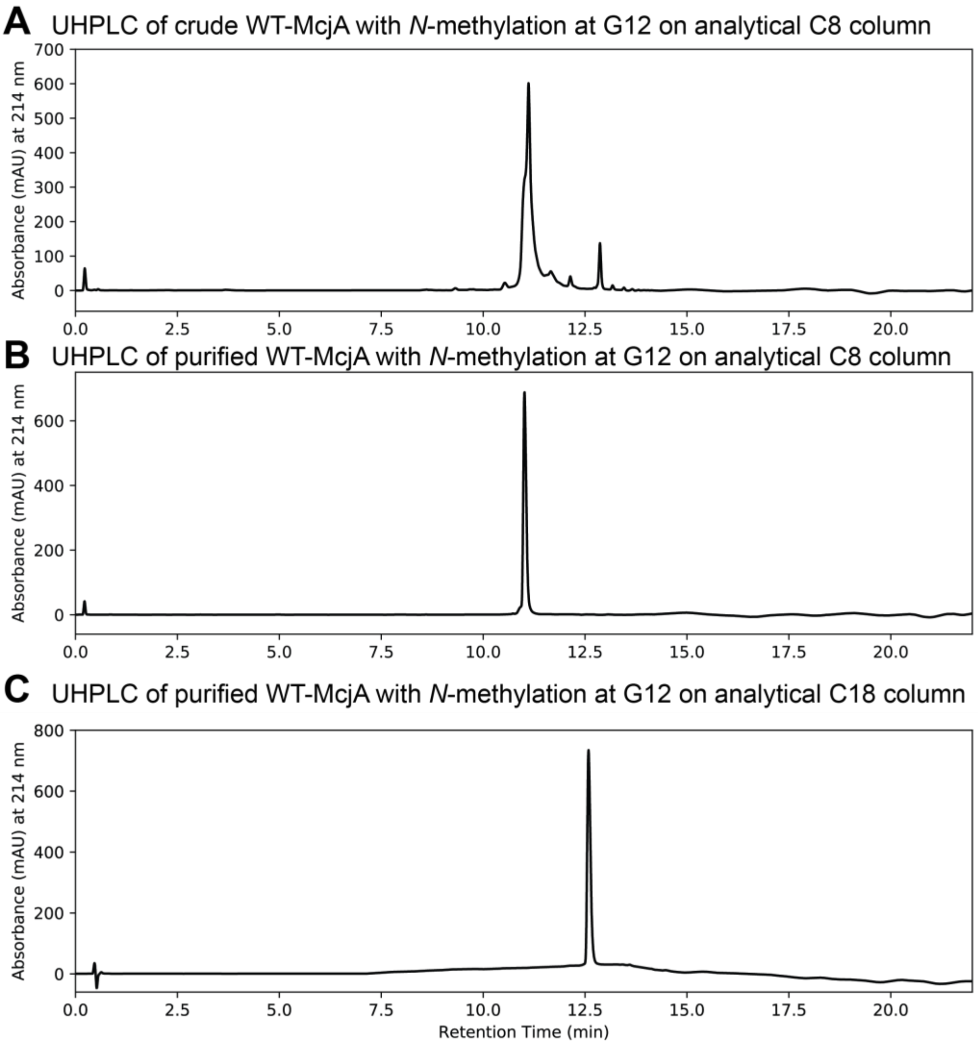

Fig. S41: **UHPLC profile of McjA with *N*-Me-Gly12:** (A) crude sample on analytical C8 column (53% purity); (B) purified sample on analytical C8 column (>95% purity, R_t = 11.01 min); (C) purified sample on analytical C18 column (>95% purity, R_t = 12.59 min).

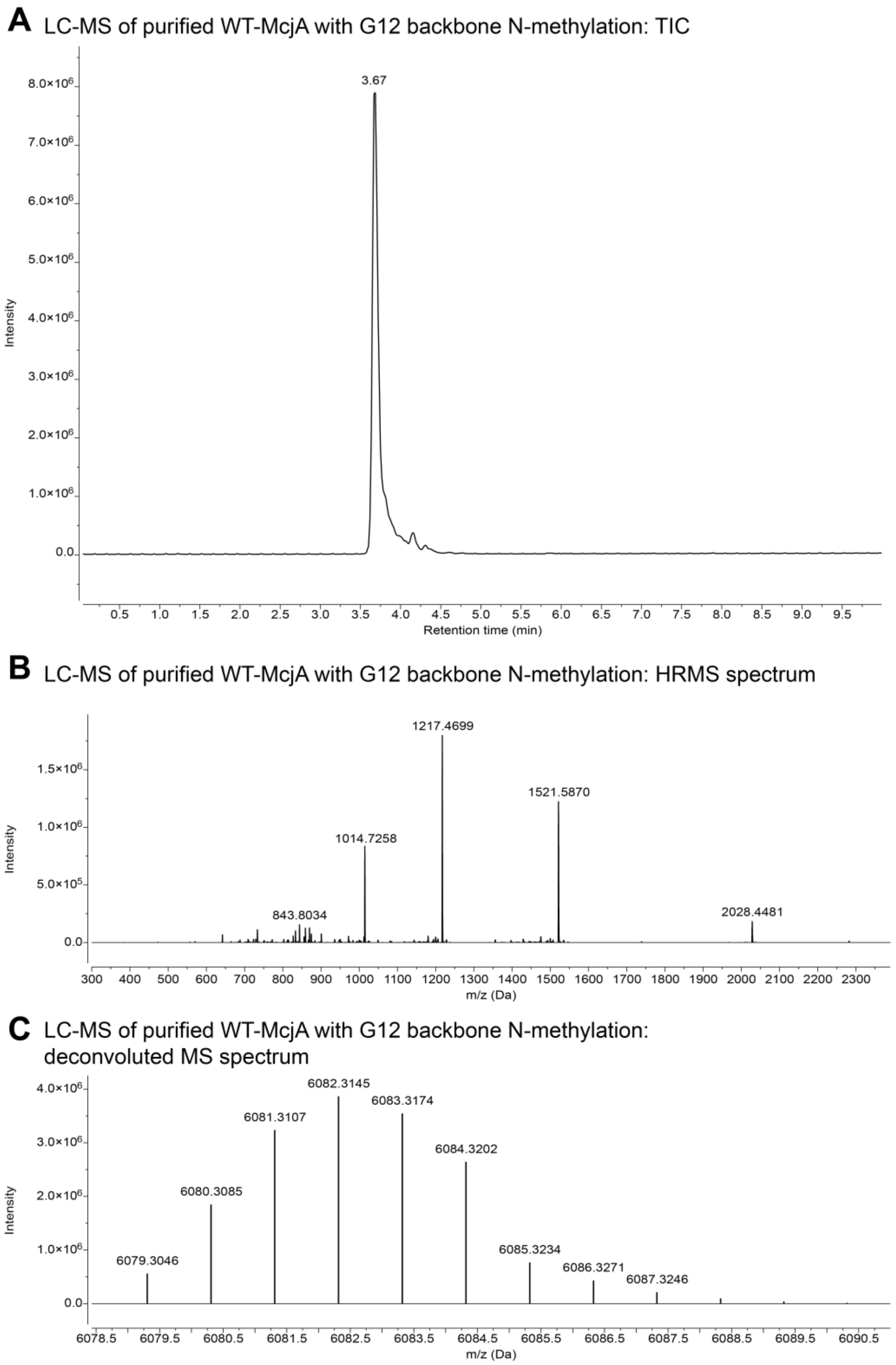

Fig. S42: **LC-MS analysis of McjA with *N*-Me-Gly12:** (A) Total ion chromatogram (TIC). (B) MS spectrum. (C) deconvoluted MS spectrum: Monoisotopic mass (ESI+): calc. [$C_{281}H_{440}N_{76}O_{75}$]: 6079.2952, found: 6079.3046; Average mass calc. [$C_{281}H_{440}N_{76}O_{75}$]: 6083.0680.

2.5.2 McjA with *N*-methylation at Gly12 and G14 (**17**)

Sequence: `H2N-IKHFHFNKLS SGKKNNVPSP AKGVIQIKKS ASQLTKGGAG HVPEYFVGIG TPISFYG-OH`

The peptide was prepared via Automated Fast-Flow Peptide Synthesis (AFPS) using TentaGel XV HMPA resin (loading = 0.29 mmol/g, 103 mg, 30 µmol). The first amino acid was introduced as described in 1.3. The first amino acid was deprotected on the AFPS system. The synthesis using AFPS was performed following the general procedure in 1.2. Fmoc-Sar-OH was incorporated as Fmoc-Gly-OH using PyAOP as coupling reagent. The Fmoc-Ile-OH after Fmoc-Sar-OH was coupled using PyAOP with more pump strokes (13 instead of 8) increasing thereby the equivalents. The synthesis was performed using Fmoc-His(Boc)-OH. The total synthesis time was approximately 3.5 h

Cleavage of the peptidyl-resin (44% of total resin) afforded the crude peptide (50% purity (C8) by UHPLC). The peptide was purified using semi-preparative HPLC as specified in the general procedure 1.9. Fractions were analyzed by LC-HR-ESI-MS, combined, and lyophilized to obtain 4.3 mg (5% yield; >95% purity (C8) and >95% purity (C18) by UHPLC) of the desired peptide.

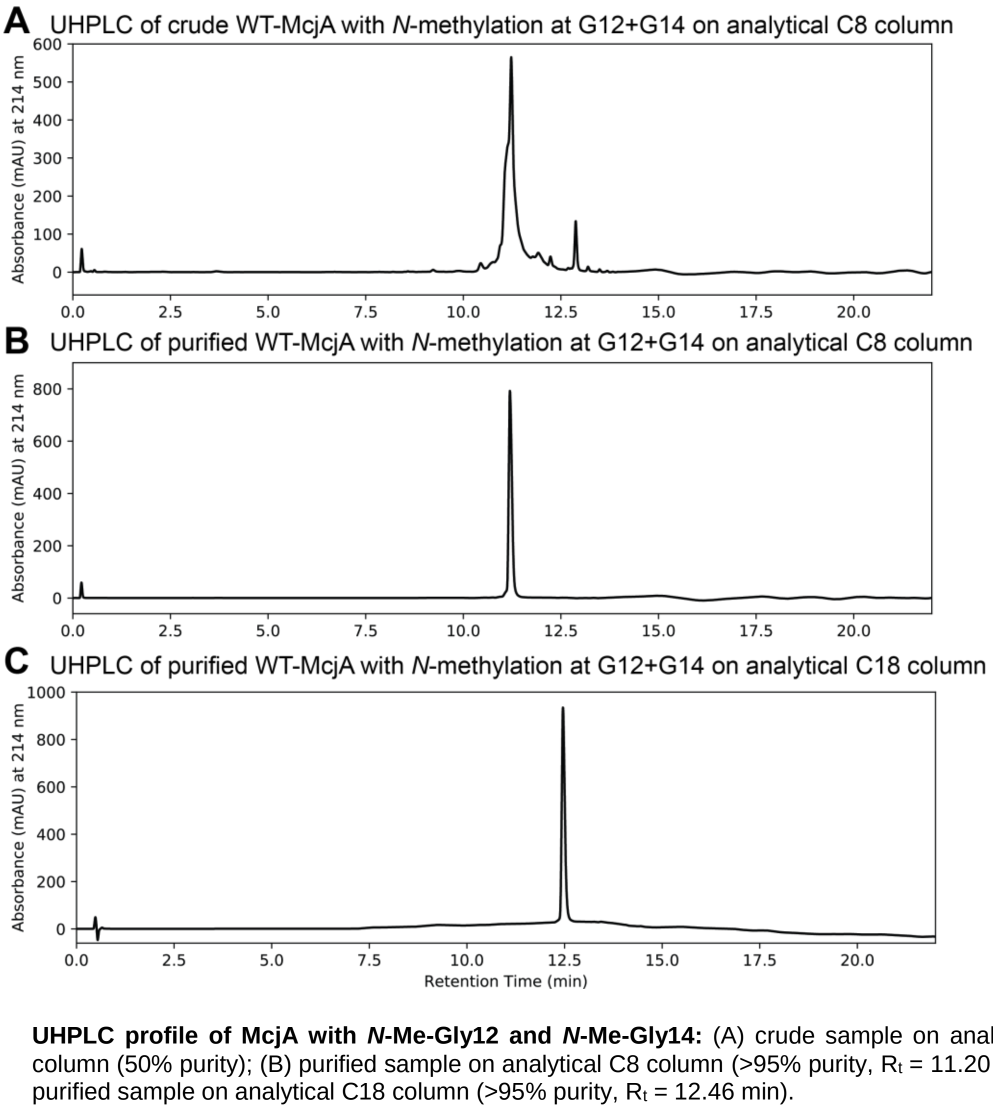

Fig. S43: UHPLC profile of McjA with *N*-Me-Gly12 and *N*-Me-Gly14: (A) crude sample on analytical C8 column (50% purity); (B) purified sample on analytical C8 column (>95% purity, R_t = 11.20 min); (C) purified sample on analytical C18 column (>95% purity, R_t = 12.46 min).

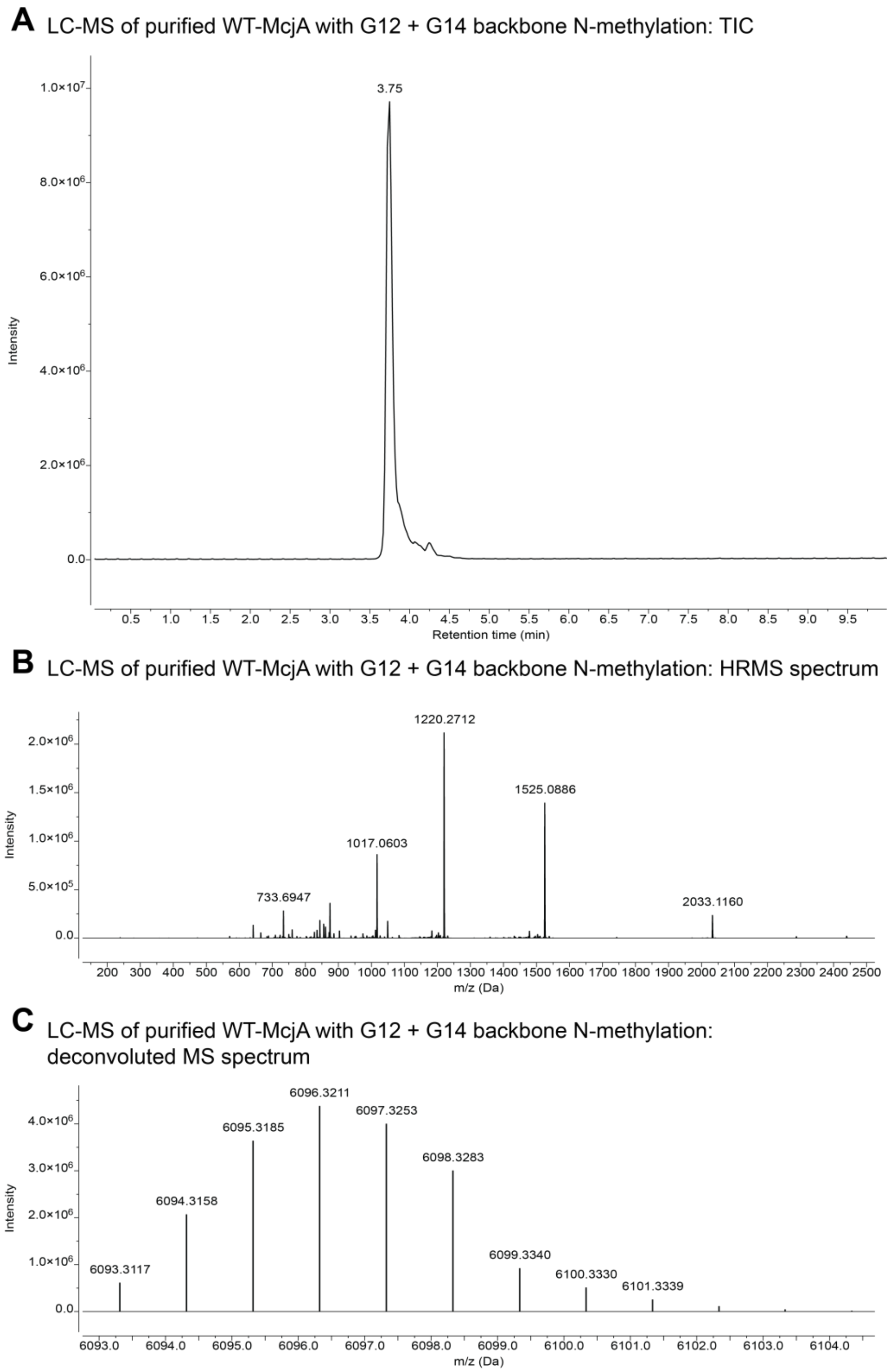

Fig. S44: **LC-MS analysis of McjA with *N*-Me-Gly12 and *N*-Me-Gly14:** (A) Total ion chromatogram (TIC). (B) MS spectrum. (C) deconvoluted MS spectrum: Monoisotopic mass (ESI+): calc. [$C_{282}H_{442}N_{76}O_{75}$]: 6093.3109, found: 6093.3117; Average mass calc. [$C_{282}H_{442}N_{76}O_{75}$]: 6097.0950.

2.6 Synthesis of branched-cyclic MccJ25 analogs

2.6.1 WT-branched-cyclic MccJ25 (**bc-1'**)

Sequence: H_2N-GGAGHVPEYF VGIGTPISFY G-OH

The peptide was prepared via the procedure described in 1.5 using NovaPEG HMPB resin (loading = 0.62 mmol/g, 79 mg, 49 μmol). One portion of the peptidyl-resin of the linear peptide (54% of total resin) was deprotected and cyclized. Cleavage of the peptidyl-resin afforded the crude peptide (50% purity (C18) by UHPLC). The peptide was purified using semi-preparative HPLC as specified in the general procedure 1.9. Fractions were analyzed by LC-HR-ESI-MS, combined and lyophilized to obtain 1.4 mg (3% yield; >95% purity (C18) and 92% purity (C8) by UHPLC) of the desired peptide.

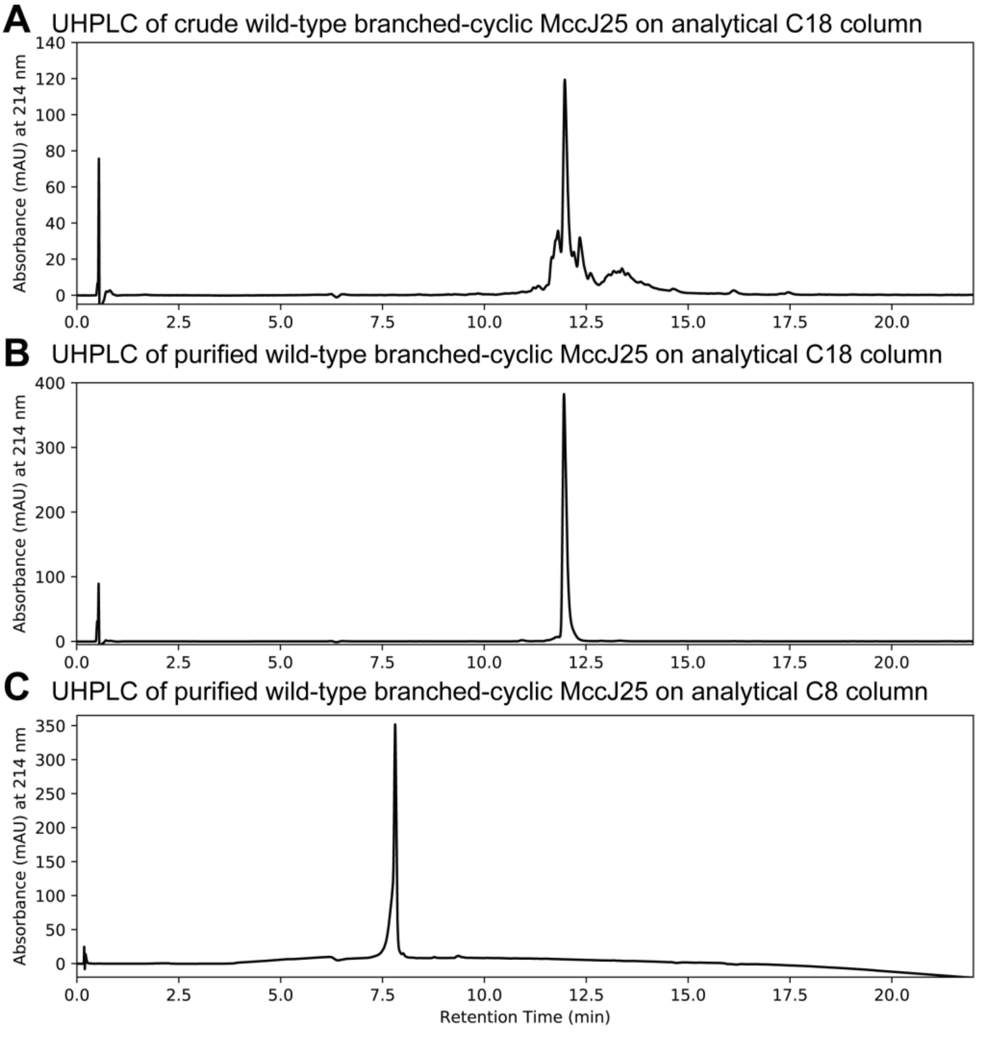

Fig. S45: UHPLC profile of wild-type branched-cyclic MccJ25: (A) crude sample on analytical C18 column (50% purity); (B) purified sample on analytical C18 column (>95% purity, R_t = 11.96 min); (C) purified sample on analytical C8 column (>92% purity, R_t = 7.81 min).

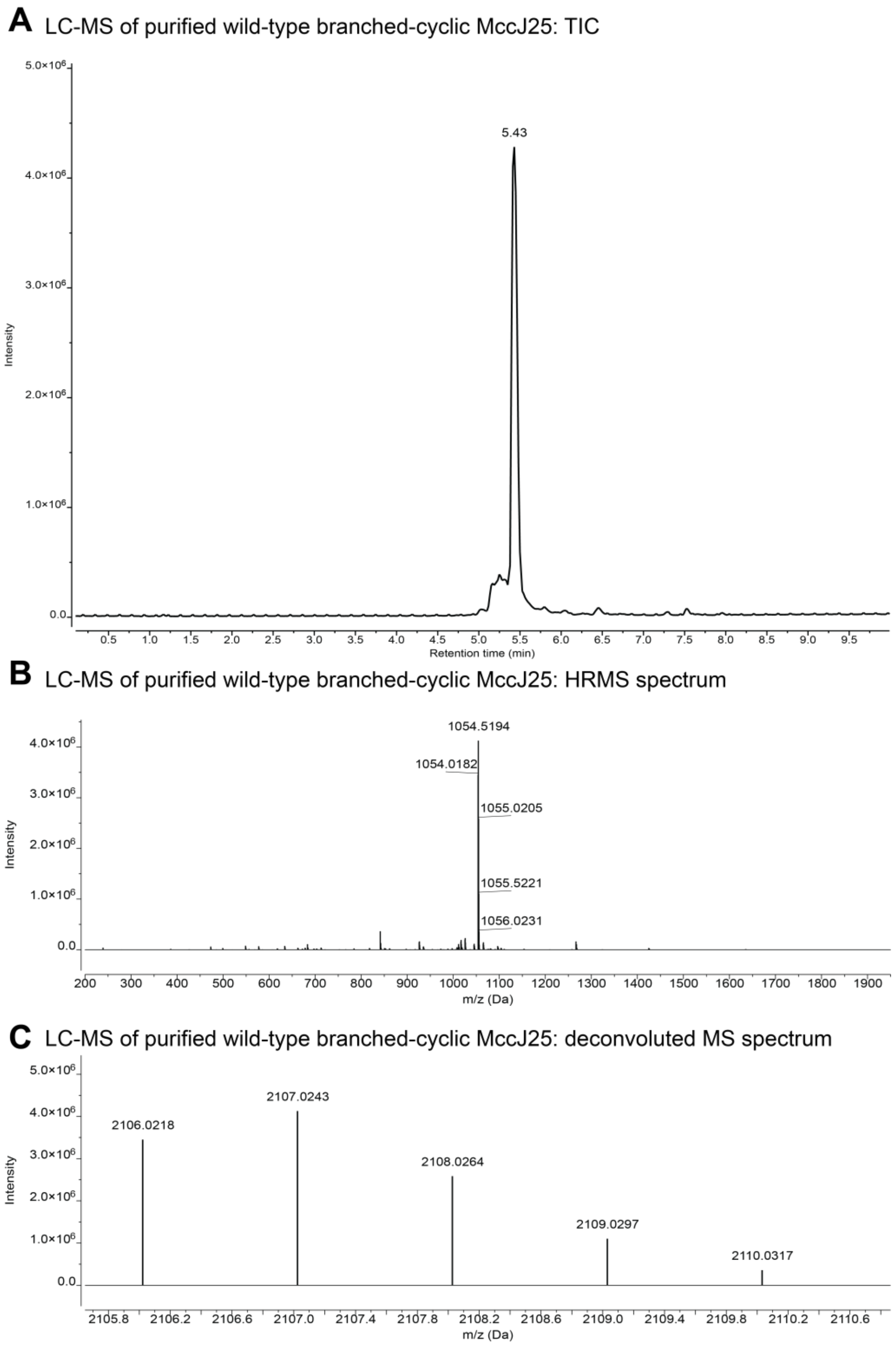

Fig. S46: LC-MS analysis of wild-type branched-cyclic MccJ25: (A) Total ion chromatogram (TIC). (B) MS spectrum. (C) deconvoluted MS spectrum: Monoisotopic mass (ESI+): calc. [$C_{101}H_{139}N_{23}O_{27}$]: 2106.0211, found: 2106.0218; Average mass calc. [$C_{101}H_{139}N_{23}O_{27}$]: 2107.3570.

2.6.2 Branched-cyclic MccJ25 with Phe(4-NH$_2$) = Y' (**bc-5'**)

Sequence: H_2N-GGAGHVPEY'F VGIGTPISFY G-OH

The peptide was prepared via the procedure described in 1.5 using NovaPEG HMPB resin (loading = 0.62 mmol/g, 84 mg, 52 µmol). One portion of the peptidyl-resin of the linear peptide (49% of total resin) was deprotected and cyclized. Cleavage of the peptidyl-resin afforded the crude peptide (46% purity (C18) by UHPLC). The peptide was purified using semi-preparative HPLC as specified in the general procedure 1.9. Fractions were analyzed by LC-HR-ESI-MS, combined and lyophilized to obtain 1.6 mg (3% yield; >95% purity (C18) and >95% purity (C8) by UHPLC) of the desired peptide.

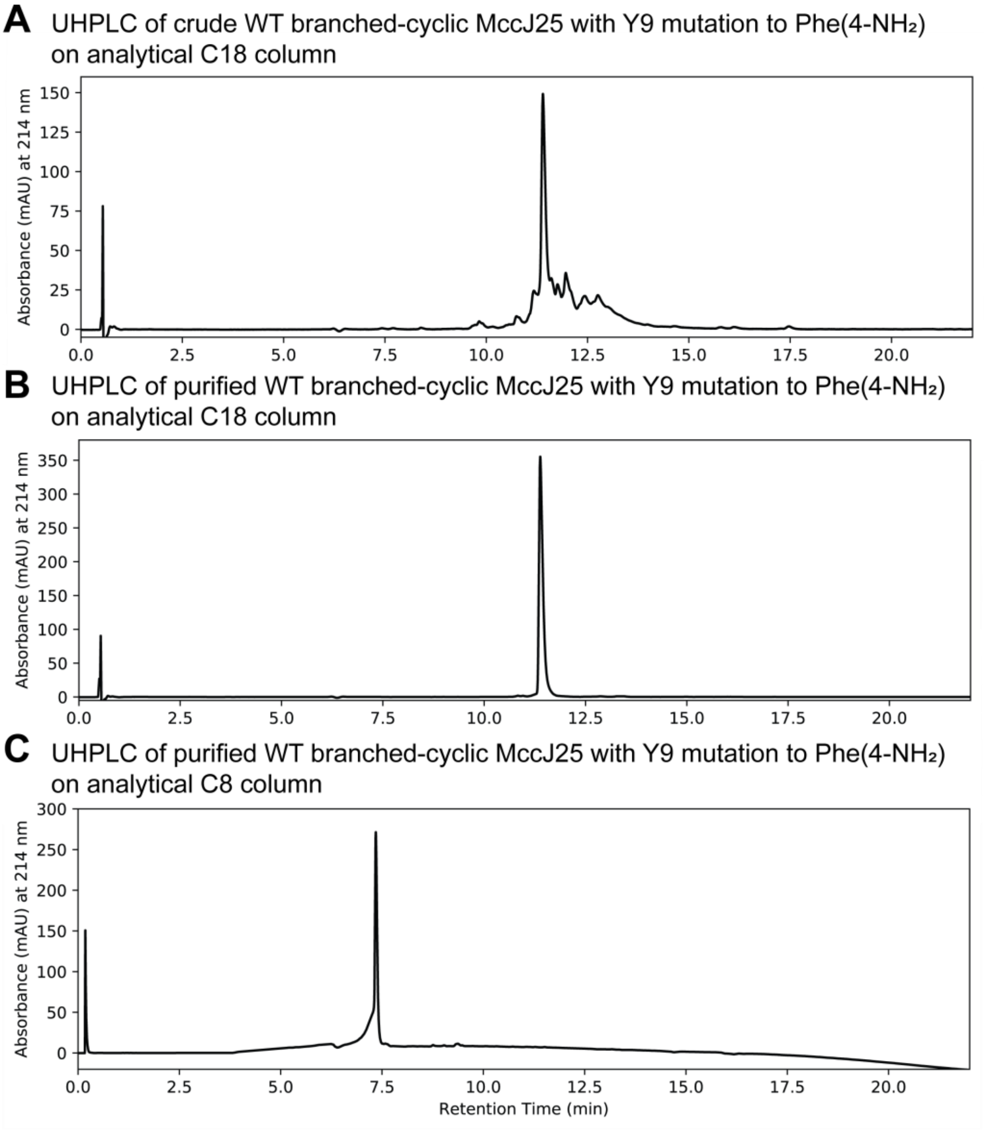

Fig. S47: UHPLC profile of wild-type branched-cyclic MccJ25 with Y9 mutation to Phe(4-NH$_2$): (A) crude sample on analytical C18 column (46% purity); (B) purified sample on analytical C18 column (>95% purity, R_t = 11.38 min); (C) purified sample on analytical C8 column (>95% purity, R_t = 7.35 min).

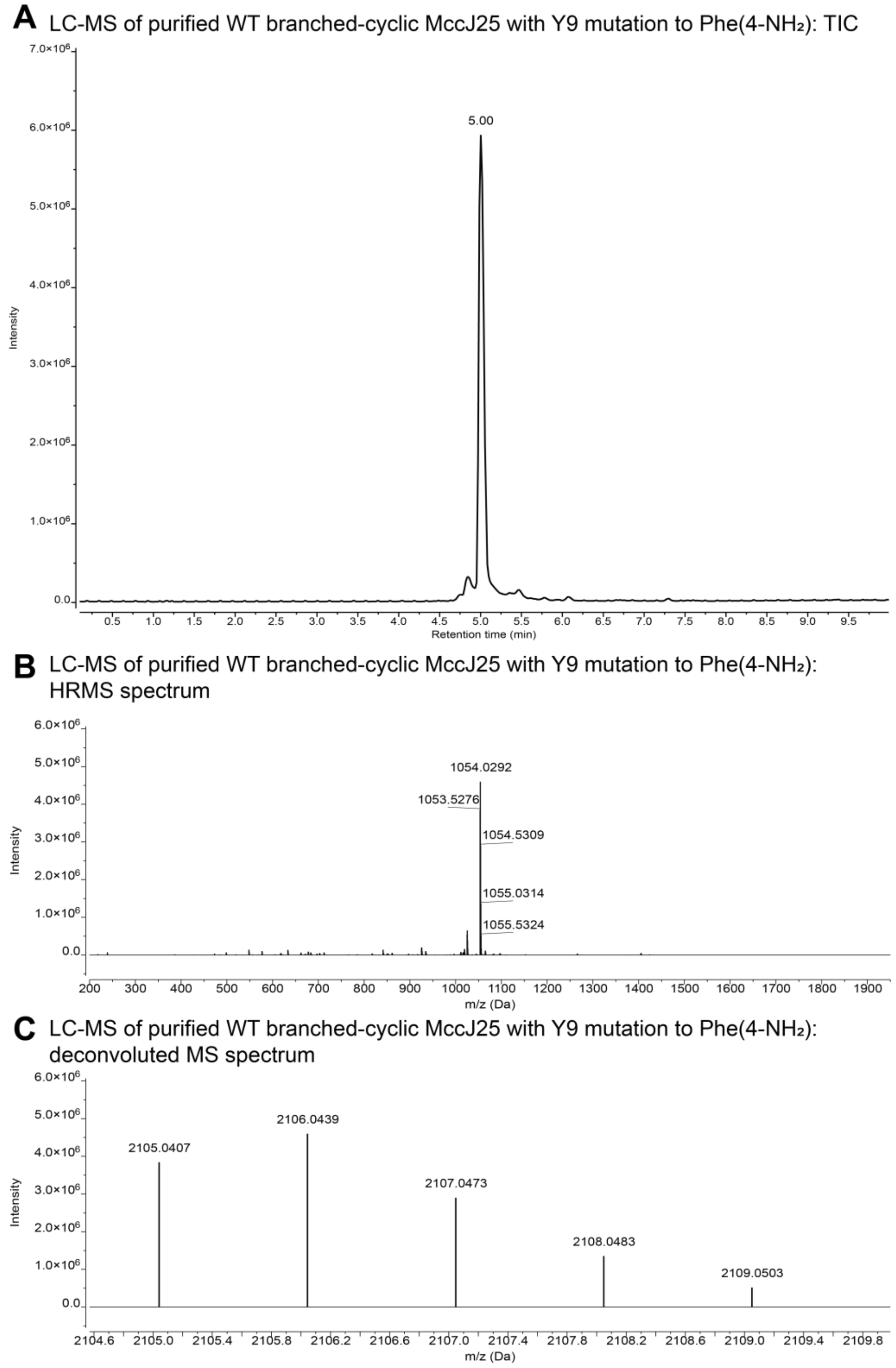

Fig. S48: LC-MS analysis of wild-type branched-cyclic MccJ25 with Y9 mutation to Phe(4-NH$_2$): (A) Total ion chromatogram (TIC). (B) MS spectrum. (C) deconvoluted MS spectrum: Monoisotopic mass (ESI+): calc. [$C_{101}H_{140}N_{24}O_{26}$]: 2105.0371, found: 2105.0407; Average mass calc. [$C_{101}H_{140}N_{24}O_{26}$]: 2106.3730.

2.6.3 Branched-cyclic MccJ25 with Tyr(3-*t*Bu) = Y’ (**bc-6’**)

Sequence: `H₂N-GGAGHVPEY’F VGIGTPISFY G-OH`

The peptide was prepared via the procedure described in 1.5 using NovaPEG HMPB resin (loading = 0.62 mmol/g, 84 mg, 52 μmol). One portion of the peptidyl-resin of the linear peptide (47% of total resin) was deprotected and cyclized. Cleavage of the peptidyl-resin afforded the crude peptide (46% purity (C18) by UHPLC). The peptide was purified using semi-preparative HPLC as specified in the general procedure 1.9. Fractions were analyzed by LC-HR-ESI-MS, combined and lyophilized to obtain 1.1 mg (2% yield; >95% purity (C18) and >95% purity (C8) by UHPLC) of the desired peptide.

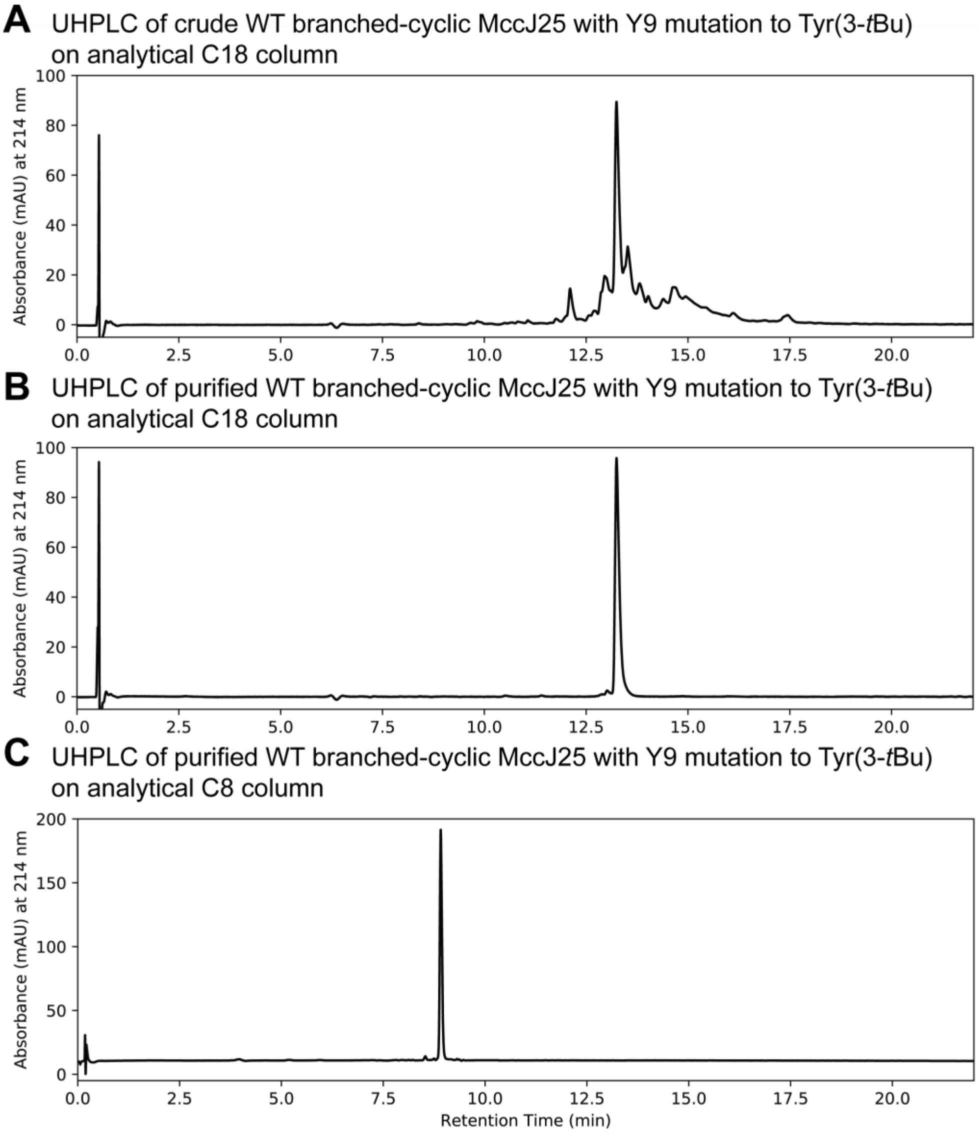

Fig. S49: UHPLC profile of wild-type branched-cyclic MccJ25 with Y9 mutation to Tyr(3-*t*Bu): (A) crude sample on analytical C18 column (46% purity); (B) purified sample on analytical C18 column (>95% purity, R_t = 13.25 min); (C) purified sample on analytical C8 column (>95% purity, R_t = 8.91 min).

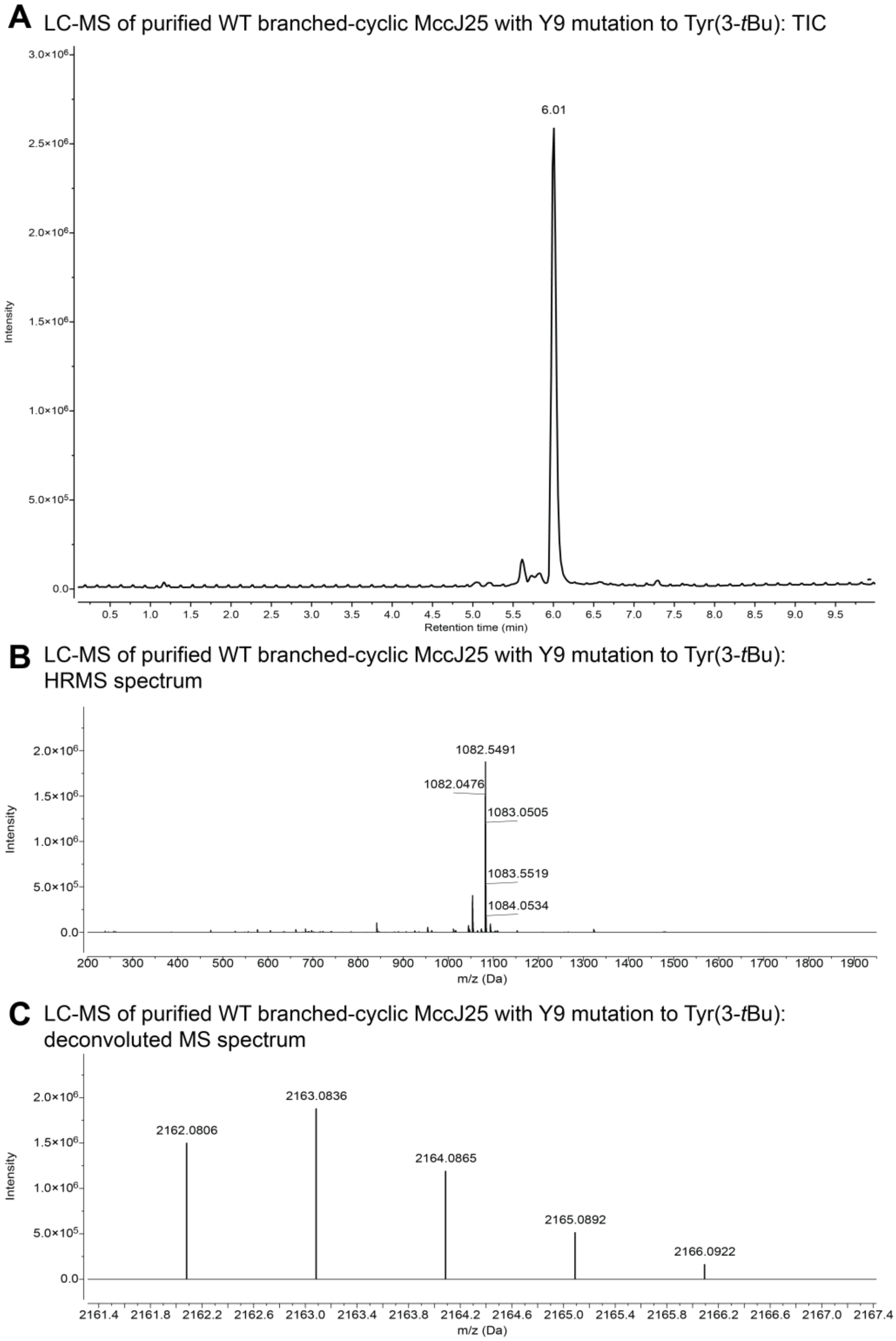

Fig. S50: LC-MS analysis of wild-type branched-cyclic MccJ25 with Y9 mutation to Tyr(3-*t*Bu): (A) Total ion chromatogram (TIC). (B) MS spectrum. (C) deconvoluted MS spectrum: Monoisotopic mass (ESI+): calc. [$C_{105}H_{147}N_{23}O_{27}$]: 2162.0837, found: 2162.0806; Average mass calc. [$C_{101}H_{140}N_{24}O_{26}$]: 2163.4650.

2.6.4 Branched-cyclic Link-MccJ25 with L-His (**bc-9'**)

Sequence: H_2N-GGAGHVPEYF VHFGIPISFY G-OH

The peptide was prepared via the procedure described in 1.5 using NovaPEG HMPB resin (loading = 0.62 mmol/g, 85 mg, 53 µmol). One portion of the peptidyl-resin of the linear peptide (45% of total resin) was deprotected and cyclized. Cleavage of the peptidyl-resin afforded the crude peptide (77% purity (C18) by UHPLC). The peptide was purified using semi-preparative HPLC as specified in the general procedure 1.9. Fractions were analyzed by LC-HR-ESI-MS, combined and lyophilized to obtain 4.3 mg (8% yield; >95% purity (C18) and >95% purity (C8) by UHPLC) of the desired peptide.

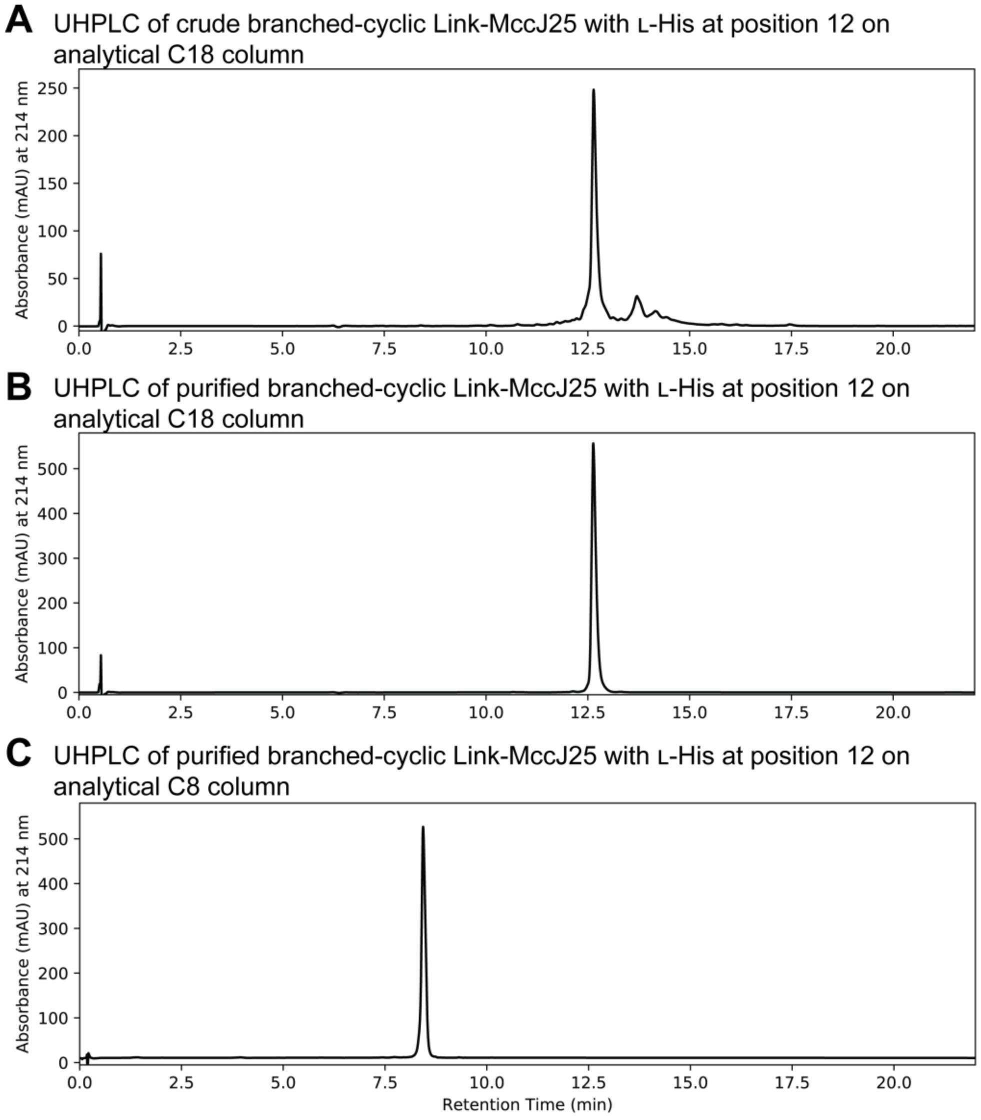

Fig. S51: UHPLC profile of branched-cyclic Link-MccJ25 with L-His at position 12: (A) crude sample on analytical C18 column (77% purity); (B) purified sample on analytical C18 column (>95% purity, R_t = 12.63 min); (C) purified sample on analytical C8 column (>95% purity, R_t = 8.43 min).

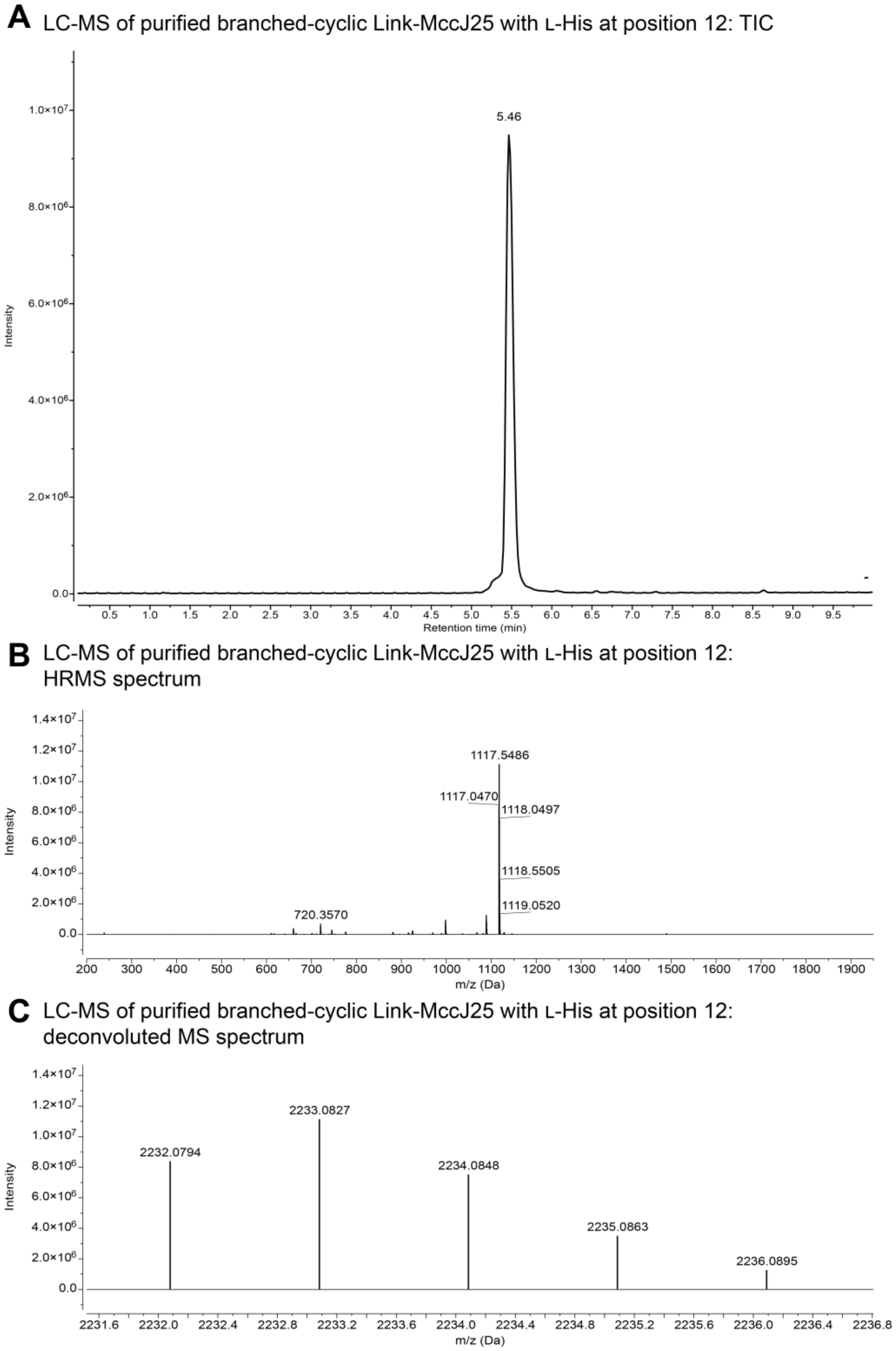

Fig. S52: **LC-MS analysis of branched-cyclic Link-MccJ25 with L-His at position 12:** (A) Total ion chromatogram (TIC). (B) MS spectrum. (C) deconvoluted MS spectrum: Monoisotopic mass (ESI+): calc. [$C_{110}H_{145}N_{25}O_{26}$]: 2232.0793, found: 2232.0794; Average mass calc. [$C_{110}H_{145}N_{25}O_{26}$]: 2233.5190.

2.6.5 Branched-cyclic Link-MccJ25 with D-His (**bc-10'**)

Sequence: H_2N-GGAGHVPEYF VhFGIPISFY G-OH

The peptide was prepared via the procedure described in 1.5 using NovaPEG HMPB resin (loading = 0.62 mmol/g, 80 mg, 50 µmol). One portion of the peptidyl-resin of the linear peptide (52% of total resin) was deprotected and cyclized. Cleavage of the peptidyl-resin afforded the crude peptide (50% purity (C18) by UHPLC). The peptide was purified using semi-preparative HPLC as specified in the general procedure 1.9. Fractions were analyzed by LC-HR-ESI-MS, combined and lyophilized to obtain 6.5 mg (11% yield; >95% purity (C18) and >95% purity (C8) by UHPLC) of the desired peptide.

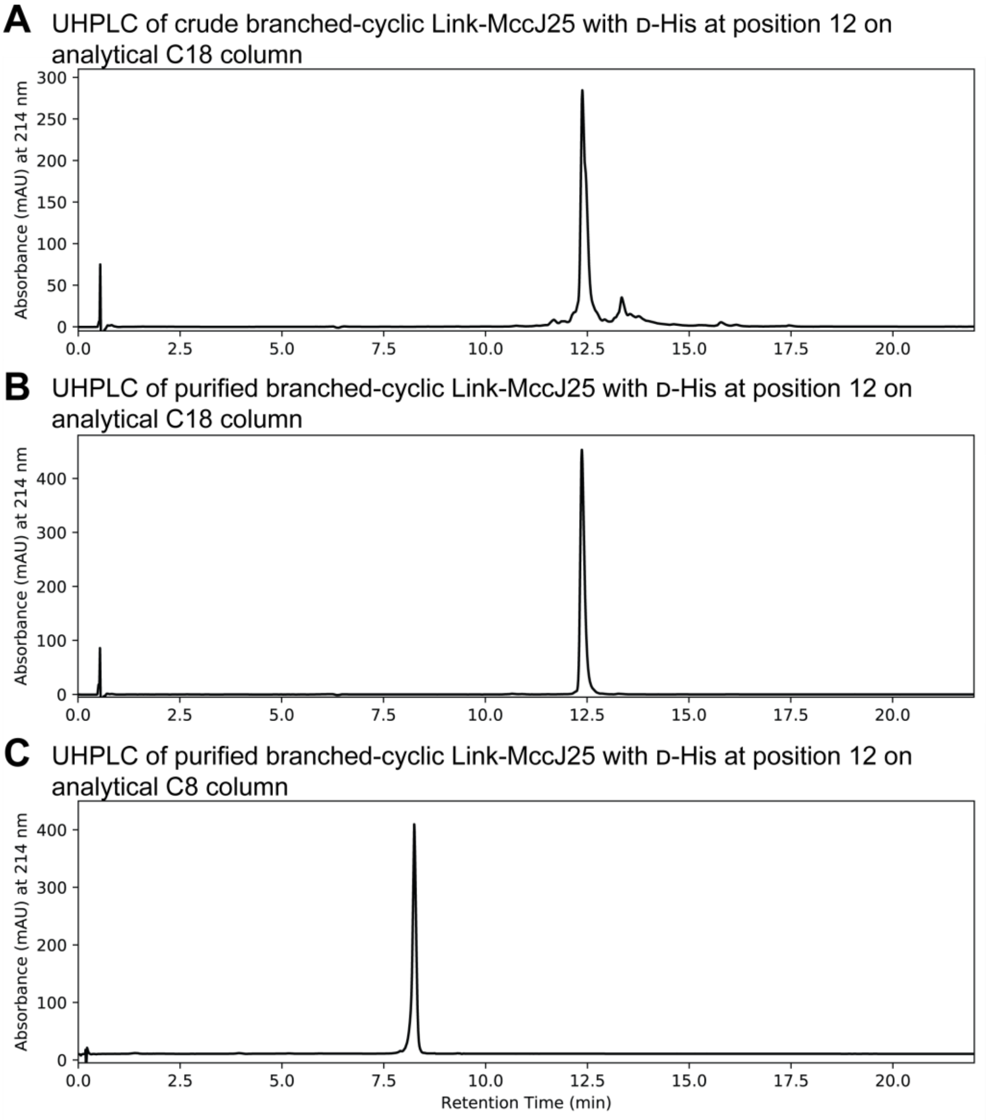

Fig. S53: UHPLC profile of branched-cyclic Link-MccJ25 with D-His at position 12: (A) crude sample on analytical C18 column (50% purity); (B) purified sample on analytical C18 column (>95% purity, R_t = 12.37 min); (C) purified sample on analytical C8 column (>95% purity, R_t = 8.25 min).

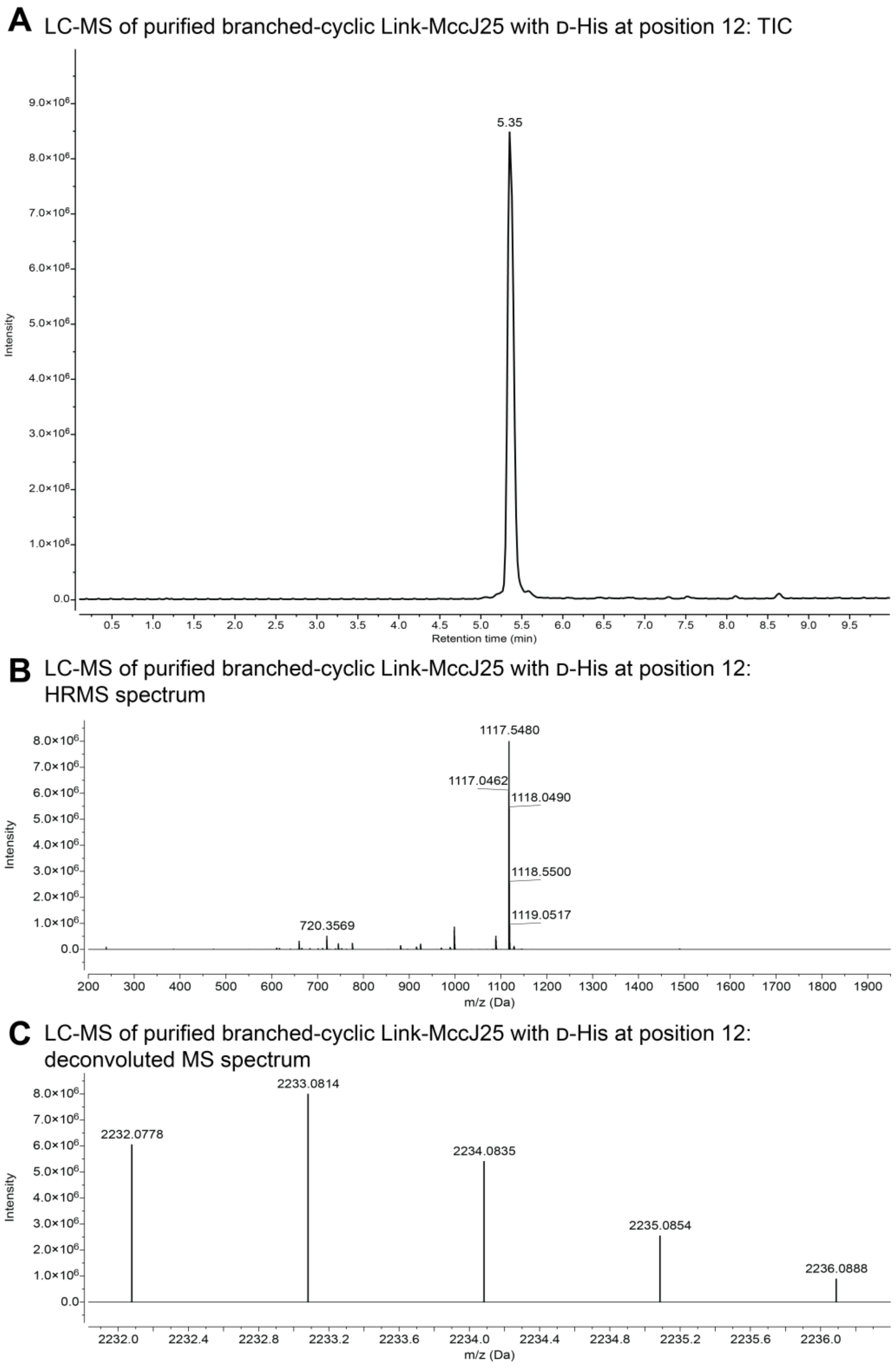

Fig. S54: LC-MS analysis of branched-cyclic Link-MccJ25 with D-His at position 12: (A) Total ion chromatogram (TIC). (B) MS spectrum. (C) deconvoluted MS spectrum: Monoisotopic mass (ESI+): calc. [$C_{110}H_{145}N_{25}O_{26}$]: 2232.0793, found: 2232.0778; Average mass calc. [$C_{110}H_{145}N_{25}O_{26}$]: 2233.5190.

2.6.6 Branched-cyclic Link-MccJ25 with three D-amino acids (**bc-15’**)

Sequence: H_2N-GGAGHVPEYF VhfGiPISFY G-OH

The peptide was prepared via the procedure described in 1.5 using NovaPEG HMPB resin (loading = 0.62 mmol/g, 79 mg, 49 µmol). One portion of the peptidyl-resin of the linear peptide (51% of total resin) was deprotected and cyclized. Cleavage of the peptidyl-resin afforded the crude peptide (57% purity (C18) by UHPLC). The peptide was purified using semi-preparative HPLC as specified in the general procedure 1.9. Fractions were analyzed by LC-HR-ESI-MS, combined and lyophilized to obtain 4.4 mg (8% yield; >95% purity (C18) and >95% purity (C8) by UHPLC) of the desired peptide.

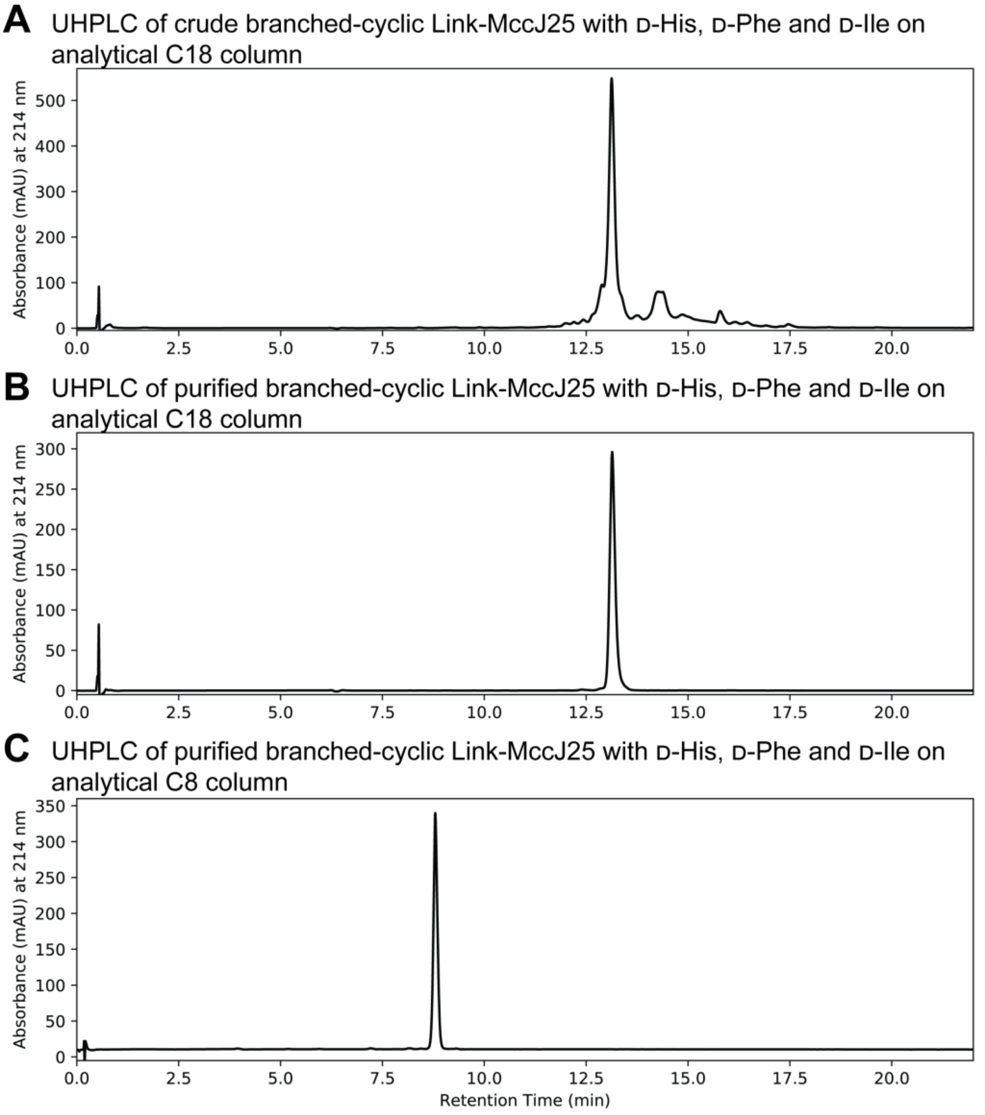

Fig. S55: UHPLC profile of branched-cyclic Link-MccJ25 with D-His-12, D-Phe-13, and D-Ile-15: (A) crude sample on analytical C18 column (57% purity); (B) purified sample on analytical C18 column (>95% purity, R_t = 13.14 min); (C) purified sample on analytical C8 column (>95% purity, R_t = 8.80 min).

A LC-MS of purified branched-cyclic Link-MccJ25 with D-His, D-Phe and D-Ile: TIC

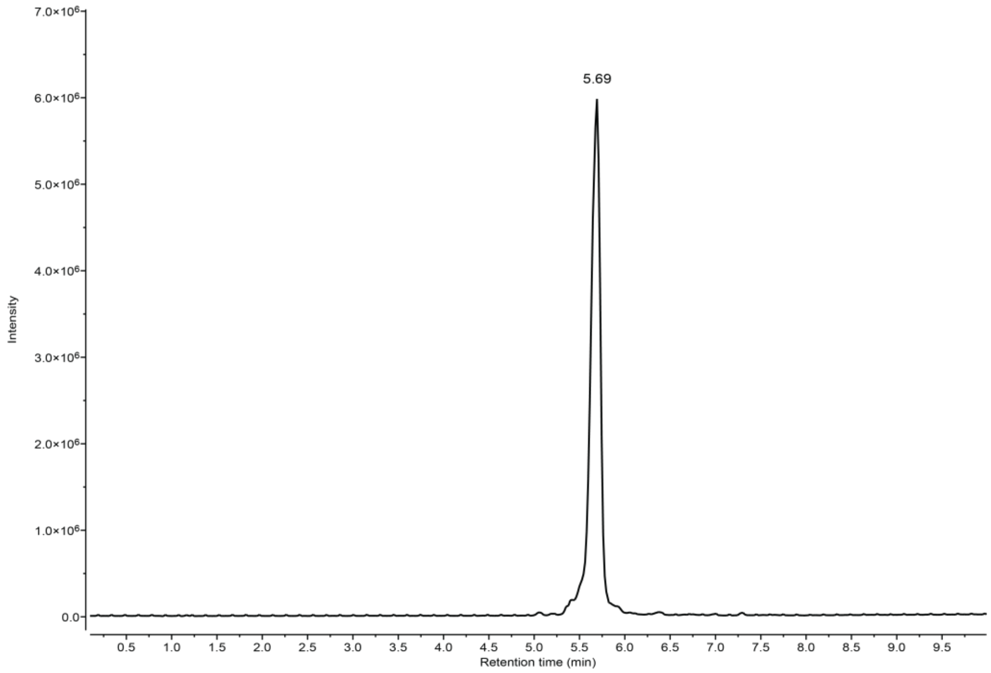

B LC-MS of purified branched-cyclic Link-MccJ25 with D-His, D-Phe and D-Ile: HRMS spectrum

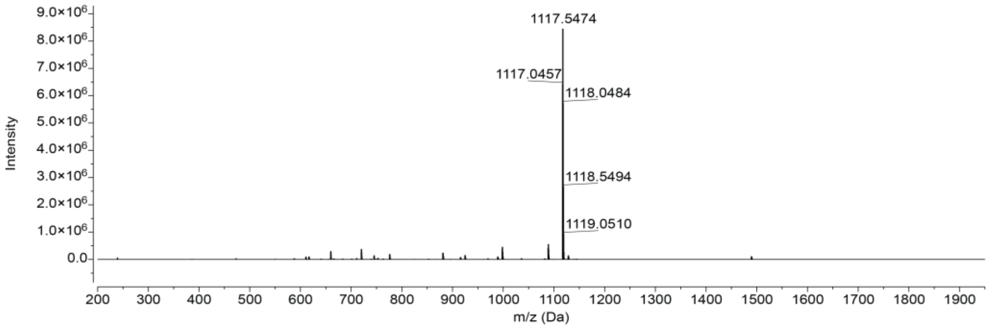

C LC-MS of purified branched-cyclic Link-MccJ25 with D-His, D-Phe and D-Ile: deconvoluted MS spectrum

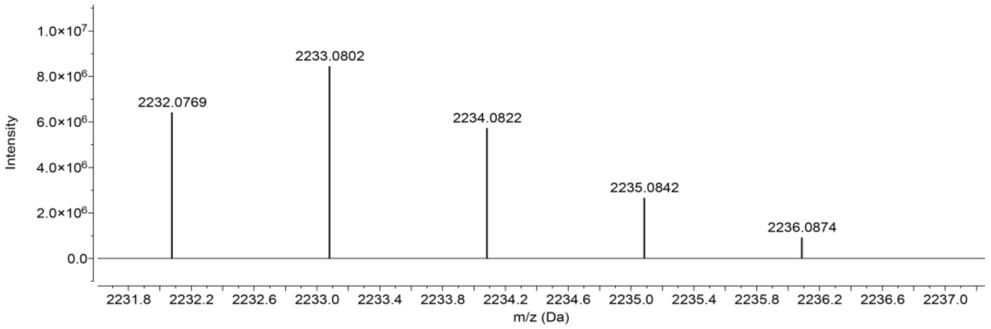

Fig. S56: LC-MS analysis of branched- cyclic Link-MccJ25 with D-His-12, D-Phe-13, and D-Ile-15: (A) Total ion chromatogram (TIC). (B) MS spectrum. (C) deconvoluted MS spectrum: Monoisotopic mass (ESI+): calc. [$C_{110}H_{145}N_{25}O_{26}$]: 2232.0793, found: 2232.0769; Average mass calc. [$C_{110}H_{145}N_{25}O_{26}$]: 2233.5190.

2.6.7 Branched-cyclic MccJ25 with *N*-Me-Gly12 (**bc-16'**)

Sequence: H_2N-GGAGHVPEYF VGIGTPISFY G-OH

The peptide was prepared via the procedure described in 1.5 using TentaGel XV HMPA resin (loading = 0.29 mmol/g, 108 mg, 31 μmol). One portion of the peptidyl-resin of the linear peptide (30% of total resin) was deprotected and cyclized. Cleavage of the peptidyl-resin afforded the crude peptide (64% purity (C18) by UHPLC). The peptide was purified using semi-preparative HPLC as specified in the general procedure 1.9. Fractions were analyzed by LC-HR-ESI-MS, combined, and lyophilized to obtain 4.6 mg (23% yield; >XX% purity (C18) and >95% purity (C8) by UHPLC) of the desired peptide.

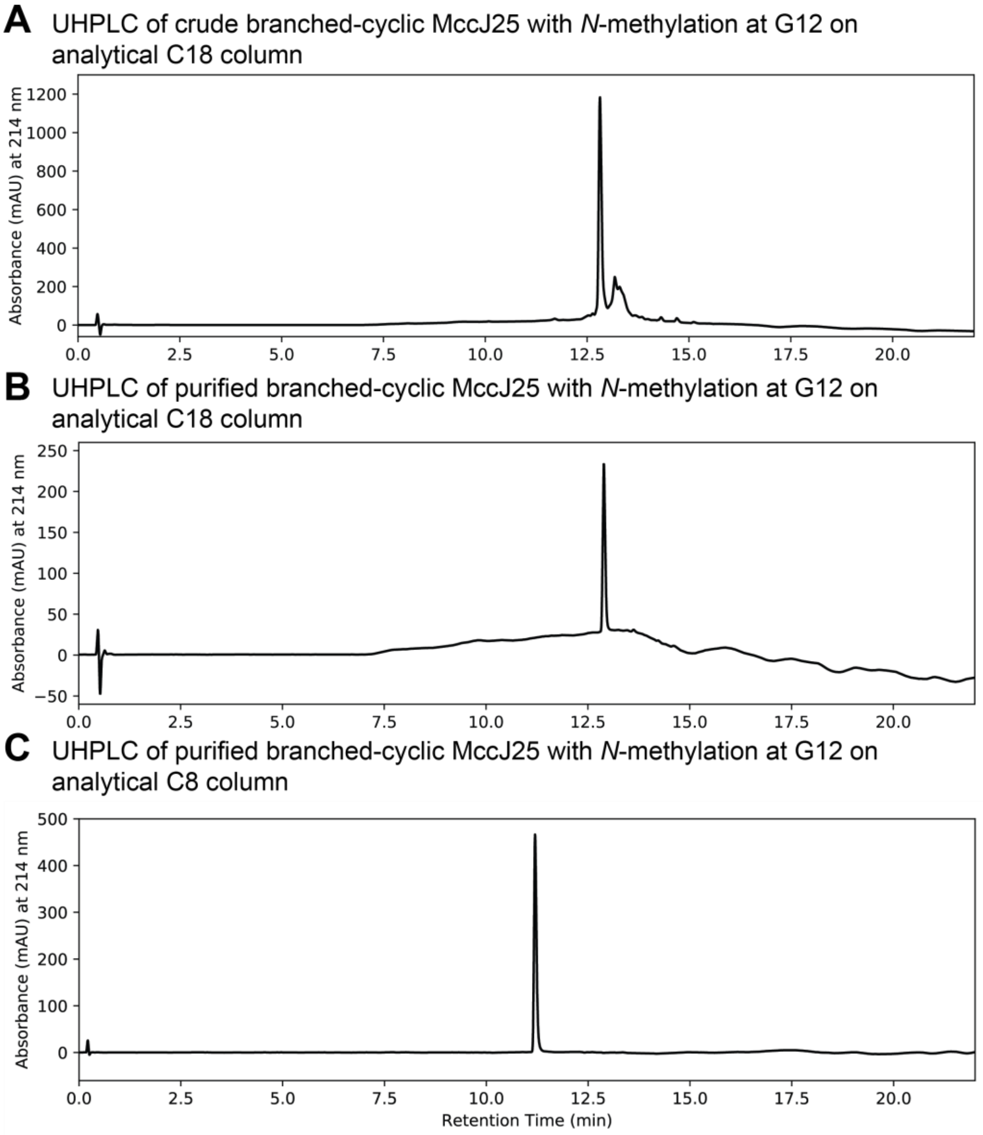

Fig. S57: UHPLC profile of branched-cyclic MccJ25 with *N*-Me-Gly12: (A) crude sample on analytical C18 column (64% purity); (B) purified sample on analytical C18 column (>95% purity, R_t = 12.89 min); (C) purified sample on analytical C8 column (>95% purity, R_t = 11.20 min).

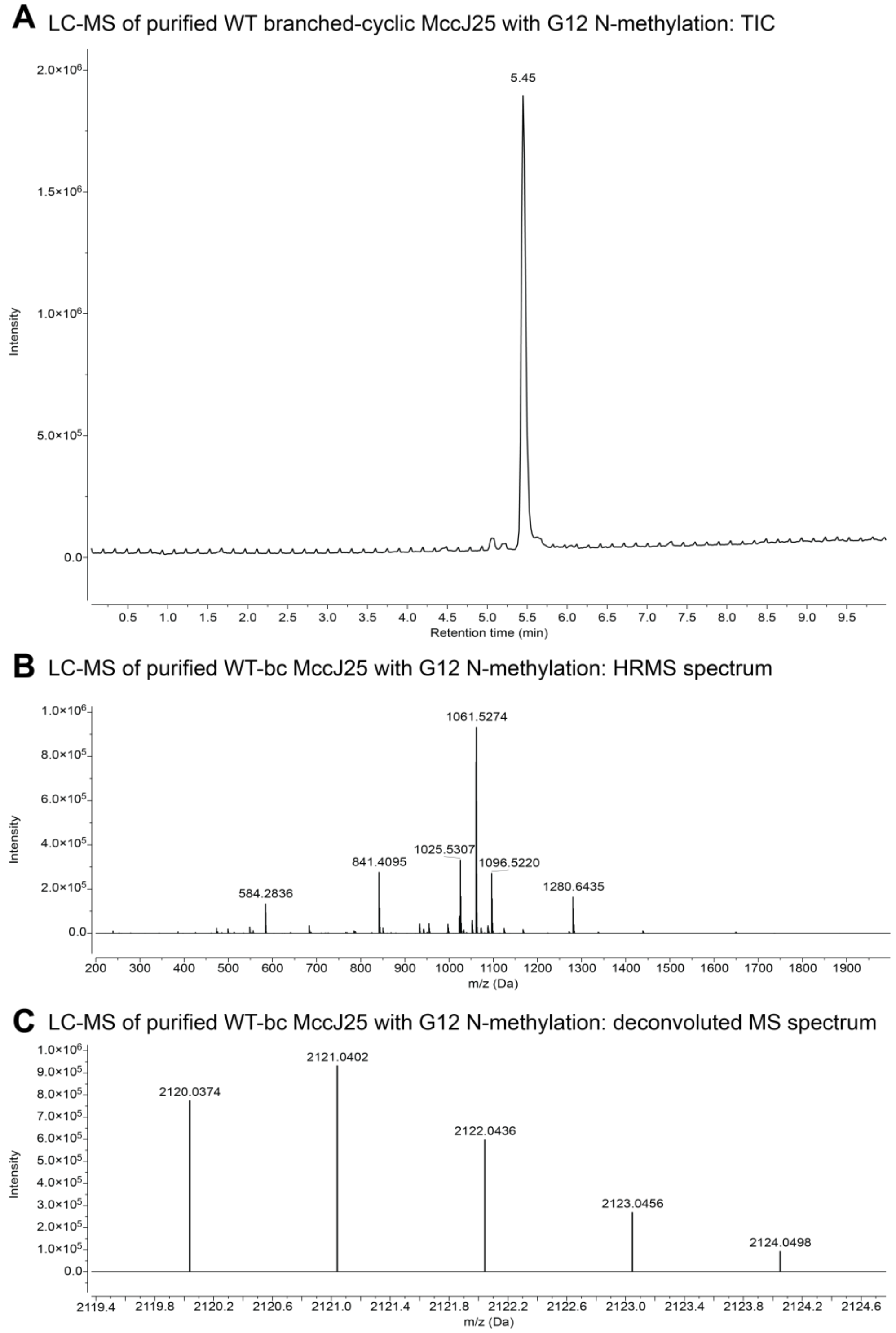

Fig. S58: **LC-MS analysis of branched-cyclic MccJ25 with *N*-Me-Gly12:** (A) Total ion chromatogram (TIC)[B]. (B) MS spectrum. (C) deconvoluted MS spectrum: Monoisotopic mass (ESI+): calc. [$C_{102}H_{141}N_{23}O_{27}$]: 2120.0367, found: 2120.0374; Average mass calc. [$C_{102}H_{141}N_{23}O_{27}$]: 2121.3840. MS-spectrum shows fragmentation due to N-methylation.

[B] The baseline peaks results from lower sample concentration. Therefore, added references show up during the LC-MS measurements.

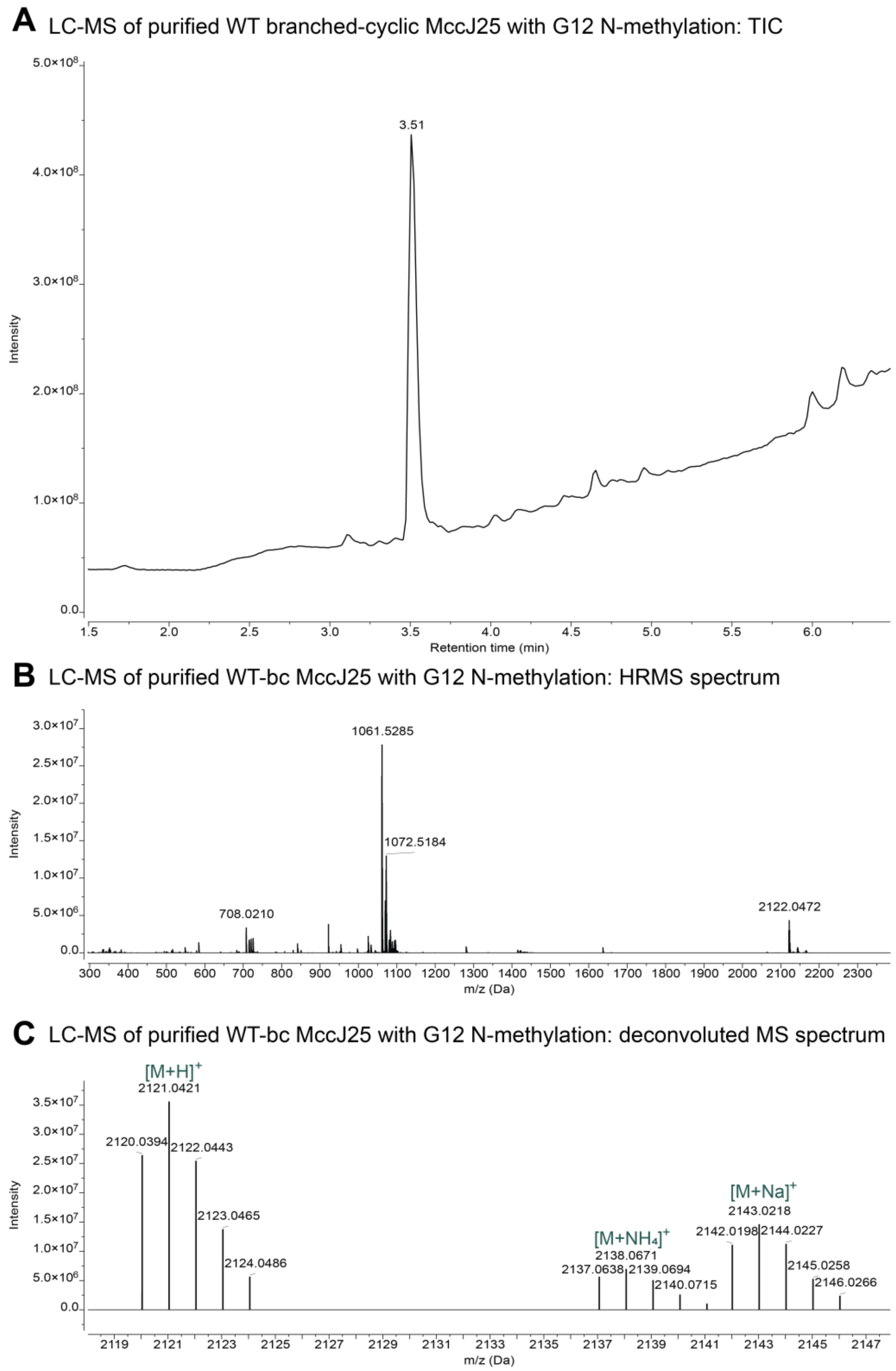

Fig. S59: LC-MS analysis of branched-cyclic MccJ25 with *N*-Me-Gly12 on Agilent LC-MS using lower fragmentator voltage: (A) Total ion chromatogram (TIC). (B) MS spectrum. (C) deconvoluted MS spectrum: Monoisotopic mass (ESI+): calc. [$C_{102}H_{141}N_{23}O_{27}$]: 2120.0367, found: 2120.0394; Average mass calc. [$C_{102}H_{141}N_{23}O_{27}$]: 2121.3840.

2.6.8 Branched-cyclic MccJ25 with *N*-Me-Gly12 and *N*-Me-Gly14 (**bc-17'**)

Sequence: H_2N-GGAGHVPEYF VGIGTPISFY G-OH

The peptide was prepared via the procedure described in 1.5 using TentaGel XV HMPA resin (loading = 0.29 mmol/g, 105 mg, 30 µmol). One portion of the peptidyl-resin of the linear peptide (32% of total resin) was deprotected and cyclized. Cleavage of the peptidyl-resin afforded the crude peptide (67% purity (C18) by UHPLC). The peptide was purified using semi-preparative HPLC as specified in the general procedure 1.9. Fractions were analyzed by LC-HR-ESI-MS, combined, and lyophilized to obtain 4.1 mg (20% yield; >95% purity (C18) and >95% purity (C8) by UHPLC) of the desired peptide.

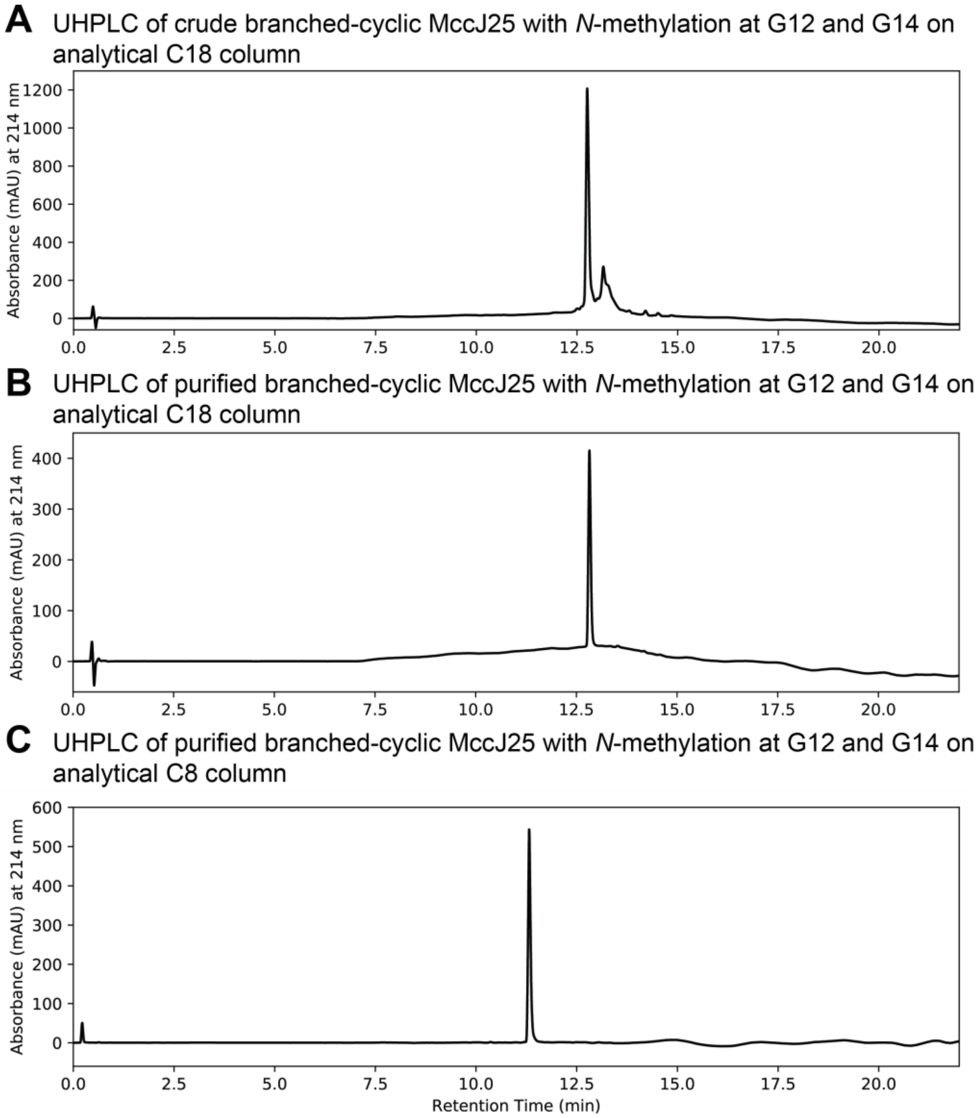

Fig. S60: UHPLC profile of branched-cyclic MccJ25 with *N*-Me-Gly12 and *N*-Me-Gly14: (A) crude sample on analytical C18 column (67% purity); (B) purified sample on analytical C18 column (>95% purity, R_t = 12.82 min); (C) purified sample on analytical C8 column (>95% purity, R_t = 11.32 min).

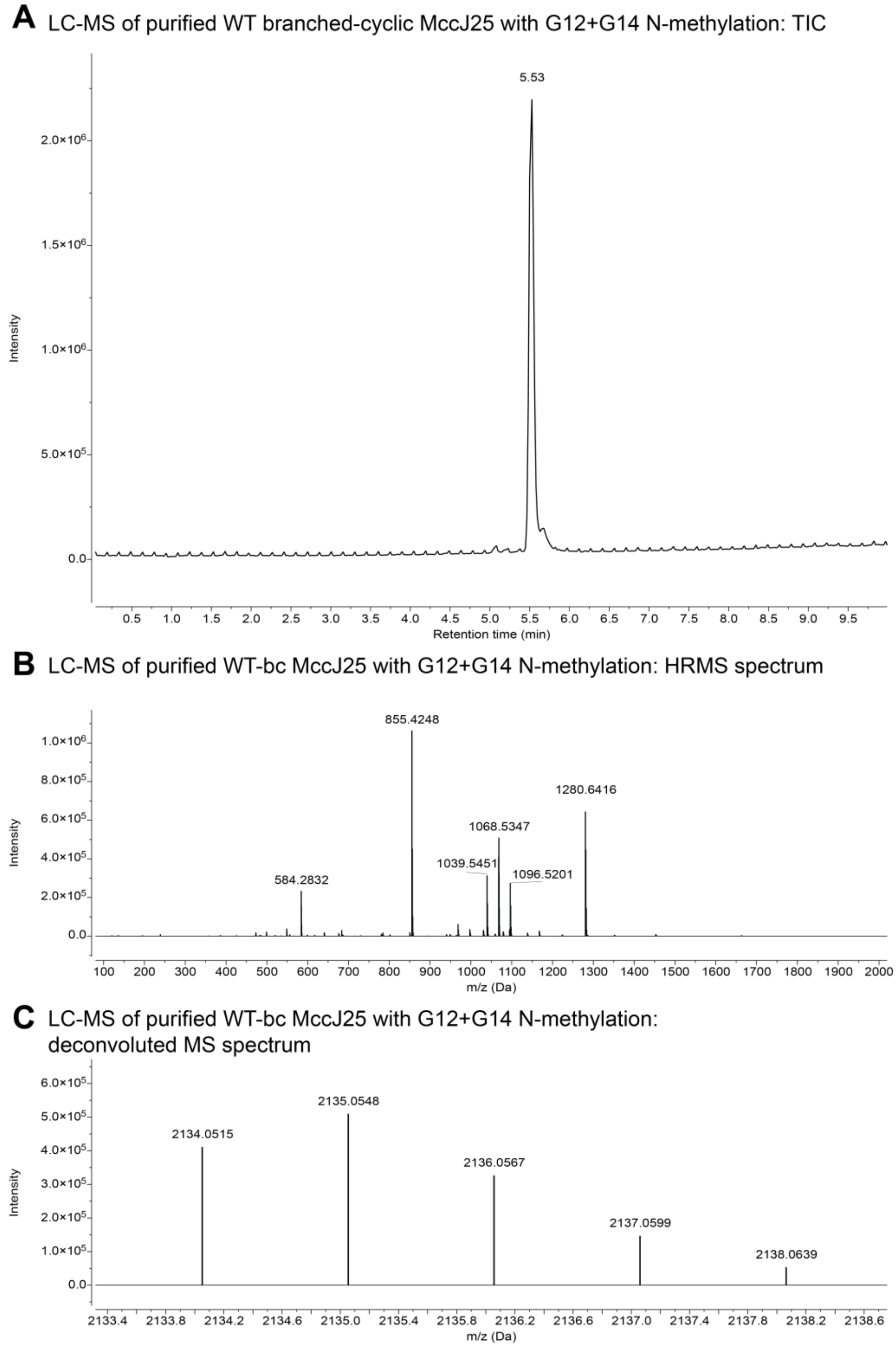

Fig. S61: **LC-MS analysis of branched-cyclic MccJ25 with *N*-Me-Gly12 and *N*-Me-Gly14:** (A) Total ion chromatogram (TIC).[C] (B) MS spectrum. (C) deconvoluted MS spectrum: Monoisotopic mass (ESI+): calc. [$C_{103}H_{143}N_{23}O_{27}$]: 2134.0524, found: 2134.0515; Average mass calc. [$C_{103}H_{143}N_{23}O_{27}$]: 2135.4110. MS-spectrum shows fragmentation due to N-methylation.

[C] The baseline peaks results from lower sample concentration. Therefore, added references show up during the LC-MS measurements.

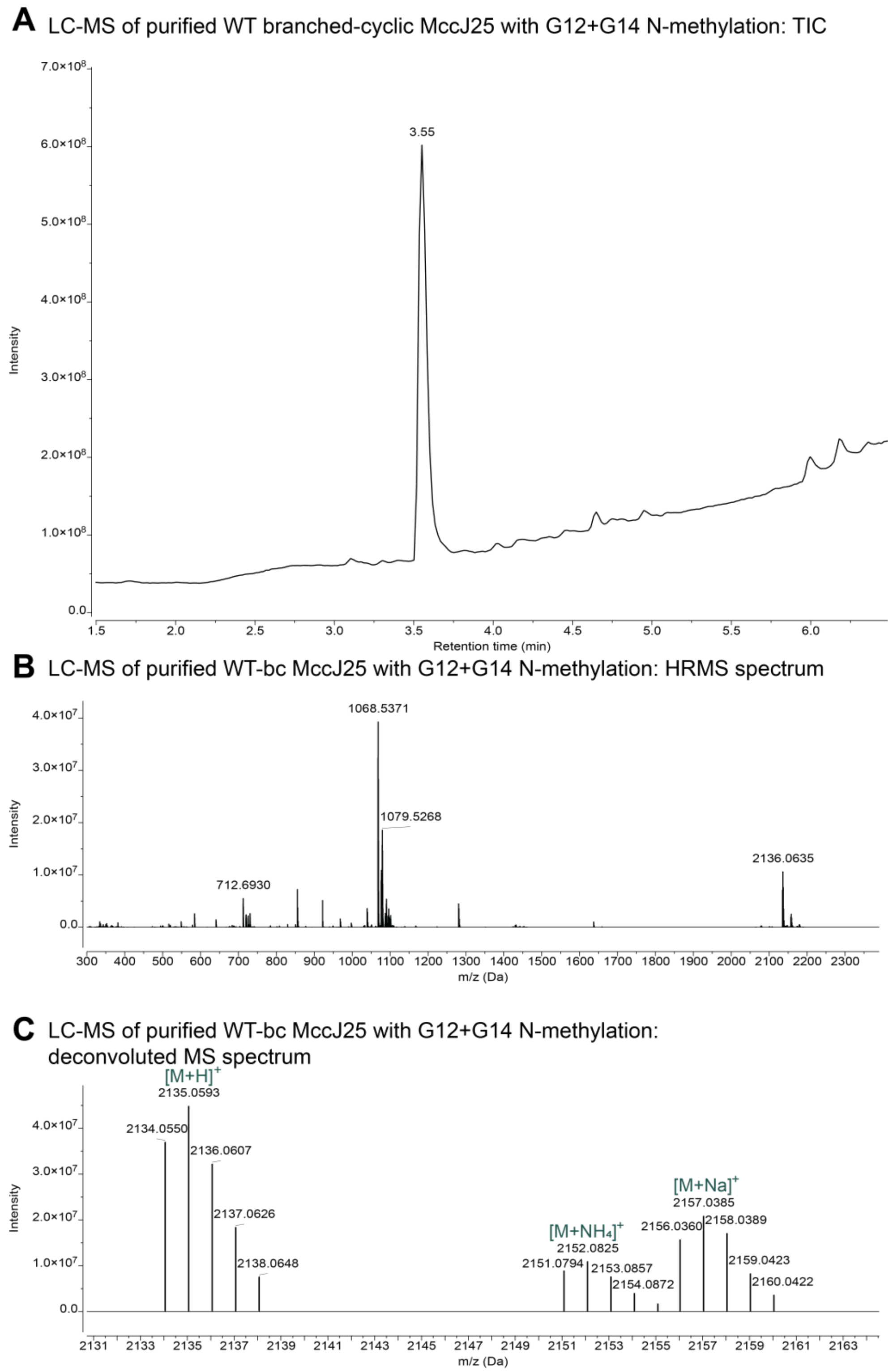

Fig. S62: LC-MS analysis of branched-cyclic MccJ25 with *N*-Me-Gly12 and *N*-Me-Gly14 on Agilent LC-MS using lower fragmentator voltage: (A) Total ion chromatogram (TIC). (B) MS spectrum. (C) deconvoluted MS spectrum: Monoisotopic mass (ESI+): calc. [$C_{103}H_{143}N_{23}O_{27}$]: 2134.0524, found: 2134.0515; Average mass calc. [$C_{103}H_{143}N_{23}O_{27}$]: 2135.4110.

3. Expression of lasso-processing enzymes McjB and McjC

3.1 Optimization of His_6-MBP-McjB expression

3.1.1 Protein sequence of His_6-MBP-McjB

His_6-MBP-McjB

```
MAHHHHHHKI EEGKLVIWIN GDKGYNGLAE VGKKFEKDTG IKVTVEHPDK LEEKFPQVAA TGDGPDIIFW
AHDRFGGYAQ SGLLAEITPD KAFQDKLYPF TWDAVRYNGK LIAYPIAVEA LSLIYNKDLL PNPPKTWEEI
PALDKELKAK GKSALMFNLQ EPYFTWPLIA ADGGYAFKYE NGKYDIKDVG VDNAGAKAGL TFLVDLIKNK
HMNADTDYSI AEAAFNKGET AMTINGPWAW SNIDTSKVNY GVTVLPTFKG QPSKPFVGVL SAGINAASPN
KELAKEFLEN YLLTDEGLEA VNKDKPLGAV ALKSYEEELV KDPRIAATME NAQKGEIMPN IPQMSAFWYA
VRTAVINAAS  GRQTVDEALK  DAQTNSSSNN  NNNNNNNNLG  LEVLFQGPLG  SIRYCLTSYR  EDLVILDIIN
DSFSIVPDAG SLLKERDKLL KEFPQLSYFF DSEYHIGSVS RNSDTSFLEE RWFLPEPDKT LYKCSLFKRF
ILLLKVFYYS WNIEKKGMAW IFISNKKENR LYSLNEEHLI RKEISNLSII FHLNIFKSDC LTYSYALKRI
LNSRNIDAHL VIGVRTQPFY SHSWVEVGGQ VINDAPNMRD KLSVIAEI
```

Molecular weight: 68377.75 Da (without Met1)

3.1.2 DNA sequence encoding for McjB

```
ATCCGTTACTGCTTAACCAGTTATAGAGAGGATCTTGTTATCCTGGATATAATTAATGATAGTTTCAGCATAGTGC
CTGACGCAGGTAGCTTGCTAAAAGAAAGAGATAAATTGCTTAAAGAATTCCCACAACTATCTTACTTTTTTGACAG
TGAATATCATATTGGAAGTGTTTCTCGTAATAGTGACACTTCTTTTCTTGAAGAACGCTGGTTTCTACCAGAACCT
GACAAAACATTATATAAGTGTTCTCTATTTAAACGATTTATATTATTACTCAAAGTCTTTTACTATAGCTGGAATA
TTGAAAAAAAAGGGATGGCATGGATTTTCATAAGTAATAAAAAAGAGAATAGGCTATACTCCTTGAATGAAGAGCA
TCTTATCCGGAAAGAAATTAGTAATCTTTCCATTATCTTTCATCTTAATATTTTTAAATCTGACTGTCTTACCTAT
TCATACGCACTAAAAAGAATTCTTAATTCCAGAAATATTGATGCTCATCTTGTTATTGGTGTAAGGACACAACCTT
TTTATAGCCACTCTTGGGTGGAGGTTGGGGGACAAGTTATCAATGATGCTCCCAATATGCGGGATAAATTATCTGT
TATTGCAGAGATATAG
```

The plasmid used in this study was constructed by Leskop and coworkers.[6]

3.1.3 Purification via Ni-NTA vs Co resin

Single colonies of *E. coli* BL21(DE3) transformed with pMAL-c5ePre-mcjB were grown on agar plates with LB medium and carbenicillin (50 µg/mL). One colony of BL21(DE3) containing the expression plasmid was picked into 10 mL LB medium supplemented with antibiotics. The cells were incubated overnight at 37 °C with agitation. The preculture (3x2.5 mL) was added to three 2 L Erlenmeyer flasks containing LB medium (3x500 mL) supplemented with carbenicillin (48 µg/mL) and the growth was allowed to proceed at 37 °C until OD_{600} reached 0.7-0.85 (around 3.5 h at 37 °C with rigorous agitation). The culture had been cooled down on ice for around 60 min before IPTG at a final concentration of 0.5 mM was added to induce protein expression. The culture was further grown at 18 °C for 24 h before harvesting. The cells were recovered by centrifugation (5000 rpm, 15 min at 4 °C) and the cell pellets were flash-frozen in liquid nitrogen before storage at -80 °C.

The cells were resuspended in lysis buffer [20 mL, Tris-HCl (50 mM) pH 7.8, imidazole (10 mM), NaCl (300 mM), glycerol (10%), magnesium chloride (5 mM), DNAse I (5 µg/mL), RNAse (10 µg/mL), and lysozyme (0.5 mg/mL)] at 4 °C. The cells were lysed by the EmulsiFlex® High-Pressure Homogenizer circulating the cells for around 5 min. The lysate was centrifuged (18000 rpm, 30 min, 4 °C) and half of the supernatant (11.4 mL) was applied to a pre-equilibrated Ni-NTA resin (2 mL of 50% agarose slurry); the

other half (11.4 mL) to a pre-equilibrated Co resin (2 mL of 50% agarose slurry) column on ice. The columns were closed and shaken on ice for 1 h before the solution was drained by gravity flow. The column was washed with resuspension buffer [2x10 mL, Tris-HCl (50 mM) pH 7.8, imidazole (10 mM), NaCl (300 mM), glycerol (10%), and magnesium chloride (5 mM)] and wash buffer [5 mL, Tris-HCl (50 mM) pH 7.8, imidazole (50 mM), NaCl (300 mM), glycerol (10%), and magnesium chloride (5 mM)]. The protein was eluted with elution buffer [8 mL, Tris-HCl (50 mM) pH 7.8, imidazole (250 mM), NaCl (300 mM), glycerol (10%), and magnesium chloride (5 mM)].

The sample was concentrated using an Amicon centrifugal device (10 kDa cutoff) and buffer exchange was performed with a PD-10 column into the storage buffer [Tris-HCl (50 mM) pH 7.5, NaCl (100 mM), glycerol (10%), and magnesium chloride (1 mM)]. The purified protein was again concentrated using an Amicon centrifugal device (30 kDa cutoff) and flash-frozen in liquid nitrogen to be stored at -80 °C.

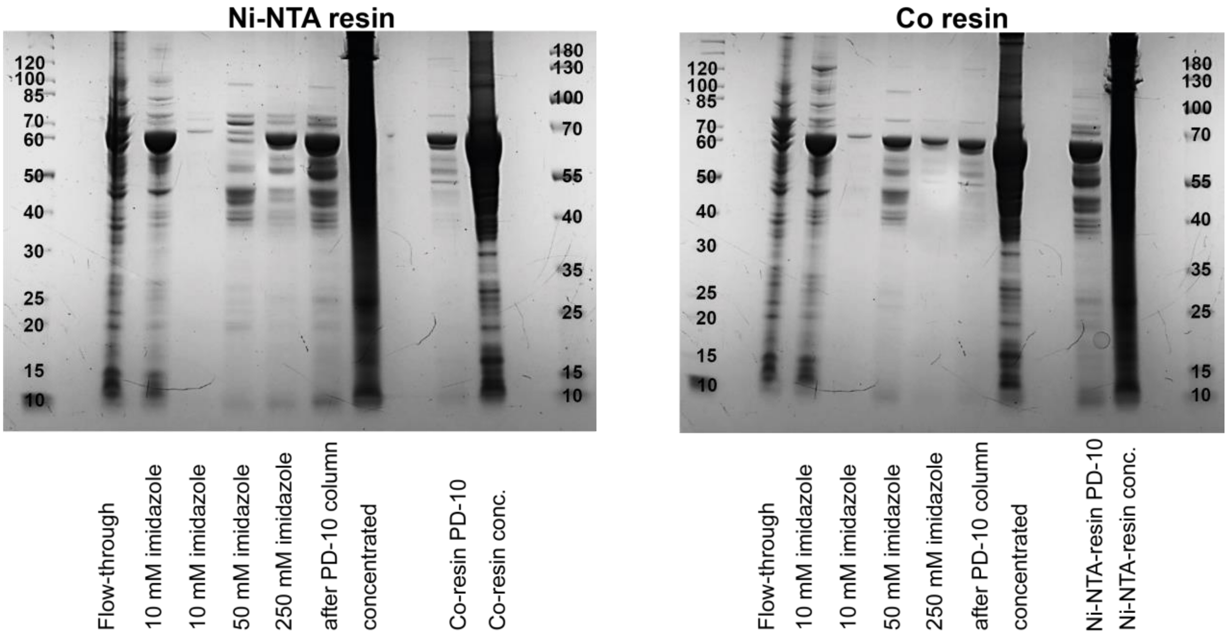

Fig. S63: SDS-PAGE analysis of fractions during His$_6$-MBP-McjB purification with Ni-NTA resin or Co resin.

3.1.4 Expression in different growth medium and cell lines

Single colonies of the *E. coli* BL21(DE3), C41(DE3) and C43(DE3) transformed with pMAL-c5ePre-mcjB were grown on agar plates with LB medium and carbenicillin (50 µg/mL). One colony of the cell line containing the expression plasmid was picked into 4 mL LB medium supplemented with carbenicillin (50 µg/mL). The cells were incubated overnight at 37 °C with agitation. The precultures were added to two 2 L Erlenmeyer flask containing LB medium or TB medium (2x500 mL) supplemented with carbenicillin (48 µg/mL) and the growth was allowed to proceed at 37 °C until OD_{600} reached 0.7-0.85 (around 3.5 h at 37 °C with rigorous agitation). The cultures had been cooled down on ice for around 60 min before IPTG at a final concentration of 0.5 mM was added to induce the protein expression. The cultures were further grown at 18 °C for 24 h before harvesting. The cells were recovered by centrifugation (5000 rpm, 15 min at 4 °C), weighed out and the cell pellets were flash-frozen in liquid nitrogen before storage at -80 °C.

Table S1: Mass of cells before lysis depending on growth conditions during McjB expression.

Growth condition (cell line, medium)	Amount of cells
BL21(DE3) in TB medium	6.3 g
BL21(DE3) in LB medium	4.6 g
C41(DE3) in LB medium	4.0 g
C43(DE3) in LB medium	4.0 g

The procedure for all cell lines and both tested media were the same. The cells were resuspended in lysis buffer [10 mL, Tris-HCl (50 mM) pH 7.8, imidazole (10 mM), NaCl (300 mM), glycerol (10%), magnesium chloride (5 mM), DNAse I (5 µg/mL), RNAse (10 µg/mL) and lysozyme (0.5 mg/mL)] at 4 °C. The cells were lysed by the EmulsiFlex® High-Pressure Homogenizer circulating the cells for around 5 min. The lysate was centrifuged (18000 rpm, 30 min, 4 °C) and the supernatant was applied to a pre-equilibrated Ni-NTA (2 mL of 50% agarose slurry) column on ice, the columns was closed and shaken on ice for 1 h, before the solution was drained by gravity flow. The column was washed with resuspension buffer [2x10 mL, Tris-HCl (50 mM) pH 7.8, imidazole (10 mM), NaCl (300 mM), glycerol (10%), and magnesium chloride (5 mM)] and wash buffer [5 mL, Tris-HCl (50 mM) pH 7.8, imidazole (50 mM), NaCl (300 mM), glycerol (10%), and magnesium chloride (5 mM)]. The protein was eluted with elution buffer [8 mL, Tris-HCl (50 mM) pH 7.8, imidazole (250 mM), NaCl (300 mM), glycerol (10%), and magnesium chloride (5 mM)].

The sample was concentrated using an Amicon centrifugal device (10 kDa cutoff) and buffer exchange was performed with a PD-10 column into the storage buffer [Tris-HCl (50 mM) pH 7.5, NaCl (100 mM), glycerol (10%), and magnesium chloride (1 mM)]. The purified protein was again concentrated using an Amicon centrifugal device (30 kDa cutoff) and flash-frozen in liquid nitrogen to be stored at -80 °C.

Table S2: Amount of enzyme depending on growth conditions for McjB-expression.

Growing conditions	Volume of concentrated sample	Concentration of concentrated sample	Amount of protein
BL21(DE3) in TB	260 µL	342 µM	6.1 mg
BL21(DE3) in LB	200 µL	219 µM	3.0 mg
C41(DE3) in LB	220 µL	235 µM	3.5 mg
C43(DE3) in LB	200 µL	263 µM	3.6 mg

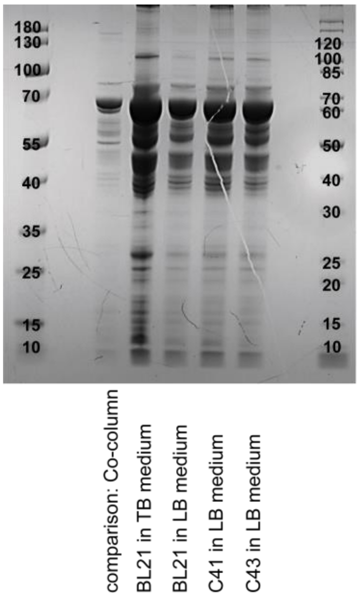

Fig. S64: SDS-PAGE analysis of fractions during His_6-MBP-McjB purification after using different expression conditions.

3.2 Optimization of His$_6$-McjC expression

3.2.1 Protein sequence of His$_6$-McjC

His$_6$-McjC

```
MGSSHHHHHH SSGLVPRGSH MEIFNVKLND TSIRIIFCKT LSAFRTENTI VMLKGKAVSN GKPVSTEEIA
RVVEEKGVSE VIENLDGVFC ILIYHFNDLL IGKSIQSGPA LFYCKKNMDI FVSDKISDIK FLNPDMTFSL
NITMAEHYLS GNRIATQESL ITGIYKVNNG EFIKFNNQLK PVLLRDEFSI TKKNNSTIDS IIDNIEMMRD
NRKIALLFSG GLDSALIFHT LKESGNKFCA YHFFSDESDD SEKYFAKEYC SKYGVDFISV NKNINFNEKL
YFNLNPNSPD EIPLIFEQTD EEGEGQPPID DDLLYLCGHG GDHIFGQNPS ELFGIDAYRS HGLMFMHKKI
VEFSNLKGKR YKDIIFSNIS AFINTSNGCS PAKQEHVSDM KLASAQFFAT DYTGKINKLT PFLHKNIIQH
YAGLPVFSLF NQHFDRYPVR YEAFQRFGSD IFWKKTKRSS SQLIFRILSG KKDELVNTIK QSGLIEILGI
NHIELESILY ENTTTRLTME LPYILNLYRL AKFIQLQSID YKG
```

Molecular weight: 60742.34 Da (without Met1)

3.2.2 Codon optimization of pET28-mcjC

The optimized gBlock for mcjC was ordered from Integrated DNA Technologies© (IDT).

```
GAAATATTTAATGTAAAACTAAACGATACCTCGATCCGCATTATTTTCTGCAAAACACTGTCTGCGTTTCGTACGG
AGAACACCATTGTGATGCTGAAGGGTAAAGCGGTCAGCAACGGTAAGCCAGTGTCGACCGAAGAAATTGCGCGTGT
TGTGGAAGAGAAAGGTGTTAGCGAGGTTATTGAGAACCTGGATGGTGTGTTCTGTATTCTCATCTACCATTTTAAC
GATTTACTGATCGGCAAAAGCATCCAGAGCGGTCCGGCTTTGTTCTATTGCAAGAAGAACATGGATATTTTCGTAA
GCGACAAAATCTCAGACATCAAGTTTCTGAATCCGGATATGACCTTTTCTCTGAACATCACGATGGCTGAACACTA
CCTGTCTGGCAATCGTATTGCCACCCAAGAGAGCCTGATCACGGGTATATATAAGGTGAATAACGGCGAATTCATC
AAGTTTAACAACCAGCTGAAACCGGTTCTGTTGCGCGACGAGTTCTCCATTACCAAAAAGAACAACAGCACCATTG
ATAGCATTATAGACAACATCGAAATGATGCGTGATAATCGTAAAATCGCGCTCCTGTTCAGCGGCGGGCTGGATAG
CGCACTGATCTTCCACACCCTGAAAGAGTCAGGAAACAAATTTTGCGCGTATCACTTCTTCTCTGACGAGAGCGAC
GACTCGGAAAAATACTTTGCTAAAGAATATTGTAGCAAATATGGTGTAGACTTTATCAGCGTTAATAAAAATATCA
ACTTTAATGAAAAGCTGTACTTCAATCTGAACCCGAATTCCCCGGACGAGATTCCGCTGATTTTCGAACAGACCGA
TGAGGAGGGCGAGGGTCAGCCGCCTATTGACGACGACCTGCTTTACTTATGCGGTCATGGTGGTGATCACATTTTC
GGTCAAAACCCATCCGAGTTGTTCGGCATCGACGCGTACCGCAGCCACGGTCTGATGTTTATGCATAAGAAGATCG
TTGAATTCTCCAACTTGAAGGGCAAGCGCTATAAAGATATCATTTTTTCTAACATCAGCGCCTTTATCAATACTTC
TAATGGCTGCTCCCCGGCAAAGCAAGAGCACGTGTCCGACATGAAATTGGCGAGCGCTCAATTTTTCGCGACCGAC
TACACCGGCAAGATCAACAAACTGACTCCGTTTCTGCATAAGAACATCATCCAACACTACGCGGGTTTACCGGTCT
TTTCCCTGTTCAACCAACACTTTGATCGTTATCCGGTGCGTTACGAAGCATTCCAGCGTTTTGGTAGCGATATTTT
CTGGAAAAAAACCAAGAGATCCAGCAGCCAGTTGATTTTCCGCATTCTGAGCGGCAAGAAGGATGAGTTAGTTAAC
ACCATTAAACAGTCTGGCCTGATCGAGATTTTGGGTATCAACCATATTGAGTTGGAAAGTATCCTTTACGAGAATA
CTACGACCCGTTTGACGATGGAGCTGCCGTATATTTTGAATCTGTATCGTCTGGCCAAGTTCATCCAACTGCAGAG
CATCGACTACAAAGGCTAATAA
```

The construct was digested with NdeI and XhoI and cloned into a pET28a vector. For that, the following two primers were ordered from Microsynth AG.

McjC_pET28_**NdeI**_fwd:

```
TTTcatatgGAAATATTTAATGTAAAACTAAACGATACCTCGATCC
```

McjC_pET28_**XhoI**_rev:

```
TTTctcgagTTATTAGCCTTTGTAGTCGATGCTCTG
```

The plasmid was amplified by transformation into *E. coli* DH5α, which was grown in LB-medium (5 mL) overnight. The plasmid was extracted from the cells and purified using QIAprep® Spin Miniprep Kit.

3.2.3 Expression in different growth medium and cell lines

For the initial pET28-mcjC (unoptimized), only Rosetta(DE3)pLysS cells were tested in LB and TB medium. The codon-optimized pET28-mcjC$_{opt}$ was expressed using Rosetta(DE3)pLysS, BL21(DE3), C41(DE3) and C43(DE3) in LB medium. Single colonies of the different *E. coli* strains transformed with the different plasmids were grown on agar plates with LB medium and kanamycin (50 µg/mL). One colony of the cell line containing the expression plasmid was picked into 2 mL LB medium supplemented with kanamycin (50 µg/mL). The cells were incubated overnight at 37 °C with agitation. The precultures were added to two 2 L Erlenmeyer flask containing LB medium or TB medium (2x500 mL) supplemented with kanamycin (48 µg/mL) (for Rosetta(DE3)pLysS: addition of chloramphenicol (33 µg/mL) and the growth was allowed to proceed at 37 °C until OD_{600} reached 0.7-0.85 (around 3.5 h at 37 °C with rigorous agitation). The cultures had been cooled down on ice for around 60 min before IPTG at a final concentration of 0.25 mM was added to induce protein expression. The cultures were further grown at 18 °C for 24 h before harvesting. The cells were recovered by centrifugation (5000 rpm, 15 min at 4 °C), weighed out and the cell pellets were flash-frozen in liquid nitrogen before storage at -80 °C.

Table S3: Mass of cells before lysis depending on growth conditions during McjC expression.

Growth condition (cell line, medium)	Amount of cells
Rosetta(DE3)pLysS in TB medium with pET28-mcjC$_{initial}$	9.9 g
Rosetta(DE3)pLysS in LB medium with pET28-mcjC$_{initial}$	7.1 g
Rosetta(DE3)pLysS in LB medium with pET28-mcjC$_{optimized}$	7.0 g
BL21(DE3) in LB medium with pET28-mcjC$_{optimized}$	6.9 g
C41(DE3) in LB medium with pET28-mcjC$_{optimized}$	7.4 g
C43(DE3) in LB medium with pET28-mcjC$_{optimized}$	7.2 g

The procedure for all cell lines and both tested media were the same. The cells were resuspended in lysis buffer [15 mL, Tris-HCl (50 mM) pH 7.8, imidazole (10 mM), NaCl (300 mM), glycerol (10%), magnesium chloride (5 mM), DNAse I (5 µg/mL), RNAse (10 µg/mL] and lysozyme (0.5 mg/mL) at 4 °C. The cells were lysed by the EmulsiFlex® High-Pressure Homogenizer circulating the cells for around 5 min. The lysate was centrifuged (18000 rpm, 30 min, 4 °C) and the supernatant was applied to a pre-equilibrated Ni-NTA (2 mL of 50% agarose slurry) column on ice, the columns was closed and shaken on ice for 1 h, before the solution was drained by gravity flow. The column was washed with resuspension buffer [5+10 mL, Tris-HCl (50 mM) pH 7.8, imidazole (10 mM), NaCl (300 mM), glycerol (10%), and magnesium chloride (5 mM)] and wash buffer [5 mL, Tris-HCl (50 mM) pH 7.8, imidazole (50 mM), NaCl (300 mM), glycerol (10%), and magnesium chloride (5 mM)]. The protein was eluted with elution buffer [8 mL, Tris-HCl (50 mM) pH 7.8, imidazole (250 mM), NaCl (300 mM), glycerol (10%), and magnesium chloride (5 mM)].

The sample was concentrated using an Amicon centrifugal device (10 kDa cutoff) and buffer exchange was performed with a PD-10 column into the storage buffer [Tris-HCl (50 mM) pH 7.5, NaCl (100 mM), glycerol (10%), and magnesium chloride (1 mM)]. The purified protein was again concentrated using an Amicon centrifugal device (30 kDa cutoff) and flash-frozen in liquid nitrogen to be stored at -80 °C.

Table S4: Amount of enzyme depending on growth conditions for McjC-expression.

Growing conditions	Volume of concentrated sample	Concentration of concentrated sample	Amount of protein
Rosetta(DE3)pLysS in TB medium with pET28-mcj$C_{initial}$	350 µL	220 µM	4.7 mg
Rosetta(DE3)pLysS in LB medium with pET28-mcj$C_{initial}$	250 µL	29 µM	0.4 mg
Rosetta(DE3)pLysS in LB medium with pET28-mcj$C_{optimized}$	250 µL	191 µM	2.9 mg
BL21(DE3) in LB medium with pET28-mcj$C_{optimized}$	300 µL	109 µM	2.0 mg
C41(DE3) in LB medium with pET28-mcj$C_{optimized}$	350 µL	222 µM	4.7 mg
C43(DE3) in LB medium with pET28-mcj$C_{optimized}$	300 µL	185 µM	3.4 mg

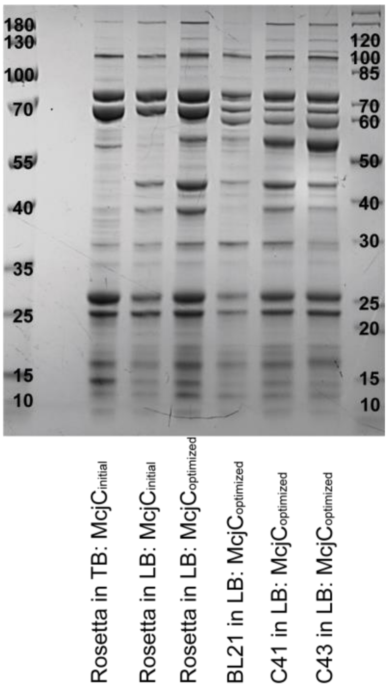

Fig. S65: SDS-PAGE analysis of fractions during His$_6$-McjC purification after using different expression conditions.

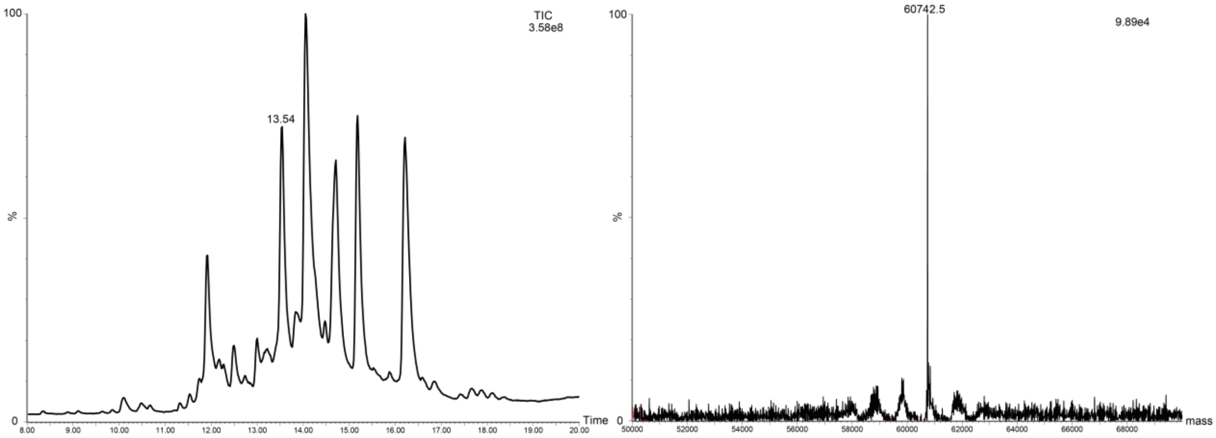

Fig. S66: LC-MS analysis of codon-optimized His$_6$-McjC expressed in *E. coli* C41(DE3). (A) TIC of the protein mixture between 8 and 20 min. (B) MS analysis of the peak at 13.54 min.

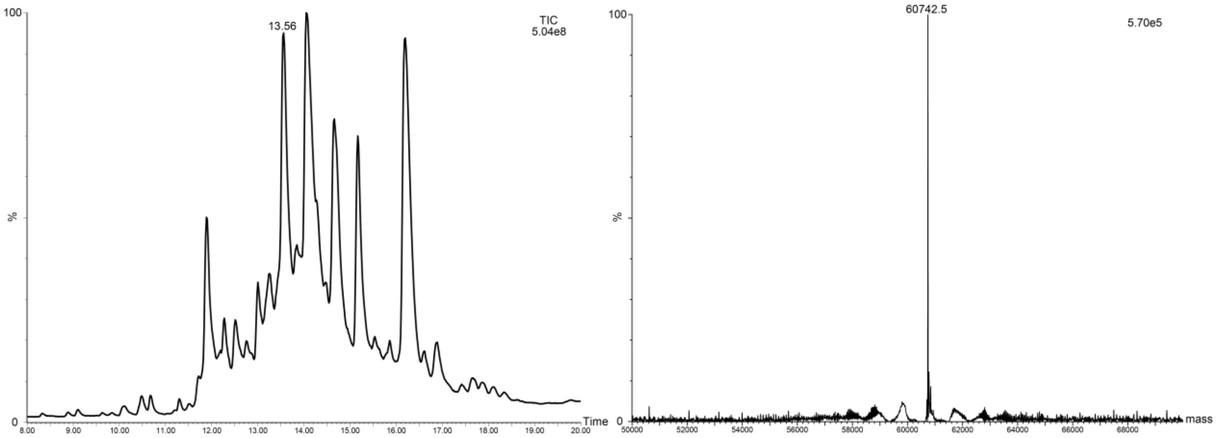

Fig. S67: LC-MS analysis of codon-optimized His$_6$-McjC expressed in *E. coli* C43(DE3). (A) TIC of the protein mixture between 8 and 20 min. (B) MS analysis of the peak at 13.56 min.

3.3 Expression of His$_6$-MBP-McjB and His$_6$-McjC for transformation of McjA derivatives

3.3.1 Expression of His$_6$-MBP-McjB and purification with Co resin

Single colonies of *E. coli* C43(DE3) transformed with pMAL-c5ePre-mcjB were grown on agar plates with LB medium and carbenicillin (50 µg/mL). One colony was picked into 50 mL TB medium supplemented with carbenicillin (50 µg/mL). The cells were incubated overnight at 37 °C with agitation. 5 mL of the precultures were added to each of the eight 2 L Erlenmeyer flasks containing TB medium (8x500 mL) supplemented with carbenicillin (48 µg/mL) and the growth was allowed to proceed at 37 °C until OD_{600} reached 0.7-0.85 (around 3.5 h at 37 °C with rigorous agitation). The cultures had been cooled down on ice for around 60 min before IPTG at a final concentration of 0.5 mM was added to induce the protein expression. The cultures were further grown at 18 °C for 21.5-22 h before harvesting. The cells were recovered by centrifugation (5000 rpm, 15 min at 4 °C), weighed out and the cell pellets were flash-frozen in liquid nitrogen before storage at -80 °C.

The cells were resuspended in lysis buffer [50 mL, Tris-HCl (50 mM) pH 7.8, imidazole (10 mM), NaCl (300 mM), glycerol (10%), magnesium chloride (5 mM), DNAse I (5 µg/mL), RNAse (10 µg/mL) and lysozyme (1 mg/mL)] at 4 °C. The cells were lysed by the EmulsiFlex® High-Pressure Homogenizer circulating the cells for around 7 min. The lysate was centrifuged (18000 rpm, 30 min, 4 °C) and the supernatant was applied to a pre-equilibrated Co resin (8 mL of 50% agarose slurry) in a 50 mL Falcon tube and shaken on ice for 1 h. The suspension was applied to the column and drained by gravity flow. The column was washed with resuspension buffer [2x10 mL, Tris-HCl (50 mM) pH 7.8, imidazole (10 mM), NaCl (300 mM), glycerol (10%), and magnesium chloride (5 mM)] and two wash buffers with different imidazole concentrations: A [10 mL, Tris-HCl (50 mM) pH 7.8, imidazole (20 mM), NaCl (300 mM), glycerol (10%), and magnesium chloride (5 mM)] and B [10 mL, Tris-HCl (50 mM) pH 7.8, imidazole (50 mM), NaCl (300 mM), glycerol (10%), and magnesium chloride (5 mM)]. The protein was eluted with elution buffer [4+8 mL, Tris-HCl (50 mM) pH 7.8, imidazole (250 mM), NaCl (300 mM), glycerol (10%), and magnesium chloride (5 mM)]. The sample was concentrated using an Amicon centrifugal device (10 kDa cutoff) and buffer exchange was performed with a PD-10 column into the storage buffer [Tris-HCl (50 mM) pH 7.5, NaCl (100 mM), glycerol (10%), and magnesium chloride (1 mM)]. The purified protein was again concentrated using an Amicon centrifugal device (30 kDa cutoff) and flash-frozen in liquid nitrogen to be stored at -80 °C.

Table S5: Outcome of 4 L Expression of His6-MBP-McjB after Co-resin purification.

Volume of concentrated sample	Concentration of sample	Amount of protein
180 μL	164 μM	2.0 mg

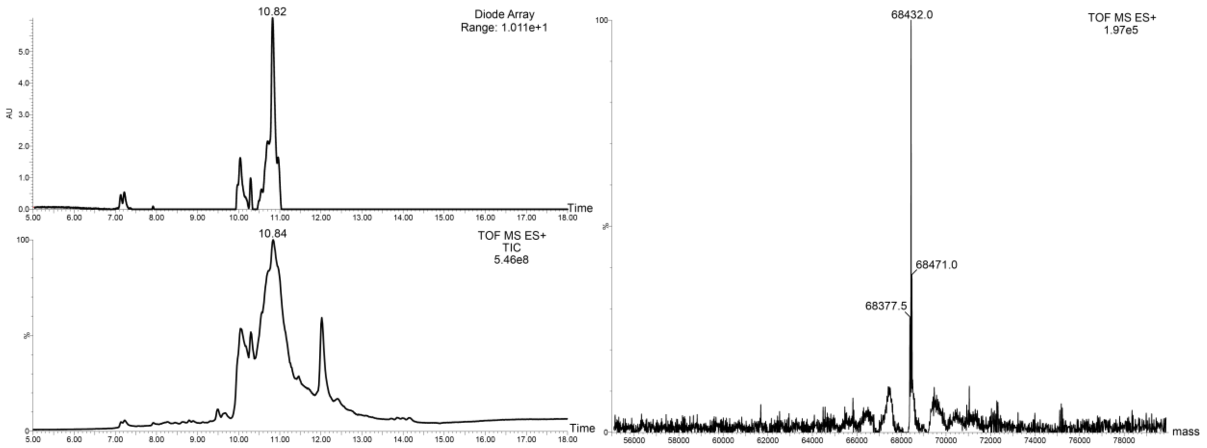

Fig. S68: LC-MS analysis of His6-MBP-McjB expressed in *E. coli* C43(DE3) in 4 L TB medium and purification via Co-resin. (A) UV and TIC of the protein mixture between 5 and 18 min. (B) MS analysis of the peak at 10.84 min.

3.3.2 Expression of His6-McjC and purification with Co resin

Single colonies of *E. coli* C43(DE3) transformed with the codon-optimized pET28-mcjC$_{opt}$ were grown on agar plates with LB medium and kanamycin (50 μg/mL). One colony was picked into 50 mL LB medium supplemented with kanamycin (50 μg/mL). The cells were incubated overnight at 37 °C with agitation. 5 mL of the precultures were added to each of the eight 2 L Erlenmeyer flasks containing TB medium (8x500 mL) supplemented with kanamycin (48 μg/mL) and the growth was allowed to proceed at 37 °C until OD$_{600}$ reached 0.7-0.85 (around 3.5 h at 37 °C with rigorous agitation). The cultures had been cooled down on ice for around 60 min before IPTG at a final concentration of 0.25 mM was added to induce protein expression. The cultures were further grown at 16 °C for 22-22.5 h before harvesting. The cells were recovered by centrifugation (5000 rpm, 15 min at 4 °C), weighed out and the cell pellets were flash-frozen in liquid nitrogen before storage at -80 °C.

The cells were resuspended in lysis buffer [90 mL, Tris-HCl (50 mM) pH 7.8, imidazole (10 mM), NaCl (300 mM), glycerol (10%), magnesium chloride (5 mM), DNAse I (10 μg/mL), RNAse (10 μg/mL)] at 4 °C. The cells were lysed by the EmulsiFlex® High-Pressure Homogenizer circulating the cells for around 7 min. The lysate was centrifuged (18000 rpm, 30 min, 4 °C) and the supernatant was applied to a pre-equilibrated Co-resin (8 mL of 50% agarose slurry) divided in two 50 mL Falcon tube and shaken on ice for 1 h. The suspension was applied to the column and drained by gravity flow. The column was washed with resuspension buffer [2x10 mL, Tris-HCl (50 mM) pH 7.8, imidazole (10 mM), NaCl (300 mM), glycerol (10%), and magnesium chloride (5 mM)] and two wash buffers with different imidazole concentrations: A [10 mL, Tris-HCl (50 mM) pH 7.8, imidazole (20 mM), NaCl (300 mM), glycerol (10%), and magnesium chloride (5 mM)] and B [10 mL, Tris-HCl (50 mM) pH 7.8, imidazole (50 mM), NaCl (300 mM), glycerol (10%), and magnesium chloride (5 mM)]. The protein was eluted with elution buffer [4+8 mL, Tris-HCl (50 mM) pH 7.8, imidazole (250 mM), NaCl (300 mM), glycerol (10%), and magnesium chloride (5 mM)]. The sample was concentrated using an Amicon centrifugal device (10 kDa cutoff) and buffer exchange was

performed with a PD-10 column into the storage buffer [Tris-HCl (50 mM) pH 7.5, NaCl (100 mM), glycerol (10%), and magnesium chloride (1 mM)]. The purified protein was again concentrated using an Amicon centrifugal device (30 kDa cutoff) and flash-frozen in liquid nitrogen to be stored at -80 °C.

Table S6: Outcome of 4 L Expression of His$_6$-McjC after Co-resin purification.

Volume of concentrated sample	Concentration of sample	Amount of protein
270 μL	135 μM	2.2 mg

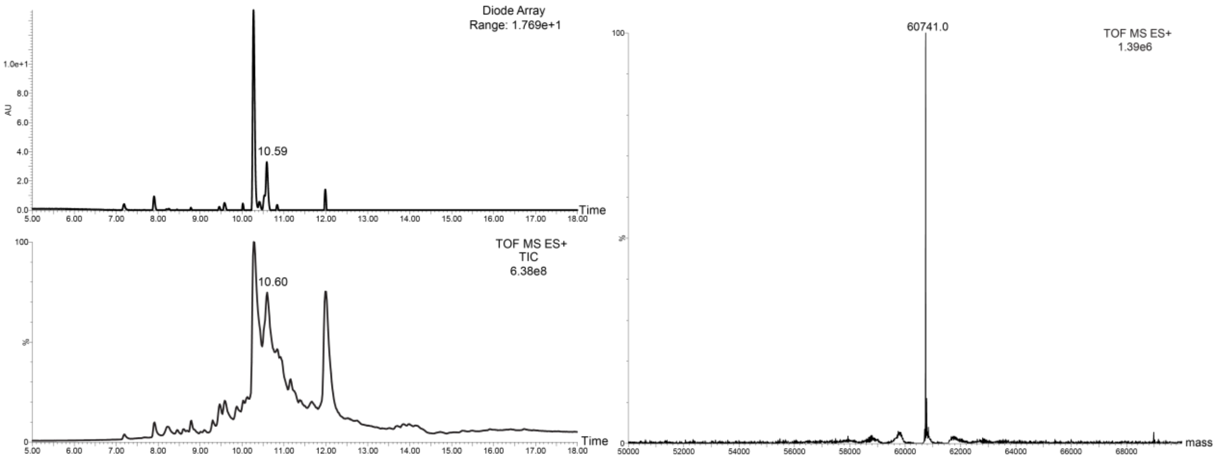

Fig. S69: LC-MS analysis of codon-optimized His$_6$-McjC expressed in E. coli C43(DE3) in 4 L TB medium and purification via Co-resin. (A) UV and TIC of the protein mixture between 5 and 18 min. (B) MS analysis of the peak at 10.60 min.

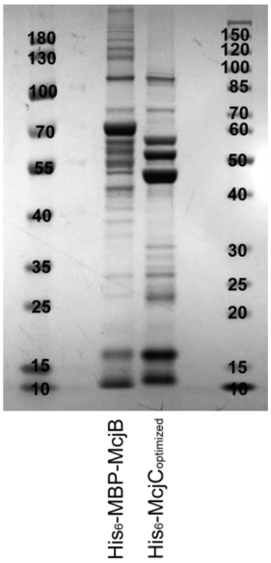

Fig. S70: SDS-PAGE analysis of big batch expression (4 L) in TB medium and Co resin purification.

3.3.3 Expression of His_6-MBP-McjB and purification with Ni-NTA resin

Single colonies of the *E. coli* C43(DE3) transformed with pMAL-c5ePre-mcjB were grown on agar plates with LB medium and carbenicillin (50 µg/mL). One colony was picked into 60 mL TB medium supplemented with carbenicillin (50 µg/mL). The cells were incubated overnight at 37 °C with agitation. 4.5 mL of the precultures were added to each of the twelve 2 L Erlenmeyer flask containing TB medium (12x500 mL) supplemented with carbenicillin (48 µg/mL) and the growth was allowed to proceed at 37 °C until OD_{600} reached 0.6-0.7 (around 3.5 h at 37 °C with rigorous agitation). The cultures had been cooled down on ice for around 45 min before IPTG at a final concentration of 0.1 mM was added to induce protein expression. The cultures were further grown at 18 °C for 20-21 h before harvesting. The cells were recovered by centrifugation (5000 rpm, 15 min at 4 °C), weighed out and the cell pellets were flash-frozen in liquid nitrogen before storage at -80 °C. The mass of cells was 38.3 g.

The cells were resuspended in lysis buffer [3x30 mL, Tris-HCl (50 mM) pH 7.8, imidazole (10 mM), NaCl (300 mM), glycerol (10%), magnesium chloride (5 mM), DNAse I (5 µg/mL), RNAse (10 µg/mL) and lysozyme (0.5 mg/mL)] at 4 °C. The cells were lysed by the EmulsiFlex® High-Pressure Homogenizer circulating the cells for around 12 min. The lysate was centrifuged (18000 rpm, 30 min, 4 °C) and the supernatant was applied to a pre-equilibrated Ni-NTA (10 mL of 50% agarose slurry) in three 50 mL Falcon tubes and shaken on ice for 1 h. The resin was centrifuged down, the supernatant was discarded and the resin was suspended in resuspension buffer [50 mL, Tris-HCl (50 mM) pH 7.8, imidazole (10 mM), NaCl (300 mM), glycerol (10%), and magnesium chloride (5 mM)]. The suspension was divided into 5 equal parts, applied to 5 columns, and drained by gravity flow. Each column was washed with resuspension buffer [10+5 mL, Tris-HCl (50 mM) pH 7.8, imidazole (10 mM), NaCl (300 mM), glycerol (10%), and magnesium chloride (5 mM)] and wash buffer [5 mL, Tris-HCl (50 mM) pH 7.8, imidazole (50 mM), NaCl (300 mM), glycerol (10%), and magnesium chloride (5 mM)]. The protein was eluted with elution buffer [4+4 mL, Tris-HCl (50 mM) pH 7.8, imidazole (250 mM), NaCl (300 mM), glycerol (10%), and magnesium chloride (5 mM)]. The samples were combined and concentrated using an Amicon centrifugal device (10 kDa cutoff) and buffer exchange was performed with a PD-10 column into the storage buffer [Tris-HCl (50 mM) pH 7.5, NaCl (100 mM), glycerol (10%), and magnesium chloride (1 mM)]. The purified protein was again concentrated using an Amicon centrifugal device (30 kDa cutoff) and flash-frozen in liquid nitrogen to be stored at -80 °C.

Table S7: Outcome of 6 L Expression of His_6-MBP-McjB after Ni-NTA purification.

Volume of concentrated sample	Concentration of sample	Amount of protein
780 µL	867 µM	46 mg

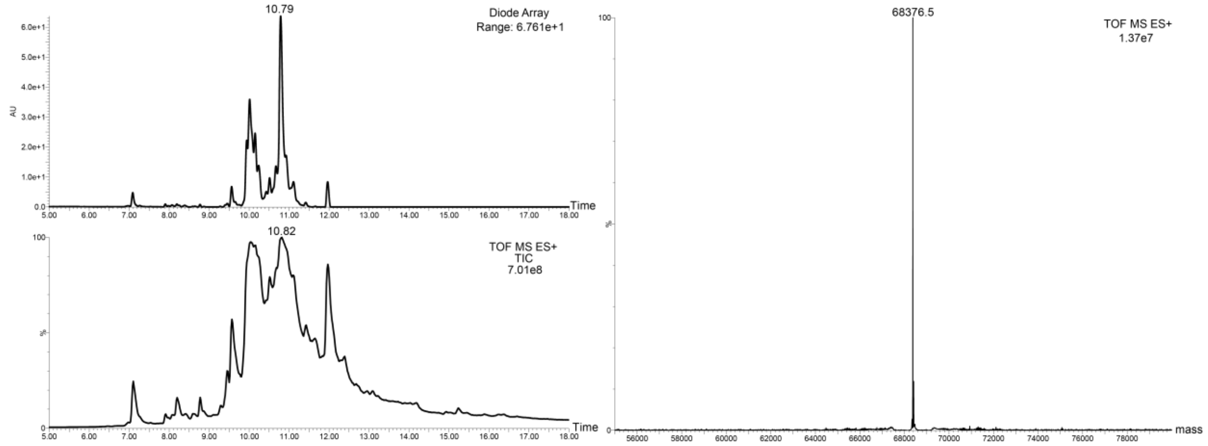

Fig. S71: LC-MS analysis of His6-MBP-McjB expressed in *E. coli* C43(DE3) in 6 L TB medium and purification via Ni-NTA-resin. (A) UV and TIC of the protein mixture between 5 and 18 min. (B) MS analysis of the peak at 10.82 min.

3.3.4 Expression of His6-McjC and purification with Ni-NTA resin

Single colonies of *E. coli* C43(DE3) transformed with the codon-optimized pET28-mcjC_{opt} were grown on agar plates with LB medium and kanamycin (50 µg/mL). One colony was picked into 60 mL TB medium supplemented with kanamycin (50 µg/mL). The cells were incubated overnight at 37 °C with agitation. 5 mL of the precultures were added to each of the twelve 2 L Erlenmeyer flasks containing TB medium (12x500 mL) supplemented with kanamycin (48 µg/mL) and the growth was allowed to proceed at 37 °C until OD_{600} reached 0.75-0.85 (around 3.5 h at 37 °C with rigorous agitation). The cultures had been cooled down on ice for around 60 min before IPTG at a final concentration of 0.1 mM was added to induce protein expression. The cultures were further grown at 18 °C for 20-21 h before harvesting. The cells were recovered by centrifugation (5000 rpm, 15 min at 4 °C), weighed out and the cell pellets were flash-frozen in liquid nitrogen before storage at -80 °C. The mass of cells was 48.8 g.

The cells were resuspended in lysis buffer [3x40 mL, Tris-HCl (50 mM) pH 7.8, imidazole (10 mM), NaCl (300 mM), glycerol (10%), magnesium chloride (5 mM), DNAse I (5 µg/mL), RNAse (10 µg/mL) and lysozyme (1 mg/mL)] at 4 °C. The cells were lysed by the EmulsiFlex® High-Pressure Homogenizer circulating the cells for around 15 min. The lysate was centrifuged (18000 rpm, 30 min, 4 °C) and the supernatant was applied to a pre-equilibrated Ni-NTA (10 mL of 50% agarose slurry) in five 50 mL Falcon tubes and shaken on ice for 1 h. The resin was centrifuged down, the supernatant was discarded and the resin was suspended in resuspension buffer [each tube 5 mL, Tris-HCl (50 mM) pH 7.8, imidazole (10 mM), NaCl (300 mM), glycerol (10%), and magnesium chloride (5 mM)] The suspension was divided into 5 equal parts, applied to 5 columns, and drained by gravity flow. Each column was washed with resuspension buffer [10 mL, Tris-HCl (50 mM) pH 7.8, imidazole (10 mM), NaCl (300 mM), glycerol (10%), and magnesium chloride (5 mM)] and wash buffer [10 mL, Tris-HCl (50 mM) pH 7.8, imidazole (50 mM), NaCl (300 mM), glycerol (10%), and magnesium chloride (5 mM)]. The protein was eluted with elution buffer [4+4 mL, Tris-HCl (50 mM) pH 7.8, imidazole (250 mM), NaCl (300 mM), glycerol (10%), and magnesium chloride (5 mM)]. The samples were combined and concentrated using an Amicon centrifugal device (10 kDa cutoff) and buffer exchange was performed with a PD-10 column into the storage buffer [Tris-HCl (50 mM) pH 7.5, NaCl (100 mM), glycerol (10%), and magnesium chloride (1 mM)]. The purified protein was again concentrated using an Amicon centrifugal device (30 kDa cutoff) and flash-frozen in liquid nitrogen to be stored at -80 °C.

Table S8: Outcome of 6 L Expression of His6-McjC after Ni-NTA purification.

Volume of concentrated sample	Concentration of sample	Amount of protein
620 µL	403 µM	15 mg

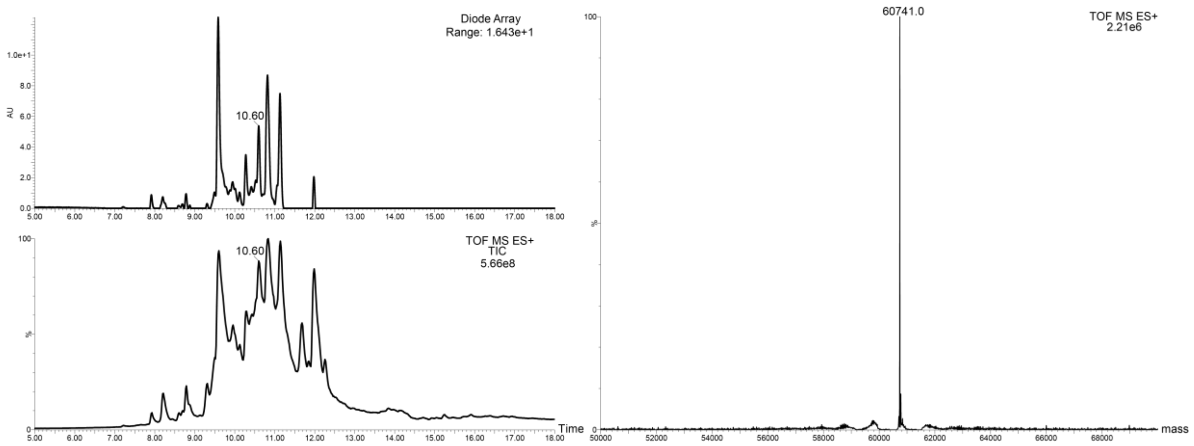

Fig. S72: LC-MS analysis of codon-optimized His6-McjC expressed in E. coli C43(DE3) in 6 L TB medium and purification via Ni-NTA-resin. (A) UV and TIC of the protein mixture between 5 and 18 min. (B) MS analysis of the peak at 10.60 min.

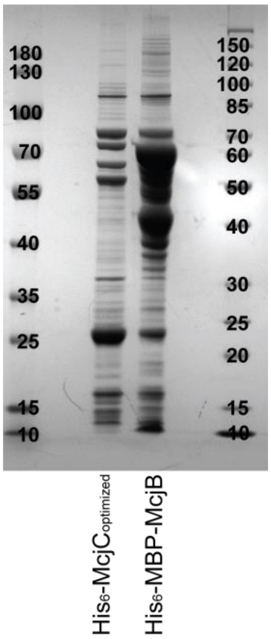

Fig. S73: SDS-PAGE analysis of big batch expression (6 L) in TB medium and Ni-NTA resin purification.

4. Transformation of precursor peptides and analysis thereof

4.1 Direct expression of wild-type MccJ25 (**lasso**) in *E. coli*

The pTUC100 plasmid was incorporated into *E. coli* MC4100 by electroporation and portions of 1 mL of an overnight grown culture were flash-frozen. One portion was divided and added into three Falcon tubes containing 5 mL M63 medium supplemented with chloramphenicol (34 µg/mL). The cells were incubated for three days at 37 °C with agitation. The cells were separated by centrifugation (5000 g, 15 min at 4 °C), and the supernatant was purified by performing solid-phase extraction using Sep-Pak® Plus C8 Cartridges. The peptide was purified using semi-preparative HPLC as specified in the general procedure 1.9. Fractions were analyzed by LC-HR-ESI-MS, combined and lyophilized to obtain 0.31 mg (>95% purity (C18) and 85% purity (C8) by UHPLC) of the desired peptide.

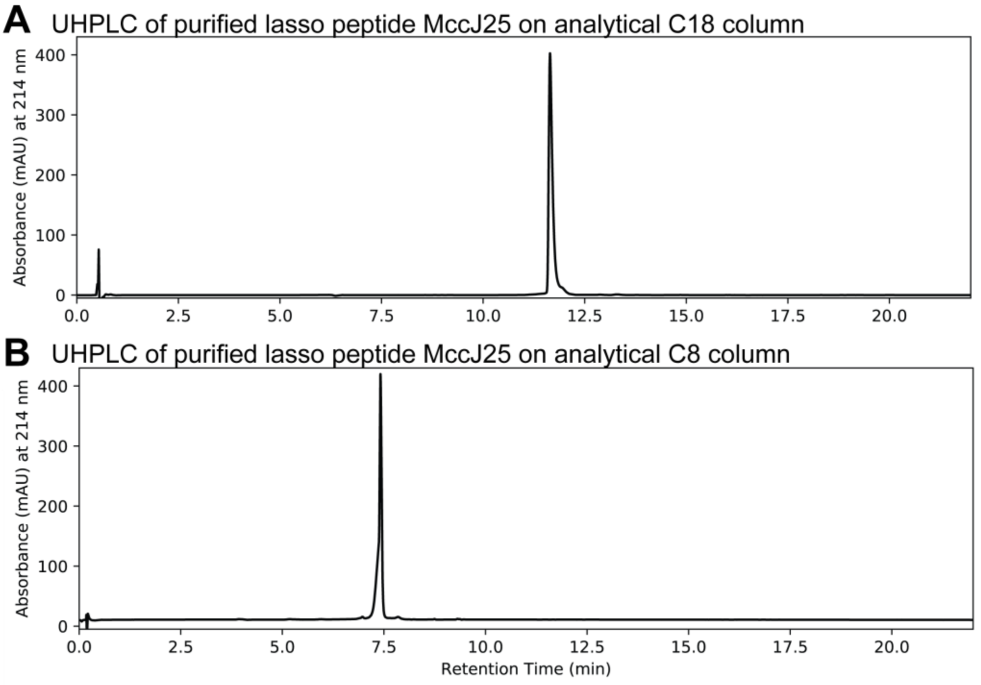

Fig. S74: UHPLC profile of expressed wild-type lasso peptide MccJ25: (A) purified sample on analytical C18 column (>95% purity, R_t = 11.65 min); (B) purified sample on analytical C8 column (85% purity, R_t = 7.42 min).

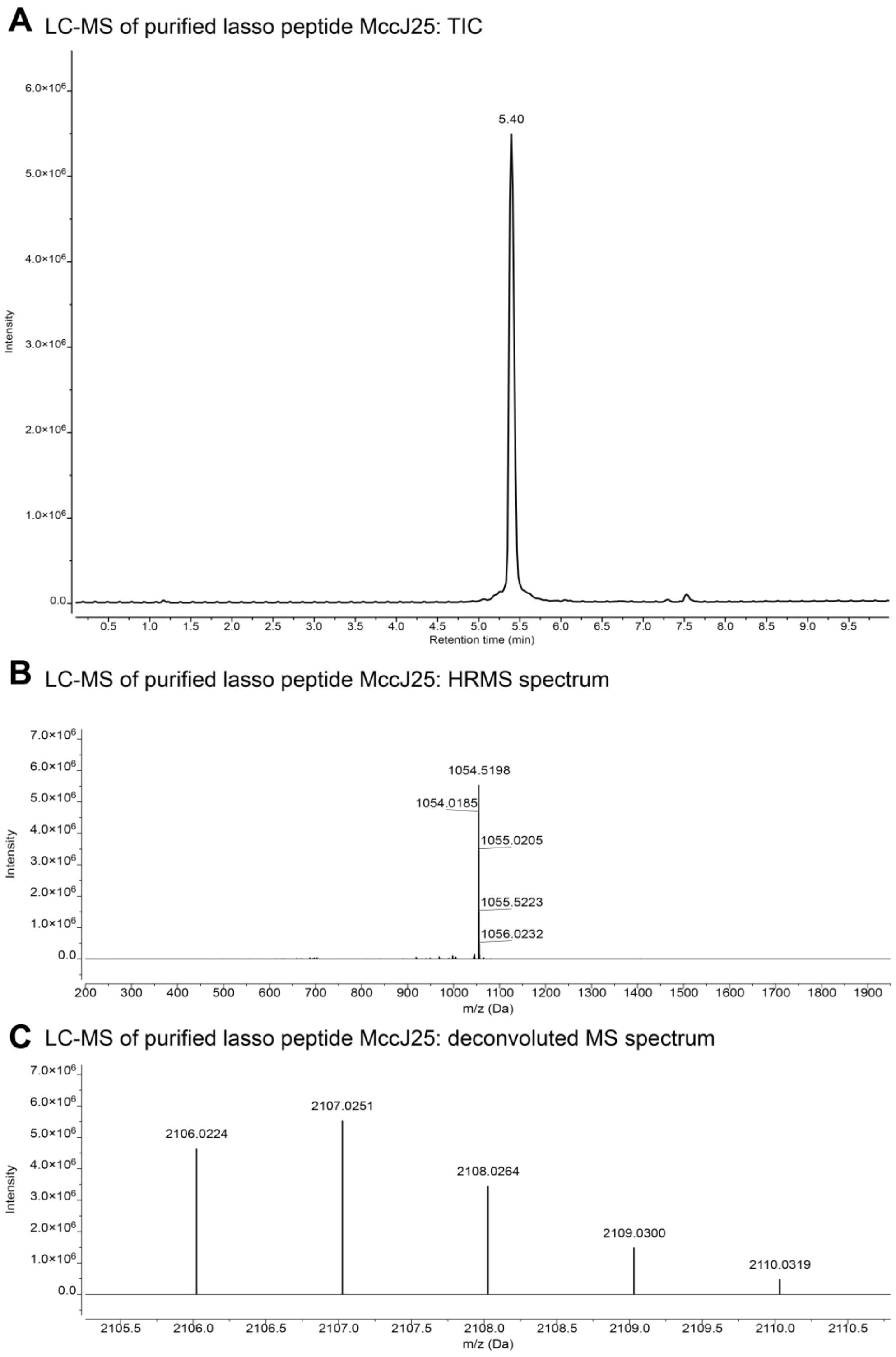

Fig. S75: LC-MS analysis of expressed wild-type lasso peptide MccJ25: (A) Total ion chromatogram (TIC). (B) MS spectrum. (C) deconvoluted MS spectrum: Monoisotopic mass (ESI+): calc. [$C_{101}H_{139}N_{23}O_{27}$]: 2106.0211, found: 2106.0224; Average mass calc. [$C_{101}H_{139}N_{23}O_{27}$]: 2107.3570.

4.2 *In vitro* transformation of precursor peptides to lasso peptides

The reaction mixture contains a total volume of 300 µL. The reaction contains 50 mM Tris-HCl (pH 8.0), 5 mM $MgCl_2$, 1 mM ATP, 5 mM TCEP, 5.0 µM McjA-derivative, 2.5 µM His_6-MBP-McjB and 2.5 µM His_6-McjC. The reactions were performed at 30 °C for 3 h. Afterwards, 10 µL formic acid (0.1 M) and 300 µL MeOH were added to stop the reaction by enzyme precipitation. The reaction mixture was centrifuged (12000 g, 5 min, 4 °C). 100 µL of the supernatant was analyzed by LC-IM-MS. The residual supernatant was transferred to another tube and concentrated using a SpeedVac vacuum concentrator at 30 °C.

Remark: The concentrations added were estimated on the whole protein solution as determined in Table S5 and Table S6 via BCA assay. Therefore, the correct ratio of the precursor to the enzyme solutions is much higher than 2:1:1. However, no full conversion of the precursor was expected (also in alignment with previous investigations[3]) during the reactions.

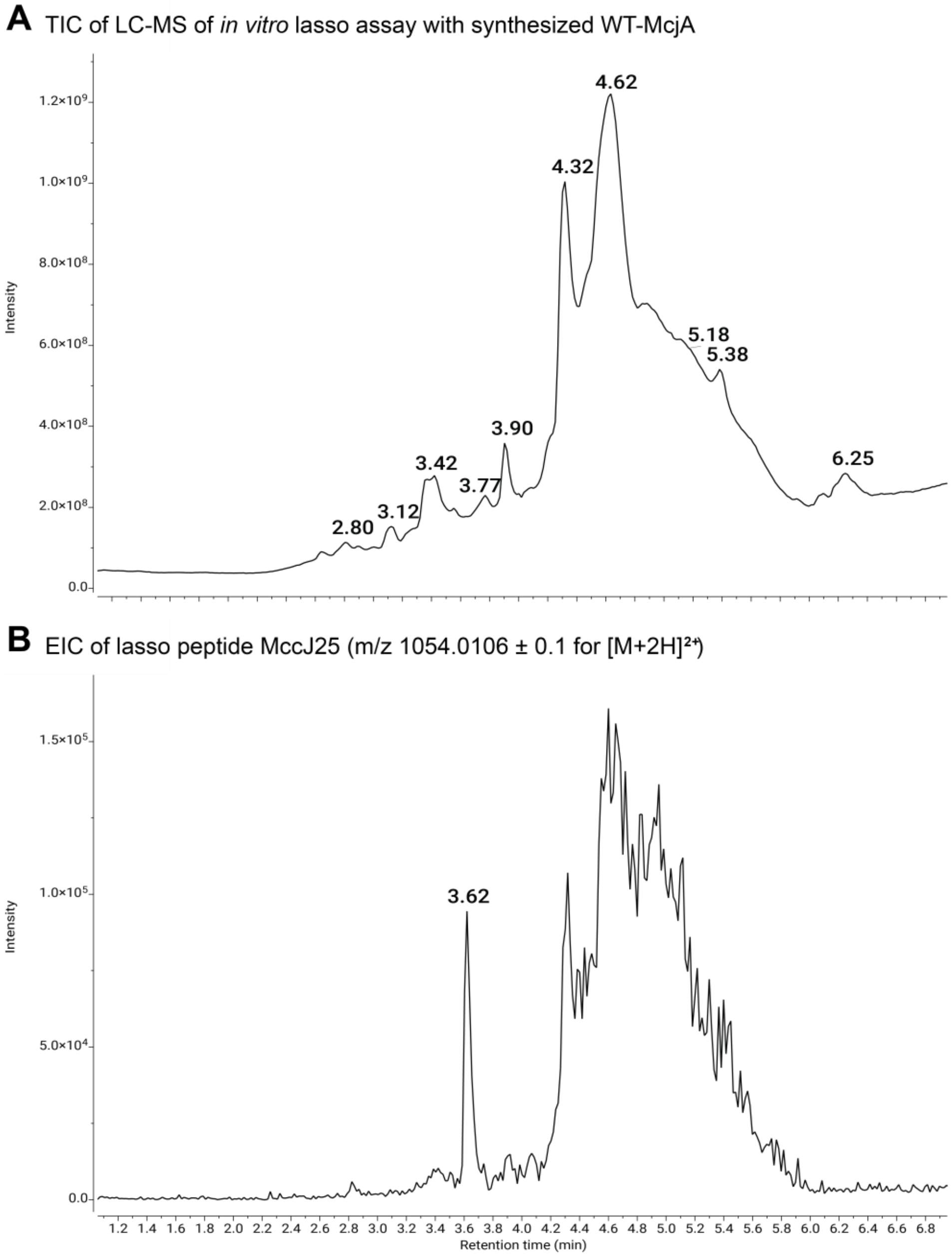

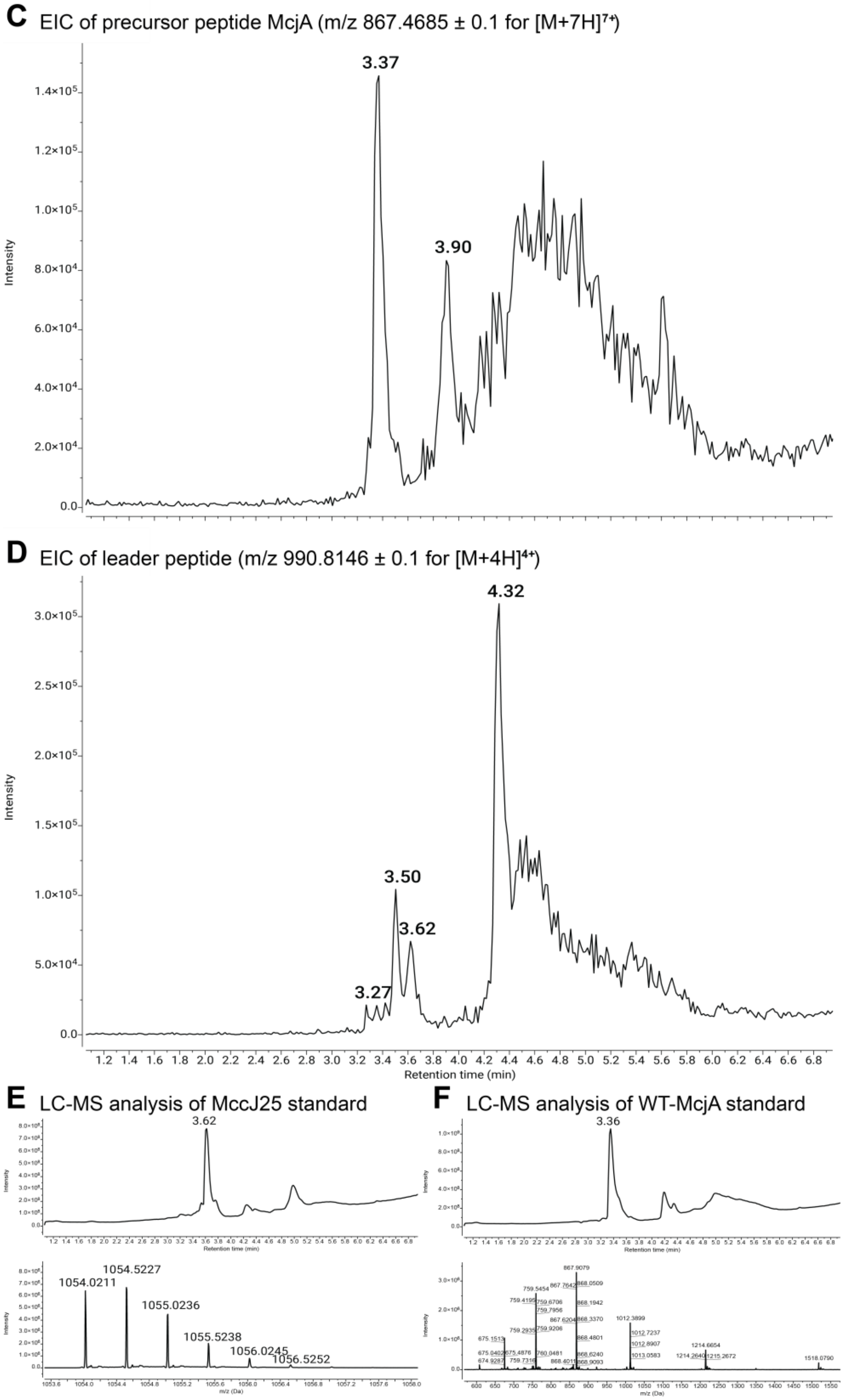

Fig. S76: LC-MS-analysis (Agilent) of the lasso transformation assay: (A) TIC analysis. (B) Extracted ion count (EIC) of MccJ25 (monoisotopic mass: 2106.0211 Da). (C) Extracted ion count (EIC) of McjA (monoisotopic mass: 6065.2796 Da). (D) Extracted ion count (EIC) of leader peptide (monoisotopic mass: 3959.2585 Da). (E) TIC of MccJ25 standard. (E) TIC of McjA standard.

4.3 Antimicrobial activity assay against Salmonella strain

The antimicrobial activity assay of MccJ25-derivatives was performed against the indicative strain *Salmonella enterica* serotype Enteritidis.

4.3.1 Preparation of M63 agar

For **M63 agar plates (20 g agar/L):** KH_2PO_4 (3 g/L), K_2HPO_4 (7 g/L), $(NH_4)_2SO_4$ (2 g/L), Casamino acids (1 g/L) were dissolved in 900 mL water. Agar (20 g/L) was added, and the suspension was filled up to 1 L and autoclaved (121 °C for 20 min). The following stock solutions (sterile filtered on 0.22 μm filter) were added before use: 1 mL $MgSO_4$ (20% m/v), 200 μL vitamin B1 (5 g/L), and 10 mL glucose (20% m/v).

For **M63 soft agar (6.5 g/L)**: it was prepared the same way as M63 agar except for the agar concentration.

4.3.2 General procedure of spot-on-lawn assay

The indicative strain is freshly streaked on a LB agar plate and incubated at 37 °C overnight. From one colony, a preculture was grown in 5 mL LB medium at 37 °C. The preculture is grown until OD_{600} reaches 0.2-0.6. The preculture is inoculated into prior melted M63 soft agar to have a final concentration of OD_{600}=0.01. The M63 soft-agar is poured onto the pre-prepared M63-agar plate. Once the soft agar is solidified, the peptide solutions are directly spotted on the soft-agar. The plate was incubated at 37 °C overnight. The plates were inspected for bacterial growth and possible halos were measured.

4.3.3 Preparation of peptide solutions and results of antimicrobial test

The dried reaction mixtures were dissolved in 30 μL MeOH resulting in a concentration of 41.7 μM (around 90 μg/mL depending on the molecular weight of the resulting lasso peptide) if full conversion were achieved. 5 μL of the peptide were spotted on the agar plate. As controls, 5 μL MeOH (negative control) and 2 μL of 1 mg/mL (475 μM) MccJ25 standard (positive control) were spotted.

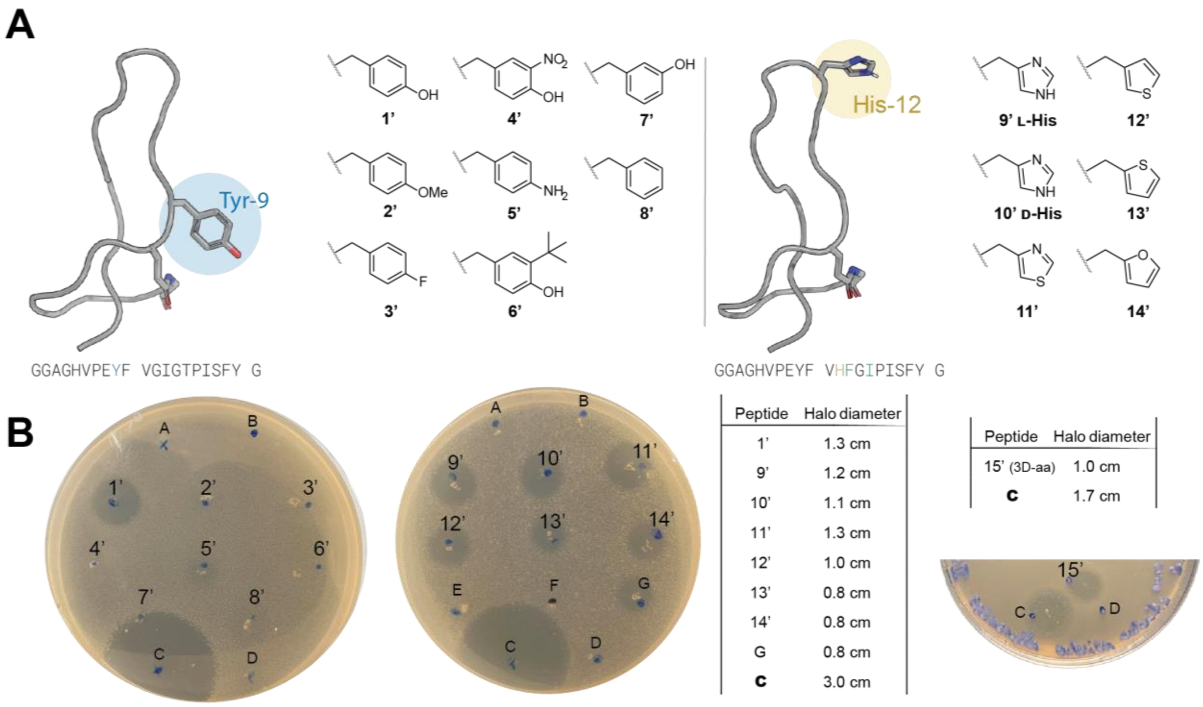

Peptide	Halo diameter
1'	1.3 cm
9'	1.2 cm
10'	1.1 cm
11'	1.3 cm
12'	1.0 cm
13'	0.8 cm
14'	0.8 cm
G	0.8 cm
**C**	3.0 cm

Peptide	Halo diameter
15' (3D-aa)	1.0 cm
**C**	1.7 cm

Controls: **A:** McjA, handled as maturation assay; **B:** McjB+McjC, handled as maturation assay; **C:** MccJ25: 2 µL (1 mg/mL, expressed+purified); **D:** 5 µL MeOH

Samples: Transformation of **E:** McyA to MccY (not active); **F:** McjA-leader with McyA-core to MccY (not active); **G:** McyA-leader with McjA-core to MccJ25 (active)

Fig. S77: Antimicrobial activity test using spot-on-lawn assays with dried maturation assay dissolved in 30 µL MeOH: (A) Peptides analyzed after maturation assay. (B) Photos of spot-on-lawn assays and measured halo diameters to evaluate trends in activity. The peptide with three D-amino acids was analyzed on a different date, therefore, diameter of control C (MccJ25).

For the Tyr9 derivatives, the test was repeated. Therefore, the solution was concentrated again and re-dissolved in 15 µL MeOH (69.5 µM, around 150 µg/mL). 8 µL of the peptide solutions were spotted on the agar plate. As controls, 8 µL MeOH (negative control) and 2 µL of 1 mg/mL (475 µM) MccJ25 standard (positive control) were spotted.

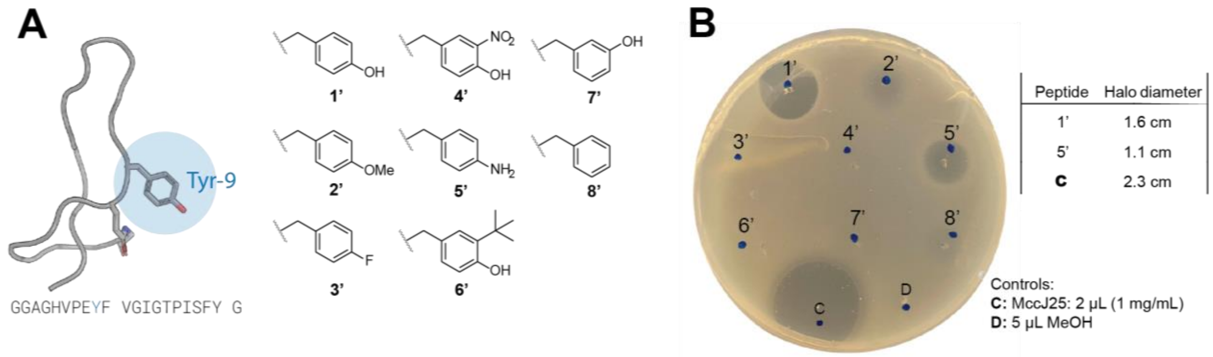

Peptide	Halo diameter
1'	1.6 cm
5'	1.1 cm
**C**	2.3 cm

Fig. S78: Antimicrobial activity test using spot-on-lawn assays with dried maturation assay after concentrating the samples again: (A) Peptides with Tyr-9 modifications analyzed after re-concentrating the maturation assay. (B) Photos of spot-on-lawn assays and measured halo diameters to evaluate trends in activity.

The branched-cyclic peptides were weighed out and dissolved in MeOH, resulting in a concentration of 200 µM. 5 µL of the peptide were spotted on the agar plate. As controls, 5 µL MeOH (negative control) and 2 µL of 1 mg/mL (475 µM) MccJ25 standard (positive control) were spotted.

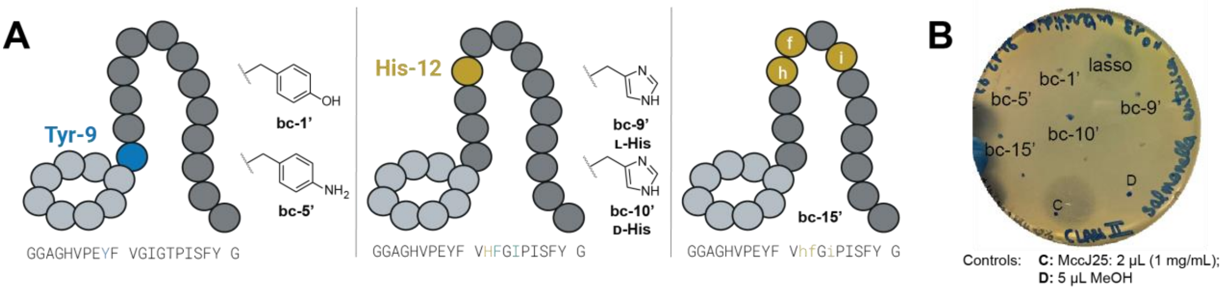

Fig. S79: **Antimicrobial activity test using spot-on-lawn assays with branched-cyclic peptide samples dissolved in MeOH (final concentration 200 µM):** (A) Peptides with non-canonical amino acid modifications analyzed. (B) Photos of spot-on-lawn assay having MccJ25 (lasso peptide) as control (5 µL at 200 µM concentration and 2 µL 1 mg/mL).

4.4 Results of Ion-Mobility Mass-Spectrometry (IM-MS) coupled to LC

Table S9: CCS-values [Å^2] of the peak with the smallest CCS value in the mobilogram.

	retention time [min]	50 V			100 V			150 V			mean	std.
**lasso**	3.70–3.80	469.3	469.3	469.3	469.2	469.4	469.6	469.5	469.4	469.8	469.4	0.1_9
**Tyr-9 derivatives**												
**1'**	3.60–3.90	469.6	469.6	469.9	469.8	469.7	469.9	470.0	470.0	469.8	469.8	0.1_5
**2'**	4.20–4.30	472.9	473.1	473.6	473.0	473.2	473.1	473.1	473.3	473.2	473.2	0.2_0
**3'**	4.30–4.60	469.1	469.0	469.2	469.2	469.4	469.2	469.2	469.3	469.1	469.2	0.1_1
**4'**	4.20–4.30	470.2	470.1	470.2	470.3	470.1	470.5	470.5	470.4	470.4	470.3	0.1_4
**5'**	2.96–3.05	469.3	468.7	468.9	468.9	469.3	469.2	468.9	469.2	469.6	469.1	0.3_0
**6'**	4.80–4.90	481.3	481.2	481.1	481.1	480.9	481.1	481.5	480.5	481.4	481.3	0.2_9
**7'**	3.90–4.00	467.2	467.4	467.4	467.3	467.1	467.3	467.6	467.2	467.4	467.3	0.1_5
**8'**	4.30–4.50	468.1	468.4	467.9	467.9	467.9	468.1	468.0	468.0	467.6	468.0	0.2_1
**bc-1'**	3.80–3.90	489.7	489.4	489.4	489.2	489.6	489.6	489.3	489.2	489.4	489.4	0.1_8
**bc-5'**	3.10–3.40	469.8	470.0	469.9	469.2	469.7	470.2	469.8	469.7	470.2	469.8	0.3_0
**bc-6'**	5.00–5.20	497.0	497.3	497.5	497.4	497.3	497.6	497.3	497.3	497.3	497.3	0.1_7
**His-12 derivatives**												
**9'**	4.00–4.10	501.5	502.1	502.9	501.7	501.7	501.4	501.3	501.7	501.1	501.7	0.5_3
**10'**	3.80–3.90	488.0	488.2	488.1	488.0	488.3	488.1	488.1	488.3	488.0	488.1	0.1_2
**11'**	5.10–5.20	497.0	496.6	496.5	496.8	496.5	496.9	496.5	496.3	496.6	496.6	0.2_2
**12'**	5.55–5.60	501.2	501.9	501.7	501.3	501.7	502.1	501.2	501.2	500.7	501.4	0.4_4
**13'**	5.55–5.59	501.6	501.5	501.6	n.f.	n.f.	501.3	n.f.	501.1	501.1	501.4	0.2_3

		50 V			100 V			150 V			mean	std.
**14’**	5.44–5.52	497.8	499.6	499.0	499.3	499.5	499.0	498.6	498.5	499.4	499.1	0.4_1
**bc-9’**	3.80–3.90	502.9	503.0	502.9	502.8	503.1	502.7	503.1	503.2	503.0	503.0	0.1_6
**bc-10’**	4.30–4.40	503.8	504.0	503.8	503.9	503.7	503.7	504.0	504.2	503.9	503.9	0.1_6
**Derivative with three D-amino acids** The peptide from the assay had peaks at two different retention times. Therefore, we have one CCS value for each peak.												
**15’-A**	3.80–4.00	504.1	504.3	504.7	503.6	504.1	504.0	504.6	504.6	504.1	504.2	0.3_5
**15’-B**	4.20–4.30	498.3	498.5	498.4	498.0	498.3	498.4	498.0	498.2	498.0	498.2	0.1_9
**bc-15’**	3.83–3.90	509.9	510.0	510.0	510.1	510.1	510.0	510.3	510.4	510.3	510.1	0.1_7
**Derivatives with backbone N-methylation** The peptide showed two peaks in the mobilogram: **max**: peak with maximal intensity, **small**: smallest CCS value												
**16’ (1Me)**	4.07–4.13	473.6	473.5	473.6	473.5	473.6	473.5	474.0	473.5	474.1	473.7	0.2_3
**bc-16’**	3.91–3.97	493.1	493.0	493.2	493.0	493.0	493.0	492.6	492.7	492.9	492.9	0.1_9
**17’small (2Me)**	4.27–4.32	479.4	479.2	479.3	n.f.	479.3	479.5	479.5	479.3	479.8	479.4	0.1_9
**17’max (2Me)**		484.2	484.1	484.2	484.5	483.8	483.8	484.5	486.0	484.2	484.4	0.6_6
**bc-17’**	4.01–4.10	497.4	497.4	497.3	497.7	497.3	497.6	497.8	497.8	497.4	497.5	0.2_0
**Derivative with McyA leader and McjA core (formation of MccJ25)**												
**18’**	3.96–4.03	469.6	469.8	469.0	469.6	469.8	469.5	469.3	469.4	470.5	469.6	0.4_2

Number’ is the analysis of lasso peptide after maturation. **bc-number’** corresponds to the branched-cyclic derivative.

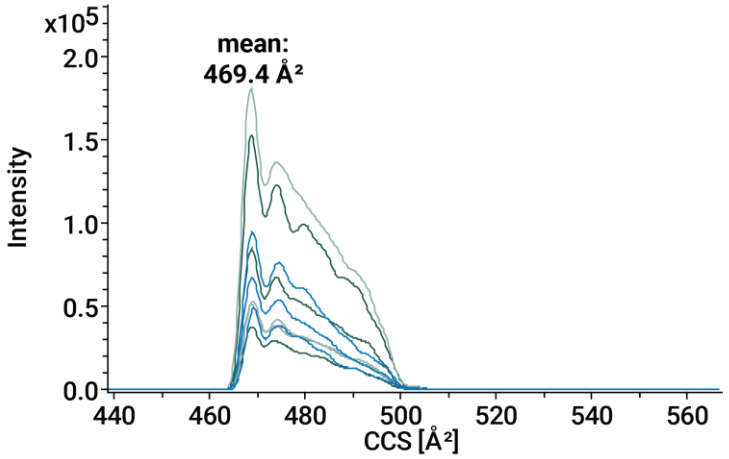

Fig. S80: LC-TIMS-MS mobilogram of the monoisotopic $[M+2H]^{2+}$ ion (m/z 1054.0204 ± 0.1) of MccJ25 lasso peptide standard (lasso). Δ6 voltage set to 150 V (blue), 100 V (light green), and 50 V (dark green).

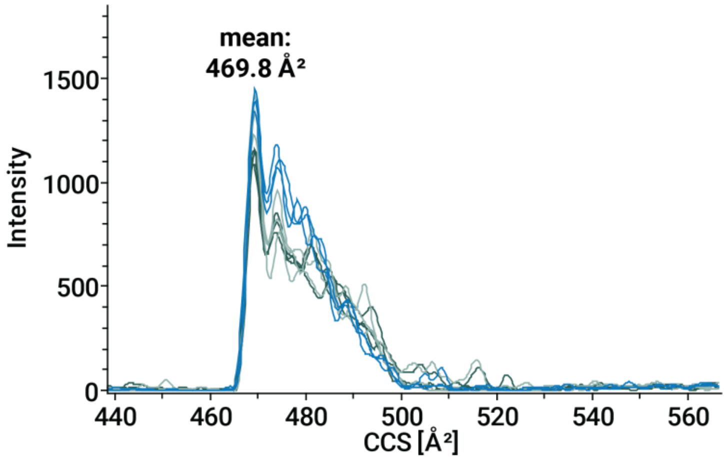

Fig. S81: LC-TIMS-MS mobilogram of the monoisotopic [M+2H]$^{2+}$ ion (m/z 1054.0186 ± 0.1) after the maturation assay of WT-McjA (1'). Δ6 voltage set to 150 V (blue), 100 V (light green), and 50 V (dark green).

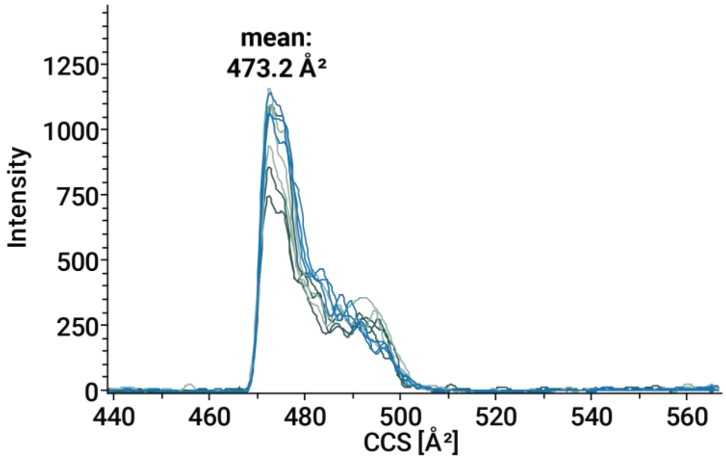

Fig. S82: LC-TIMS-MS mobilogram of the monoisotopic [M+2H]$^{2+}$ ion (m/z 1061.0286 ± 0.1) after the maturation assay of McjA with Tyr(O-Me) (2'). Δ6 voltage set to 150 V (blue), 100 V (light green), and 50 V (dark green).

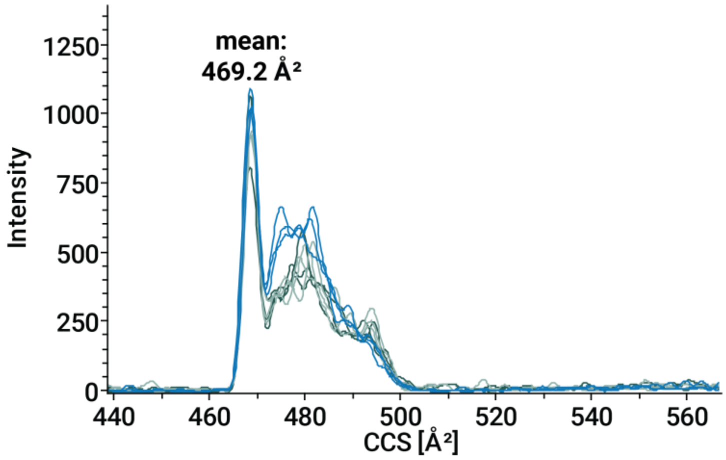

Fig. S83: LC-TIMS-MS mobilogram of the monoisotopic [M+2H]$^{2+}$ ion (m/z 1055.0164 ± 0.1) after the maturation assay of McjA with Phe(4-F) (3'). Δ6 voltage set to 150 V (blue), 100 V (light green), and 50 V (dark green).

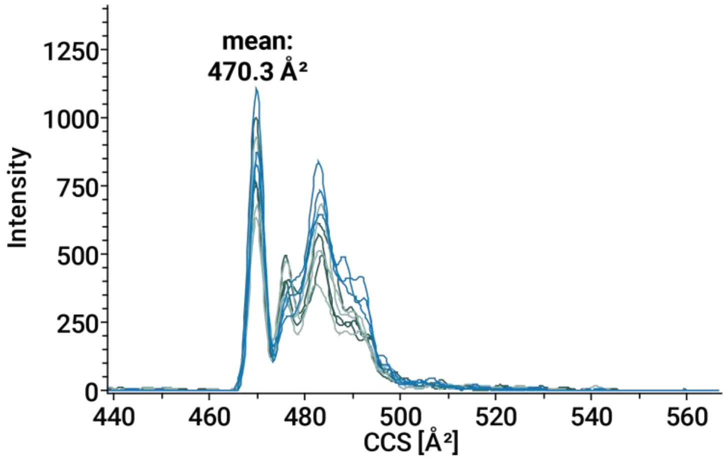

Fig. S84: LC-TIMS-MS mobilogram of the monoisotopic [M+2H]$^{2+}$ ion (m/z 1076.5118 ± 0.1) after the maturation assay of McjA with Tyr(3-NO_2) (4'). Δ6 voltage set to 150 V (blue), 100 V (light green), and 50 V (dark green).

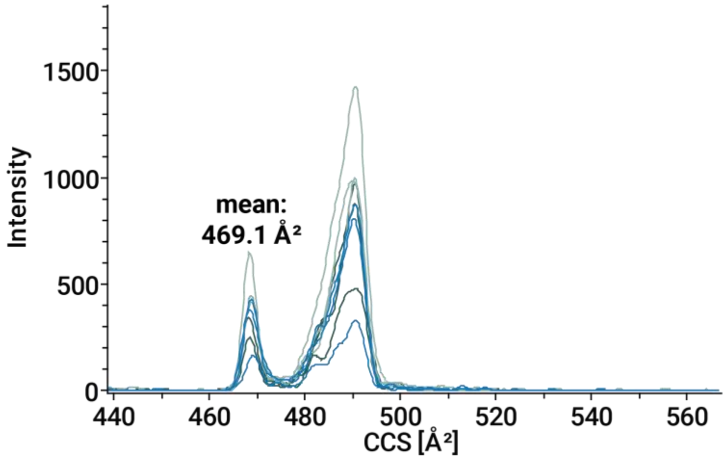

Fig. S85: LC-TIMS-MS mobilogram of the monoisotopic [M+2H]$^{2+}$ ion (m/z 1053.5284 ± 0.1) after the maturation assay of McjA with Phe(4-NH_2) (5'). Δ6 voltage set to 150 V (blue), 100 V (light green), and 50 V (dark green).

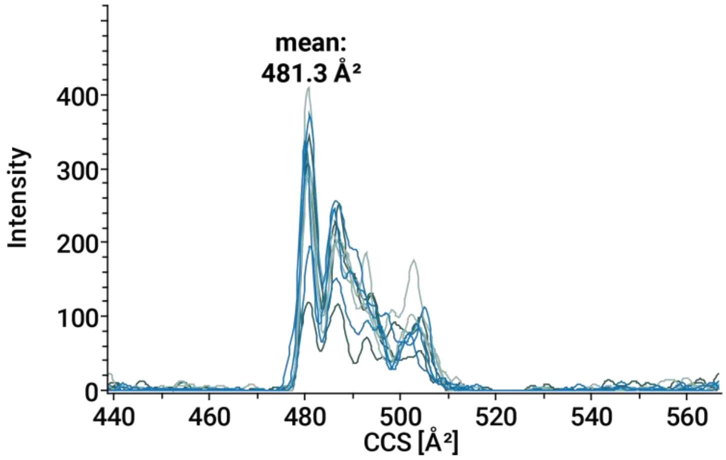

Fig. S86: LC-TIMS-MS mobilogram of the monoisotopic [M+2H]$^{2+}$ ion (m/z 1082.0514 ± 0.1) after the maturation assay of McjA with Tyr(3-*t*Bu) (6'). Δ6 voltage set to 150 V (blue), 100 V (light green), and 50 V (dark green).

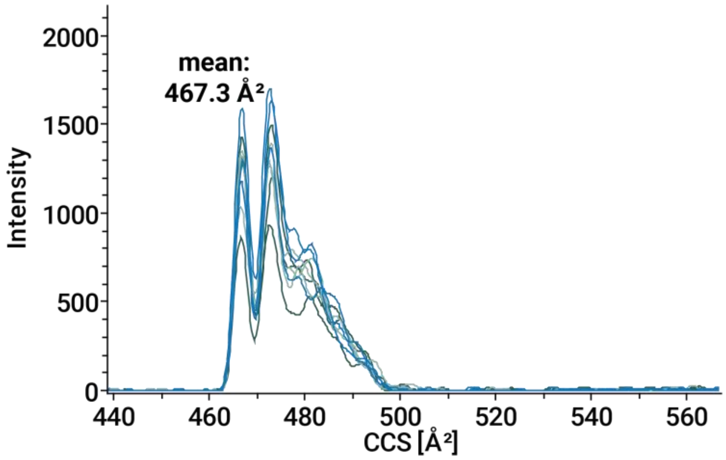

Fig. S87: LC-TIMS-MS mobilogram of the monoisotopic [M+2H]$^{2+}$ ion (m/z 1054.0187 ± 0.1) after the maturation assay of McjA with Phe(3-OH) (7'). Δ6 voltage set to 150 V (blue), 100 V (light green), and 50 V (dark green).

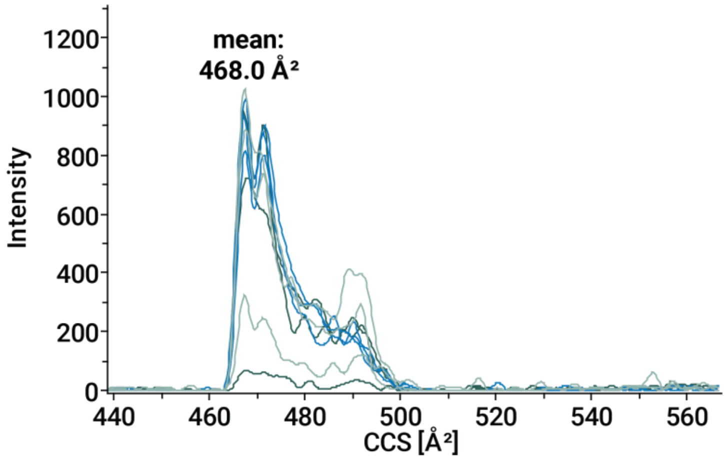

Fig. S88: LC-TIMS-MS mobilogram of the monoisotopic [M+2H]$^{2+}$ ion (m/z 1046.0230 ± 0.1) after the maturation assay of McjA with Phe (8'). Δ6 voltage set to 150 V (blue), 100 V (light green), and 50 V (dark green).

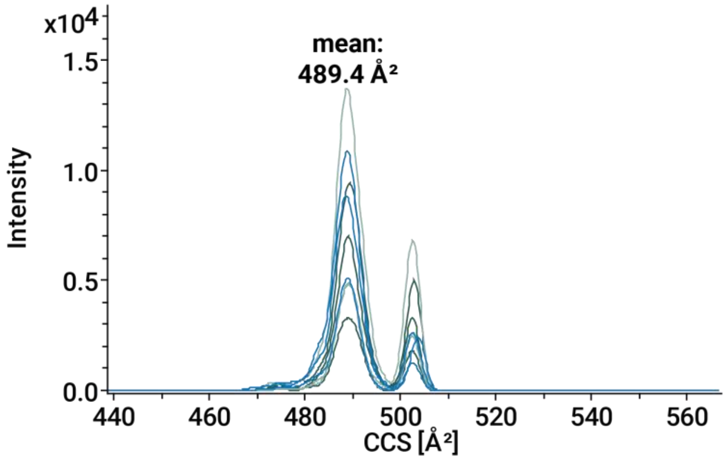

Fig. S89: LC-TIMS-MS mobilogram of the monoisotopic [M+2H]$^{2+}$ ion (m/z 1054.0197 ± 0.1) of WT-branched-cyclic peptide (bc-1'). Δ6 voltage set to 150 V (blue), 100 V (light green), and 50 V (dark green).

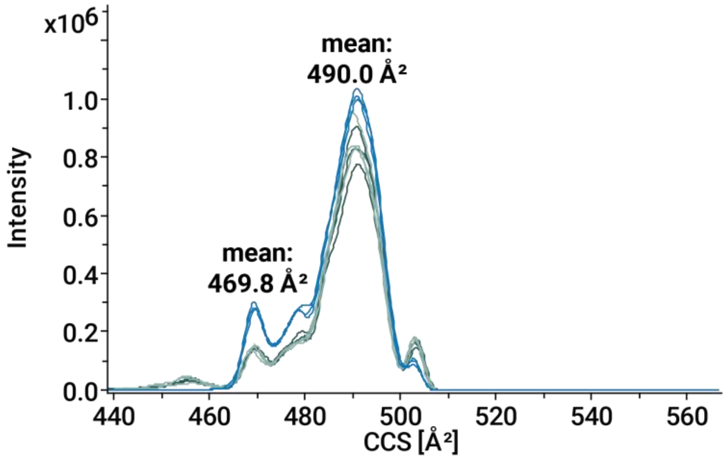

Fig. S90: LC-TIMS-MS mobilogram of the monoisotopic $[M+2H]^{2+}$ ion (m/z 1053.5264 ± 0.1) of branched-cyclic peptide with Phe(4-NH_2) (bc-5'). Δ6 voltage set to 150 V (blue), 100 V (light green), and 50 V (dark green).

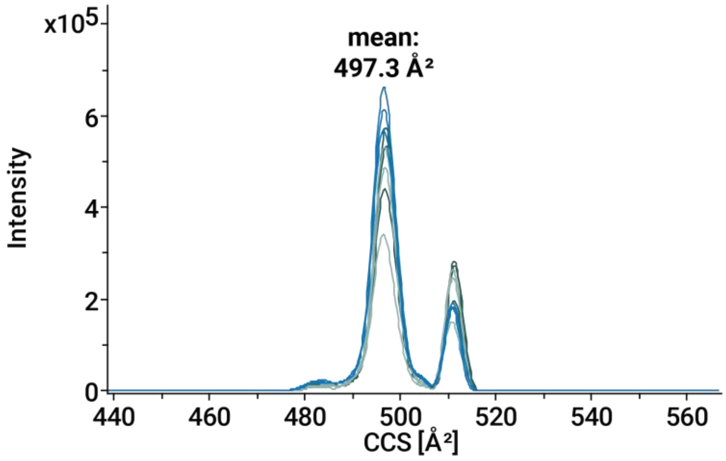

Fig. S91: LC-TIMS-MS mobilogram of the monoisotopic $[M+2H]^{2+}$ ion (m/z 1082.0497 ± 0.1) of branched-cyclic peptide with Tyr(3-*t*Bu) (bc-6'). Δ6 voltage set to 150 V (blue), 100 V (light green), and 50 V (dark green).

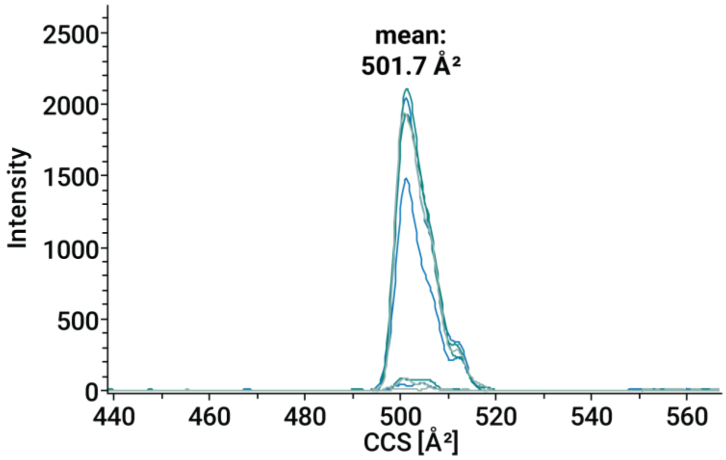

Fig. S92: LC-TIMS-MS mobilogram of the monoisotopic $[M+2H]^{2+}$ ion (m/z 1117.0467 ± 0.1) after the maturation assay of Link-McjA with L-His (9'). Δ6 voltage set to 150 V (blue), 100 V (light green), and 50 V (dark green).

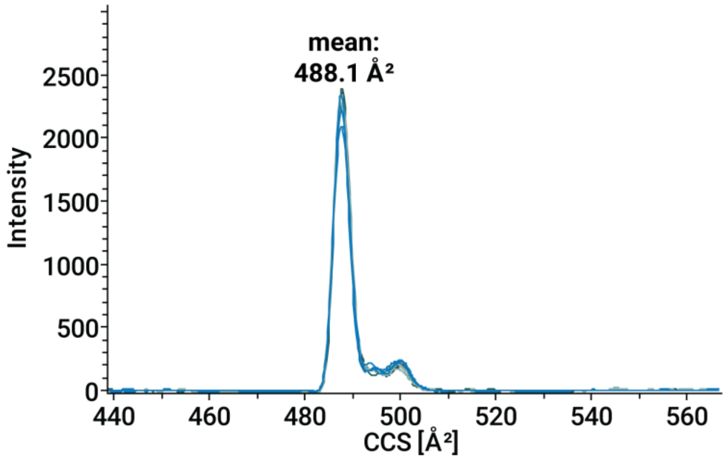

Fig. S93: LC-TIMS-MS mobilogram of the monoisotopic $[M+2H]^{2+}$ ion (m/z 1117.0469 ± 0.1) after the maturation assay of Link-McjA with D-His (10'). Δ6 voltage set to 150 V (blue), 100 V (light green), and 50 V (dark green).

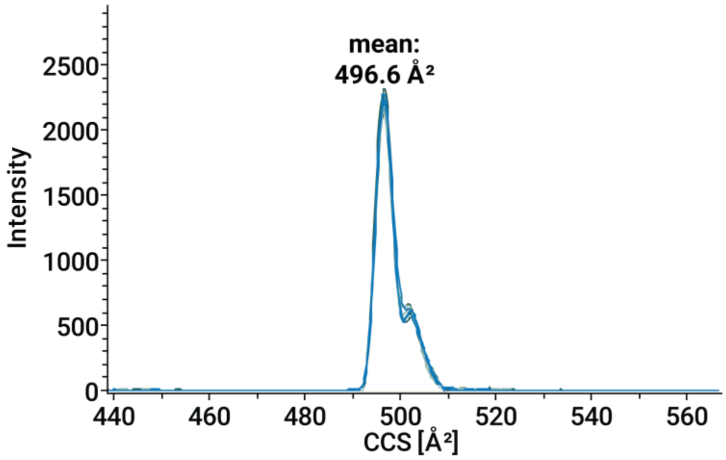

Fig. S94: LC-TIMS-MS mobilogram of the monoisotopic $[M+2H]^{2+}$ ion (m/z 1125.5286 ± 0.1) after the maturation assay of Link-McjA with Ala(4-Thz) (11'). Δ6 voltage set to 150 V (blue), 100 V (light green), and 50 V (dark green).

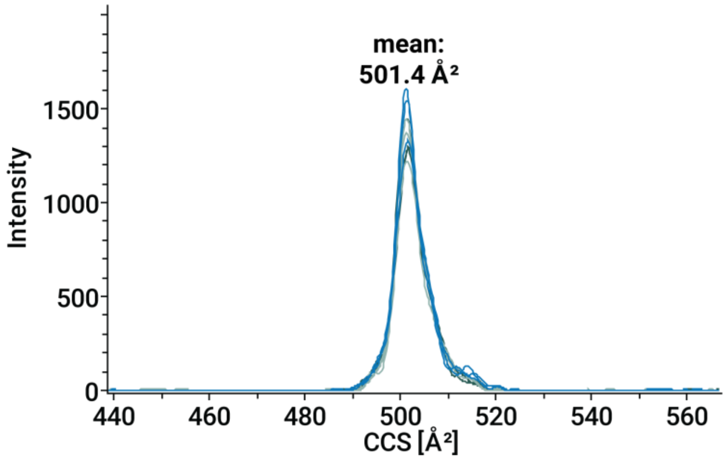

Fig. S95: LC-TIMS-MS mobilogram of the monoisotopic $[M+2H]^{2+}$ ion (m/z 1125.0315 ± 0.1) after the maturation assay of Link-McjA with Ala(3-Thi) (12'). Δ6 voltage set to 150 V (blue), 100 V (light green), and 50 V (dark green).

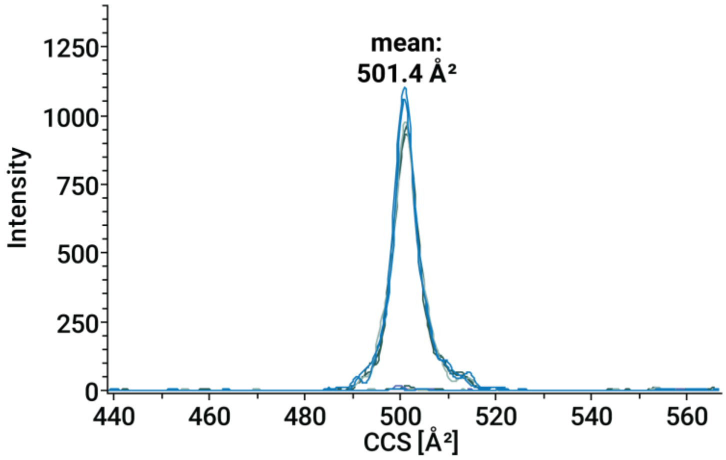

Fig. S96: LC-TIMS-MS mobilogram of the monoisotopic [M+2H]$^{2+}$ ion (m/z 1125.0304 ± 0.1) after the maturation assay of Link-McjA with Ala(2-Thi) (13'). Δ6 voltage set to 150 V (blue), 100 V (light green), and 50 V (dark green).

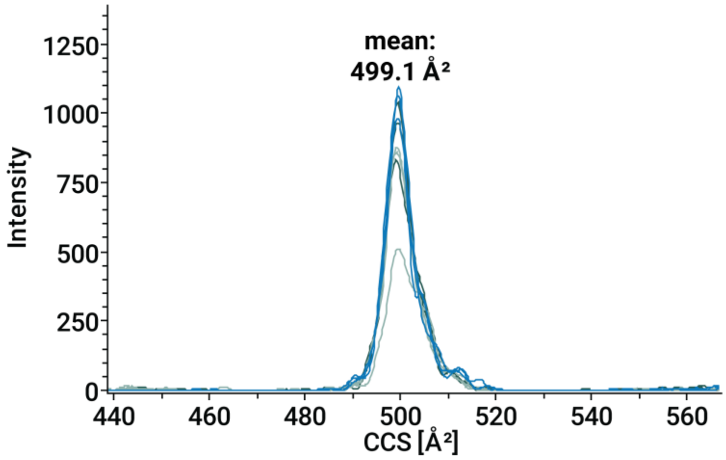

Fig. S97: LC-TIMS-MS mobilogram of the monoisotopic [M+2H]$^{2+}$ ion (m/z 1117.0474 ± 0.1) after the maturation assay of Link-McjA with Ala(2-Furyl) (14'). Δ6 voltage set to 150 V (blue), 100 V (light green), and 50 V (dark green).

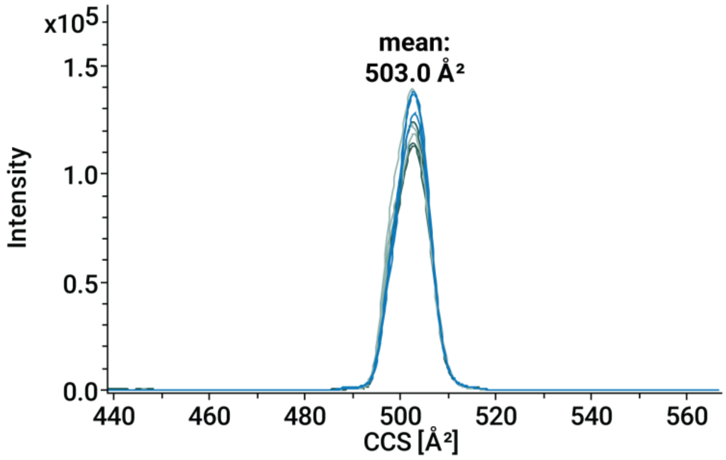

Fig. S98: LC-TIMS-MS mobilogram of the monoisotopic [M+2H]$^{2+}$ ion (m/z 1117.0476 ± 0.1) of Link-branched-cyclic peptide with L-His (bc-9'). Δ6 voltage set to 150 V (blue), 100 V (light green), and 50 V (dark green).

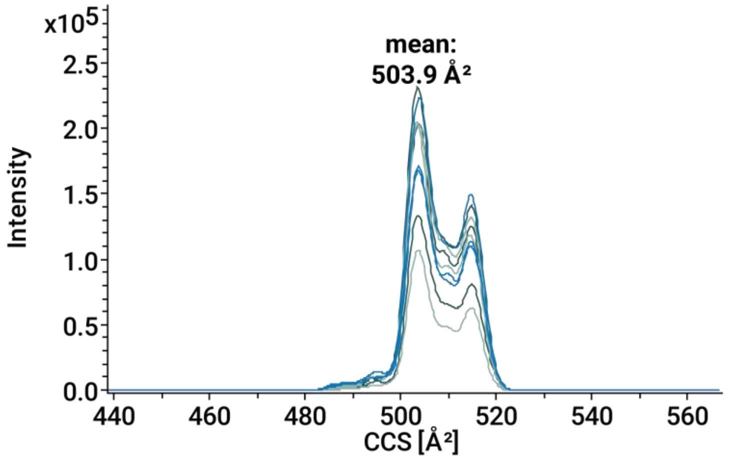

Fig. S99: **LC-TIMS-MS mobilogram of the monoisotopic [M+2H]$^{2+}$ ion (m/z 1117.0474 ± 0.1) of Link-branched-cyclic peptide with D-His (bc-10').** Δ6 voltage set to 150 V (blue), 100 V (light green), and 50 V (dark green).

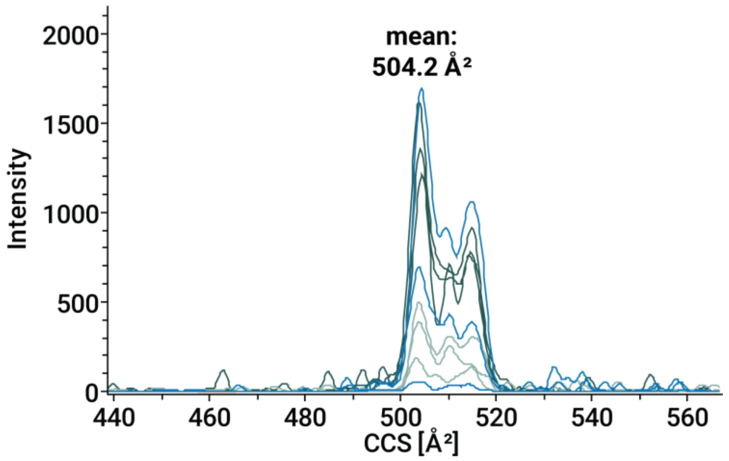

Fig. S100: **LC-TIMS-MS mobilogram of the monoisotopic [M+2H]$^{2+}$ ion (m/z 1117.0470 ± 0.1) after the maturation assay of Link-McjA D-His-12, D-Phe-13, and D-Ile-15 (15'A).** LC retention time: 3.80-4.00 min. Δ6 voltage set to 150 V (blue), 100 V (light green), and 50 V (dark green).

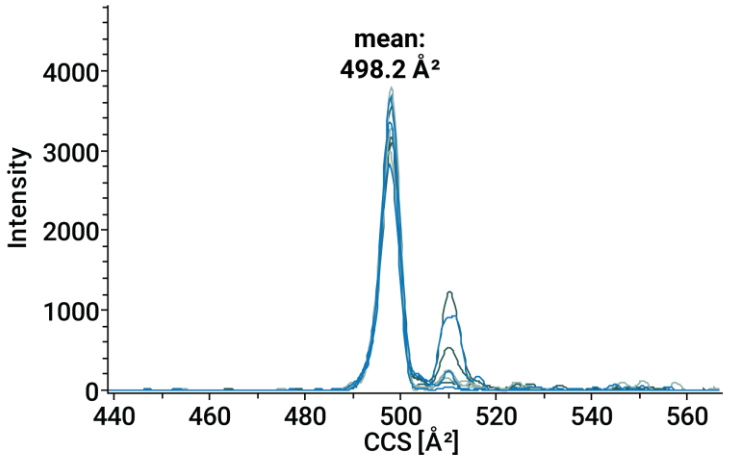

Fig. S101: **LC-TIMS-MS mobilogram of the monoisotopic [M+2H]$^{2+}$ ion (m/z 1117.0469 ± 0.1) after the maturation assay of Link-McjA with D-His-12, D-Phe-13, and D-Ile-15 (15'B).** LC retention time: 4.20-4.30 min. Δ6 voltage set to 150 V (blue), 100 V (light green), and 50 V (dark green).

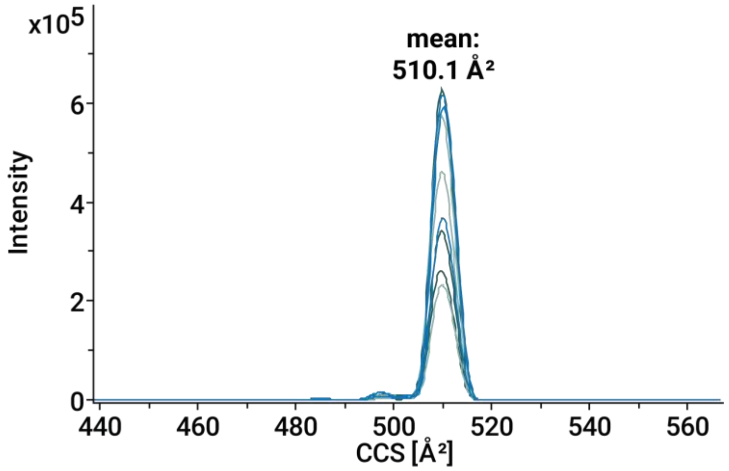

Fig. S102: **LC-TIMS-MS mobilogram of the monoisotopic [M+2H]$^{2+}$ ion (m/z 1117.0472 ± 0.1) of Link-branched-cyclic peptide with D-His-12, D-Phe-13, and D-Ile-15 (bc-15').** Δ6 voltage set to 150 V (blue), 100 V (light green), and 50 V (dark green).

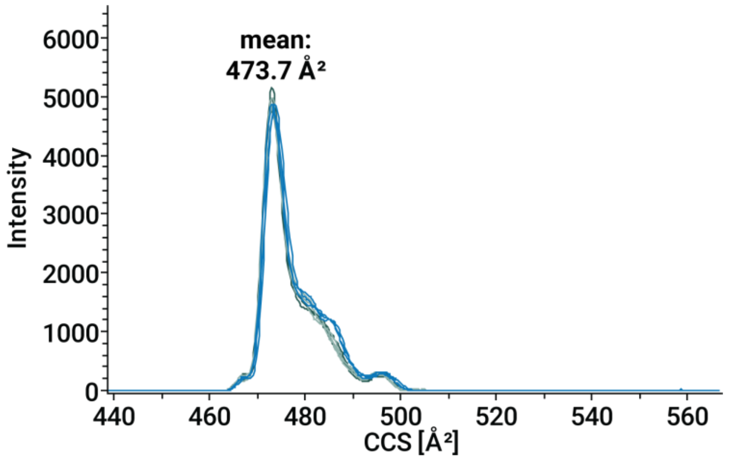

Fig. S103: **LC-TIMS-MS mobilogram of the monoisotopic [M+2H]$^{2+}$ ion (m/z 1061.0268 ± 0.1) after the maturation assay of WT-McjA with N-Me-Gly12 (16).** Δ6 voltage set to 150 V (blue), 100 V (light green), and 50 V (dark green).

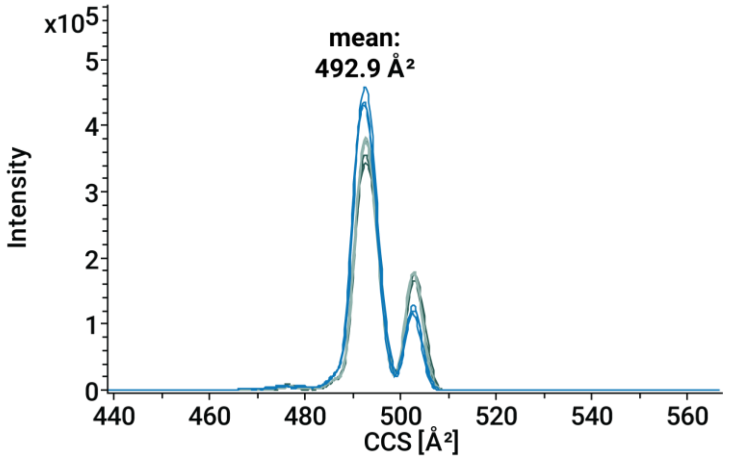

Fig. S104: **LC-TIMS-MS mobilogram of the monoisotopic [M+2H]$^{2+}$ ion (m/z 1061.0276 ± 0.1) of branched-cyclic peptide with N-Me-Gly12 (bc-16').** Δ6 voltage set to 150 V (blue), 100 V (light green), and 50 V (dark green).

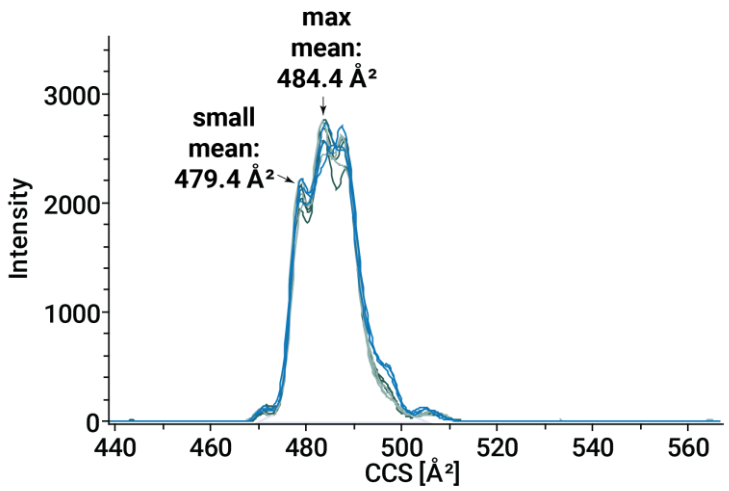

Fig. S105: **LC-TIMS-MS mobilogram of the monoisotopic [M+2H]$^{2+}$ ion (m/z 1068.0336 ± 0.1) after the maturation assay of WT-McjA with N-Me-Gly12 and N-Me-Gly14 (17).** Δ6 voltage set to 150 V (blue), 100 V (light green), and 50 V (dark green).

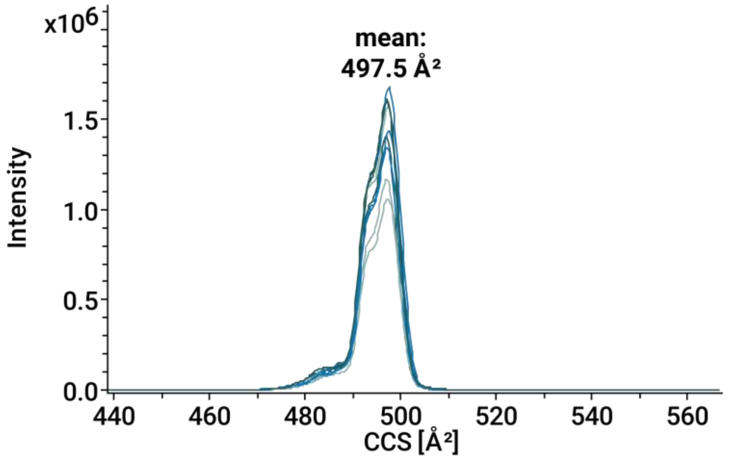

Fig. S106: **LC-TIMS-MS mobilogram of the monoisotopic [M+2H]$^{2+}$ ion (m/z 1068.0346 ± 0.1) of branched-cyclic peptide with N-Me-Gly12 and N-Me-Gly14 (bc-17').** Δ6 voltage set to 150 V (blue), 100 V (light green), and 50 V (dark green).

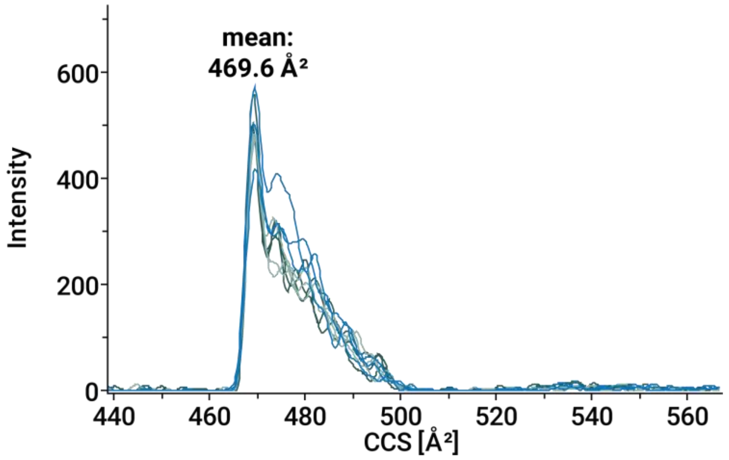

Fig. S107: **LC-TIMS-MS mobilogram of the monoisotopic [M+2H]$^{2+}$ ion (m/z 1054.0178 ± 0.1) after the maturation assay of McyA-leader with McjA-core forming MccJ25 (18').** Δ6 voltage set to 150 V (blue), 100 V (light green), and 50 V (dark green).

For McyA, the conversion was too low to obtain CCS values via LC-TIMS-MS. Therefore, only extracted ion chromatograms for all nine injections were analyzed. All resulted in similar EICs, therefore, only one trace is shown.

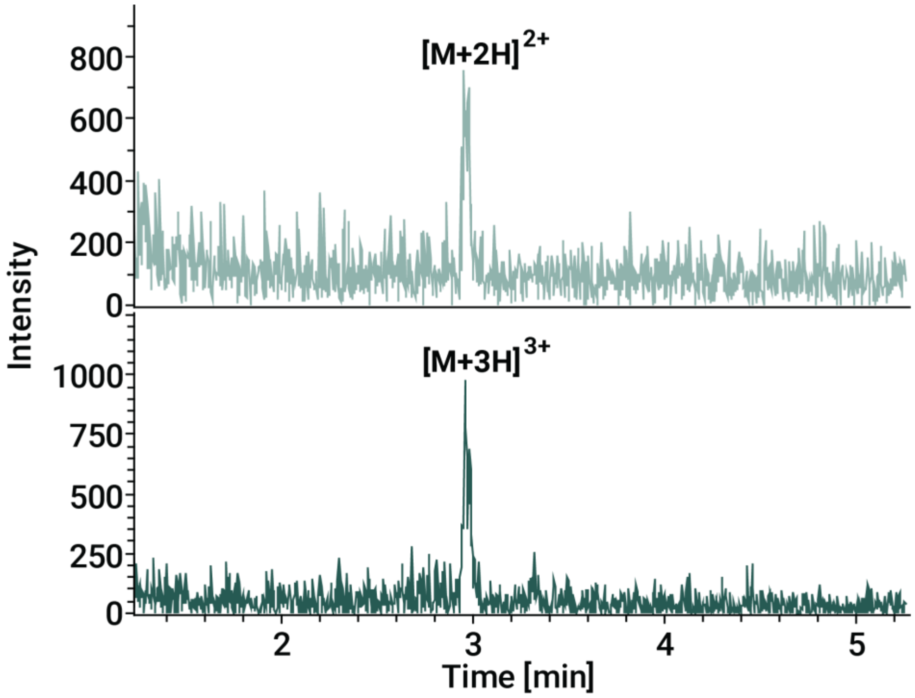

Fig. S108: LC-MS analysis of formation of MccY from maturation of McyA. Top: $[M+2H]^{2+}$ ion (m/z 1113.0424 ± 0.1) in light green. Bottom: Top: $[M+2H]^{2+}$ ion (m/z 742.3640 ± 0.1) in dark green.

4.5 Thermolysin digestion of MccJ25 and LC-MS analysis thereof

4.5.1 Thermolysin protocol for MccJ25 and branched-cyclic peptide standards

The standard protocol described in literature[7] was adjusted regarding samples from maturation assay. The peptide was dissolved in Tris buffer (50 mM, pH 8) and equal amount of 8 M urea to reach a final concentration of around 0.83 µM. The peptide was incubated at 46 °C for 30 minutes. Twice the volume of NH_4HCO_3 solution (0.17 M) and 1/15 of the volume of Tris buffer of a $CaCl_2$ solution (50 mM) were added and supplemented with thermolysin (40 µg/µmol of peptide). The reaction proceeds for 60 minutes at 46 °C. Afterwards, acetic acid (1.5 times the amount of initial Tris buffer) was added to stop the reaction. The peptide was analyzed by LC-MS (Section 1.7). Controls were set up without thermolysin treatment.

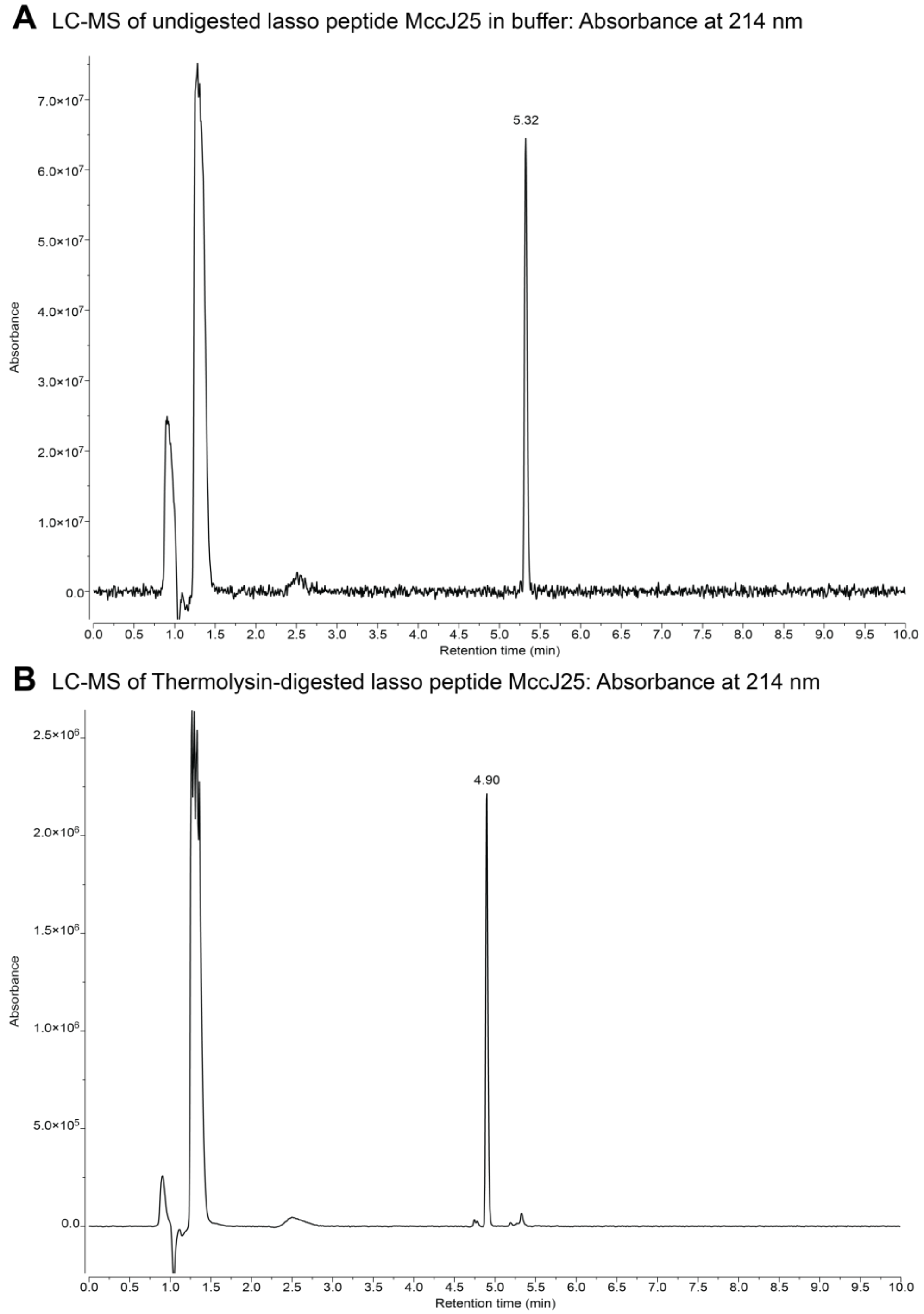

C LC-MS of Thermolysin-digested lasso peptide MccJ25: HRMS spectrum

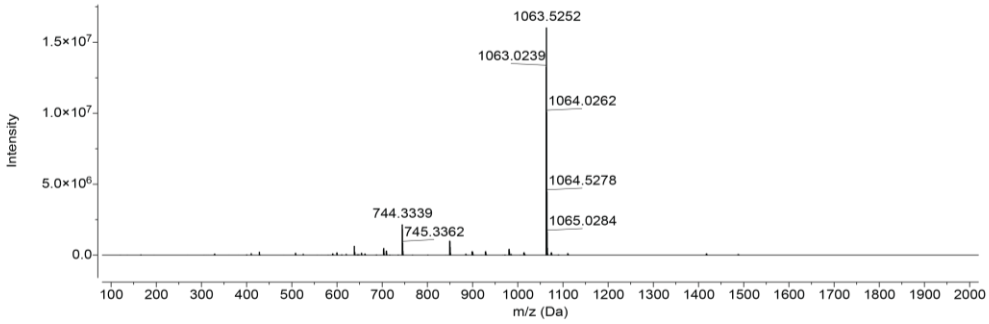

D LC-MS of Thermolysin-digested lasso peptide MccJ25: deconvoluted MS spectrum

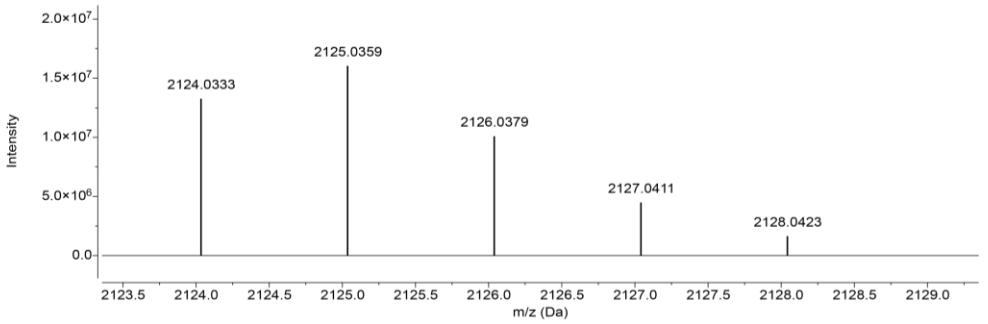

Fig. S109: LC-MS analysis of thermolysin digestion of expressed wild-type lasso peptide MccJ25 forming a stable peptide [2]-rotaxane: (A) LC chromatogram of undigested peptide in buffer analyzed at 214 nm. (B) LC chromatogram of thermolysin-digested peptide in buffer analyzed at 214 nm. (C) MS spectrum of thermolysin-digested peptide ([2]-rotaxane). (D) deconvoluted MS spectrum: Monoisotopic mass (ESI+): calc. [$C_{101}H_{141}N_{23}O_{28}$]: 2124.0316, found: 2124.0333; Average mass calc. [$C_{101}H_{141}N_{23}O_{28}$]: 2125.3720.

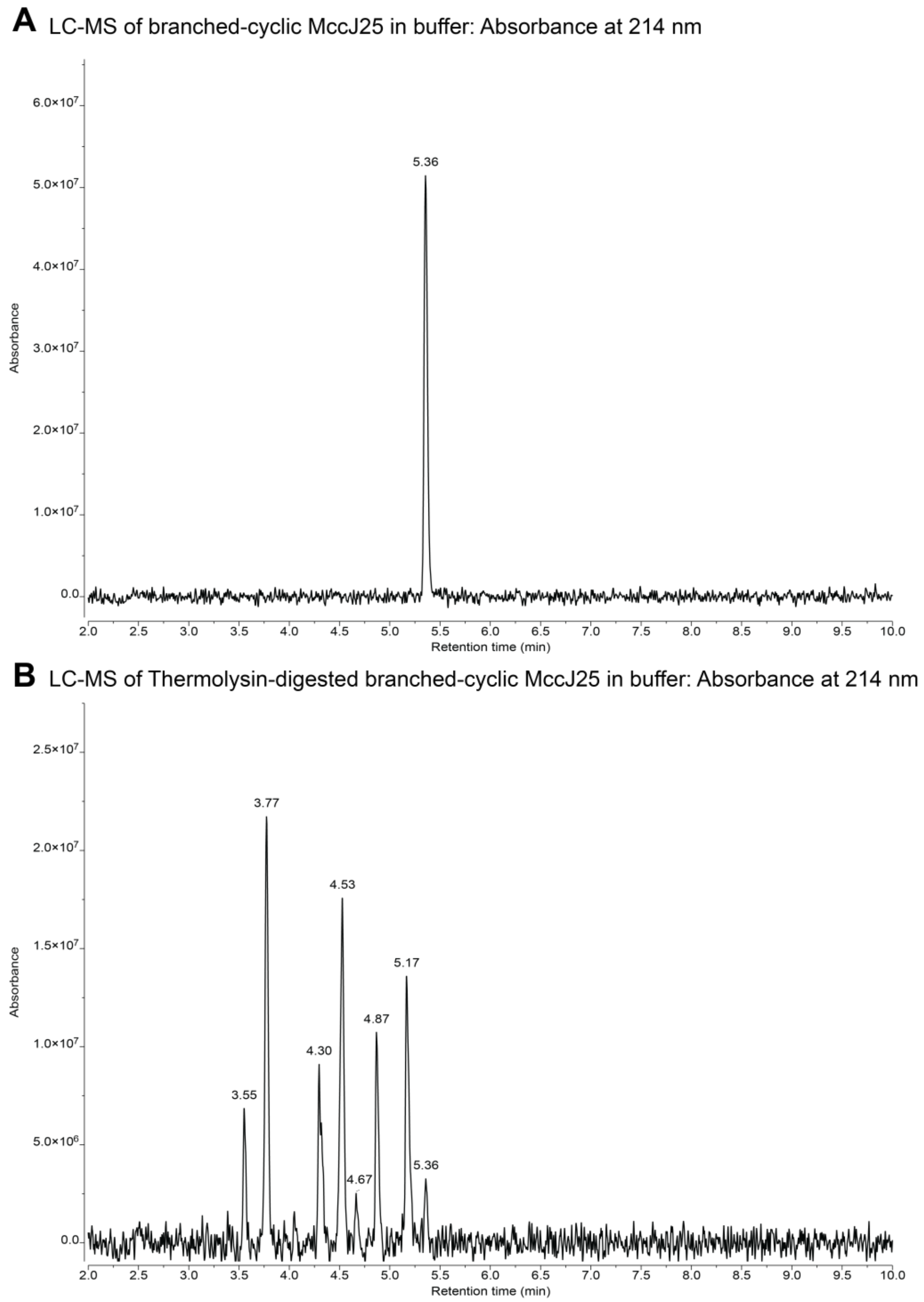

Fig. S110: LC-MS (UV) analysis of thermolysin digestion of wild-type branched-cyclic MccJ25 (bc-1') at 214 nm: (A) LC chromatogram of undigested peptide. (B) LC chromatogram of thermolysin-digested peptide.

A LC-MS of branched-cyclic MccJ25 with Y9 mutation to Phe(4-NH_2) in buffer: Absorbance at 214 nm

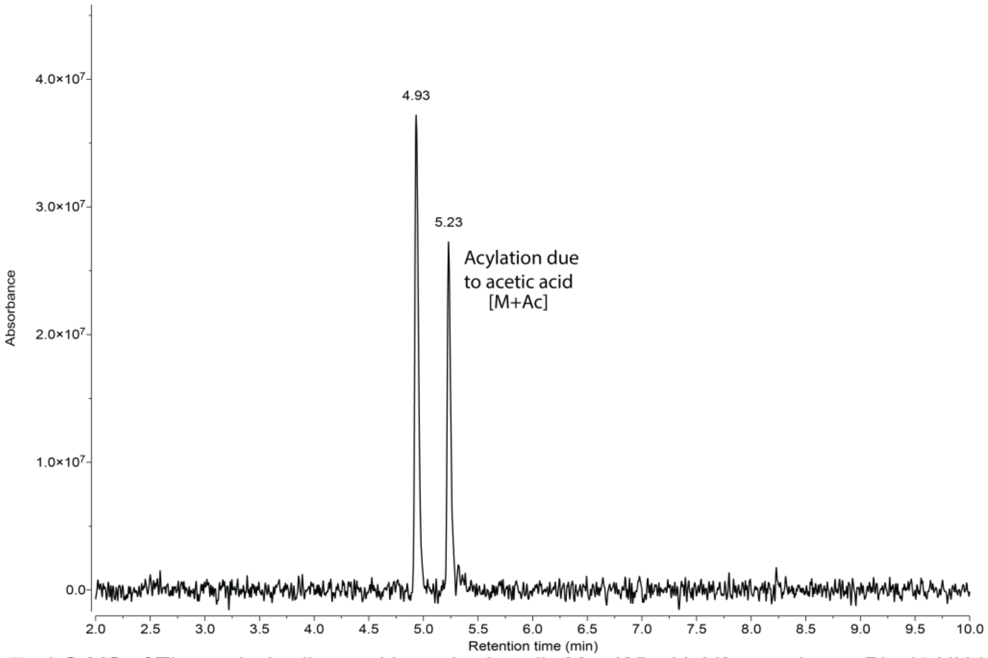

B LC-MS of Thermolysin-digested branched-cyclic MccJ25 with Y9 mutation to Phe(4-NH_2) in buffer: Absorbance at 214 nm

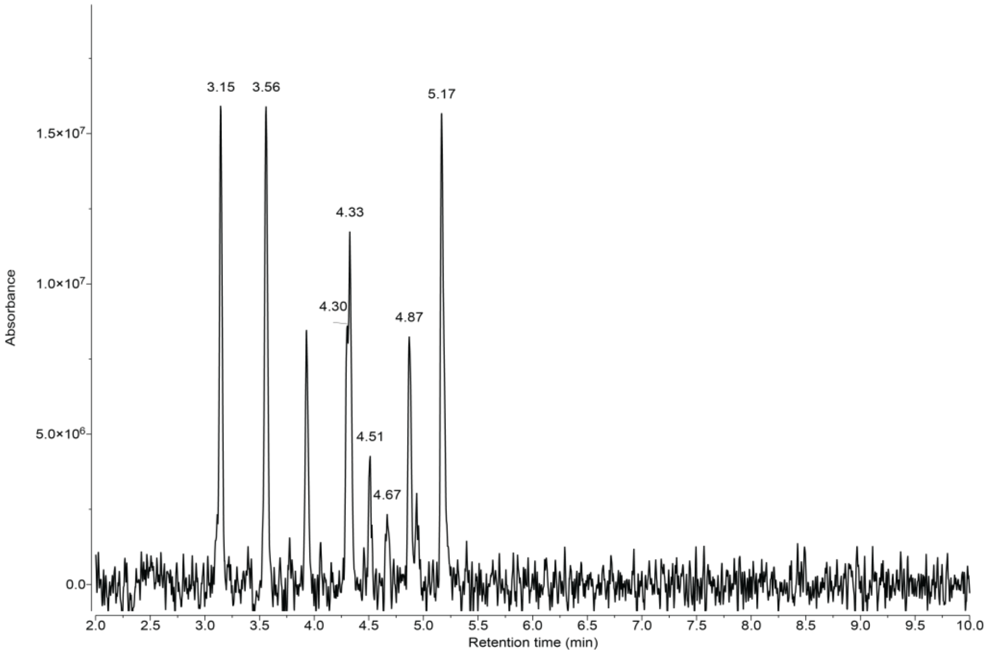

Fig. S111: LC-MS (UV) analysis of thermolysin digestion of wild-type branched-cyclic MccJ25 with Y9 mutation to Phe(4-NH_2) (bc-5') at 214 nm: (A) LC chromatogram of undigested peptide. (B) LC chromatogram of thermolysin-digested peptide.

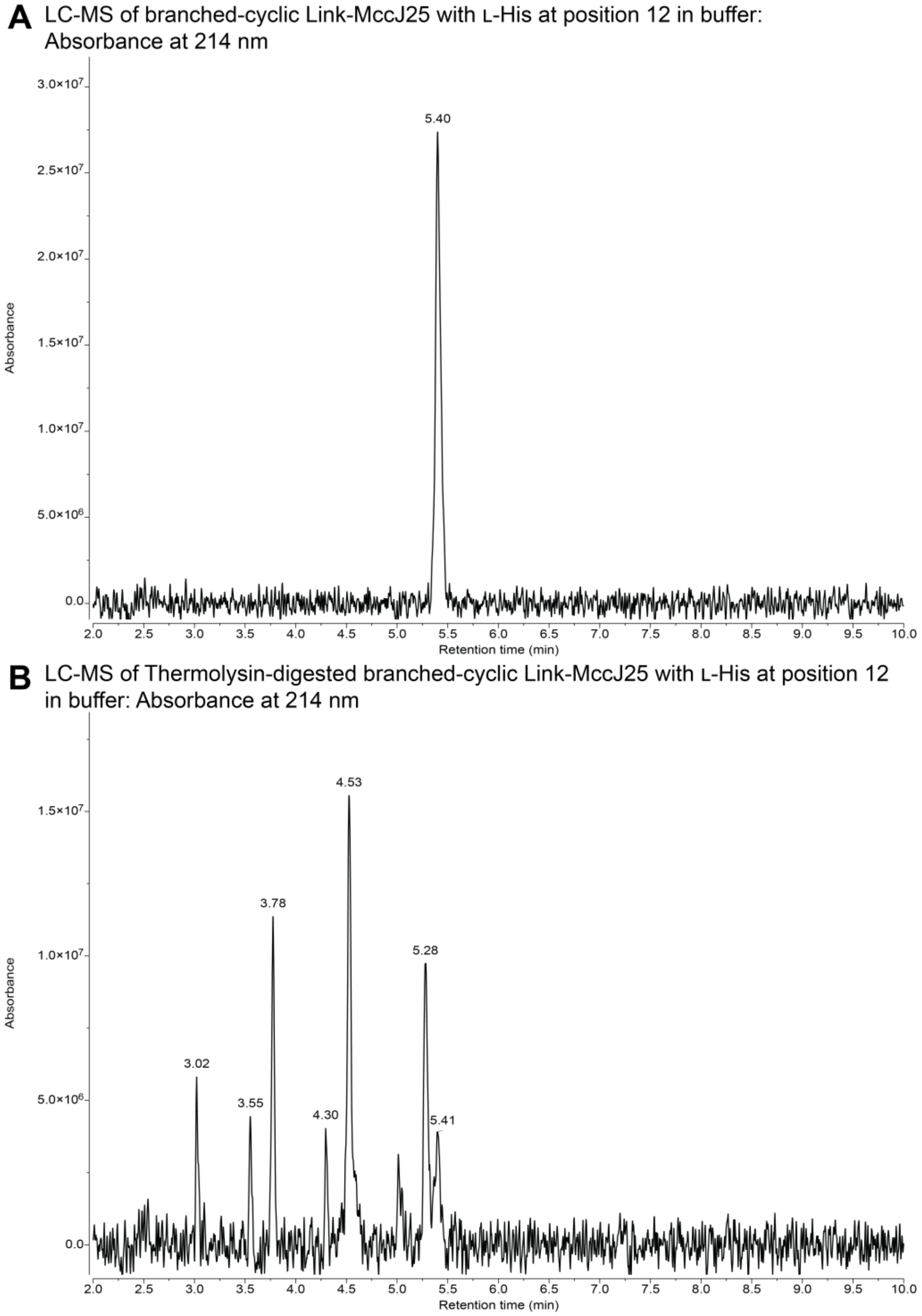

Fig. S112: LC-MS (UV) analysis of thermolysin digestion of branched-cyclic Link-MccJ25 with L-His at position 12 (bc-9') at 214 nm: (A) LC chromatogram of undigested peptide. (B) LC chromatogram of thermolysin-digested peptide.

A LC-MS of branched-cyclic Link-MccJ25 with D-His at position 12 in buffer: Absorbance at 214 nm

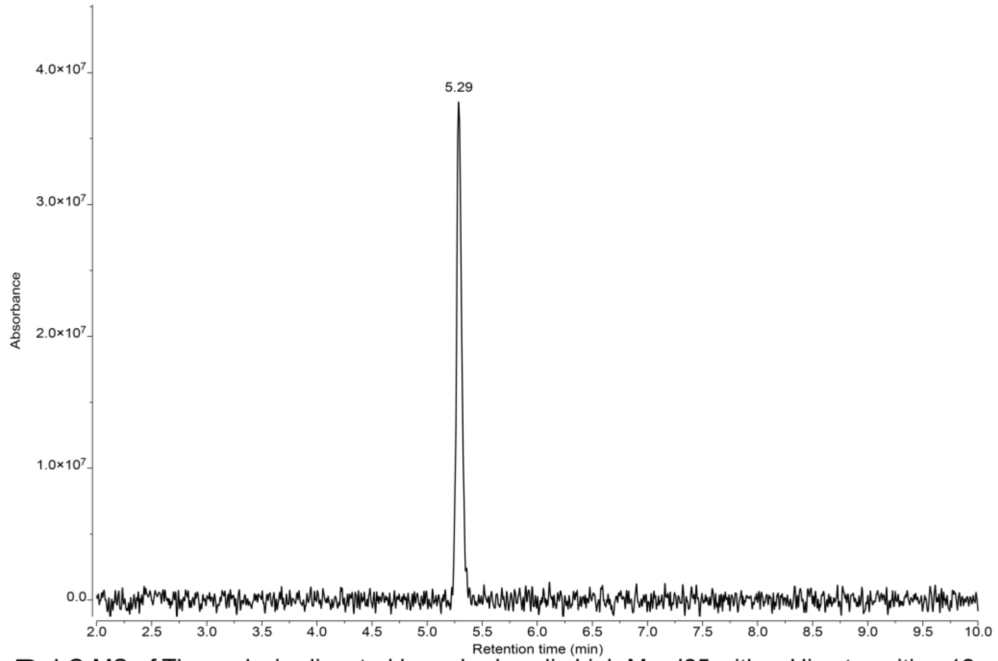

B LC-MS of Thermolysin-digested branched-cyclic Link-MccJ25 with D-His at position 12 in buffer: Absorbance at 214 nm

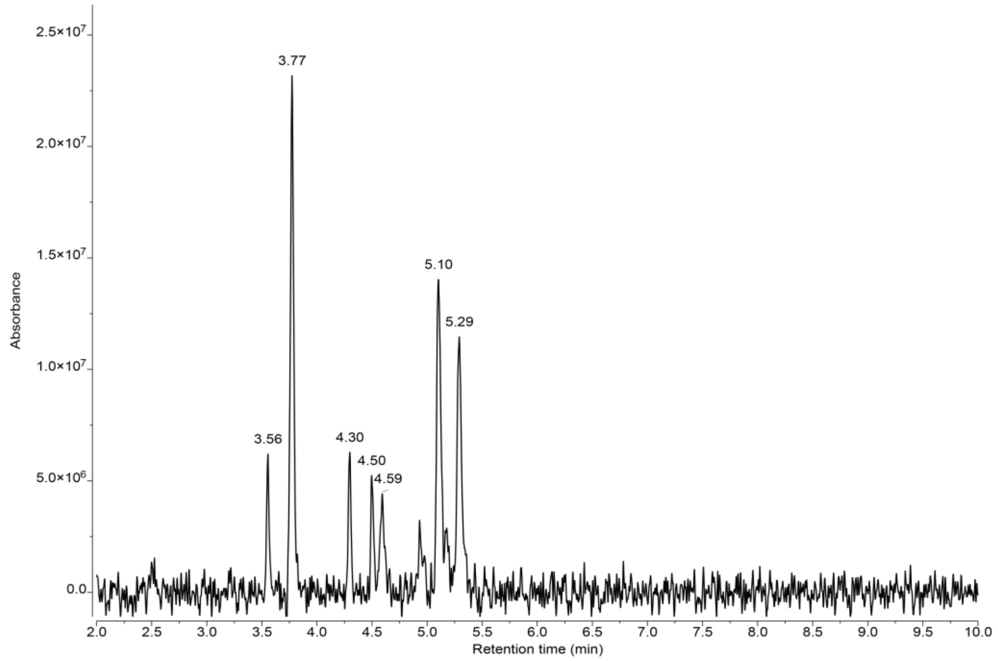

Fig. S113: LC-MS (UV) analysis of thermolysin digestion of branched-cyclic Link-MccJ25 with D-His at position 12 (bc-10') at 214 nm: (A) LC chromatogram of undigested peptide. (B) LC chromatogram of thermolysin-digested peptide.

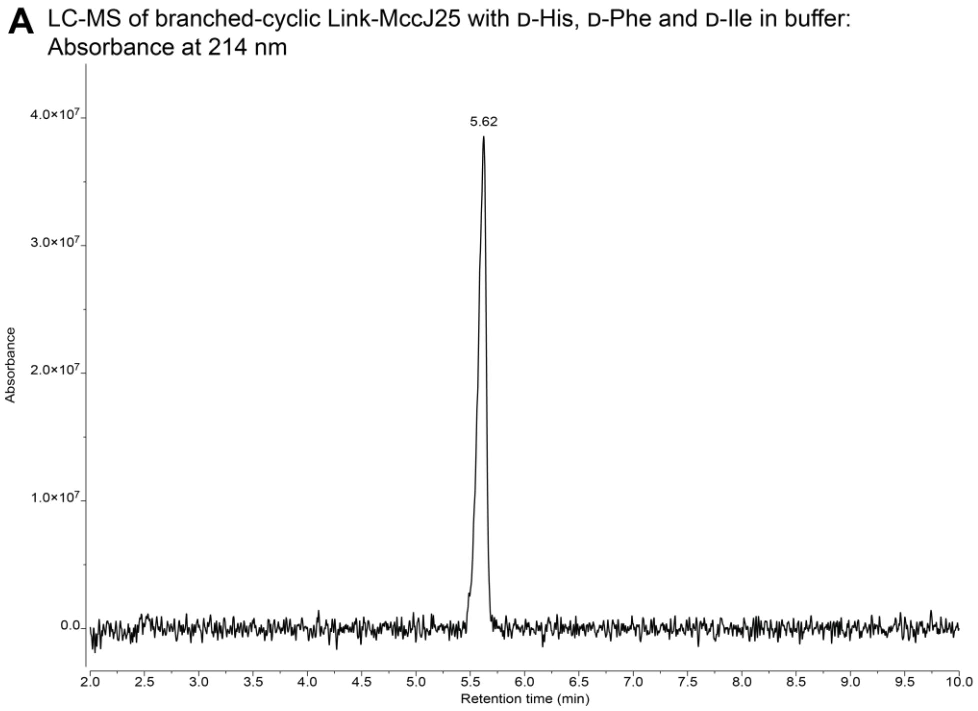

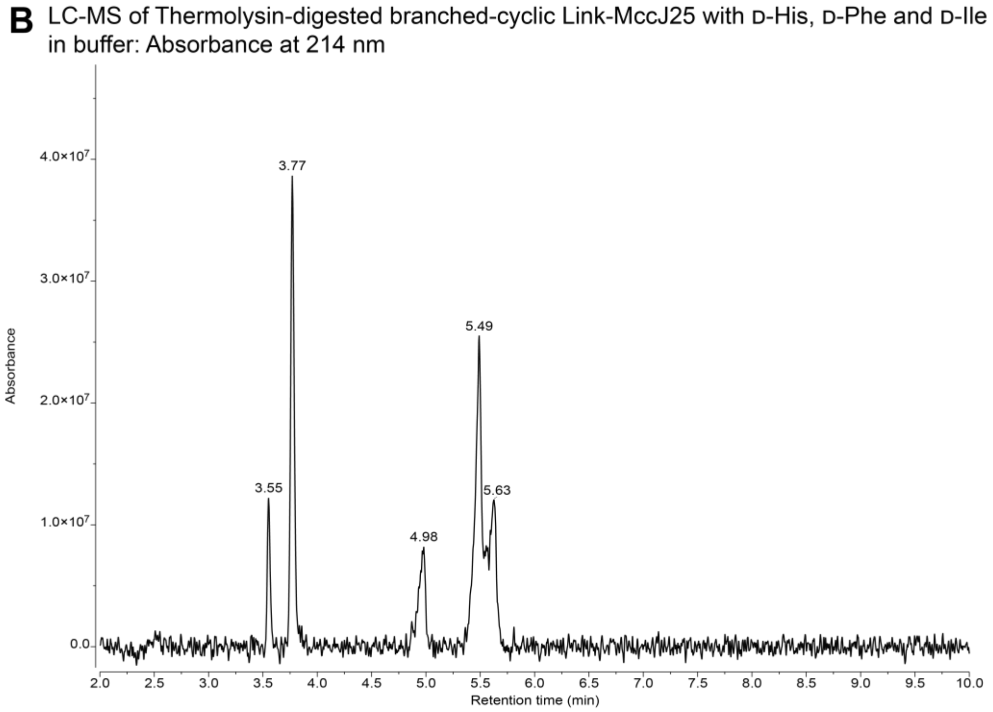

Fig. S114: LC-MS (UV) analysis of thermolysin digestion of branched-cyclic Link-MccJ25 with D-His-12, D-Phe-13, and D-Ile-15 (bc-15') at 214 nm: (A) LC chromatogram of undigested peptide. (B) LC chromatogram of thermolysin-digested peptide.

4.5.2 Analysis of MccJ25 derivatives after maturation

The samples from maturation (12 µL) were dried and prepared by following the protocol in 3.5.2 (dissolved in Tris-buffer pH 8 (12 µL) with 8 M urea (12 µL). The samples were analyzed by LC-MS to confirm lasso conformation via the formation of 2-rotaxane ([M+H_2O]) or branched-cyclic conformation (more fragmentation).

For the tested lasso peptides, the corresponding mass of the cleaved 2-rotaxanes was found. Therefore, the peptides were analyzed regarding CCS values via LC-IM-MS.

Table S10: CCS-values [Å^2] of the peak with the smallest value in the mobilogram after thermolysin digestion.

	100 V			mean	std.
**lasso-M**	484.4	484.6	484.8	484.6	$0{,}2_0$
**lasso-S**	473.8	474.3	474.0	474.0	$0{,}2_5$
**1'-M**	488.3	487.0	484.9	486.7	$1{,}7_1$
**1'-S**	477.7	477.2	477.5	477.5	$0{,}2_5$
**5'-M**	486.6	486.7	487.2	486.8	$0{,}3_2$
**5'-S**	473.1	473.6	473.5	473.4	$0{,}2_6$

Legend: **M+S:** Two peaks in the mobilogram: **M**: main peak, **S**: smallest CCS value

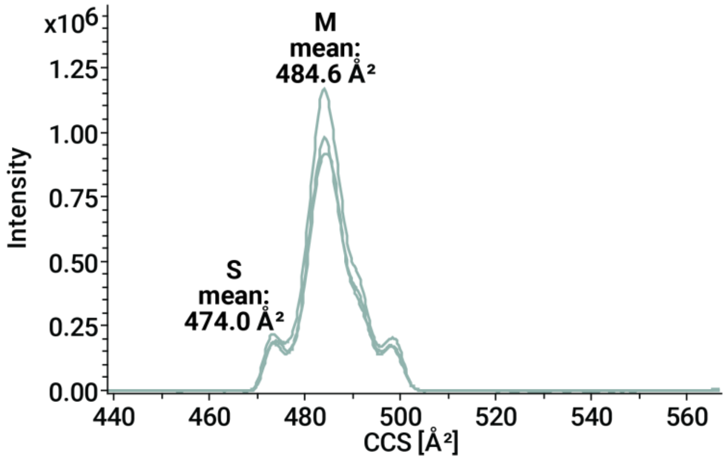

Fig. S115: LC-TIMS-MS mobilogram of the monoisotopic [M+2H]$^{2+}$ ion (m/z 1063.0236 ± 0.1) after thermolysin digestion of MccJ25 lasso standard (entry I). Δ6 voltage: 100 V.

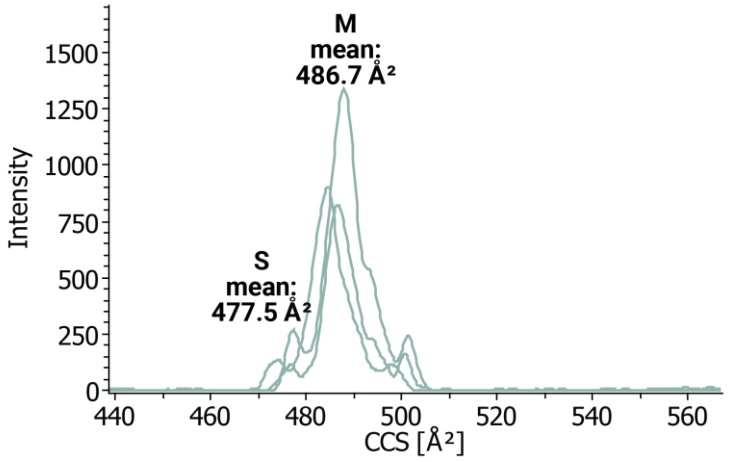

Fig. S116: LC-TIMS-MS mobilogram of the monoisotopic $[M+2H]^{2+}$ ion (m/z 1063.0234 ± 0.1) after thermolysin digestion of maturation assay of WT-McjA (entry 1). Δ6 voltage: 100 V.

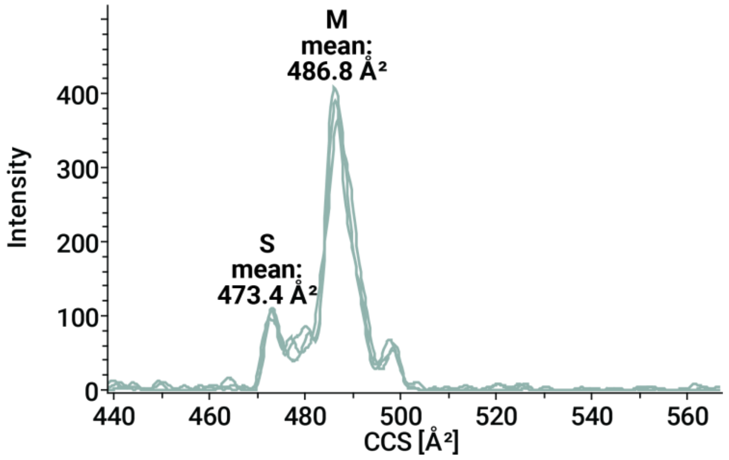

Fig. S117: LC-TIMS-MS mobilogram of the monoisotopic $[M+2H]^{2+}$ ion (m/z 1062.5328 ± 0.1)after thermolysin digestion of maturation assay of McjA with Phe(4-NH_2) (entry 5). Δ6 voltage: 100 V.

5. Literature

(1) Hartrampf, N.; Saebi, A.; Poskus, M.; Gates, Z. P.; Callahan, A. J.; Cowfer, A. E.; Hanna, S.; Antilla, S.; Schissel, C. K.; Quartararo, A. J.; et al. Synthesis of proteins by automated flow chemistry. *Science* **2020**, *368* (6494), 980-987.
(2) Dumon-Seignovert, L.; Cariot, G.; Vuillard, L. The toxicity of recombinant proteins in Escherichia coli: a comparison of overexpression in BL21 (DE3), C41 (DE3), and C43 (DE3). *Protein expression and purification* **2004**, *37* (1), 203-206.
(3) Yan, K. P.; Li, Y.; Zirah, S.; Goulard, C.; Knappe, T. A.; Marahiel, M. A.; Rebuffat, S. Dissecting the maturation steps of the lasso peptide microcin J25 in vitro. *Chembiochem* **2012**, *13* (7), 1046-1052.
(4) Gabelica, V.; Shvartsburg, A. A.; Afonso, C.; Barran, P.; Benesch, J. L.; Bleiholder, C.; Bowers, M. T.; Bilbao, A.; Bush, M. F.; Campbell, J. L. Recommendations for reporting ion mobility Mass Spectrometry measurements. *Mass spectrometry reviews* **2019**, *38* (3), 291-320.
(5) Pan, S. J.; Link, A. J. Sequence diversity in the lasso peptide framework: discovery of functional microcin J25 variants with multiple amino acid substitutions. *J Am Chem Soc* **2011**, *133* (13), 5016-5023.
(6) Assrir, N.; Pavelkova, A.; Dazzoni, R.; Ducasse, R.; Morellet, N.; Guittet, E.; Rebuffat, S.; Zirah, S.; Li, Y.; Lescop, E. Initial molecular recognition steps of McjA precursor during microcin J25 lasso peptide maturation. *Chembiochem* **2016**, *17* (19), 1851-1858.
(7) Rosengren, K. J.; Blond, A.; Afonso, C.; Tabet, J. C.; Rebuffat, S.; Craik, D. J. Structure of thermolysin cleaved microcin J25: extreme stability of a two-chain antimicrobial peptide devoid of covalent links. *Biochemistry* **2004**, *43* (16), 4696-4702.
